# The effectiveness of interventions used to improve general health check uptake by the older adult population: a systematic review and meta-analysis

**DOI:** 10.1371/journal.pgph.0004362

**Published:** 2025-03-31

**Authors:** Wing Yan Lau, Jinxiao Lian, Maurice Yap

**Affiliations:** Public Health Research Group, School of Optometry, The Hong Kong Polytechnic University, Hong Kong, SAR, China; SRMIST: SRM Institute of Science and Technology (Deemed to be University), INDIA

## Abstract

Undergoing general health check enable early detection of common diseases and giving individuals a sense of control over their wellbeing. However, the general health check uptakes are typically unsatisfactory. Various interventions have been introduced to improve general health check uptakes. This review aims to answer how well these interventions work. A comprehensive literature search was conducted in four electronic databases in August 2020 and updated between 2021 and 2024. Randomised controlled trials (RCTs) that met the inclusion criteria were selected. Meta-analysis was performed on qualified RCTs to estimate the overall effectiveness of the interventions. The components of intervention were characterised using the Behaviour Change Technique Taxonomy. A total of 3360 records were screened. Eight RCTs were finally included. Among these RCTs, nine types of interventions were identified with all implemented in the invitation stage, including enhanced invitation letters, telephone invitations, question-behaviour-effect (QBE) questionnaires, financial incentives, leaflets, pre-notification short message service (SMS), SMS reminders, reminder letters and point-of-care automated prompts to clinical staff. All these interventions showed a significant improvement in the general health check uptakes than the control groups, except leaflets and QBE questionnaires. A total of fifteen behaviour change techniques were used in these interventions. A meta-analysis showed the pooled effect of these interventions was significantly associated with the improvement in the general health check uptakes than the control (OR =1.30, 95% CI =1.15 – 1.46). However, the high heterogeneity observed (84%) could reduce the reliability of the pooled summary effect. This review found that interventions primarily implemented during the invitation process are effective in improving the general health check uptake rates. Future research should aim to extend these interventions beyond the invitation stage to address internal and external barriers that deter older adults from seeking general health checks. The systematic review protocol is registered on PROSPERO (ref: CRD42021221041).

## Introduction

The global population is ageing, with an estimated increase in the number of people over 65 years old from 703 million in 2019 to 1.5 billion in 2050 [1]. Concurrent with this growth is an increasing demand for healthcare services [2]. To address the associated costs and improve the health experience of older people, encouraging healthy behaviour and using appropriate preventive services are two common strategies adopted. General health checks, as one of the preventive services, provide an overall physical examination of multiple risk factors of diseases and allow timely modification of risky behaviour related to chronic illnesses and early disease detection [3,4].

Access to health check programmes varies in different jurisdictions. Some require users to pay out-of-pocket, while others are covered under national health insurance schemes or subsidised by the government. One universal health check programme which has been widely researched is the National Health Service (NHS) healthcare programme in the United Kingdom (UK). Launched in 2009, it offers general health check services to individuals aged 40 to 75 years with no pre-existing cardiovascular conditions. A recent study reported an overall uptake rate of 52% [5]. This programme was projected to improve long-term health outcomes in terms of reducing premature death and compressing morbidity [6]. Real-world evidence has shown that those who attended general health checks had lower BMI, systolic blood pressure and proportion of smoking compared to controls who did not attend [7].

Despite the health benefits and cost-saving potential [8], the uptake of general health checks remained relatively low, ranging from 43% to 48.2% among older people [9–12]. This indicates the need for additional strategies to improve the uptake of general health checks further.

Previous systematic reviews have identified several interventions that have shown potential to improve the uptake of general health checks [13–15]. However, the overall effectiveness of these interventions remains unknown. A previous meta-analysis of 21 studies (including randomised, non-randomised controlled trials and pre- and post-studies) provided an estimate of the overall effectiveness of the interventions to improve cardiovascular disease risk screening. However, it did not specifically target the older adult group (targeted 18 years or above) [13]. Another systematic review included nine studies, but only two were randomised controlled trials, which was insufficient for a meta-analysis to estimate an overall effect [14]. It has been suggested that people from different age groups, particularly the younger versus older age groups, were likely to have different healthcare-seeking behaviours [16]. Healthcare settings could also influence the effectiveness of the intervention being used to improve uptake [17]. Further research is needed to identify effective strategies and optimise intervention components tailored to meet the needs of older adults.

Apart from overall effectiveness, understanding how an intervention works is important for replication in other healthcare settings. In one review of 12 studies, three key components were identified as effective in improving cardiovascular risk factors screening: providing feedback, increasing knowledge, and enhancing health-related dialogue [18]. However, the techniques used to drive behavioural change in these components were not systematically categorised, making it difficult for future replication and generalisation.

The Behaviour Change Techniques Taxonomy (BCTTv1) [19], which includes 93 potential behaviour change techniques, has been developed to provide a standardised way to characterise behaviour change intervention components. Behaviour change technique (BCT) is described as the “observable, replicable, irreducible” intervention component [19]. The BCTTv1 can be used in evidence synthesis. The findings from evidence synthesis can enable researchers to examine how behaviour change techniques were used to bring about behaviour changes by extracting the potentially effective BCT from existing interventions [19] and enhancing the external generalisation of an intervention [20]. Together, these will contribute to the cumulative evidence to advance our understanding of how to enhance general health check uptake.

This systematic review sought to answer two questions: “What is the overall effectiveness of interventions used to improve general health check uptake of older adults?” and “What are the behaviour change techniques used in these effective interventions?”. This information would enrich the evidence base to better inform how an intervention could be designed to improve older adults’ general health check uptake [21]. The objectives were:

1. To review the empirical evidence and identify the interventions that have been used to increase the uptake of general health checks in the older adult population.
2. To determine the overall effectiveness of these interventions using meta-analysis
3. To identify the intervention components and associated intervention functions

## Methods

The reporting of the method section followed the PRISMA 2020 reporting guideline [22,23] (S1 Appendix). This review protocol was registered on PROSPERO (ref: CRD42021221041) and uploaded as a preprint elsewhere [24]. All data relevant to this review’s findings are publicly available (with DIO provided in the reference) and can be found in the supplementary files.

### Eligibility criteria

#### Inclusion criteria.

This review only included randomised controlled trials (RCT) with interventions focusing on improving the general health check service utilisation of older adults. A general health check was defined as “a service that aims to look at the general risk factors of different diseases using several screening tests to assess the general health, but not health services that aim to detect a disease using a specific diagnostic test” [25]. Studies were included as long as the targeted population for the general health check included adults aged 50 years or above. Although older adults are generally defined as those over 65 years old, this lower age criterion was established after considering that preventive services are typically offered to this age and onwards and possible variation in defining the term ‘older adult’ across different studies. The outcome measurement was the actual completion of general health checks.

#### Exclusion criteria.

Based on the operationalised definition mentioned in the inclusion criteria section, disease-specific health checks, e.g. cancer screening, were excluded. Studies which measured intention or willingness to get a general health check would be excluded. Studies were excluded if there was no control or comparison group. No limitation was imposed on the type of intervention comparator. Study protocol, systematic review, conference paper and qualitative empirical studies were also excluded.

### Information sources

Four electronic databases relevant to social science, psychology, and healthcare were searched, including PubMed, PsycINFO, EMBASE and Web of Science. A final decision on the search strategy was reached after modifying the search based on the research team’s feedback and help from a university librarian. A literature search was conducted on 19 August 2020, followed by three search updates on 4 May 2021, 1 June 2023, and 7 May 2024, respectively. The record subtraction method suggested by Bramer and Bain [26] was adopted to identify any additional records that were not included in the previous search.

### Search strategy

Three main concepts were considered in the search strategy development: “general health check”, “intervention” and “uptake”. Together with the alternative search terms, these key terms were combined into a search strategy and executed in four databases, respectively. No language or date limit was imposed on the search strategy. A full search strategy for each database can be found in S2 Appendix. Below is a search strategy executed in PubMed.

• (((“general” AND “health check”) OR “health check?” OR “health check” OR “preventive health check?” OR “medical checkup?” OR “medical check?” OR “comprehensive health check?”) AND (“uptake” OR attend* OR participat* OR utili?ation OR adher* OR appointment?)) AND (“intervention” OR “strategy” OR “strategies” OR “method?” OR “technique?”)

### Study selection

The primary reviewer (WYL) executed the search strategy in the databases and imported the retrieved records into EndNote, where duplicates were eliminated. Then, the dataset was exported in Excel, where the screening of titles and abstracts was performed. Following the coding scheme to record reasons for study exclusion (S3 Appendix), two reviewers worked independently and performed the initial screening of titles and abstracts. Those records that passed the initial eligibility assessment were screened for full text. For any inaccessible records, the primary reviewer (WYL) sought assistance from the university library service to retrieve records from the publisher. Irretrievable records were excluded. The selection results were presented in a PRISMA flowchart.

### Data collection

To address Objective 1, the characteristics of the included studies were extracted by the first reviewer, and the accuracy of the result was checked by the second reviewer. The extracted items included the targeted group, intervention comparators and outcome, details on the study design and the use of theory in the intervention development. The type of intervention was categorised based on the description, e.g. invitation letter, telephone invitation, or questionnaire.

To address Objective 2, the outcome data of the general health check for each intervention was used in meta-analysis to determine the overall effectiveness of the interventions.

To address Objective 3, the components of intervention were characterised using the BCTTv1 [19]. In BCTTv1, a total of 93 BCTs are available for categorisation. These BCTs can be further clustered into 16 groups where each group shares a similar mechanism of change, i.e. how these techniques aim to change behaviour [19]. Two reviewers have completed and passed the online training course on the use of the BCTTv1 [19,21]. They independently identified the BCTs according to the five coding principles [19,27]: 1) coding BCT only if it focused on the behaviour and the target group; 2) identifying if there were different behaviour change types for the BCT (whether the BCT was targeted to the behaviour or the outcome); 3) looking for technical terms that were relevant to BCTTv1; 4) looking for action verbs used in intervention descriptions; 5) following the definition of each BCTs to code the presence of a BCT. For any discrepancy in the identified BCTs, two reviewers justified their initial coding decision and discussed to arrive at a consensus.

To elucidate what intervention functions these BCTs served to change the behaviour, the identified BCTs were mapped to the Behaviour Change Wheel (BCW), where there are nine intervention functions to consider, i.e. education, persuasion, enablement, incentivisation, coercion, restriction, training, environmental restructuring, and modelling [28]. The linkages between intervention functions and BCTs were listed in the design guide [21]. The guide listed the BCTs that were most frequently or less frequently used for specific intervention functions. For this review, the intervention functions of the BCTs were mapped if it was either included in the most frequent or less frequent linkage.

For the BCTs identified in the interventions from the literature updates (search conducted between 2021 and 2024), only WYL was involved in BCT identification and mapping of the corresponding intervention functions.

### Study risk of bias assessment

The Risk of Bias Tool, developed by the Cochrane Review, was used to assess the quality of the included RCTs [29,30]. For studies identified through our initial literature search (conducted in August 2020), two reviewers assessed the study quality independently and provided each domain with a rating of either “low risk”, “high risk”, or “unclear risk” accordingly. All judgements were supported by illustrative quotations. The judgments made by two reviewers were compared, and any discrepancies were resolved through discussion. For the RCTs identified from the literature search updates, only the primary reviewer (WYL) participated in the risks of bias appraisal. The risk of bias illustrations was generated in RevMan5.

Publication bias would be investigated using a funnel plot if there were sufficient studies (more than ten studies) to do so [31].

### Synthesis of results

The study and intervention characteristics were narratively synthesised in a table. The identified BCTs in terms of BCTTv1 were collated and presented in a table for better comparison.

The overall effectiveness of the interventions was obtained by conducting a pairwise meta-analysis. If the RCT contained multiple trial arms, the intervention arms (of any intervention type) would be collapsed into a single intervention group to avoid unit-of-analysis error whereby the intervention groups have been compared more than once [32,33]. A random-effect model (inverse-variance weighting) was considered to estimate the overall effect of the interventions if there were expected factors contributing to heterogeneity (e.g. varied intervention design) as suggested by the Cochrane Systematic Review guideline [34]. This decision was also supplemented by the observation of moderate heterogeneity (i.e. I^2^ value higher than 50%) across studies [34,35]. Otherwise, a fixed-effect model would be used. An effective intervention refers to an intervention group with an odds ratio (OR) greater than one when compared to a control or comparison group. The result for heterogeneity was reported with Q statistics and degree of freedom (*df*), along with the p-value and the 95% confidence intervals. *I*^*2*^ statistics were also provided to illustrate the proportion of the true effect explained by the between-study variance rather than by chance (sampling error) [36]. If it was not possible to further investigate the factors that may have contributed to the extent of heterogeneity, a descriptive discussion would be provided. The pooled summary effect was estimated in Excel and validated using RevMan 5. The forest plot was produced in RevMan5.

### Subgroup analysis

If heterogeneity was evident, and there were sufficient studies (more than ten studies) [36], subgroup analysis would be conducted to explore the potential impact on the results. Otherwise, the potential source of heterogeneity would be discussed narratively and descriptively based on intervention types, demographics, and healthcare settings.

### Sensitivity analysis

If there were evidence with a high risk of bias, sensitivity analysis would be performed to explore the influence of poor-quality RCTs on the findings.

## Results

### Study Selection

**Fig 1:**
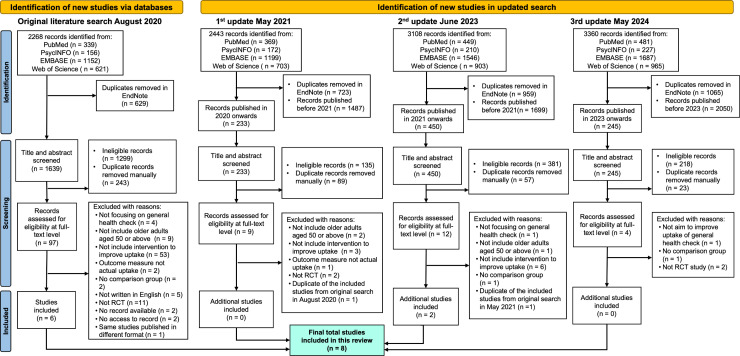
PRISMA flowchart.

The initial literature search yielded 2268 records on 19 August 2020 (Fig 1). After removing duplicates and preliminary screening of titles and abstracts, a total of 97 records were eligible for full-text assessment. Seven RCTs were eligible for inclusion. However, two of them were records of the same study that were published in different formats; one was a detailed report [37], and the other was an empirical research paper [38]. The report paper was included as it would provide more information to enhance our understanding of the intervention design [37]. In total, six relevant RCTs were identified from the initial literature search [37,39–43].

The first updated search yielded 233 new records with the publication year from 2020 onwards to May 2021, among which nine studies were assessed for full text after titles and abstract screening (Fig 1). No new RCT met the inclusion criteria. The second update search yielded 450 new records with a publication year from 2021 onwards to June 2023, among which 12 records were assessed for full-text, and two new RCTs met the inclusion criteria [44,45]. The third update search yielded 245 new records published from 2023 to 7 May 2024, among which four records were screened for full text, but no new RCT met the inclusion criteria. In the end, a total of eight RCTs were finally included in this review [37,39–45].

### Study characteristics

Table 1 shows the characteristics of the eight RCTs. Three RCTs had a two-armed design [39,44,45], four had a three-arm design [37,40,42,43], and one had a mixed-factorial design [41].

**Table 1:** Study characteristics of included studies.

Author/ Year	Country	RCT design	Control group (n)	Intervention group(s) (n)	Theory used ^a^	Outcome measurement	Uptake rate(s), %
Gidlow et al. (2019) [43]	UK	Three-arm	• Standard letter (n = 1454)	• Telephone invitation (n = 1167) • Risk-personal personalised letter (n = 1993)	Not explicitly mentioned, but the intervention was based on framing effect	NHSHC attendance within the 12-month trial	1.Standard letter (control), 30.9% 2.Telephone invitation, 47.6% 3.Risk-personalised letter, 31.3%
Gold et al. (2019) [42]	UK	Double-blinded RCT, Three-arm	• Standard letter (n = 3677)	• Loss-framed leaflet (n = 3664) • Gain-framed leaflet (n = 3697)	Prospect theory	NHSHC attendance by November 2018 (around five months)	1.Standard letter (control), 17.6% 2.Loss-framed leaflet, 17.4% 3.Gain-framed leaflet, 18.2%
Gold et al. (2021) [44]	UK	Pseudo-RCT, Two-arm	• No prompt to clinical staff (n = 3778)	• Point-of-care computerised prompt (n = 3786)	Not mentioned	NHSHC attendance six weeks after the intervention implementation (by 28 August 2015)	1.No prompt (control), 7.4% 2.Point-of-care computerised prompt, 12.0%
McDermott et al. (2016) [37]	UK	Three-arm	• Standard invitation letter (n = 4095)	• QBE questionnaire (n = 3988) • QBE questionnaire + financial incentive (n = 3969)	Theory of planned behaviour	NHSHC attendance within 6 months of receipt of invitation letter	1.Standard invitation (control), 14.4% 2.QBE questionnaire, 15.8% 3.QBE questionnaire + financial incentive, 15.8%
Sallis et al. (2016) [39]	UK	Pragmatic-quasi RCT, Two-arm	• Standard invitation letter (n = 1755)	• Enhanced invitation letter (n = 1756)	Not explicitly mentioned, but focused on a range of behavioural insights	NHSHC attendance during the trial period (specific time frame was not mentioned, subjects were people who were eligible for an NHS health check in 2023 - 2024)	1.Standard invitation (control), 29.3% 2.Enhanced invitation letter, 33.5%
Sallis et al. (2021) [40]	UK	Three-arm	• Standard invitation letter (n = 2123)	• Sunk-cost letter (n = 2105) • Counter-argument letter (n = 2085)	Protection motivation theory; Health belief model; Sunk cost effect; Loss aversion	NHSHC attendance by 12 weeks (after 31 January 2015)	1.Standard invitation letter (control), 34.2% 2.Sunk-cost letter, 38.5% 3.Counter-argument letter, 39.7%
Sallis et al. (2019) [41] ^b^	UK	Double-blinded mixed-factorial	The control arm was without pre-notification SMS, control invitation letter, and without reminder SMS. • NoPre_CO_NoRem (n = 814)	The mixed factorial design resulted in 15 intervention conditions. The combination described followed the pattern: with or without pre-notification sent via SMS + the type of letter + with or without reminder sent via SMS. 1.NoPre_OE_NoRem (n = 724) 2.NoPre_TL_NoRem (n = 747) 3.NoPre_SN_NoRem (n = 800) 4.NoPre_CO_Rem (n = 885) 5.NoPre_OE_Rem (n = 692) 6.NoPre_TL_Rem (n = 754) 7.NoPre_SN_Rem (n = 723) 8.Pre_CO_NoRem (n = 783) 9.Pre_OE_NoRem (n = 765) 10.Pre_TL_NoRem (n = 761) 11.Pre_SN_NoRem (n = 754) 12.Pre_CO_Rem (n = 803) 13.Pre_OE_Rem (n = 727) 14.Pre_TL_Rem (n = 734) 15.Pre_SN_Rem (n = 778)	Not explicitly mentioned, but focused on a range of behavioural insights	NHSHC attendance (results were based on attendance recorded between 5 December 2014 and 2 March 2015)	1.NoPre_CO_NoRem (control), 18.2% Intervention uptakes ranged from 19.9% to 30.0% 2.NoPre_OE_NoRem, 23.1% 3.NoPre_TL_NoRem, 21.2% 4.NoPre_SN_NoRem, 19.9% 5.NoPre_CO_Rem, 23.7% 6.NoPre_OE_Rem, 28.0% 7.NoPre_TL_Rem, 26.9% 8.NoPre_SN_Rem, 24.3% 9.Pre_CO_NoRem, 21.5% 10.Pre_OE_NoRem, 26.7% 11.Pre_TL_NoRem, 25.9% 12.Pre_SN_NoRem, 23.7% 13.Pre_CO_Rem, 23.9% 14.Pre_OE_Rem, 24.6% 15.Pre_TL_Rem, 30.0% 16.Pre_SN_Rem, 24.4%
Shimoda et al. (2022) [45]	Japan	Two-arm	• Template postal reminder (include a website link listing all available clinics) (n = 10543)	• Tailored postal reminder (with the information of the nearest clinics tailored for each participant) (n = 10474)	Not explicitly mentioned. The intervention focused on information seeking for people with lower socioeconomic disadvantages	Attendance of the health check service provided by an insurance association within one month of the receipt of a postal reminder	1.Template postal reminder (control), 2.1% 2.Tailored Postal reminder, 3.2%

RCT = randomised controlled trial; UK = United Kingdom; NHSHC = National Health Service Health Check; QBE = question-behaviour-effect; SMS = short message service.

^a^ The theory used in the intervention was identified from the discussion of the rationale for developing the intervention in the introduction or methods section of the published articles.

^b^ An example of the intervention arm reported in this study [41] using without pre-notification, with Open-ended and with reminder is denoted as NoPre_OE_Rem. The abbreviations used to refer to the intervention components were as follows. Pre = pre-notification; NoPre = no pre-notification; CO = control letter; OE = open-ended letter; TL = time-limited letter; SN = social norms letter; Rem = reminder; NoRem = no reminder

A total of 78,353 participants were included in these eight RCTs, which were conducted in the UK or Japan. The participants from the UK were eligible recipients of the NHS health check programme and were recruited from a range of general practices around the UK. These participants were aged 40 to 74 years, with no previously identified cardiovascular condition [37,39–44]. For the participants included in the RCT conducted in Japan, people who were aged 40 to 69 years, had no health check by December 2016 and were the dependent of an insurer who were eligible for the Specific Health Check-up programme [45].

The service provision of the two general health check programmes varied in payment amount. The NHS Health Check was fully subsidised by the government for eligible individuals [46]. The general health checks in Japan included in this review required a co-payment (either 500 JPY or 1380 JPY, depending on the selected clinics offering the general health check), with the rest covered by the insurance association [45]. No information was provided on the proportion of participants receiving co-payment and at what level [45].

Of the eight RCTs, seven considered the full completion of a general health check as the primary outcome [37,40–45], while one trial considered the completion of an NHS health check and a cholesterol blood test as the primary outcome [39]. The arrangement of the cholesterol blood tests differed among the participating general practices within the trial, which was either arranged before or after the general health check component [39].

The primary outcome was recorded at various time intervals, ranging from one month after the receipt of the invitation letter [45] to within the trial period which last for 15 months [41]. The criteria for classifying the primary outcome as a non-attender differed among the trials. Some studies involved sending reminders to participants before they were classified as non-attenders, but the time could vary between eight weeks [40] and twelve weeks [37] after the first invitation letter was sent out. For the telephone invitation, the general practices staff could make up to three phone calls before considering the case as a non-attender [43].

### Intervention characteristics

Nine intervention types were identified, including postal invitation letters [39–41,43]; telephone invitation [43]; question-behaviour-effect questionnaire [37]; financial incentive [37]; leaflet [42]; pre-notification short message service (SMS) [41], reminder SMS [41], point-of-care automated prompts to clinical staff [44] and postal reminder letter [45].

Among the eight RCTs, three RCTs reported that theory was used to inform the intervention design [37,40,42]. Other interventions incorporated a range of behavioural insights, including simplification, behavioural specificity, personal salience, implementation intention and social comparison [39,41]. The other interventions have focused on addressing the issues identified in the invitation stage that hindered general health check uptake, including not remembering the receipt of the invitation letter [41] and barriers to information seeking [45].

The chosen locations for implementing the intervention were mainly based on the available invitation system or information technology system [37,39,41–44] or the size of the general practices [40]. Two trials reported the selection of the location or the specific group of people focused on areas or groups which have been found to have a low uptake rate [37,45].

The control conditions were different between the UK and Japan. Among the RCTs conducted in the UK, the control arms involved the standard invitation methods by sending out the invitation letter (NHS national template) [37,39–41,43], NHS national template leaflet [42], and having no prompt provided to the clinical staff [44]. For the mixed factorial RCT conducted in the UK, the control arm was sending a control letter (NHS national invitation letter template) in the absence of pre-notification SMS and reminder SMS [41].

The RCT findings on enhanced invitation letters conducted by Sallis et al. [39] were used to revise the content of the NHS national invitation letter template. This enhanced invitation letter was used as a control condition in another included RCT conducted from 2014 to 2015 [43] and was delivered as part of the invitation procedures, which were sent along with the leaflet intervention conducted in 2018 [42].

For the RCT conducted in Japan, the control arm was a standard postal reminder that included website links to a list of available clinics for the participants to choose from [45].

### Identification of behaviour change techniques and intervention functions

Fifteen BCTs were identified across the nine intervention types, accounting for 16.1% (15/93) of the available BCTs from the BCTTv1 (Table 2). No BCT was identified in the pre-notification SMS [38], given the limited description of the instruction provided to the patients to book a general health check appointment. A list of the identified BCTs in each study and illustrative quotations can be found in S4 Appendix.

**Table 2:** Summary of BCTs identified in the included studies.

Study (Year)	Intervention(s)	Behaviour Change Technique(s) a														
		1.4	3.1	4.1	5.1	5.3	5.5	6.2	6.3	7.1	9.1	9.3	10.1	13.2	13.3	15.1
Gidlow et al. (2019) [43]	Telephone invitation		x		x					x						
	Risk-personalised letters				x											
Gold et al. (2019) [42]	Loss-framed leaflet				x	x		x	x			x		x		x
	Gain-framed leaflet				x	x		x	x					x		
Gold et al. (2021) [44]	Point-of-care computerised prompt to clinical staff		x													
McDermott et al. (2016) [37]	QBE questionnaire						x								x	
	QBE questionnaire + financial incentive						x						x		x	
Sallis et al. (2016) [39]	Enhanced invitation letter b	x								x						
Sallis et al. (2021) [40]	Sunk-cost letter			x						x						
	Counterargument letter			x	x						x			x		
Sallis et al. (2019) [41]	Open-ended letter b	x		x						x						
	Time-limited letter b	x		x						x						
	Social-norm letter b			x				x								
	Pre-notification SMS ^c^															
	Reminder SMS			x												
Shimoda et al. (2022) [45]	Tailored postal reminder	x														

SMS = short message service.

^a^In the Behaviour Change Technique Taxonomy (version 1), each behaviour change technique has been assigned a number. This table reports the corresponding number to the behaviour change technique. The full name for each identified BCT is provided as follows. 1.4 = Action planning; 3.1 = Social support (unspecified); 4.1 = Instruction on how to perform the behaviour; 5.1 = Information about health consequences; 5.3 = Information about social and environmental consequences; 5.5 = Anticipated regret; 6.2 = Social comparison; 6.3 = Information about others’ approval; 7.1 = Prompts/ cues; 9.1 = Credible sources; 9.3 = Comparative imagining of future outcomes; 10.1 = Material reward (behaviour); 13.2 = Framing/ reframing;13.3 = Incompatible belief; 15.1= Verbal persuasion about capability

^b^The BCTs used in the intervention have already been characterised by the authors themselves.

^c^There was insufficient information to code the intervention content for this intervention.

These 15 BCTs have been used to increasing knowledge, providing social support, and changing reflective motivation to use general health checks. These 15 BCTs were grouped under ten different BCT groups including “goals and planning” (action planning), “social support” (social support (unspecified)), “shaping knowledge” (instruction on how to perform the behaviour), “natural consequences” (information about health consequences; information about social and environmental consequences; anticipated regret), “comparison of behaviour” (social comparison; information about others’ approval), “associations’ (prompts/ cues), “comparison of outcomes” (credible sources; comparative imagining of future outcomes); “reward and threat” (material reward (behaviour)), “identify” (framing/ reframing; incompatible belief), and “self-belief” (verbal persuasion about capability).

These 15 BCTs can be mapped to seven different intervention functions in the BCW, namely, education, persuasion, coercion, incentivisation, training, environmental restructuring, and enablement (S5 Appendix).

The most common BCT were “Instructions on how to perform the behaviour” (n = 6), followed by “Prompts/ cues” (n =5) and “Information about health consequence” (n = 5). “Action planning” techniques were also a common technique incorporated into the design of a letter or reminder letter (n = 4).

### Risk of bias

**Fig 2:**
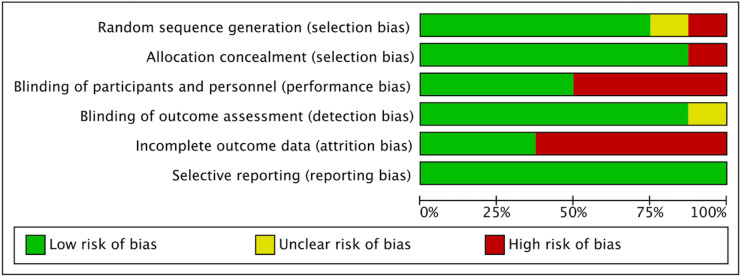
Risk of bias graph.

**Fig 3:**
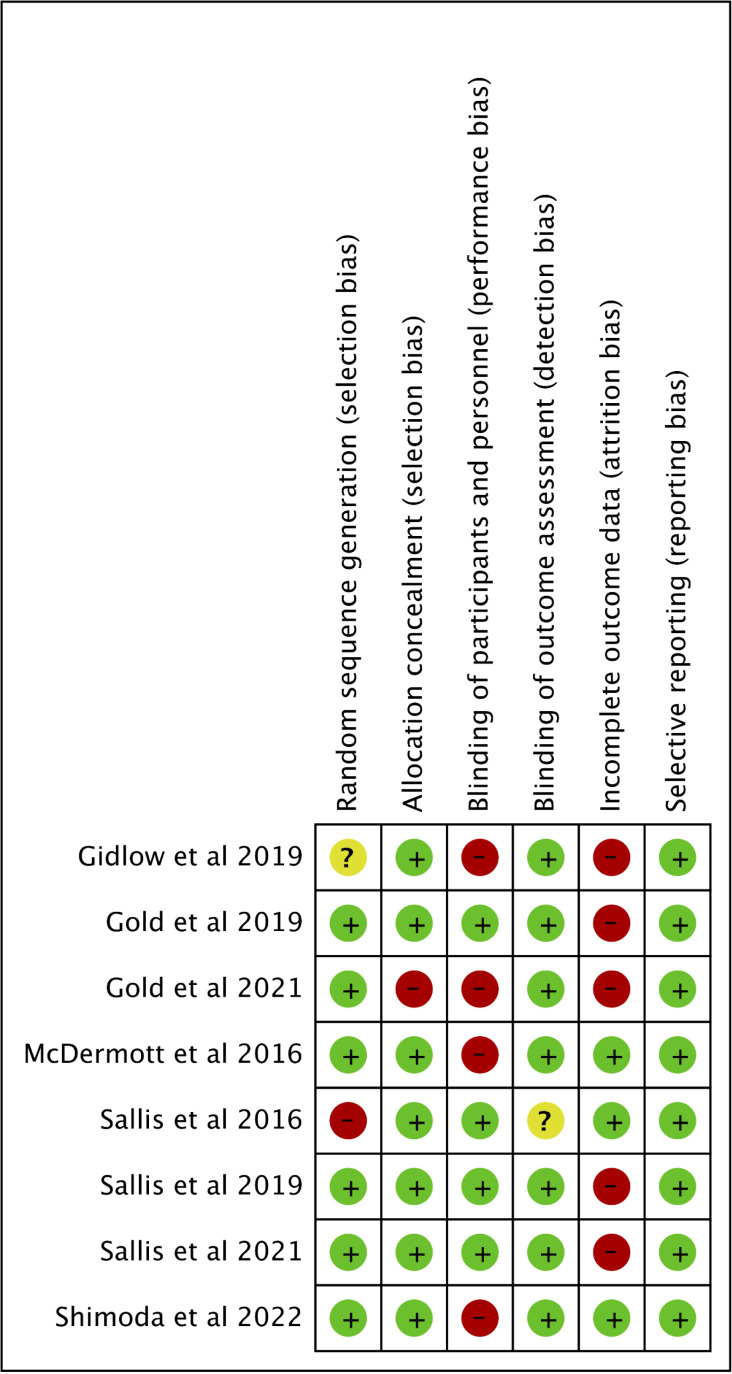
Risk of bias summary.

The risk of bias graph and risk of bias summary are shown in Fig 2 and Fig 3, respectively.

Overall, all studies demonstrated good randomisation, except for two RCTs [39,43]. For allocation concealment, only one RCT could not conceal the allocation from the researchers due to IT restrictions [44]. The blinding of participants and personnel was not possible for four RCTs due to the intervention characteristics being impossible to blind [37,43–45]. For the blinding of the outcome assessor, only one RCT did not describe clearly how the outcome measurement was handled [36].

Incomplete outcome data was identified as a potential risk of bias for five RCTs, and the reasons included errors found during randomisation [44] and during intervention delivery [41, 42]. Three RCTs reported that data were lost [40,43,44]. Reasons included failure to follow protocol fully [43] and missing ethnicity data for non-attenders due to logistics for data collection [40]. In Gold et al. [44], the authors reported that two out of 15 participating practices reported zero general health check attendance, yet suspected reasons were not explored.

### Synthesis of results

The intervention arms reported by individual studies were significantly more effective than the control arm in improving general health check uptake, except for leaflets and QBE questionnaires with or without financial incentives [37,42]. In the mixed factorial RCTs, four intervention arms were not significantly more effective than the control arm (with no pre-notification SMS, control letter and no SMS reminder) [41]. The characteristics of these ineffective intervention arms included the intervention arms without both pre-notification SMS and SMS reminder (a total of three intervention arms) and the intervention arm with control letter and pre-notification SMS but no SMS reminder [41].

### Meta-analysis

**Fig 4:**
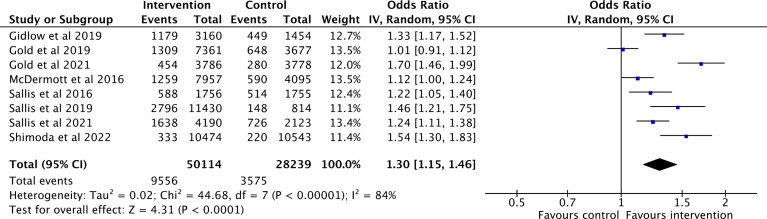
Meta-analysis results.

All uptake outcome data were available for all trials. Random-effect meta-analysis using inverse-variance weighting was used to estimate the summary effect, assuming there was a different true effect amongst studies given the different identified intervention types. A total of 78,353 participants were included in the meta-analysis. The overall pooled effect size suggested the interventions were effective in improving general health check uptakes (OR = 1.30, 95% CI =1.15 - 1.46) (Fig 4). There was evidence of heterogeneity, with 84% of the variation was caused by variation across studies and not by random sampling error (*df* = 7, *p* < 0.00, *I* ^2^ = 84%). However, there were not enough studies to explore the heterogeneity. In the absence of further investigation, the direction of the intervention effect of individual studies was reviewed [36]. Six out of eight studies were statistically effective in improving general health check uptake, which aligned with the direction of the summary effect estimate.

### Subgroup analysis

Given the constraint that only eight studies were included in this review, subgroup analysis was not feasible to be conducted.

### Sensitivity analysis

No specific RCTs reported a high risk of bias in all domains. Sensitivity analysis was not performed to explore the potential impact of risk of bias on the results.

## Discussion

This systematic review synthesised the most reliable and relevant evidence available to establish the overall effectiveness of interventions and identified the BCTs applied in the design to enhance participation rates for general health checks by older adults. Eight relevant RCTs were included in this review. Notably, all interventions were designed for the invitation stage. The types of interventions varied by the mode of invitation (i.e. via letter, telephone or questionnaire), financial incentives, leaflets, mode of reminder (i.e. pre-notification, or reminders), and opportunistic invitations using computer prompts to clinical staff. Fifteen BCTs were identified in these interventions, providing supporting evidence on the use of these BCTs in intervention to improve general health check uptake [47].

In general, these interventions significantly improved the general health check uptakes compared to the control group [39–41,43–45]. However, sending invitation letters with leaflets (loss-framed or gain-framed) [42] or QBE questionnaires (with or without financial incentives) [37] were found to be ineffective. One of the possible reasons was that the theories applied in both interventions (leaflet and QBE questionnaire) emphasised changing the knowledge, attitude or intentions about general health checks [37,42], without addressing the gap to cue from intention to actual behaviour change, i.e. an intention-behaviour gap still existed [37]. Second, considering the target group with a large involvement of older adults, the provision of supplementary materials or required completion of questionnaires could be less motivated and acceptable by this age group due to the required literacy level and the inconvenience caused.

Apart from altering invitation letter contents was found to be effective in improving general health check uptake, other effective interventions involved interaction with clinical staff, in terms of offering an appointment booking through telephone invitation [43] or providing automated computerised prompts to clinical staff and prompting them book an appointment for the patients [44]. The influence of clinical staff was also evident in the use of credible sources while disseminating health-related information in letters using the phrase “*Your GP says”* [40]. The use of social comparison, which involves letting individuals know that other people performed a similar behaviour so they can make comparisons themselves [42], was found to be effective in invitation letters but not in leaflets. Therefore, it was observed that effective intervention tended to include a combination of BCTs that provided knowledge to perform the behaviour, action planning and prompts/ cues to facilitate behaviour change. Seeking input from the stakeholders can further evaluate the intervention design from a recipient’s perspective, ensuring the understanding of the intervention would align with the designer’s intention [48].

The identified BCTs have the potential for generalisation across different contexts. It should be noted that some intervention contents might not be fully captured by the BCTTv1. In this study, no BCTs were identified for pre-notification SMS [41]. While future researchers should consider using BCTTv1 to describe the techniques used in intervention components, detailed intervention descriptions should be provided to facilitate study replications and modifications.

## Limitations

Limitations of the review were identified. First, the number of RCTs yielded may be limited by only including publications in English. Second, most RCTs were conducted in the UK, where the NHS is a publicly funded healthcare system where the financial barrier was minimised. This would limit the types of intervention and BCTs being considered in the intervention design which may not be able to address various barriers in the pathway of deterring the uptake of general health checks under different health care systems. Beyond the UK health care system, financial [49] and non-financial barriers such as cultural and accessibility issues [50,51] were reported by studies across developed and developing regions.

Thirdly, the included studies showed high heterogeneity in meta-analysis. Given the insufficient number of studies to perform subgroup analysis, the potential sources of heterogeneity were narratively discussed by observing trends from reviewed studies. One of the potential sources of heterogeneity was the variation in the intervention design as discussed above. For example, interventions involving interpersonal interactions seem to have higher effects on improving general health check uptake than those without such components. The relative improvement in general health check uptake for telephone invitations [43] and point-of-care prompts to clinical staff [44] compared to their own control groups were generally higher than those involved in sending printed materials. This trend aligned with those reported in the literature [52]. The other potential sources could be the participants’ demographics. Varied age groups were included in the reviewed studies. Recruited participants from five studies were generally older [39,40,42,43,45], while the other three studies were generally younger [37,41,44], i.e. reported a mean/ median age below the age of 50 or had a higher proportion of participants below age 50. Two studies reported that the intervention was more effective in improving the general health check uptake among the younger age group than the older group [43,44]. Lastly, this review included RCTs mainly from the UK, with only one trial conducted in Japan [45]. Therefore, the influence of healthcare setting differences on the overall pooled effect size would be trivial. Considering these between-study variations and that in the absence of subgroup analysis, the reliability of the overall pooled effect size could be reduced.

## Implication

This review highlights the knowledge gap on strategies designed to improve general health check uptakes. The invitation stage is only part of the general health check pathway [46]. There are opportunities to consider implementing interventions at different time points along the general health check pathways [47].

Identifying BCTs in intervention components will allow us to understand theoretically what BCTs are most likely to result in effective behaviour change. Informed by the theory-driven design, e.g. using BCTs, is one of the sources of the intervention’s success, but it is not the only one. Beyond a theory-driven intervention design, other sources of factors during the implementation and surrounding feasibility need to be considered, as suggested by the current literature [53,54].

The findings from this review can be generalised to settings where there is an organised and subsidised general health check service in place, particularly with the potential to implement such intervention in the invitation stage. The published RCTs were conducted in developed regions. It is unclear if these findings could be generalised to other developing regions where subsidised service is unavailable. Previous research has reported that healthcare settings and healthcare reimbursement models play a role in influencing preventive service seeking behaviour [54]. The effectiveness of intervention types and BCTs identified in the current review should be further investigated across other healthcare contexts. More importantly, studying the context-specific barriers in other healthcare settings can determine if the theory- and evidence-based BCTs would be useful to be incorporated in interventions to improve general health check uptakes.

The findings of this review only provided evidence regarding the overall effectiveness of the identified BCTs associated with effective intervention when being implemented as a set of BCTs. It is unclear if implementing a single BCT could lead to significant improvement in general health check uptakes. When more empirical evidence becomes available, it might be possible to study the effect of a single or a set of BCTs and their impact on general health check uptakes, such as by conducting meta-regression [55]. This will allow researchers to understand how BCTs could be combined or implemented alone to improve general health check uptakes, allowing more flexibility in their application under specific healthcare contexts.

## Conclusion

This review identified the interventions in the current literature were all implemented during the invitation stage and were overall effective in improving general health check uptake. This review also identified the BCTs associated with these interventions. More research is needed to investigate context-specific barriers to general health check uptake under different healthcare settings to allow holistic interventions to be developed to address the barriers along the general health check seeking pathway, especially those beyond the invitation stage.

## Supporting information

S1 Appendix PRISMA 2020 reporting completed checklist. (DOCX)

S2 Appendix Full search strategy. (DOCX)

S3 Appendix Screening selection criteria coding scheme and exclusion reasons for excluded records. (PDF)

S4 Appendix Identified BCTs in each intervention with illustrative quotes. (DOCX)

S5 Appendix Linking BCTs to the identified intervention functions in the BCW. (DOCX)
