# Supplementary material for: The effectiveness of interventions used to improve general health check uptake by the older adult population: a systematic review and meta-analysis
Source: PLOS Glob Public Health. 2025 Mar 31;5(3):e0004362. doi: 10.1371/journal.pgph.0004362 (PMC11957279; doi:10.1371/journal.pgph.0004362)
Supplement: S2 Appendix — (DOCX) [file pgph.0004362.s002.docx]

S2 Appendix. Full search strategy.

| **Database** | **Search strategy** |
| --- | --- |
| PubMed | ((("general" AND "health check") OR "health check?" OR "health check" OR "preventive health check?" OR "medical checkup?" OR "medical check?" OR "comprehensive health check?") AND ("uptake" OR attend* OR participat* OR utili?ation OR adher* OR appointment?)) AND ("intervention" OR "strategy" OR "strategies" OR "method?" OR "technique?") |
| PsycINFO | (((“general” AND “health check”) OR "health check?" OR "health check" OR "preventive health check?" OR "medical checkup?" OR "medical check?" OR "comprehensive health check?") AND ("uptake" OR attend* OR participat* OR utili?ation OR adher* OR appointment?)) AND (("intervention" OR "strategy" OR "strategies" OR "method?" OR "technique?")) |
| EMBASE | (('general' AND 'health check') OR 'health check?' OR 'health check' OR 'preventive health check?' OR 'medical checkup?' OR 'medical check?' OR 'comprehensive health check?') AND ('uptake' OR attend* OR participat* OR utili?ation OR adher* OR appointment?) AND ('intervention' OR 'strategy' OR 'strategies' OR 'method?' OR 'technique?') |
| Web of Science | (((“general” AND “health check”) OR "health check?" OR "health check" OR "preventive health check?" OR "medical checkup?" OR "medical check?" OR "comprehensive health check?") AND ("uptake" OR attend* OR participat* OR utili?ation OR adher* OR appointment?)) AND (("intervention" OR "strategy" OR "strategies" OR "method?" OR "technique?")) |
