# Supplementary material for: The effectiveness of interventions used to improve general health check uptake by the older adult population: a systematic review and meta-analysis
Source: PLOS Glob Public Health. 2025 Mar 31;5(3):e0004362. doi: 10.1371/journal.pgph.0004362 (PMC11957279; doi:10.1371/journal.pgph.0004362)
Supplement: S3 Appendix — (PDF) [file pgph.0004362.s003.pdf]

# S3 Appendix

## Coding for exclusion criteria and the summary of the excluded studies and their excluded reasons

Excluded studies and their reason for exclusion is stated. Below is the coding scheme.

| Code | Exclusion criteria |
|---|---|
| A | The research was not about general health check/ or disease-specific screening |
| B | Health check service did not include older people population **aged 50** or above |
| C | The **intervention** was not designed to improve the uptake or utilisation of general health check |
| D | The outcome measure was not the **actual uptake or utilisation** (i.e. intention/ willingness to use) |
| E | Study did ***not*** include a **control/comparison** group |
| F | Not written in English |
| G | Not published as an empirical research article, randomised controlled trial |
| H | Have no record available |
| I | Have no access to the paper |

**Literature search: August 2020**
**Titles and abstracts screening with excluded reasons**

| # | Author | Year | Title | Excluded with reason |
|---|---|---|---|---|
| 1 | Family Heart Study Group | 1994 | British family heart study: its design and method, and prevalence of cardiovascular risk factors | A |
| 2 | Aalto, M.; , Alho, H.; , Halme, J. T.; and Seppa, K. | 2009 | AUDIT and its abbreviated versions in detecting heavy and binge drinking in a general population survey | A |
| 3 | Aarup, M.; , Sokolowski, I.; and Lous, J. | 2008 | [The prevalence of obesity and overweight among 3 year-old children in the municipality of Aalborg and identification of risk factors] | A |
| 4 | Ab Rahman, N.; , Sivasampu, S.; , Noh, K. M.; and Khoo, E. M. | 2016 | Health profiles of foreigners attending primary care clinics in Malaysia | A |
| 5 | Abe, T.; Aoki, T.; Yata, S.; Okada, M. | 2011 | Sleep duration is significantly associated with carotid article atherosclerosis incidence in a Japanese population | A |
| 6 | Abera, B.; Yitayew, G.; Amare, H. | 2016 | Salmonella serotypetyphi, shigella, and intestinal parasites among food handlers at bahir dar university, Ethiopia | A |
| 7 | Adesokan, H. K.; , Akinseye, V. O.; and Sulaimon, M. A. | 2018 | Knowledge and practices about zoonotic tuberculosis prevention and associated determinants amongst livestock workers in Nigeria; 2015 | A |
| 8 | Agarwal, N.; Sebastian, M. P.; Agarwal, S. | 2016 | Assessing the adoption of a home health provisioning system in India: An analysis of patients | A |
| 9 | Aguado Taberné, C.; , Martínez de la Iglesia, J.; , Espejo Espejo, J.; , Yun Casalilla, A.; , Muñoz Alamo, M.; and Ruiz Moral, R. | 1996 | [Clinical situation, knowledge and risky behavior of patients with human immunodeficiency virus infection attending a health center] | A |
| 10 | Ahmed, F.; Waslien, C.; Al-Sumaie, M. A.; Prakash, P.; Allafi, A. | 2013 | Trends and risk factors of hyperglycemia and diabetes among Kuwaiti adults: National Nutrition Surveillance Data from 2002 to 2009 | A |
| 11 | Aida, Y.; Shibata, Y.; Osaka, D.; Abe, S.; Inoue, S.; Fukuzaki, K.; Tokairin, Y.; Igarashi, A.; Yamauchi, K.; Nemoto, T.; Nunomiya, K.; Kishi, H.; Sato, M.; Watanabe, T.; Konta, T.; Kawata, S.; Kato, T.; Kubota, I. | 2011 | The relationship between serum uric acid and spirometric values in participants in a health check: The takahata study | A |
| 12 | Akasaka, H.; , Katsuya, T.; , Saitoh, S.; , Sugimoto, K.; , Fu, Y.; , Takagi, S.; , Ohnishi, H.; , Rakugi, H.; , Ura, N.; , Shimamoto, K.; and Ogihara, T. | 2006 | Effects of angiotensin II type 1 receptor gene polymorphisms on insulin resistance in a Japanese general population: the Tanno-Sobetsu study | A |

| | | | | |
|---|---|---|---|---|
| 13 | Akter, S.; Nanri, A.; Kuwahara, K.; Matsushita, Y.; Nakagawa, T.; Konishi, M.; Honda, T.; Yamamoto, S.; Hayashi, T.; Noda, M.; Mizoue, T. | 2017 | Circulating ferritin concentrations and risk of type 2 diabetes in Japanese individuals | A |
| 14 | Alaei-Shahmiri, F.; Khamseh, M. E.; Manhoei, K.; Yadegari, H.; Kazemi, H.; Meshkini, M. | 2020 | The optimal vitamin D cut-off value associated with hyperglycemia in an Iranian population | A |
| 15 | Alageel, S.; , Gulliford, M. C.; , McDermott, L.; and Wright, A. J. | 2018 | Implementing multiple health behaviour change interventions for cardiovascular risk reduction in primary care: a qualitative study | A |
| 16 | Alageel, Samah; , Gulliford, Martin C.; , Wright, Alison; , Khoshaba, Bernadette; and Burgess, Caroline | 2019 | Engagement with advice to reduce cardiovascular risk following a health check programme: A qualitative study | A |
| 17 | AlHamdan, N. A.; , Abbas, M. A. F.; , Aiqahtani, M. S.; , Klantan, S.; , Fiala, L. A.; and ElGhazali, G. | 2013 | Comparison of Tuberculin Skin Test (TST) and Quantiferon Test (QFT) for detection of Latent TB infection among Health Care Workers (HCWs) in a Tertiary Care Hospital in Riyadh, Saudi Arabia | A |
| 18 | Alkon, A.; Bernzweig, J.; To, K.; Wolff, M.; Mackie, J. F. | 2009 | Child Care Health Consultation Improves Health and Safety Policies and Practices | A |
| 19 | Alonso, I.; , Valdivielso, P.; , Josefa Zamudio, M.; , Sánchez Chaparro, M. A.; , Pérez, F.; , Ramos, H.; and González Santos, P. | 2009 | [Usefulness of the ankle-arm index for detection of peripheral arterial disease in a working population of Junta de Andalucía at Málaga] | A |
| 20 | Alshammari, S. A.; , AlShowair, M. A.; and AlRuhaim, A. | 2017 | Use of hormones and nutritional supplements among gyms' attendees in Riyadh | A |
| 21 | Amorin-Woods, L. G.; Parkin-Smith, G. F.; Nedkoff, L.; Fisher, C. | 2016 | Outcomes of a pilot study in chiropractic practices in Western Australia | A |
| 22 | Ampt, A. J.; Amoroso, C.; Harris, M. F.; McKenzie, S. H.; Rose, V. K.; Taggart, J. R. | 2009 | Attitudes, norms and controls influencing lifestyle risk factor management in general practice | A |
| 23 | An, T. D.; , McDermott, R.; , Knight, J.; , Hua, X. Y.; , Barr, E. L. M.; , Arabena, K.; , Palmer, A.; and Clarke, P. M. | 2020 | Development and Use of Prediction Models for Classification of Cardiovascular Risk of Remote Indigenous Australians | A |
| 24 | Andersson, S.; , Karlsson, V.; , Bennet, L.; , Fellbrant, K.; and Hellgren, M. | 2016 | Attitudes Regarding Participation in a Diabetes Screening Test among an Assyrian Immigrant Population in Sweden | A |
| 25 | Aouira, N.; Khan, S.; Heussler, H.; McDermott, B.; Haywood, A.; Bor, W. | 2018 | Metabolic monitoring of youth prescribed antipsychotics: The evidence gap widens in failing to provide reasonable standard of physical healthcare | A |
| 26 | Arabyat, Rasha M.; , Raisch, Dennis W.; and Bakhireva, Ludmila | 2018 | Influenza vaccination for patients with chronic obstructive pulmonary disease: Implications for pharmacists | A |
| 27 | Arthur, A. J.; Jagger, C.; Lindesay, J.; Matthews, R. J. | 2002 | Evaluating a mental health assessment for older people with depressive symptoms in general practice: A randomised controlled trial | A |
| 28 | Arthur, A. J.; Matthews, R. J.; Jagger, C.; Clarke, M.; Hipkin, A.; Bennison, D. P. | 2002 | Improving uptake of influenza vaccination among older people: A randomised controlled trial | A |

| | | | | |
|---|---|---|---|---|
| 29 | Asare, M.; Mustian, K. M.; Kleckner, I.; Peppone, L. J.; Magnuson, A.; Tejani, M. A.; Heckler, C. E.; Kamen, C. S. | 2016 | Ethnic/racial and socioeconomic disparities as predictors of behavioral risk factors among 7559 cancer survivors | A |
| 30 | Asfaw, A.; Colopy, M. | 2017 | Association between parental access to paid sick leave and children's access to and use of healthcare services | A |
| 31 | Ashavaid, T. F.; Todur, S. P.; Dherai, A. J. | 2004 | Health status of Indian population--current scenario | A |
| 32 | Ashavaid, T.; Sawant, A.; Mankeshwar, R. | 2011 | Correlation of genetic variations of genes involved in low high density lipoproteins cholesterol synthesis in Indians | A |
| 33 | Aslesh, O. P.; Paul, S.; Paul, L.; Jayasree, A. K. | 2015 | High prevalence of tobacco use and associated oral mucosal lesion among interstate male migrant workers in urban Kerala, India | A |
| 34 | Aswini Dutt, R.; Satish Kumar, N. S.; Ramaswamy, C.; Ramesh Bhat, M.; Niranjan Murthy, H. L. | 2011 | A comparative study of blood pressure, heart rate variability and metabolic risk factors in software professionals | A |
| 35 | Atsuta, Y.; Kawase, H.; Hamajima, N.; Nishio, K.; Niwa, Y.; Tanaka, D.; Yamamoto, K.; Tamakoshi, A. | 2005 | Use of duplex PCR-CTPP methods for CYP2E1 RsaI/IL-2 T-330G and IL-1B C-31T/TNF-A T-1031C polymorphisms | A |
| 36 | Avdal, E. U.; , Kizilci, S.; and Demirel, N. | 2011 | The effects of web-based diabetes education on diabetes care results: a randomized control study | A |
| 37 | Avila, Jaqueline C.; , Kuo, Yong-Fang; , Rodriguez, Ana M.; , Wong, Rebeca; and Kaul, Sapna | 2017 | Preventive services use among female survivors of adolescent and young adult cancer | A |
| 38 | Awasthi, M. S.; Awasthi, K. R.; Thapa, H. S.; Saud, B.; Pradhan, S.; Khatry, R. A. | 2018 | Utilization of Antenatal Care Services in Dalit Communities in Gorkha, Nepal: A Cross-Sectional Study | A |
| 39 | Bae, J. C.; Han, J. M.; Kwon, S.; Jee, J. H.; Yu, T. Y.; Lee, M. K.; Kim, J. H. | 2016 | LDL-C/apoB and HDL-C/apoA-1 ratios predict incident chronic kidney disease in a large apparently healthy cohort | A |
| 40 | Bae, J. C.; Jin, S. M.; Kim, J. H.; Hur, K. Y.; Lee, M. S.; Lee, M. K. | 2014 | Non HDL-cholesterol/HDL-cholesterol is a better predictor of future development of metabolic syndrome than apolipoprotein B/apolipo protein A1 | A |
| 41 | Bae, J. C.; Rhee, E. J.; Lee, W. Y.; Park, S. E.; Park, C. Y.; Oh, K. W.; Park, S. W.; Kim, S. W. | 2011 | Optimal range of HbA1c for the prediction of future diabetes: A 4-year longitudinal study | A |
| 42 | Bae, J. C.; Suh, S.; Jin, S. M.; Kim, S. W.; Hur, K. Y.; Kim, J. H.; Min, Y. K.; Lee, M. S.; Lee, M. K.; Jeon, W. S.; Lee, W. Y.; Kim, K. W. | 2014 | Hemoglobin A1c values are affected by hemoglobin level and gender in non-anemic Koreans | A |
| 43 | Bagliesi, G.; , Boccali, T.; , Della Ricca, G.; , Donvito, G.; , Paganoni, M.; and Iop | 2012 | Building a Prototype of LHC Analysis Oriented Computing Centers6. Conclusion The ECAL safety systems are based on very solid hardware configurations with extremely long life cycles, avoiding the need for short- to mid-term upgrades. In respect to the PLC codes, there is still room for non-critical improvements and new features, which could be considered during the LHC long shutdown periods. The planning for the coming years should focus on preventive maintenance, periodic health checks and systematic routine tests. The control system provides excellent support and additional protection | A |

| | | | | |
|---|---|---|---|---|
| | | | for the detector operation. While reaching a consolidation phase, it faces several challenges to be kept in-line not only with the new CMS DCS standards, but also to follow the natural evolution of hardware interfaces and software platforms. In addition, new solutions are available and under implementation to overcome existing hardware limitations and to improve the provided services | |
| 44 | Bailie, R. S.; , Si, D. M.; , Connors, C. M.; , Kwedza, R.; , O'Donoghue, L.; , Kennedy, C.; , Cox, R.; , Liddle, H.; , Hains, J.; , Dowden, M. C.; , Burke, H. P.; , Brown, A.; , Weeramanthri, T.; and Thompson, S. | 2011 | Variation in quality of preventive care for well adults in Indigenous community health centres in Australia | A |
| 45 | Balata, H.; Crosbie, P.; Evison, M.; Yarnell, L.; Threlfall, A.; Barber, P.; Tonge, J.; Booton, R. | 2017 | Manchester lung screening, targeting highrisk individuals in deprived areas of the community | A |
| 46 | Balata, H.; Harvey, J.; Barber, P. V.; Colligan, D.; Duerden, R.; Elton, P.; Evison, M.; Greaves, M.; Howells, J.; Irion, K.; Karunaratne, D.; Mellor, S.; Newton, T.; Sawyer, R.; Sharman, A.; Smith, E.; Taylor, B.; Taylor, S.; Tonge, J.; Walsham, A.; Whittaker, J.; Vestbo, J.; Booton, R.; Crosbie, P. A. | 2020 | Spirometry performed as part of the Manchester community-based lung cancer screening programme detects a high prevalence of airflow obstruction in individuals without a prior diagnosis of COPD | A |
| 47 | Balata, H.; Traverse-Healy, L.; Blandin-Knight, S.; Armitage, C.; Barber, P.; Colligan, D.; Elton, P.; Kirwan, M.; Lyons, J.; McWilliams, L.; Novasio, J.; Sharman, A.; Slevin, K.; Taylor, S.; Tonge, J.; Waplington, S.; Yorke, J.; Evison, M.; Booton, R.; Crosbie, P. A. J. | 2020 | Attending community-based lung cancer screening influences smoking behaviour in deprived populations | A |
| 48 | Baldwin, D. | 2018 | How Is Lung Cancer Screening Evolving to Be More Efficient and Effective? | A |
| 49 | Bandurska-Stankiewicz, E.; Kołakowska, H.; Zbikowska, E. | 2002 | Analysis of effectiveness of continuous educational rehabilitation program of diabetic patients with visual disability | A |
| 50 | Banerjee, S. R. | 1991 | Child labor in suburban areas of Calcutta, West Bengal | A |
| 51 | Banim, P. | 2013 | Is pancreatic cancer a metabolic disease? Data from a UK prospective cohort study | A |
| 52 | Banim, P. J. R.; Luben, R.; Khaw, K. T.; Wareham, N.; Hart, A. R. | 2011 | Physical activity and the risk of developing pancreatic cancer-data from a UK prospective study (epic-norfolk) | A |
| 53 | Banim, P. R.; Luben, R.; Khaw, K. T.; Hart, A. | 2012 | Is pancreatic cancer a metabolic disease? Data from a UK prospective cohort study | A |
| 54 | Banka, R.; Lonkar, S.; Walkar, M.; Pabidha, G.; Shorofsky, M.; Bourbeau, J.; Pinto, L. | 2015 | There use of the vitalograph COPD-6 device as a pointof-care screening tool for COPD in resource-limited settings | A |
| 55 | Bansal, P.; , Chaudhary, A.; , Wander, P.; , Satija, M.; , Sharma, S.; , Girdhar, S.; , Kaushal, P.; and Gupta, V. K. | 2016 | Cardiovascular Risk Assessment Using WHO/ISH Risk Prediction Charts In a Rural Area of North India | A |

| | | | | |
|---|---|---|---|---|
| 56 | Barnes, S.; Greenhouse, P. | 2013 | Would you go to your own hospital's sexual health clinic? | A |
| 57 | Barta, A. G. B.; Turgonyi, Z. T.; Kiss, E. K.; Simon, E. S.; Szilfai, N. S.; Sumanszki, C. S.; Reismann, P. R. | 2019 | The impact of therapy adherence on the quality of life in adult Phenylketonuria patients | A |
| 58 | Bartlett, E. C.; Kemp, S.; Derbyshire, J.; Morris, K.; Addis, J.; Ridge, C.; Mirsadraee, S.; Padley, S.; Desai, S. R.; Devaraj, A. | 2019 | Implications and outcomes of clinical and radiological incidental lung cancer screening findings for primary care - Results from a pilot screening study | A |
| 59 | Bartlett, E. C.; Kemp, S. V.; Ridge, C. A.; Desai, S. R.; Mirsadraee, S.; Morjaria, J. B.; Shah, P. L.; Popat, S.; Nicholson, A. G.; Rice, A. J.; Jordan, S.; Begum, S.; Mani, A.; Derbyshire, J.; Morris, K.; Chen, M.; Peacock, C.; Addis, J.; Martins, M.; Kaye, S. B.; Padley, S. P. G.; Devaraj, A.; McDonald, F.; Robertus, J. L.; Lim, E.; Barnett, J.; Finch, J.; Dalal, P.; Yousaf, N.; Jamali, A.; Ivashniova, N.; Phillips, C.; Newsom-Davies, T.; Lee, R.; Vaghani, P.; Whiteside, S.; Vaughan-Smith, S. | 2020 | Baseline Results of the West London lung cancer screening pilot study – Impact of mobile scanners and dual risk model utilisation | A |
| 60 | Bartlett, E.; Kemp, S.; Desai, S.; Mirsadraee, S.; Ridge, C.; Morjaria, J.; Shah, P.; Morris, K.; Derbyshire, J.; Chen, M.; Peacock, C.; Ivashniova, N.; Martins, M.; Addis, J.; Padley, S.; Devaraj, A. | 2019 | MA10.10 Uptake in Lung Cancer Screening – Does CT Location Matter? A Pilot Study Comparison of a Mobile and Hospital Based CT Scanner | A |
| 61 | Bassani, D. G.; Arora, P.; Wazny, K.; Gaffey, M. F.; Lenters, L.; Bhutta, Z. A. | 2013 | Financial incentives and coverage of child health interventions: a systematic review and meta-analysis | A |
| 62 | Bassi, S.; Bahl, D.; Hundal, N.; Wipfli, H.; Arora, M. | 2018 | Strengthening tobacco-free worksite policies in India | A |
| 63 | Baum, E.; Donner-Banzhoff, N.; Jakle, C.; Keller, S.; Miko, M.; Sarafowa, A.; Basler, H. D. | 1999 | Health education and motivation to change. A study of high-risk patients detected by health check-up | A |
| 64 | Baum, M.; Pfeiffer, W.; Ciré, L.; Kentner, M. | 2002 | Alcohol consumption in executives compared to in the normal population | A |
| 65 | Beardslee, W. R.; Klosinski, L. E.; Saltzman, W.; Mogil, C.; Pangelinan, S.; McKnight, C. P.; Lester, P. | 2013 | Dissemination of Family-Centered Prevention for Military and Veteran Families: Adaptations and Adoption within Community and Military Systems of Care | A |
| 66 | Bellini, M. A. S. | 2019 | Group hypnosis in the treatment of psychological and somatic symptoms in adjustment disorder with study abroad university students | A |
| 67 | Bener, A.; El Ayoubi, H. R.; Moore, M. A.; Basha, B.; Joseph, S.; Chouchane, L. | 2009 | Do we need to maximise the breast cancer screening awareness?: Experience with an endogamous society with high fertility | A |
| 68 | Berks, D.; Hoedjes, M.; Raat, H.; Duvekot, H. J.; Steegers, E. A. P. | 2015 | Effects of lifestyle intervention after complicated pregnancy: Results of the Pro-Active study | A |

| | | | | |
|---|---|---|---|---|
| 69 | Berry, E.; Silvera, A.; Whitney Downer, W.; Agar, D.; Beske Radford, S.; Chiromeras, N.; Bogart, M.; Bonnet, N. | 2017 | Patient engagement improvement following implementation of a remote monitoring system | A |
| 70 | Bhasin, V.; Mehta, A.; Skopicki, H. A.; Parikh, P. B. | 2020 | Predictors of Aspirin Nonadherence in Adults With Prior Myocardial Infarction | A |
| 71 | Bhatt, Trushna | 2017 | Workplace health promotion | A |
| 72 | Bhaumik, S.; Watson, J. M.; Thorp, C. F.; Tyrer, F.; McGrother, C. W. | 2008 | Body mass index in adults with intellectual disability: Distribution, associations and service implications: A population-based prevalence study | A |
| 73 | Bheenick, Yajnah Neomi | 2018 | What factors organise a GP's aptitude to elicit the disclosure of psychological distress in men and how do they utilise this information? | A |
| 74 | Bikmaeva, E. F.; Godunova, A. R.; Musin, S. G.; Meshcheryakova, V. D. | 2013 | Organization of care for patients with cerebrovascular disease in a single-taken large industrial area with a population of 500 000 people naberezhnye chelny, Tatarstan | A |
| 75 | Billstedt, E.; Nilsson, G.; Leffler, L.; Carlsson, L.; Olsson, I.; Fernell, E.; Gillberg, C. | 2020 | Cognitive functioning in a representative cohort of preschool children with febrile seizures | A |
| 76 | Bjerre, B.; Marques, P.; Selén, J.; Thorsson, U. | 2007 | A Swedish alcohol ignition interlock programme for drink-drivers: Effects on hospital care utilization and sick leave | A |
| 77 | Blake, Holly; , Hussain, Basharat; , Hand, Jenny; , Juma, Amdani;  and Evans, Catrin | 2019 | Employers' views of the "Healthy Hub Roadshow": A workplace HIV testing intervention in England | A |
| 78 | Blake, H.; Hussain, B.; Hand, J.; Rowlands, D.; Juma, A.; Evans, C. | 2018 | Employee perceptions of a workplace HIV testing intervention | A |
| 79 | Blake, H.; Somerset, S.; Evans, C. | 2020 | Development and fidelity testing of the test@work digital toolkit for employers on workplace health checks and opt-in HIV testing | A |
| 80 | Blariza, M. G.; Calvano, L.; Pedrozo, W. R.; Martinez, M. A.; Bonneau, G. A. | 2019 | Obesity, hypertension, metabolic syndrome and type 2 diabetes mellitus in presumably healthy blood donor individuals of the Blood, Tissue and Biological Bank from the city of Posadas | A |
| 81 | Boongird, P.; Chamnan, P.; Laptikultham, S.; Krittayapoositpot, P.; Nitiyanant, W.; Aekplakorn, W.; Mangklabruks, A. | 2020 | Dose–response relationship between physical exercise and risk of physician-diagnosed dementia in 206 073 Thai community-dwelling men and women: HCUR study | A |
| 82 | Borg, A. M.; Salmelin, R.; Joukamaa, M.; Tamminen, T. | 2014 | Cutting a long story short? the clinical relevance of asking parents, nurses, and young children themselves to identify children's mental health problems by one or two questions | A |
| 83 | Bosanquet, K.; Gilbody, S.; Watt, I.; Shiers, D.; Coventry, P.; Owen, C. | 2018 | Closing the mortality gap: Meeting the physical health needs of people with serious mental illness (SMI) in primary care | A |
| 84 | Bosman, L. C.; , Roelen, C. A. M.; , Twisk, J. W. R.; , Eekhout, I.;  and Heymans, M. W. | 2019 | Development of Prediction Models for Sick Leave Due to Musculoskeletal Disorders | A |

| | | | | |
|---|---|---|---|---|
| 85 | Bosman, L. C.; Dijkstra, L.; Joling, C. I.; Heymans, M. W.; Twisk, J. W. R.; Roelen, C. A. M. | 2018 | Prediction models to identify workers at risk of sick leave due to low-back pain in the dutch construction industry | A |
| 86 | Bosman, L. C.; Twisk, J. W. R.; Geraedts, A. S.; Heymans, M. W. | 2020 | Effect of Partial Sick Leave on Sick Leave Duration in Employees with Musculoskeletal Disorders | A |
| 87 | Bougault, V.; , Drouard, F.; , Legall, F.; , Dupont, G.; and Wallaert, B. | 2017 | Allergies and Exercise-Induced Bronchoconstriction in a Youth Academy and Reserve Professional Soccer Team | A |
| 88 | Bourne, C.; Zablotska, I.; Williamson, A.; Calmette, Y.; Guy, R. | 2012 | Promotion and uptake of a new online partner notification and retesting reminder service for gay men | A |
| 89 | Brailsford, S.; Byrne, L.; Lattimore, S.; Ball, J. E.; McDonald, C.; Pitt, T. | 2012 | Bacterial screening: Clinical follow-up of positive donations, the English experience | A |
| 90 | Brekke, M.; Hunskaar, S.; Straand, J. | 2006 | Self-reported drug utilization, health, and lifestyle factors among 70-74 year old community dwelling individuals in Western Norway. The Hordaland Health Study (HUSK) | A |
| 91 | Brennan, Leah; and Murphy, Kylie | 2018 | Cognitive behavioural intervention for obesity | A |
| 92 | Bressington, Daniel T.; , Mui, Jolene; , Cheung, Eric F. C.; , Petch, Joel; , Clark, Allan B.; and Gray, Richard | 2013 | The prevalence of metabolic syndrome amongst patients with severe mental illness in the community in Hong Kong—A cross sectional study | A |
| 93 | Britton, J. P.; Dowell, A. C.; Whelan, P. | 1989 | Dipstick haematuria and bladder cancer in men over 60: Results of a community study | A |
| 94 | Brockmann, P. E.; , Diaz, B.; , Damiani, F.; , Villarroel, L.; , Nunez, F.; and Bruni, O. | 2016 | Impact of television on the quality of sleep in preschool children | A |
| 95 | Browning, C.; , Chapman, A.; , Cowlishaw, S.; , Li, Z. X.; , Thomas, S. A.; , Yang, H.; and Zhang, T. H. | 2011 | The Happy Life Club (TM) study protocol: A cluster randomised controlled trial of a type 2 diabetes health coach intervention | A |
| 96 | Browning, C.; , Chapman, A.; , Yang, H.; , Liu, S.; , Zhang, T. H.; , Enticott, J. C.; and Thomas, S. A. | 2016 | Management of type 2 diabetes in China: the Happy Life Club, a pragmatic cluster randomised controlled trial using health coaches | A |
| 97 | Brutting, J.; , Druschke, D.; , Spitzer, S.; and Seibt, R. | 2018 | HEALTH STATUS OF LONG-TERM SICK LEAVE AND WORKING FEMALE TEACHERS IN GERMANY: A CROSS-SECTIONAL STUDY | A |
| 98 | Bryce, G.; Wilkinson, P.; Nicholson, S.; Jeffery, A.; Hankins, M.; Jackson, D. | 2011 | A study to assess the acceptability, feasibility and cost-effectiveness of universal HIV testing with newly registering patients (aged 16-59) in primary care | A |
| 99 | Byles, J.; Leigh, L.; Chojenta, C.; Loxton, D. | 2014 | Adherence to recommended health checks by women in mid-life: data from a prospective study of women across Australia | A |
| 100 | Cabrera, M.; , Sanchez-Chaparro, M. A.; , Valdivielso, P.; , Quevedo-Aguado, L.; , Catalina-Romero, C.; , Fernandez-Labandera, C.; , Ruiz-Moraga, M.; , Gonzalez-Santos, P.; , Calvo-Bonacho, E.; and Ris, Icaria Ibermutuamur CArdiovasc | 2014 | Prevalence of atherogenic dyslipidemia: Association with risk factors and cardiovascular risk in Spanish working population. "ICARIA" study | A |

| | | | | |
|---|---|---|---|---|
| 101 | Campbell, B.; Carlson-Phillips, A.; Woolf, K. | 2011 | Core Performance Workplace Wellness: Positive impact on body composition | A |
| 102 | Campbell, S.; , Lynch, J.; , Esterman, A.; and McDermott, R. | 2011 | Pre-pregnancy predictors linked to miscarriage among Aboriginal and Torres Strait Islander women in North Queensland | A |
| 103 | Campbell, S. K.; , Lynch, J.; , Esterman, A.; and McDermott, R. | 2013 | Pre-pregnancy predictors of hypertension in pregnancy among Aboriginal and Torres Strait Islander women in north Queensland, Australia; a prospective cohort study | A |
| 104 | Cao, X.; , Wang, D. L.; , Zhou, J. S.; and Chen, Z. H. | 2017 | Comparison of lipoprotein derived indices for evaluating cardio-metabolic risk factors and subclinical organ damage in middle-aged Chinese adults | A |
| 105 | Carey, I. M.; Hosking, F. J.; Harris, T.; DeWilde, S.; Beighton, C.; Shah, S. M.; Cook, D. G. | 2017 | Do health checks for adults with intellectual disability reduce emergency hospital admissions? Evaluation of a natural experiment | A |
| 106 | Carlson-Phillips, A.; Campbell, B.; Woolf, K. | 2011 | Core performance workplace wellness: Positive impact on cardiovascular disease risk and fitness | A |
| 107 | Carrington, M. J.; Stewart, S. | 2011 | Providing effective primary prevention to vulnerable communities: Impact of a nurse-led, individual and community-based primary prevention program | A |
| 108 | Carstensen, Kathrine; , Brostrøm Kousgaard, Marius; and Burau, Viola | 2018 | Sustaining an intervention for physical health promotion in community mental health services: A multisite case study | A |
| 109 | Chae, J. M.; Cheon, J. H.; Moon, J. Y.; Oh, T. Y. | 2015 | The study for reliability and validity of smartphone applications to measure scoliosis | A |
| 110 | Chamnan, P.; Boongird, P.; Laptikultham, S.; Nitiyanont, W.; Aekplakorn, W. | 2018 | Physical activity as key risk factors for incident dementiain a thai population: Health checks ubon ratchathani (HCUR) study | A |
| 111 | Chan, O. K.; Suen, S. S. H.; Lao, T. T. H.; Leung, V. K. T.; Yeung, S. W.; Leung, T. Y. | 2009 | Determinants of hepatitis B vaccine uptake among pregnant Chinese women in Hong Kong | A |
| 112 | Chang, Y.; Ryu, S.; Sung, E.; Woo, H. Y.; Cho, S. I.; Yoo, S. H.; Ahn, H. Y.; Choi, N. K. | 2009 | Weight gain within the normal weight range predicts ultrasonographically detected fatty liver in healthy Korean men | A |
| 113 | Chatterjee, R.; , Chapman, T.; , Brannan, M. G.; and Varney, J. | 2017 | GPs' knowledge, use, and confidence in national physical activity and health guidelines and tools: a questionnaire-based survey of general practice in England | A |
| 114 | Chattopadhyay, K.; Biswas, M.; Moore, R. | 2020 | NHS Health Check and healthy lifestyle in Leicester, England: analysis of a survey dataset | A |
| 115 | Chaudhari, T. N.; Rajkumari, Ch N. | 2016 | Association of blood groups in diabeties mellitus | A |
| 116 | Chen, H. F.; Li, C. Y.; Chen, P.; See, T. T.; Lee, H. Y. | 2006 | Seroprevalence of hepatitis B and C in type 2 diabetic patients | A |
| 117 | Chen, M. J.; Weng, S. S. | 2017 | Psychological symptoms among hospital nurses in Taiwan: A cross sectional study | A |

| | | | | |
|---|---|---|---|---|
| 118 | Chen, M. Y.; Donovan, B.; Harcourt, C.; Morton, A.; Moss, L.; Wallis, S.; Cook, K.; Batras, D.; Groves, J.; Tabrizi, S. N.; Garland, S.; Fairley, C. K. | 2010 | Estimating the number of unlicensed brothels operating in Melbourne | A |
| 119 | Chen, W.; , Li, T. Y.; , Zou, G. Y.; , Li, X. D.; , Shi, L. Y.; , Feng, S. S.; , Shi, J. R.; , Zhou, F. J.; , Han, S. Q.;  and Ling, L. | 2016 | Study protocol: a cluster randomized controlled trial to assess the effectiveness of a multi-pronged behavioural intervention to improve use of personal protective equipment among migrant workers exposed to organic solvents in small and medium-sized enterprises | A |
| 120 | Chen, W.; , Li, T. Y.; , Zou, G. Y.; , Renzaho, A. M. N.; , Li, X. D.; , Shi, L. Y.;  and Ling, L. | 2019 | Results of a Cluster Randomized Controlled Trial to Promote the Use of Respiratory Protective Equipment among Migrant Workers Exposed to Organic Solvents in Small and Medium-Sized Enterprises | A |
| 121 | Chen, W.; Li, T.; Zou, G.; Renzaho, A. M. N.; Li, X.; Shi, L.; Ling, L. | 2019 | Results of a cluster randomized controlled trial to promote the use of respiratory protective equipment among migrant workers exposed to organic solvents in small and medium-sized enterprises | A |
| 122 | Chen, Y. F.; , Wang, C. X.; , Yuan, Z. S.; , Zhang, W. C.; , Liu, Y. F.; , Li, X. Y.; , Dayimu, A.; , Xu, Y. B.; , Xue, F. Z.;  and Zhang, C. Q. | 2016 | [Association between hematocrit and risk of incident hypertension: a cohort study] | A |
| 123 | Chen, Y. Y.; Chen, T. H.; Su, M. Y.; Ning, H. C.; Kuo, C. J.; Lin, W. P.; Ho, Y. P.; Lin, C. J.; Hsu, C. M.; Chiu, C. T.; Chen, P. C. | 2014 | Accuracy of immunochemical fecal occult blood test for detecting colorectal neoplasms in individuals undergoing health check-ups | A |
| 124 | Ching, J. Y. L.; Luk, A. K. C.; Tang, W. W. Y.; Wong, M. C. S.; Hirai, H. W.; Lam, T. Y. T.; Sung, J. J. Y. | 2012 | Mailing invitations for colorectal cancer (CRC) screening programme in Hong Kong: A comparison between private and public estates | A |
| 125 | Chinn, D. | 2019 | 'So this just gives you a bit of information': How accessible easy read health information is used in health care interactions with people with intellectual disabilities | A |
| 126 | Chinn, Deborah | 2019 | An empirical examination of the use of easy read health information in health consultations involving patients with intellectual disabilities | A |
| 127 | Chinn, D. | 2020 | An empirical examination of the use of Easy Read health information in health consultations involving patients with intellectual disabilities | A |
| 128 | Chinn, D. J.; , White, M.; , Howel, D.; , Harland, J. O. E.;  and Drinkwater, C. K. | 2006 | Factors associated with non-participation in a physical activity promotion trial | A |
| 129 | Chng, A. Y. R.; Chakera, A.; Huber, J. | 2017 | The nine essential health checks on people with maturity onset diabetes of the young (MODY): An audit of clinical care | A |
| 130 | Cho, E. R.; Shin, A.; Choi, K. S.; Lee, H. Y.; Kim, J. | 2010 | Factors associated with use of ultrasonography screening for hepatocellular carcinoma among hepatitis B or C carriers | A |
| 131 | Cho, E. Y.; Lee, Y. W.; Kim, H. S. | 2005 | The effect of job stress and lifestyle on blood lipid levels in male aircrew personnel | A |
| 132 | Cho, I. H.; Kong, E. J.; Chun, K. A. | 2017 | Evaluation of fatty liver with Dixon sequence for attenuation correction and FDG uptake in PET/MR | A |

| | | | | |
|---|---|---|---|---|
| 133 | Cho, T.; Negoro, H.; Saka, Y.; Morikawa, M.; Kishimoto, T. | 2016 | Two-year prognosis after residential treatment for patients with alcohol dependence: Three chief guidelines for sobriety in Japan | A |
| 134 | Cho, T.; Saka, Y.; Morikawa, M.; Kishimoto, T. | 2014 | Motivational interviewing in the clinical settings in Japan | A |
| 135 | Choi, H. Y.; Kim, Y. G.; Choi, J. I.; Choi, Y. Y.; Boo, K. Y.; Kim, D. Y.; Lee, K. N.; Shim, J.; Kim, J. S.; Han, K. D.; Kim, Y. H. | 2019 | Frequent drinking is a more important risk factor for new-onset atrial fibrillation than binge drinking: A nationwide population-based study | A |
| 136 | Chou, M. Y.; Hsu, P. I.; Chou, S. L.; Chou, Y. M.; Wang, F. W.; Hsueh, K. C.; Chen, L. K.; Hwang, S. J.; Tu, M. S. | 2007 | Factors related to incomplete flexible sigmoidoscopy among adult Chinese in Taiwan | A |
| 137 | Clarke, W.; Turner, K.; Priestley, C.; Chapman, C.; Callaghan, S.; Callaghan, S.; Scofield, S. | 2013 | Increasing the uptake of sexually transmitted infection screening in a high-risk population | A |
| 138 | Claus, M.; Schuster, M.; Webendörfer, S.; Oberlinner, C. | 2018 | Sociodemographic and health-related factors associated with temporary work disability in a large German chemical company: Results of a cross-sectional study | A |
| 139 | Claus, M.; Schuster, M.; Webendörfer, S.; Groneberg, D. A.; Jähner, J.; Schiffmann, D. | 2019 | Prevalence of back pain in employees of a German chemical company: Results of a large cross-sectional study | A |
| 140 | Coates, C.; Meisman, A.; Gordon, C.; Chandler, E.; Braverman, P. | 2019 | Evaluation of A Health Education Module About Accessing Community Based Healthcare Services For Youth In A Juvenile Detention Facility | A |
| 141 | Coley, N.; , Rosenberg, A.; , van Middelaar, T.; , Soulier, A.; , Barbera, M.; , Guillemont, J.; , Steensma, J.; , Igier, V.; , Eskelinen, M.; , Soininen, H.; , van Charante, E. M.; , Richard, E.; , Kivipelto, M.; , Andrieu, S.; , Sindi, S.; , Solomon, A.; , Hartmann, T.; , Brayne, C.; , van Gool, P.; , Beishuizen, C.; , Jongstra, S.; , van Wanrooij, L.; , Hoevenaar-Blom, M.; , Ngandu, T.; , Mangiasche, F.; , Meiller, Y.; , van de Groep, B.; , Grp;, Mind-Ad and Grp, Hatice | 2019 | Original Study Older Adults' Reasons for Participating in an eHealth Prevention Trial: A Cross-Country, Mixed-Methods Comparison | A |
| 142 | Collier, Stephanie; , Tesfaye, Markos; , Henderson, Tanya; , Abafita, Jemal; , Tarbi, Elise; , Pietras, Alison; and Chemali, Zeina | 2014 | Microfinance and women's health: An evaluation of women's health behaviors in Jimma, Ethiopia | A |
| 143 | Coolidge, J. A.; Herner, E. B.; Bülow, S. L.; Biering-Sørensen, S. | 2010 | Obesity and life-style in 5-8-year-old children | A |
| 144 | Cooper, Cary L.; and Faragher, E. Brian | 1993 | Psychosocial stress and breast cancer: The inter-relationship between stress events, coping strategies and personality | A |
| 145 | Cooper, Sally-Ann; , Smiley, Elita; , Morrison, Jillian; , Williamson, Andrew; and Allan, Linda | 2007 | An epidemiological investigation of affective disorders with a population-based cohort of 1023 adults with intellectual disabilities | A |
| 146 | Cooper, S. A.; , Smiley, E.; , Jackson, A.; , Finlayson, J.; , Allan, L.; , Mantry, D.; and Morrison, J. | 2009 | Adults with intellectual disabilities: Prevalence, incidence and remission of aggressive behaviour and related factors | A |

| | | | | |
|---|---|---|---|---|
| 147 | Copin, Mme Nane; , Geslain, Christine; , Vol, Sylviane; , Errard, Gabrielle; , Lepinay, Patrick; , Gusto, Gaëlle; , Lantieri, Olivier; and Tichet, Jean | 2015 | Cannabis et jeunes consultants: De centres d'examens de santé | A |
| 148 | Cornelissen, R. | 2019 | ES08.01 Participation of the Target Population in Lung Cancer Screening | A |
| 149 | Cornelius, V. R.; McDermott, L.; Forster, A. S.; Ashworth, M.; Wright, A. J.; Gulliford, M. C. | 2018 | Automated recruitment and randomisation for an efficient randomised controlled trial in primary care | A |
| 150 | Coyne-Beasley, T.; Reiter, P. L.; Desousa, N.; Ford, C. A.; Lees, A. C.; Miles, D.; Austin, S.; Brewer, N. T. | 2011 | Awareness is not enough: The need to increase meningococcal vaccine uptake | A |
| 151 | Cuskelly, M.; Jobling, A.; Mallardo, M. | 2019 | Mental health and well being of adults with down syndrome | A |
| 152 | D'Onise, K.; , McDermott, R. A.; and Campbell, S. K. | 2013 | Benefits of modest weight or waist circumference loss in a remote North Queensland Indigenous population | A |
| 153 | Dalager, T.; , Justesen, J. B.; , Murray, M.; , Boyle, E.; and Sjogaard, G. | 2016 | Implementing intelligent physical exercise training at the workplace: health effects among office workers-a randomized controlled trial | A |
| 154 | Dalal, K.; and Dawad, S. | 2009 | Non-utilization of public healthcare facilities: examining the reasons through a national study of women in India | A |
| 155 | Davis, Alissa; , Terlikbayeva, Assel; , Terloyeva, Dina; , Primbetova, Sholpan; and El-Bassel, Nabila | 2017 | What prevents central Asian migrant workers from accessing HIV testing? Implications for increasing HIV testing uptake in Kazakhstan | A |
| 156 | Davison, K. L.; , Reynolds, C. A.; , Andrews, N.; , Brailsford, S. R.; and Grp, U. K. Blood Donor Survey Steering | 2015 | Getting personal with blood donors - the rationale for, methodology of and an overview of participants in the UK blood donor survey | A |
| 157 | De Bats, F.; Nitenberg, C. V.; Fantino, B.; Kodjikian, L. | 2013 | ' DODMLA ' : Organized screening of agerelated macular degeneration by delayed reading of retinophotography | A |
| 158 | Dearnaley, D. P.; Kirby, R. S.; Kirk, D.; Malone, P.; Simpson, R. J.; Williams, G. | 1999 | Diagnosis and management of early prostate cancer. Report of a British Association of Urological Surgeons Working Party | A |
| 159 | Debattista, J.; Bryson, G.; Roudenko, N.; Dwyer, J.; Kelly, M.; Hogan, P.; Patten, J. | 2007 | Pilot of non-invasive (oral fluid) testing for HIV within a clinical setting | A |
| 160 | Deeks, A.; Lombard, C.; Michelmore, J.; Teede, H. | 2009 | The effects of gender and age on health related behaviors | A |
| 161 | Del Carpio, X. V.; , Loayza, N. V.; and Wada, T. | 2016 | The Impact of Conditional Cash Transfers on the Amount and Type of Child Labor | A |
| 162 | del Rosario, C. R.; , Diaz, S. N.; , de Carlos, P. G.; , Palmero, I. R.; , Mahtani, V. M.; , Rodriguez, M. A. H.; , Rodriguez, L. M.; , Martinez, C. O.; , Moreno, S. D.; and Bilbao, J. L. A. | 2008 | Health care for African immigrants arriving in the Canary Islands a descriptive study | A |
| 163 | den Engelsen, C.; , Koekkoek, P. S.; , Godefrooij, M. B.; , Spigt, M. G.; and Rutten, G. E. | 2014 | Screening for increased cardiometabolic risk in primary care: a systematic review | A |
| 164 | Denney-Wilson, E.; Robinson, A.; Laws, R.; Harris, M. F. | 2011 | Child obesity prevention in primary care: The Healthy 4 Life pilot study | A |

| | | | | |
|---|---|---|---|---|
| 165 | Denney-Wilson, E.; Vagholkar, S.; Wan, Q.; McKenzie, S.; Harris, M. F. | 2011 | Patient characteristics and outcomes of weight management advice in primary care | A |
| 166 | Dhanapalaratnam, R.; , Fanaian, M.;  and Harris, M. F. | 2011 | Lifestyle intervention A study on maintenance in general practice | A |
| 167 | Diaz, J. J.;  and Saldarriaga, V. | 2019 | Encouraging use of prenatal care through conditional cash transfers: Evidence from JUNTOS in Peru | A |
| 168 | Dickson, J.; Quaife, S.; Horst, C.; Hall, H.; Tisi, S.; Mullin, A.; Farrelly, L.; Gyertson, K.; Nnorom, S.; Levermore, C.; Bojang, F.; Anastasiadis, T.; Sennett, K.; Clarke, C.; Allen, B.; Hamilton, S.; Hartmann, A.; Hackshaw, A.; Janes, S. | 2020 | The SUMMIT study: invitation strategy and screening uptake of the first 36,680 invited | A |
| 169 | Dickson, N.; Ludlam, A.; Saxton, P.; Hughes, A. | 2015 | Self-reported STIs and sexual health checks in a cross-sectional study of gay and bisexual men in New Zealand | A |
| 170 | Diller, G. P.; Helm, P.; Gundlach, C.; Baumgartner, H.; Bauer, U. M. M. | 2019 | Sexual activity and dysfunction in adult patients with congenital heart disease | A |
| 171 | Dive, C. | 2019 | MS12.01 Circulating Biomarkers | A |
| 172 | Dodani, S.; Qureshi, R.; Ali, B. S. | 1999 | Syndrome X and family practitioners | A |
| 173 | Dol, J.; , Kohi, T.; , Campbell-Yeo, M.; , Murphy, G. T.; , Aston, M.;  and Mselle, L. | 2019 | Exploring maternal postnatal newborn care postnatal discharge education in Dar es Salaam, Tanzania: Barriers, facilitators and opportunities | A |
| 174 | Dolak, F.; , Sedova, L.; , Novakova, D.;  and Olisarova, V. | 2016 | Approach to prevention of obesity of Roma population in the Region of South Bohemia with focus on selected eating behaviors | A |
| 175 | Doshi, Maulik Sumantbhai; , Kulkarni, Shaunak P.; , Ghia, Canna J.; , Gogtay, Nithya J.;  and Thatte, Urmila Mukund | 2013 | Evaluation of factors that motivate participants to consent for non-therapeutic trials in India | A |
| 176 | Dummer, J. | 2012 | Sodium reduction in Canadian food products: With the Health Check program | A |
| 177 | Dunn, K. M.; Das, S.; Das, R. | 2004 | Male Reproductive Health: A village based study of camp attenders in rural India | A |
| 178 | Durako, A. R.; Mishkel, G. | 2018 | Creating the virtual cardiac surgical home: Implementation of an app based enhanced recovery after surgery platform to improve outcomes and patient engagement | A |
| 179 | Durán, M. J.; Valdés, J.; Velázquez, P.; Sancho, A.; Sánchez, M. J.; Mera, C.; Diéguez, M. J. | 2016 | Election of IUD as a contraception method | A |
| 180 | Durao, S.; , Ajumobi, O.; , Kredo, T.; , Naude, C.; , Levitt, N. S.; , Steyn, K.; , Bradshaw, D.;  and Young, T. | 2015 | Evidence insufficient to confirm the value of population screening for diabetes and hypertension in low- and middle-income settings | A |
| 181 | Dutta, N. | 2018 | STREET CHILDREN IN INDIA: A STUDY ON THEIR ACCESS TO HEALTH AND EDUCATION | A |
| 182 | Dyavarishetty, P.; Kowali, S. | 2016 | Breast cancer, awareness and screening programme in Mumbai | A |

| | | | | |
|---|---|---|---|---|
| 183 | Elliott, A. F.; , Chou, C. F.; , Zhang, X.; , Crews, J. E.; , Saaddine, J. B.; , Beckles, G. L.; and Owens-Gary, M. D. | 2010 | Eye-care utilization among women aged ≥40 years with eye diseases—19 states, 2006-2008 | A |
| 184 | Emdadi, M.; Safarian, M.; Doosti, H. | 2011 | Standardized percentile curves of body mass index of northeast Iranian children aged 25 to 60 months | A |
| 185 | Emily, N.; Joyce, O.; Robert, L. R.; Anzala, A. | 2016 | Clinical research volunteers' perceptions and experiences of screening for enrolment at KAVI-Institute of Clinical Research, Kenya | A |
| 186 | Empana, J. P.; Gaye, B. G.; Prugger, C. P.; Plichart, M. P.; Perier, M. C. P.; Thomas, F. T.; Pannier, B. P.; Boutouyrie, P. B.; Jouven, X. J. | 2015 | Ideal cardiovascular health and carotid stiffness. The Paris Prospective Study III | A |
| 187 | Enocson, A.; , Jolly, K.; , Jordan, R. E.; , Fitzmaurice, D. A.; , Greenfield, S. M.; and Adab, P. | 2018 | Case-finding for COPD in primary care: a qualitative study of patients' perspectives | A |
| 188 | Ensslin, A. S.; Koller, M. F. | 2014 | Convulsions and hypoglycemia due to tetramethyl succinonitrile intoxication in the polyvinyl chloride (PVC) industry: A 4-year follow-up | A |
| 189 | Esler, D.; Johnston, F.; Thomas, D.; Davis, B. | 2008 | The validity of a depression screening tool modified for use with Aboriginal and Torres Strait Islander people | A |
| 190 | Evangelopoulos, A.; Vallianou, N.; Georgousopoulou, E.; Bountziouka, V.; Bonou, M.; Vogiatzakis, E.; Avgerinos, P.; Barbetseas, J.; Panagiotakos, D. | 2017 | Mediterranean diet and serum cystatin C levels | A |
| 191 | Famoroti, T. O.; Fernandes, L.; Chima, S. C. | 2013 | Stigmatization of people living with HIV/AIDS by healthcare workers at a tertiary hospital in KwaZulu-Natal, South Africa: a cross-sectional descriptive study | A |
| 192 | Faruqi, N.; Stocks, N.; Denney-Wilson, E.; Liaw, S. T.; Spooner, C.; Lloyd, J.; Laws, R.; El-Haddad, N.; Hermiz, O.; Harris, M. | 2014 | Better management of weight in general practice-study protocol | A |
| 193 | Faruqi, N.; Stocks, N.; Spooner, C.; el Haddad, N.; Harris, M. F. | 2015 | Research protocol: Management of obesity in patients with low health literacy in primary health care | A |
| 194 | Fetohy, E. M. | 2004 | Impact of a simple health education program about antenatal care on knowledge, attitudes, subjective norms and intention of pregnant women | A |
| 195 | Fhadil, S.; Wright, P.; Khuu, M.; Hazelrigg, B.; Jung, A.; Ruthsatz, O.; Antoniou, S. | 2019 | Lipid modification therapy for primary prevention of cardiovascular disease | A |
| 196 | Fidoe, D.; Hill, S.; Gaynor, E.; Field, J.; Duffy, S.; Ledson, M.; Grundy, S. | 2018 | Liverpool healthy lung project: Detection of undiagnosed airways disease and significant other findings in a risk stratified lung health check | A |

| | | | | |
|---|---|---|---|---|
| 197 | Fischer, J. E.; Jahns, O.; Genser, B.; Nauroth, P.; Knoll, K. P. | 2019 | Predicting sick-leave rates at the work-group level: Why do averaged psychosocial work conditions matter more than averaged medical conditons or group average health behaviors? | A |
| 198 | Fischer, J. E.; Knoll, K. P.; Nauroth, P.; Jahns, O. | 2019 | Changing eating behavior in canteens for good: Preliminary results from a multimodal complex intervention | A |
| 199 | FitzSimons, D.; Hendrickx, G.; Lernout, T.; Badur, S.; Vorsters, A.; Van Damme, P. | 2014 | Incentives and barriers regarding immunization against influenza and hepatitis of health care workers | A |
| 200 | Foliaki, S.; Matheson, A. | 2015 | Barriers to cervical screening among Pacific women in a New Zealand Urban Population | A |
| 201 | Forsyth, P.; Ali, S.; Ameen, M.; Khan, R.; Khan, F.; Sheikh, A. R.; Scoular, A.; Lowrie, R. | 2012 | Pharmacist-led anticipatory care for the South Asian community | A |
| 202 | Forward, K.; Hatchette, J. | 2012 | Improved utilization of nugent scores after physician specific feedback of testing results with peer comparison | A |
| 203 | Fourie, J. M.; De Villiers, A.; Draper, C.; Gwebushe, N.; April, E.; Steyn, N. P.; Lambert, E. V. | 2013 | Cardiovascular risk profile of primary school educators participating in the healthkick study-possible implications | A |
| 204 | François, P.; , Guyomard, A.; , Baudet, D.; , Dubois-Fabing, D.; , Boussuges, S.; , Perrin, F.; and Seigneurin, A. | 2014 | [Evaluation of an obesity prevention program for school-aged children in deprived urban areas] | A |
| 205 | Fujibayashi, K.; , Yokokawa, H.; , Gunji, T.; , Sasabe, N.; , Okumura, M.; , Iijima, K.; , Haniu, T.; , Hisaoka, T.; and Fukuda, H. | 2015 | Utility of 75-g oral glucose tolerance test results and hemoglobin a1c values for predicting the incidence of diabetes mellitus among middle-aged Japanese men -a large-scale retrospective cohort study performed at a single hospital | A |
| 206 | Fujii, R.; , Ueyama, J.; , Aoi, A.; , Ichino, N.; , Osakabe, K.; , Sugimoto, K.; , Suzuki, K.; , Hamajima, N.; , Wakai, K.; and Kondo, T. | 2018 | Oxidized human serum albumin as a possible correlation factor for atherosclerosis in a rural Japanese population: the results of the Yakumo Study | A |
| 207 | Fujii, R.; , Yamada, H.; , Yamazaki, M.; , Munetsuna, E.; , Ando, Y.; , Ohashi, K.; , Ishikawa, H.; , Shimoda, H.; , Sakata, K.; , Ogawa, A.; , Kobayashi, S.; , Suzuki, K.; and Grp, Rias Study | 2019 | Circulating microRNAs (miR-126, miR-197, and miR-223) are associated with chronic kidney disease among elderly survivors of the Great East Japan Earthquake | A |
| 208 | Fujii, R.; Yamada, H.; Munetsuna, E.; Yamazaki, M.; Ohashi, K.; Ishikawa, H.; Maeda, K.; Hagiwara, C.; Ando, Y.; Hashimoto, S.; Hamajima, N.; Suzuki, K. | 2020 | Associations of Circulating MicroRNAs (miR-17, miR-21, and miR-150) and Chronic Kidney Disease in a Japanese Population | A |
| 209 | Fujimatsu, D.; Kotooka, N.; Inoue, T.; Nishiyama, M.; Node, K. | 2009 | Association between high molecular weight adiponectin levels and metabolic parameters | A |
| 210 | Fujimoto, A. I.; Hashimoto, M.; Hoteya, S.; Iizuka, T.; Ogawa, O.; Mitani, T.; Matsui, A.; Nakamura, M.; Kikuchi, D.; Yamashita, S.; Furuhata, T.; Yamada, A.; Igarashi, Y.; Kaise, M. | 2012 | Obesity and gastrointestinal disease | A |

| | | | | |
|---|---|---|---|---|
| 211 | Fujiwara, Y.; , Nishi, M.; , Watanabe, N.; , Lee, S.; , Inoue, K.; , Yoshida, H.; , Sakuma, N.; , Kureta, Y.; , Ishii, K.; , Uchida, H.; , Kakuno, F.;  and Shinkai, S. | 2006 | [An intergenerational health promotion program involving older adults in urban areas. "Research of Productivity by Intergenerational Sympathy (REPRINTS)": first-year experience and short-term effects] | A |
| 212 | Fujiwara, Y.; Suzuki, H.; Yasunaga, M.; Sugiyama, M.; Ijuin, M.; Sakuma, N.; Inagaki, H.; Iwasa, H.; Ura, C.; Yatomi, N.; Ishii, K.; Tokumaru, A. M.; Homma, A.; Nasreddine, Z.; Shinkai, S. | 2010 | Brief screening tool for mild cognitive impairment in older Japanese: Validation of the Japanese version of the Montreal Cognitive Assessment | A |
| 213 | Fukino, Y.; Ikeda, A.; Maruyama, K.; Aoki, N.; Okubo, T.; Iso, H. | 2008 | Randomized controlled trial for an effect of green tea-extract powder supplementation on glucose abnormalities | A |
| 214 | Fukuda, Y.; Nakamura, K.; Takano, T. | 2005 | Accumulation of health risk behaviours is associated with lower socioeconomic status and women's urban residence: A multilevel analysis in Japan | A |
| 215 | Fukunaga, I.; Jitsunari, F.; Takeda, N.; Maruyama, Y.; Kitamado, T.; Shiraishi, H.; Hoshikawa, Y.; Asakawa, F.; Kasai, S. | 1998 | Factors associated with participation in chest X-ray screening by young and middle aged residents | A |
| 216 | Fukushima, K.; , Fukushima, N.; , Sato, H.; , Yokota, J.;  and Uchida, K. | 2020 | Association between nutritional level, menstrual-related symptoms, and mental health in female medical students | A |
| 217 | Fukushima, S.; Nakagami, T.; Suto, C.; Hirose, A.; Uchigata, Y. | 2013 | Prevalence of retinopathy and its risk factors in a Japanese population | A |
| 218 | Gamble, D. T.; , Clark, A. B.; , Luben, R. N.; , Wareham, N. J.; , Khaw, K. T.;  and Myint, P. K. | 2018 | Baseline anticholinergic burden from medications predicts incident fatal and non-fatal stroke in the EPIC-Norfolk general population | A |
| 219 | Gandhi, M.; Arivazhagan, R.; Sangeetha, R.; Swaminathan, S. | 2017 | Association between insulin, ghrelin, homeostasis model assessment-insulin resistance, homeostasis model assessment-β, waist-to-hip ratio and body mass index to plasma glucose and glycosylated hemoglobin and its clinical usefulness in type 2 diabetes mellitus patients with obesity | A |
| 220 | Garedew-Kifelew, L.; , Wondafrash, N.;  and Feleke, A. | 2014 | Identification of drug-resistant Salmonella from food handlers at the University of Gondar, Ethiopia | A |
| 221 | Gaye, B.; Perrier, M. C.; Thomas, F.; Guibout, C.; Pannier, B.; Boutouyrie, P.; Jouven, X.; Empana, J. P. | 2016 | Ideal cardiovascular health and vascular aging | A |
| 222 | Genc, M.; , Ruusuvaara, L.;  and Mardh, P. A. | 1993 | AN ECONOMIC-EVALUATION OF SCREENING FOR CHLAMYDIA-TRACHOMATIS IN ADOLESCENT MALES | A |
| 223 | Gerdle, B.; , Brulin, C.; , Elert, J.; , Eliasson, P.;  and Granlund, B. | 1995 | Effect of a general fitness program on musculoskeletal symptoms, clinical status, physiological capacity, and perceived work environment among home care service personnel | A |

| | | | | |
|---|---|---|---|---|
| 224 | Gharipour, M.; Sadeghi, M.; Nouri, F.; Nezafati, P.; Qader, S. S.; Taheri, M.; Maghroun, M.; Abdalvand, A.; Soleimani, B.; Sarrafzadegan, N. | 2016 | Socioeconomic determinants and metabolic syndrome: Results from the Isfahan Healthy Heart Program | A |
| 225 | Ghimire, B.; Maroni, R.; Vulkan, D.; Shah, Z.; Gaynor, E.; Timoney, M.; Jones, L.; Arvanitis, R.; Ledson, M.; Lukehirst, L.; Rutherford, P.; Clarke, F.; Gardner, K.; Marcus, M. W.; Hill, S.; Fideo, D.; Mason, S.; Smith, S. G.; Quaife, S. L.; Fitzgerald, K.; Poirier, V.; Duffy, S. W.; Field, J. K. | 2019 | Evaluation of a health service adopting proactive approach to reduce high risk of lung cancer: The Liverpool Healthy Lung Programme | A |
| 226 | Gil Chung, W.; Kyun Cho, Y.; Joo Kim, H.; Wan Kim, T.; Kyu Jeon, W.; Ik Kim, B. | 2011 | Effect of non-alcoholic fatty liver disease on the development of type 2 diabetes in nonobese and non-diabetic korean men | A |
| 227 | Glover, G. | 2019 | Opportunities for using population-level data in health promotion for people with intellectual disabilities in england: Potential and realities | A |
| 228 | Gogo, E.; van Sluijs, R. M.; Cheung, T.; Gaskell, C.; Jones, L.; Alwan, N. A.; Hill, C. M. | 2019 | Objectively confirmed prevalence of sleep-related rhythmic movement disorder in pre-school children | A |
| 229 | Gonoi, W.; , Akai, H.; , Hagiwara, K.; , Akahane, M.; , Hayashi, N.; , Maeda, E.; , Yoshikawa, T.; , Tada, M.; , Uno, K.; , Ohtsu, H.; , Koike, K.;  and Ohtomo, K. | 2011 | Pancreas divisum as a predisposing factor for chronic and recurrent idiopathic pancreatitis: initial in vivo survey | A |
| 230 | González-Mesa, Ernesto; , Kabukcuoglu, Kamile; , Körükcü, Oznur; , Blasco, Marta; , Ibrahim, Nadia; , Cazorla-Granados, Olga;  and Kavas, Türker | 2019 | Correlates for state and trait anxiety in a multicultural sample of Turkish and Spanish women at first trimester of pregnancy | A |
| 231 | González-Mesa, Ernesto; , Kabukcuoglu, Kamile; , Körükcü, Oznur; , Blasco, Marta; , Ibrahim, Nadia;  and Kavas, Türker | 2018 | Cultural factors influencing antenatal depression: A cross-sectional study in a cohort of Turkish and Spanish women at the beginning of the pregnancy | A |
| 232 | González-Santos, P.; Valdivielso, P.; Cabrera, M.; Quevedo-Aguado, L.; Sánchez-Chaparro, M. A.; Calvo-Bonacho, E. | 2014 | Association of atherogenic dyslipidemia with cardiovascular risk in Spanish working population: Results from the icaria study | A |
| 233 | Gopalan, S. S.; Mutasa, R.; Friedman, J.; Das, A. | 2014 | Health sector demand-side financial incentives in low- and middle-income countries: A systematic review on demand- and supply-side effects | A |
| 234 | Goutbeek, A. M.; Rotteveel, J.; De Wit, M.; Walenkamp, M. J.; Goverde, M.; Bijl, J.; Finken, M.; Ijzerman, R. | 2016 | Putting theory into practice: Implementation of a transition program into routine diabetes care | A |
| 235 | Granollers Mercader, S.;  and Pont Ribas, A. | 1993 | [Nurse care in primary health care: diagnosis and follow-up of health problems] | A |
| 236 | Grassie, S. S.;  and Gevrek, S. C. | 2016 | Investigation of Hepatitis Serology and Occupational Exposure Risk to Viral Hepatitis in Hospital Housekeeping Staff | A |
| 237 | Greaves, C.; Gillison, F.; Stathi, A.; Bennett, P.; Reddy, P.; Dunbar, J.; Perry, R.; Messom, D.; Chandler, R.; Francis, M.; Davis, M.; Green, C.; Evans, P.; Taylor, G. | 2015 | Waste the waist: A pilot randomised controlled trial of a primary care based intervention to support lifestyle change in people with high cardiovascular risk | A |

| | | | | |
|---|---|---|---|---|
| 238 | Gregorich, M. G.; Urach, C.; Breitenecker, F. | 2016 | Cost-effectiveness analysis of colorectal cancer screening in Austria and assessment of the impact of cancer-causing risk factors | A |
| 239 | Griffiths, C.; , Sturdy, P.; , Brewin, P.; , Bothamley, G.; , Eldridge, S.; , Martineau, A.; , MacDonald, M.; , Ramsay, J.; , Tibrewal, S.; , Levi, S.; , Zumla, A.; and Feder, G. | 2007 | Educational outreach to promote screening for tuberculosis in in primary care: a cluster randomised controlled trial | A |
| 240 | Griniene, E.; Liutaite, N. | 2009 | School nurses' contribution to schoolchildren's future health | A |
| 241 | Großschädl, F.; , Titze, S.; , Burkert, N.; and Stronegger, W. J. | 2013 | Moderate- and vigorous-intensity exercise behaviour according to the Transtheoretical Model: associations with smoking and BMI among Austrian adults | A |
| 242 | Großschädl, F.; Titze, S.; Stronegger, W. J. | 2012 | Physical activity behavior change according to the transtheoretical model: Associations with BMI and smoking behavior | A |
| 243 | Grubb, N. R.; , Elder, D.; , Broadhurst, P.; , Reoch, A.; , Tassie, E.; and Neilson, A. | 2019 | Atrial fibrillation case finding in over 65 s with cardiovascular risk factors - Results of initial Scottish clinical experience | A |
| 244 | Gudka, S.; Marshall, L.; Creagh, A.; Clifford, R. M. | 2013 | To develop and measure the effectiveness and acceptability of a pharmacy-based chlamydia screening intervention in Australia | A |
| 245 | Gudmundsson, Olafur O.; , Magnusson, Pall; , Saemundsen, Evald; , Lauth, Bertrand; , Baldursson, Gisli; , Skarphedinsson, Gudmundur; and Fombonne, Eric | 2013 | Psychiatric disorders in an urban sample of preschool children | A |
| 246 | Gunji, T.; Sato, H.; Iijima, K.; Fujibayashi, K.; Okumura, M.; Sasabe, N.; Urabe, A.; Matsuhashi, N. | 2011 | Risk factors for erosive esophagitis: A cross-sectional study of a large number of Japanese males | A |
| 247 | Guo, S.; Wang, L.; Yan, R. | 2002 | Health service needs of women with reproductive tract infections in selected areas of China | A |
| 248 | Gupta, A.; Gupta, D.; Raizada, A.; Gupta, N. P.; Yadav, R.; Vinayak, K.; Tewari, V. | 2014 | A hospital based study on reference range of serum prostate specific antigen levels | A |
| 249 | Gurdogan, M.; Gurdogan, E. P.; Ozkan, U.; Kurt, C. | 2019 | What do college of sports students think about sudden cardiac death in athletes? | A |
| 250 | Guy, R. J.; Prestage, G. P.; Grulich, A.; Holt, M.; Conway, D. P.; Jamil, M. S.; Keen, P.; Cunningham, P.; Wilson, D. P. | 2015 | Potential public health benefits of HIV testing occurring at home in Australia | A |
| 251 | Hafner, M. B.; , Kolsek, M.; and Rebek, K. | 2014 | ALCOHOL DRINKING AMONG STUDENTS OF THE UNIVERSITY OF LJUBLJANA | A |
| 252 | Haga, H.; Konta, T.; Saito, T.; Okumoto, K.; Katsumi, T.; Mizuno, K.; Nishina, T.; Kato, T.; Kubota, I.; Kayama, T.; Ueno, Y. | 2016 | The association between alanine aminotransferase and renal function in the Japan population: The Yamagata (Takahata) study | A |
| 253 | Hakkanen, P.; , Ketola, E.; and Laatikainen, T. | 2018 | Screening and treatment of obesity in school health care - the gap between clinical guidelines and reality | A |
| 254 | Hall, R. H. | 2003 | Promoting men's health | A |

| | | | | |
|---|---|---|---|---|
| 255 | Halvorson, Holly W.; , Cohen, Stuart J.; , Brekke, Karen L.; , McClatchey, Maureen W.;  and Cohen, Max M. | 1993 | Process evaluation of a system (Partners for Prevention) for prevention-oriented primary care | A |
| 256 | Hammarberg, K.; , Hassard, J.; , de Silva, R.;  and Johnson, L. | 2020 | Acceptability of screening for pregnancy intention in general practice: a population survey of people of reproductive age | A |
| 257 | Han, J. T.; Park, I. S.; Kang, S. B.; Seo, B. G. | 2018 | Developing the high-risk drinking scorecard model in Korea | A |
| 258 | Han, M. A.; , Choi, K. S.; , Lee, H. Y.; , Kim, Y.; , Jun, J. K.; and Park, E. C. | 2011 | Current status of thyroid cancer screening in Korea: results from a nationwide interview survey | A |
| 259 | Han, M.; Son, K.; Lee, H.; Kwon, H.; Cho, B. | 2014 | Pilot study of information communication technology based weight loss program in workplace | A |
| 260 | Hand, J.; Evans, C.; Blake, H.; Hussain, B.; Rowlands, D. | 2017 | Innovative HIV testing in the workplace | A |
| 261 | Hanprathet, N.; Lertmaharit, S.; Lohsoonthorn, V.; Rattananupong, T.; Ammaranond, P.; Jiamjarasrangsi, W. | 2019 | Shift Work and Leukocyte Count Changes among Workers in Bangkok | A |
| 262 | Hao, L.; , Wang, Z. Z.; , Wang, Y.; , Wang, J.;  and Zeng, Z. P. | 2020 | Association between Cardiorespiratory Fitness, Relative Grip Strength with Non-Alcoholic Fatty Liver Disease | A |
| 263 | Hardweir, V.; Jones, M.; Bonner, S.; Patel, H.; Aderogba, K. | 2019 | Annual viral load monitoring in virologically suppressed patients | A |
| 264 | Hargreaves, S.; Seedat, F.; Car, J.; Escombe, R.; Hasan, S.; Eliahoo, J.; Friedland, J. S. | 2014 | Screening for latent TB, HIV, and hepatitis B/C in new migrants in a high prevalence area of London, UK: A cross-sectional study | A |
| 265 | Harris, M. F.; , Fanaian, M.; , Jayasinghe, U. W.; , Passey, M. E.; , McKenzie, S. H.; , Davies, G. P.; , Lyle, D. M.; , Laws, R. A.; , Schutze, H.;  and Wan, Q. | 2012 | A cluster randomised controlled trial of vascular risk factor management in general practice | A |
| 266 | Harshbarger, C.; , Burrus, O.; , Zulkiewicz, B. A.; , Ortiz, A. M.; , Galindo, C. A.; , Garner, B. R.; , Furberg, R. D.;  and Lewis, M. A. | 2019 | Implementing Web-Based Interventions in HIV Primary Care Clinics: Pilot Implementation Evaluation of Positive Health Check | A |
| 267 | Haruyama, Y.; Fukuda, H.; Arai, T.; Muto, T. | 2013 | Change in lifestyle through health promotion program without face-to-face intervention in a large-scale Japanese enterprise | A |
| 268 | Hasegawa, M.; , Akter, S.; , Hu, H. H.; , Kashino, I.; , Kuwahara, K.; , Okazaki, H.; , Sasaki, N.; , Ogasawara, T.; , Eguchi, M.; , Kochi, T.; , Miyamoto, T.; , Nakagawa, T.; , Honda, T.; , Yamamoto, S.; , Murakami, T.; , Shimizu, M.; , Uehara, A.; , Yamamoto, M.; , Imai, T.; , Nishihara, A.; , Tomita, K.; , Nagahama, S.; , Hori, A.; , Konishi, M.; , Kabe, I.; , Mizoue, T.; , Kunugita, N.; , Dohi, S.;  and Japan Epidemiology, Collaboration | 2020 | Five-year cumulative incidence of overweight and obesity, and longitudinal change in body mass index in Japanese workers: The Japan Epidemiology Collaboration on Occupational Health Study | A |
| 269 | Hasegawa, T.; Asakura, M.; Asanuma, H.; Amaki, M.; Takahama, H.; Sugano, Y.; Kanzaki, H.; Yasuda, S.; Anzai, T.; Izumi, C.; Kitakaze, M. | 2020 | Difference in the prevalence of subclinical left ventricular impairment among left ventricular geometric pattern in a community-based population | A |

| | | | | |
|---|---|---|---|---|
| 270 | Hassoun, D.; and Périn, I. | 2006 | [Ambulatory medical abortion performed in a family planning center] | A |
| 271 | Haug, Kjell; , Aarø, Leif E.; and Fugelli, Per | 1992 | Smoking habits in early pregnancy and attitudes towards smoking cessation among pregnant women and their partners | A |
| 272 | Hawkins, A.; McKay, J.; Hedley, J.; Church, D. B.; Brodbelt, D. C.; O'Neil, D. G. | 2017 | Demography and veterinary care of reptiles attending primary-care veterinary practices in England | A |
| 273 | He, P.; Su, W.; Ma, J. X.; He, D. K.; Li, Q. H.; Ding, W.; Huang, X. H.; Chen, W. H. | 2016 | HSP70 gene polymorphism on genetic susceptibility to the coal worker's penumoconiosis of han nationality in Xinjiang, China | A |
| 274 | Hedderson, M. M.; Darbinian, J.; Havel, P. J.; Quesenberry, C. P.; Sridhar, S.; Ehrlich, S.; Ferrara, A. | 2013 | Low prepregnancy adiponectin concentrations are associated with a marked increase in risk for development of gestational diabetes mellitus | A |
| 275 | Hedderson, M. M.; Xu, F.; Darbinian, J. A.; Quesenberry, C. P.; Sridhar, S.; Kim, C.; Gunderson, E. P.; Ferrara, A. | 2014 | Prepregnancy SHBG concentrations and risk for subsequently developing gestational diabetes mellitus | A |
| 276 | Heianza, Y.; , Hara, S.; , Arase, Y.; , Saito, K.; , Fujiwara, K.; , Tsuji, H.; , Kodama, S.; , Hsieh, S. D.; , Mori, Y.; , Shimano, H.; , Yamada, N.; , Kosaka, K.; and Sone, H. | 2011 | HbA1c 5·7-6·4% and impaired fasting plasma glucose for diagnosis of prediabetes and risk of progression to diabetes in Japan (TOPICS 3): a longitudinal cohort study | A |
| 277 | Heida, A.; , Dijkstra, A.; , Groen, H.; , Kobold, A. M.; , Verkade, H.; and van Rheenen, P. | 2015 | Comparing the efficacy of a web-assisted calprotectin-based treatment algorithm (IBD-live) with usual practices in teenagers with inflammatory bowel disease: study protocol for a randomized controlled trial | A |
| 278 | Hellstrom, M.; Hellstrom, L.; Joep Perk, J. | 2012 | Resting heart rate: A cardiovascular risk predictor for healthy middle aged men | A |
| 279 | Henderson, Angela Lafaye | 2001 | The role of parental pressures and interviewing techniques on children's eyewitness reports | A |
| 280 | Henderson, L. J.; , Smolders, T. V.; and Roughan, J. V. | 2020 | Identifying obstacles preventing the uptake of tunnel handling methods for laboratory mice: An international thematic survey | A |
| 281 | Hengel, B.; , Guy, R.; , Garton, L.; , Ward, J.; , Rumbold, A.; , Taylor-Thomson, D.; , Silver, B.; , McGregor, S.; , Dyda, A.; , Knox, J.; , Kaldor, J.; , Maher, L.; and Investigators, Strive | 2015 | Barriers and facilitators of sexually transmissible infection testing in remote Australian Aboriginal communities: results from the Sexually Transmitted Infections in Remote Communities, Improved and Enhanced Primary Health Care (STRIVE) Study | A |
| 282 | Hergens, M. P.; Lambe, M.; Pershagen, G.; Terent, A.; Ye, W. | 2008 | Smokeless tobacco and the risk of stroke | A |
| 283 | Hergils, L.; and Hergils, A. | 2000 | Universal neonatal hearing screening--parental attitudes and concern | A |
| 284 | Hermansson, U.; Knutsson, A.; Rönnberg, S.; Brandt, L. | 1998 | Feasibility of brief intermention in the workplace for the detection and treatment of excessive alcohol consumption | A |
| 285 | Hernandez, Teresa Marie | 2018 | A quantitative study of the utilization of interim assessment in Colorado turnaround schools | A |
| 286 | Herr, M.; Nikasinovic, L.; Foucault, C.; Le Marec, A. M.; Giordanella, J. P.; Just, J.; Momas, I. | 2012 | Management of wheezing disorders in infants participating in the PARIS birth cohort | A |

| | | | | |
|---|---|---|---|---|
| 287 | Herrero, M. B.; Ramos, S.; Arrossi, S. | 2015 | Determinants of non adherence to tuberculosis treatment in Argentina: barriers related to access to treatment | A |
| 288 | Hewer, Lee-Anne;  and Whyatt, David | 2006 | Improving the implementation of an early literacy program by child health nurses through addressing local training and cultural needs | A |
| 289 | Hilgenkamp, T.; Evenhuis, H. | 2016 | Involving professional caregivers in promoting physical activity: Determinants and strategies | A |
| 290 | Hiramine, Y.; Imamura, Y.; Uto, H.; Koriyama, C.; Horiuchi, M.; Oketani, M.; Hosoyamada, K.; Kusano, K.; Ido, A.; Tsubouchi, H. | 2010 | Alcohol drinking patterns and the risk of fatty liver in Japanese men | A |
| 291 | Hirayama, A.; Konta, T.; Kamei, K.; Suzuki, K.; Ichikawa, K.; Fujimoto, S.; Iseki, K.; Moriyama, T.; Yamagata, K.; Tsuruya, K.; Kimura, K.; Narita, I.; Kondo, M.; Asahi, K.; Kurahashi, I.; Ohashi, Y.; Watanabe, T. | 2015 | Blood Pressure, Proteinuria, and Renal Function Decline: Associations in a Large Community-Based Population | A |
| 292 | Hirose, H.; , Takayama, T.; , Hozawa, S.; , Hibi, T.;  and Saito, I. | 2011 | Prediction of metabolic syndrome using artificial neural network system based on clinical data including insulin resistance index and serum adiponectin | A |
| 293 | Hirose, H.; Takayama, T.; Hozawa, S.; Saito, I. | 2011 | Artificial neural network system predicts 6-year incidence of metabolic syndrome using serum markers for atherosclerosis | A |
| 294 | Hjarnoe, L.; Leppin, A. | 2013 | Health promotion in the Danish maritime setting: challenges and possibilities for changing lifestyle behavior and health among seafarers | A |
| 295 | Ho, S. S.; , Choi, K. C.; , Wong, C. L.; , Chan, C. W.; , Chan, H. Y.; , Tang, W. P.; , Lam, W. W.; , Shiu, A. T.; , Goggins, W. B.;  and So, W. K. | 2014 | Uptake of breast screening and associated factors among Hong Kong women aged ≥50 years: a population-based survey | A |
| 296 | Hoj, K.; , Skriver, M. V.; , Maindal, H. T.; , Christensen, B.;  and Sandbaek, A. | 2017 | High prevalence of poor fitness among Danish adults, especially among those with high cardiovascular mortality risk | A |
| 297 | Høj, K.; Vinther Skriver, M.; Terkildsen Maindal, H.; Christensen, B.; Sandbæk, A. | 2017 | High prevalence of poor fitness among Danish adults, especially among those with high cardiovascular mortality risk | A |
| 298 | Hollenstein, Y.; Elzi, L.; Hatz, C.; Battegay, M.; Stöckle, M.; Halter, J.; Egli, A. | 2014 | Travelling after allogeneic stem cell transplantation-a single centre experience | A |
| 299 | Holzmann, M. J.; , Carlsson, A. C.; , Hammar, N.; , Ivert, T.; , Walldius, G.; , Jungner, I.; , Wandell, P.;  and Arnlov, J. | 2016 | Chronic kidney disease and 10-year risk of cardiovascular death | A |
| 300 | Homer, Stephanie | 2014 | Improving health and access to health services through community-based rehabilitation | A |
| 301 | Honda, A.; Tanabe, N.; Seki, N.; Ogawa, Y.; Suzuki, H. | 2014 | Underweight/overweight and the risk of long-term care: Follow-up study using data of the Japanese long-term care insurance system | A |
| 302 | Honda, C.; Adachi, K.; Arima, N.; Tanaka, S.; Yagi, J.; Morita, T.; Tanimura, T.; Furuta, K.; Kinoshita, Y. | 2008 | Helicobacter pylori infection does not accelerate the age-related progression of arteriosclerosis: A 4-year follow-up study | A |

| | | | | |
|---|---|---|---|---|
| 303 | Honda, K.; Seike, M.; Saito, T.; Iwao, M.; Tokoro, M.; Arakawa, M.; Endo, M.; Murakami, K. | 2019 | Long-term changes in body composition after interferon-free therapy for hepatitis c | A |
| 304 | Honda, Y.; Watanabe, T.; Takahashi, T.; Kinoshita, D.; Yokoyama, M.; Kadowaki, S.; Narumi, T.; Nishiyama, S.; Takahashi, H.; Arimoto, T.; Shishido, T.; Miyamoto, T.; Kubota, I. | 2014 | Anemia plays a role in pre-clinical myocardial damage and cardiovascular mortality in general population | A |
| 305 | Honda, Y.; Watanabe, T.; Shibata, Y.; Otaki, Y.; Kadowaki, S.; Narumi, T.; Takahashi, T.; Kinoshita, D.; Yokoyama, M.; Nishiyama, S.; Takahashi, H.; Arimoto, T.; Shishido, T.; Inoue, S.; Miyamoto, T.; Konta, T.; Kawasaki, R.; Daimon, M.; Kato, T.; Ueno, Y.; Kayama, T.; Kubota, I. | 2017 | Impact of restrictive lung disorder on cardiovascular mortality in a general population: The Yamagata (Takahata) study | A |
| 306 | Honda, Y.; Watanabe, T.; Tamura, H.; Nishiyama, S.; Takahashi, H.; Arimoto, T.; Shishido, T.; Miyamoto, T.; Shibata, Y.; Konta, T.; Kayama, T.; Kubota, I. | 2018 | Presence of Myocardial Damage Predicts Future Development of Hypertension in a Normotensive Japanese General Population: The Yamagata (Takahata) Study | A |
| 307 | Horibe, Y.; Ueda, T.; Watanabe, Y.; Motokawa, K.; Edahiro, A.; Hirano, H.; Shirobe, M.; Ogami, K.; Kawai, H.; Obuchi, S.; Kim, H.; Sakurai, K. | 2018 | A 2-year longitudinal study of the relationship between masticatory function and progression to frailty or pre-frailty among community-dwelling Japanese aged 65 and older | A |
| 308 | Horst, C.; Ruparel, M.; Quaife, S.; Ahmed, A.; Taylor, M.; Bhowmik, A.; Burke, S.; Shaw, P.; McEwen, A.; Waller, J.; Baldwin, D. R.; Navani, N.; Thakrar, R.; Janes, S. M. | 2016 | The prevalence of undiagnosed COPD on spirometry and emphysema on low-dose ct scans in a lung cancer screening demonstration pilot: A teachable moment? | A |
| 309 | Hsieh, C. J.; Weng, P. H.; Chen, J. H.; Chen, T. F.; Sun, Y.; Wen, L. L.; Yip, P. K.; Chu, Y. M.; Chen, Y. C. | 2015 | Sequence variants of the aging gene CISD2 and the risk for Alzheimer's disease | A |
| 310 | Hu, B.; Shi, X.; Du, X.; Xu, M.; Wang, Q.; Zhao, H. | 2020 | Pattern of immune infiltration in lung cancer and its clinical implication | A |
| 311 | Huang, Y.; Wang, T.; Du, X.; Jian, J.; Kang, J.; Chen, F.; Zhu, A. | 2014 | The reference value of non esterified fatty acids determined by enzymatic method in healthy population | A |
| 312 | Hui, B. B.; Reulin, C.; Guy, R. J.; Donovan, B.; Hocking, J. S.; Law, M. G.; Regan, D. G. | 2017 | Prevalence of trichomonas vaginalis predicted to increase substantially in australia due to replacement of pap smears with HPV testing for cervical screening: A modelling study | A |
| 313 | Hulsegge, G.; , van Mechelen, W.; , Proper, K. I.; , Paagman, H.; and Anema, J. R. | 2020 | Shift work, and burnout and distress among 7798 blue-collar workers | A |
| 314 | Hulsegge, G.; van Mechelen, W.; Paagman, H.; Proper, K. I.; Anema, J. R. | 2020 | The moderating role of lifestyle, age, and years working in shifts in the relationship between shift work and being overweight | A |
| 315 | Hulsegge, G.; van Mechelen, W.; Proper, K. I.; Paagman, H.; Anema, J. R. | 2020 | Shift work, and burnout and distress among 7798 blue-collar workers | A |
| 316 | Humphries, S. E.; Neil, H. A. W. | 2010 | Developing and applying clinically useful approaches to identify individuals with familial hypercholesterolemia in the UK | A |

| | | | | |
|---|---|---|---|---|
| 317 | Hung, S.; Mount, J.; Svarstad, B. | 2010 | Examining perceptions of task performance among pharmacists and technicians providing hypertension management services in thirteen community pharmacies | A |
| 318 | Husdal, R.; Rosenblad, A.; Leksell, J.; Eliasson, B.; Jansson, S.; Jerdén, L.; Stålhammar, J.; Steen, L.; Wallman, T.; Adolfsson, E. T. | 2017 | Resource allocation and organisational features in Swedish primary diabetes care: Changes from 2006 to 2013 | A |
| 319 | Hwang, H. J.; Kim, S. H. | 2015 | The association among three aspects of physical fitness and metabolic syndrome in a Korean elderly population | A |
| 320 | Ibanga, O.; Otabor-Olubor, E. | 2019 | Application of geographical information system in stroke risk hotspots mapping in Uhunmwonde local government area, Edo state, Nigeria | A |
| 321 | Ichino, N.; Osakabe, K.; Sugimoto, K.; Suzuki, K.; Yamada, H.; Takai, H.; Sugiyama, H.; Yukitake, J.; Inoue, T.; Ohashi, K.; Hata, T.; Hamajima, N.; Nishikawa, T.; Hashimoto, S.; Kawabe, N.; Yoshioka, K. | 2015 | The NAFLD Index: A Simple and Accurate Screening Tool for the Prediction of Non-Alcoholic Fatty Liver Disease | A |
| 322 | Ide, M. | 2006 | Cancer screening with FDG-PET | A |
| 323 | Igase, M.; , Okada, Y.; , Ochi, M.; , Igase, K.; , Ochi, H.; , Okuyama, S.; , Furukawa, Y.;  and Ohyagi, Y. | 2018 | Auraptene in the Peels of Citrus Kawachiensis (Kawachibankan) Contributes to the Preservation of Cognitive Function: A Randomized, Placebo-Controlled, Double-Blind Study in Healthy Volunteers | A |
| 324 | Ikeda, K.; Hanashiro, S.; Takazawa, T.; Kawase, Y.; Sawada, M.; Yanagihashi, M.; Ishikawa, Y.; Miura, K.; Hirayama, T.; Kano, O.; Kawabe, K.; Iwasaki, Y. | 2015 | The prevalence and the clinical profile of primary exercise headache on Japanese physical check-up | A |
| 325 | Ikemiyagi, H.; Ishida, A.; Kinjo, K.; Ohya, Y. | 2020 | A high normal ankle--brachial index is associated with electrocardiography-determined left ventricular hypertrophy: the Okinawa Peripheral Arterial Disease Study (OPADS) | A |
| 326 | Imaeda, N.; Goto, C.; Tokudome, Y.; Hirose, K.; Tajima, K.; Tokudome, S. | 2007 | Reproducibility of a short food frequency questionnaire for Japanese general population | A |
| 327 | Imai, E.; Tsubota-Utsugi, M.; Satoh, M.; Kikuya, M.; Asayama, K.; Inoue, R.; Murakami, T.; Metoki, H.; Imai, Y.; Ohkubo, T. | 2017 | High-protein dietary pattern increased a risk of impaired glucose tolerance: The Ohasama study | A |
| 328 | Imamura, Y.; Uto, H.; Oketani, M.; Hiramine, Y.; Hosoyamada, K.; Sho, Y.; Hiwaki, T.; Baba, Y.; Tahara, K.; Kubozono, O.; Kusano, K.; Tsubouchi, H. | 2008 | Association between changes in body composition and the increasing prevalence of fatty liver in Japanese men | A |
| 329 | Ingole, J. R.; , Patel, R. D.; , Ingole, S. J.;  and Pandave, H. T. | 2015 | Opportunistic Screening of Vitamin B12 Deficiency in IT Professionals Presenting for Routine Health Check-up | A |
| 330 | Inoue, H.; Tsukuya, G.; Crawford, B.; Fukuyama, S.; Samukawa, T.; Kiyohara, Y.; Nakanishi, Y.; Nakamura, T.; Ichinose, M.; Matsumoto, K. | 2013 | Validation of the COPD population screener (COPD-PS) and establishment of diagnostic cut-points in Japan | A |

| | | | | |
|---|---|---|---|---|
| 331 | Inoue, I.; Mukoubayashi, C.; Yoshimura, N.; Watanabe, M.; Moribata, K.; Shingaki, N.; Deguchi, H.; Ueda, K.; Enomoto, S.; Maekita, T.; Iguchi, M.; Tamai, H.; Yanaoka, K.; Arii, K.; Fujishiro, M.; Oka, M.; Mohara, O.; Kato, J.; Ichinose, M. | 2011 | Elevated risk of colorectal adenoma with helicobacter pylori-related chronic gastritis: A population-based case-control study | A |
| 332 | Inoue, K.; , Kashima, S.; , Ohara, C.; , Matsumoto, M.; and Akimoto, K. | 2012 | Concordance of two diabetes diagnostic criteria using fasting plasma glucose and hemoglobin A1c: the Yuport Medical Checkup Centre study | A |
| 333 | Irer, B. | 2018 | Socioeconomic Predictors and Patient Perspectives of Prostate-specific Antigen Testing | A |
| 334 | Isaka, Y.; Moriyama, T.; Kanda, K. | 2017 | The SONG (Salt intake and OrigiN from general foods) study - A large-scale survey of the eating habits and dietary salt intake in the working-age population | A |
| 335 | Iseki, K. | 2013 | Nephrology for the people: Presidential Address at the 42nd regional meeting of the Japanese society of nephrology in Okinawa 2012 | A |
| 336 | Iseki, K.; , Konta, T.; , Asahi, K.; , Yamagata, K.; , Fujimoto, S.; , Tsuruya, K.; , Narita, I.; , Kasahara, M.; , Shibagaki, Y.; , Moriyama, T.; , Kondo, M.; , Iseki, C.; , Watanabe, T.; and Design Comprehensive Hlth Care, Sys | 2018 | Association of dipstick hematuria with all-cause mortality in the general population: results from the specific health check and guidance program in Japan | A |
| 337 | Iseki, K.; Asahi, K.; Yamagata, K.; Fujimoto, S.; Tsuruya, K.; Narita, I.; Konta, T.; Kasahara, M.; Shibagaki, Y.; Yoshida, H.; Moriyama, T.; Kondo, M.; Iseki, C.; Watanabe, T. | 2017 | Mortality risk among screened subjects of the specific health check and guidance program in Japan 2008–2012 | A |
| 338 | Iseki, K.; Konta, T.; Asahi, K.; Yamagata, K.; Fujimoto, S.; Tsuruya, K.; Narita, I.; Kasahara, M.; Shibagaki, Y.; Moriyama, T.; Kondo, M.; Iseki, C.; Watanabe, T. | 2017 | Association of dipstick proteinuria with all cause mortality in general population: Results from the specific health check and guidance program in Japan | A |
| 339 | Iseki, K.; Konta, T.; Asahi, K.; Yamagata, K.; Fujimoto, S.; Tsuruya, K.; Narita, I.; Kasahara, M.; Shibagaki, Y.; Moriyama, T.; Kondo, M.; Iseki, C.; Watanabe, T. | 2018 | Glucosuria and all-cause mortality among general screening participants | A |
| 340 | Iseki, K.; Konta, T.; Asahi, K.; Yamagata, K.; Fujimoto, S.; Tsuruya, K.; Narita, I.; Kasahara, M.; Shibagaki, Y.; Moriyama, T.; Kondo, M.; Iseki, C.; Watanabe, T. | 2018 | Association of dipstick hematuria with all-cause mortality in the general population: Results from the specific health check and guidance program in Japan | A |
| 341 | Iseki, K.; Konta, T.; Asahi, K.; Yamagata, K.; Fujimoto, S.; Tsuruya, K.; Narita, I.; Kasahara, M.; Shibagaki, Y.; Moriyama, T.; Kondo, M.; Iseki, C.; Watanabe, T. | 2020 | Impact of Metabolic Syndrome on the Mortality Rate among Participants in a Specific Health Check and Guidance Program in Japan | A |
| 342 | Ishikawa, H.; Teramoto, S.; Tachi, H.; Hizawa, N. | 2014 | Unrecognized mild COPD is a significant risk factor for lung cancer | A |
| 343 | Ishimaru, M.; Kubota, K. | 2017 | Attitude and perceptions of self-management and health monitoring of community-dwelling older-adults participating in residential activities in japan | A |

| | | | | |
|---|---|---|---|---|
| 344 | Ito, K. | 2014 | [Screening for prostate cancer: present status and future perspectives] | A |
| 345 | Ito, Y.; Kurata, M.; Suzuki, K.; Hamajima, N.; Hishida, H.; Aoki, K. | 2006 | Cardiovascular disease mortality and serum carotenoid levels: A Japanese population-based follow-up study | A |
| 346 | Ito, Y.; Suzuki, K.; Ishii, J.; Hishida, H.; Tamakoshi, A.; Hamajima, N.; Aoki, K. | 2006 | A population-based follow-up study on mortality from cancer or cardiovascular disease and serum carotenoids, retinol and tocopherols in Japanese inhabitants | A |
| 347 | Iwasawa, S.; Kikuchi, Y.; Nishiwaki, Y.; Nakano, M.; Michikawa, T.; Tsuboi, T.; Tanaka, S.; Uemura, T.; Ishigami, A.; Nakashima, H.; Takebayashi, T.; Adachi, M.; Morikawa, A.; Maruyama, K.; Kudo, S.; Uchiyama, I.; Omae, K. | 2009 | Effects of SO2 on respiratory system of adult Miyakejima resident 2 years after returning to the island | A |
| 348 | Iwelunmor, J.; Blackstone, S.; Nwaozuru, U.; Conserve, D.; Iwelunmor, P.; Ehiri, J. E. | 2018 | Sexual and reproductive health priorities of adolescent girls in Lagos, Nigeria: Findings from free-listing interviews | A |
| 349 | Jacobs, E.; , Tamayo, M.; , Rosenbauer, J.; , Schulze, M. B.; , Kuss, O.; and Rathmann, W. | 2018 | Protocol of a cluster randomized trial to investigate the impact of a type 2 diabetes risk prediction model on change in physical activity in primary care | A |
| 350 | Jain, M. | 2016 | Public pre-schooling and maternal labour force participation in rural India | A |
| 351 | Jakes, A.; Ewens, M. | 2014 | Reducing the risk of re-infection: Are women in Yorkshire changing their sexual behaviour after chlamydia? | A |
| 352 | Jan, C. F.; Chien, Y. C.; Huang, K. C.; Lin, M. H.; Chen, C. J. | 2013 | Young adults with family history of hepatitis B carriers had higher risk to get natural infection of hepatitis B | A |
| 353 | Jan, C. F.; Chien, Y. C.; Huang, K. C.; Lin, M. H.; Chen, C. J. | 2013 | The serostatus of hepatitis B after 50 months followup among 313 hepatitis B seronegative young adults born after neonatal hepatitis B vaccination era | A |
| 354 | Jang, E. S.; Jeong, S. H.; Hwang, S. H.; Kim, H. Y.; Ahn, S. Y.; Lee, J.; Lee, S. H.; Park, Y. S.; Hwang, J. H.; Kim, J. W.; Kim, N.; Lee, D. H. | 2012 | Effects of coffee, smoking, and alcohol on liver function tests: A comprehensive cross-sectional study | A |
| 355 | Janibekyan, Z. | 2012 | Breast cancer early detection in Armenia | A |
| 356 | Jeannot, E.; Mahler, P.; Duperrex, O.; Chastonay, P. | 2010 | Evolution of overweight and obesity among 5-6-year-old schoolchildren in Geneva | A |
| 357 | Jeannot, E.; Mahler, P.; Duperrex, O.; Wyler, C. A. | 2010 | Prevalence of overweight and obesity in 5 to 6 year old school children in the canton of Geneva | A |
| 358 | Jennings, C. S.; Jones, J.; Mead, A.; Connolly, S.; Kotseva, K.; Holden, A.; Fiumicelli, G.; Stevenson, J.; Hunjan, M.; Turner, E.; Wood, D. A. | 2010 | Lifestyle and health related quality of life changes in coronary and high CVD risk patients and their partners attending the MYACTION integrated vascular prevention programme in the community | A |

| | | | | |
|---|---|---|---|---|
| 359 | Jeon, S. W.; Chang, Y.; Lim, S. W.; Cho, J.; Kim, H. N.; Kim, K. B.; Kim, J.; Kim, Y. H.; Shin, D. W.; Oh, K. S.; Shin, Y. C.; Ryu, S. | 2020 | Bidirectional association between blood pressure and depressive symptoms in young and middle-age adults: A cohort study | A |
| 360 | Jeong, K. D.;  and Kim, J. S. | 2016 | Association between Metabolic Syndrome and Retinal Vascular Changes in Koreans based on Health Check-ups | A |
| 361 | Jeong, S. M.; Choi, S.; Kim, K.; Kim, S. M.; Lee, G.; Park, S. Y.; Kim, Y. Y.; Son, J. S.; Yun, J. M.; Park, S. M. | 2018 | Effect of change in total cholesterol levels on cardiovascular disease among young adults | A |
| 362 | Jhon, M.; Kim, S. Y.; Kim, S. W. | 2019 | P.383 Factors related to physical health monitoring in community-dwelling patients with schizophrenia spectrum disorder | A |
| 363 | Jin, Seok Won; , Lee, Jongwook;  and Yun Lee, Hee | 2018 | Analyzing factors associated with decisional stage of adopting breast cancer screening among korean american women using precaution adoption process model | A |
| 364 | Jin, S. W.; Lee, J.; Lee, S. | 2019 | Analyzing Factors Associated With Decisional Stage of Adoption for Colorectal Cancer Screening Among Older Korean Americans Using Precaution Adoption Process Model | A |
| 365 | Jin, S. W.; Lee, H. Y.; Lee, J. | 2019 | Analyzing Factors of Breast Cancer Screening Adherence among Korean American Women Using Andersen's Behavioral Model of Healthcare Services Utilization | A |
| 366 | Jobayer, M.; , Chowdhury, S. S.; , Shamsuzzaman, S. M.;  and Islam, M. S. | 2016 | Prevalence of Hepatitis B Virus, Hepatitis C Virus, and HIV in Overseas Job Seekers of Bangladesh with the Possible Routes of Transmission | A |
| 367 | Johannes Scholl, J.; Bots, M. L.; Peters, S. A. E. | 2014 | The contribution of cardiorespiratory fitness relative to traditional risk factors on subclinical atherosclerosis | A |
| 368 | Johnson, N.; , Lancaster, T.; , Fuller, A.;  and Hodgson, S. V. | 1995 | THE PREVALENCE OF A FAMILY HISTORY OF CANCER IN GENERAL-PRACTICE | A |
| 369 | Johnson, R. M.; Smith, P.; Strauss, E. J.; Higgins, A.; Jensen, D. R.; Weiss, B. D. | 2008 | Breast cancer screening in an adult literacy program | A |
| 370 | Jon, D.; Hong, N.; Bahk, W.; Yoon, B.; Shin, Y.; Min, K.; Lee, S.; Jung, M. | 2015 | The validity of the modified Korean version of the mood disorder questionnaire | A |
| 371 | Jones, J.; Lumsden, N.; Simons, K.; Fernando, S.; Neil, C.; Manski-Mankervis, J. A.; Hamblin, P.; Janus, E.; Nelson, C. | 2019 | Detection and management of chronic kidney disease and diabetes with e-technology based intervention: Analysis of the chronic disease early detection and improved management in primary care project (CD IMPACT] | A |
| 372 | Jones, M.; Ridout, J.; Wignall, A.; Asumah, M.; Gable, D. | 2013 | Homeless persons can access and engage with specialist diabetes services: Experience from an inner city community based case management proactive engagement approach | A |
| 373 | Joshi, B. N.; , Chauhan, S. L.; , Donde, U. M.; , Tryambake, V. H.; , Gaikwad, N. S.;  and Bhadoria, V. | 2006 | Reproductive health problems and help seeking behavior among adolescents in urban India | A |

| | | | | |
|---|---|---|---|---|
| 374 | Jung, J. Y.; Park, S. K.; Choi, J. M.; Hong, H. P.; Choi, Y. J.; Ryoo, J. H. | 2017 | Evaluation of risk for metabolic syndrome according to the fasting insulin concentration in Korean men | A |
| 375 | Kabasawa, K.; Tanaka, J.; Nakamura, K.; Ito, Y.; Yoshida, K.; Takachi, R.; Sawada, N.; Tsugane, S.; Narita, I. | 2020 | Study Design and Baseline Profiles of Participants in the Uonuma CKD Cohort Study in Niigata, Japan | A |
| 376 | Kabeya, Y.; Kato, K.; Tomita, M.; Katsuki, T.; Oikawa, Y.; Shimada, A. | 2016 | Higher Body Mass Index and Increased Prevalence of Paranasal Sinus Disease | A |
| 377 | Kadoh, K.; Fujikawa, J. | 2010 | Correlation between EEG alpha wave frequency and intima-media thickness of the carotid artery, aortic pulse wave velocity, brain MRI findings | A |
| 378 | Kadowaki, T.; , Watanabe, M.; , Okayama, A.; , Hishida, K.; , Okamura, T.; , Miyamatsu, N.; , Hayakawa, T.; , Kita, Y.;  and Ueshima, H. | 2006 | Continuation of smoking cessation and following weight change after intervention in a healthy population with high smoking prevalence | A |
| 379 | Kailasanathan, A. N. | 2012 | The prevalence of non-alcoholic fatty liver in patients attending master health check up | A |
| 380 | Kakuta, E.; Yamashita, N.; Katsube, T.; Kushiyama, Y.; Suetsugu, H.; Furuta, K.; Kinoshita, Y. | 2011 | Abdominal symptom-related QOL in individuals visiting an outpatient clinic and those attending an annual health check | A |
| 381 | Kalampakorn, S.; , Kaewpan, W.;  and Luksamijarulkul, P. | 2009 | Health responsibilities of an aging Thai male workforce | A |
| 382 | Kameda, T.; Kumamaru, H.; Nishimura, S.; Kohsaka, S.; Miyata, H. | 2020 | Use of oral antidiabetic drugs in Japanese working-age patients with type 2 diabetes mellitus: dosing pattern for metformin initiators | A |
| 383 | Kamei, K.; Araumi, A.; Watanabe, S.; Sato, H.; Ichikawa, K.; Konta, T.; Kubota, I.; Iseki, K.; Yamagata, K.; Fujimoto, S.; Tsuruya, K.; Narita, I.; Moriyama, T.; Yugo, S.; Asahi, K.; Kondo, M.; Kasahara, M.; Watanabe, T. | 2016 | The association between serum uric acid and incidence of non-fatal stroke in the CKD population: A longitudinal survey of a nationwide cohort in Japan | A |
| 384 | Kamei, K.; Konta, T.; Hirayama, A.; Ichikawa, K.; Kubota, I.; Fujimoto, S.; Iseki, K.; Moriyama, T.; Yamagata, K.; Tsuruya, K.; Narita, I.; Kondo, M.; Shibagaki, Y.; Kasahara, M.; Asahi, K.; Watanabe, T. | 2017 | Associations between serum uric acid levels and the incidence of nonfatal stroke: a nationwide community-based cohort study | A |
| 385 | Kamei, K.; Kon, S.; Ichikawa, K.; Konta, T.; Kubota, I.; Fujimoto, S.; Iseki, K.; Moriyama, T.; Yamagata, K.; Tsuruya, K.; Narita, I.; Kondo, M.; Kasahara, M.; Shibagaki, Y.; Asahi, K.; Watanabe, T. | 2017 | The association between serum uric acid and mortality in the CKD population: A longitudinal survey of a nationwide cohort in Japan | A |
| 386 | Kaneko, M.; Ohnishi, I.; Bessho, M.; Ohashi, S.; Tobita, K.; Nakamura, K. | 2011 | Prediction of proximal femur strength by a quantitative computed tomography-based finite element method-creation of predicted strength data of the proximal femur according to age range in a normal population and analysis of risk factors for hip fracture | A |
| 387 | Kang, K.; Kim, J.; Ha, H.; Kim, J.; Choi, Y.; Joo, E.; Kim, M. | 2017 | Effect of age on the association between subjective sleep quality and metabolic syndrome | A |

| | | | | |
|---|---|---|---|---|
| 388 | Kannan, S.; Jaipalreddy, C.; Annapandian, V. M.; Murali Mohan, B. V.; Damodar, S.; Khadilkar, K. S.; Shivaprasad, K. S. | 2019 | Impact of anemia and red cell indices on the diagnosis of pre-diabetes and diabetes in Indian adult population: Is there a cut-off guide for clinicians? | A |
| 389 | Karathana, M.; , Krackhardt, B.; , Schade, M.; and Heudorf, U. | 2018 | [School Entrance Examination for Lateral Entrants - What Can and What Should They be Able to Do? A Discussion Contribution Based on the Data from the Health Authority Frankfurt am Main 2006-2016] | A |
| 390 | Karefylakis, C.; Näslund, I.; Edholm, D.; Sundbom, M.; Karlsson, F. A.; Rask, E. | 2014 | Prevalence of Anemia and Related Deficiencies 10 Years After Gastric Bypass—a Retrospective Study | A |
| 391 | Karlsmose, B.; Lauritzen, T.; Engberg, M.; Parving, A. | 2001 | A randomised controlled trial of screening for adult hearing loss during preventive health checks | A |
| 392 | Kashiwagi, K.; Inoue, N.; Yoshida, T.; Bessho, R.; Yoneno, K.; Imaeda, H.; Ogata, H.; Kanai, T.; Sugino, Y.; Iwao, Y. | 2017 | The impact of visceral adipose tissue as best predictor for difficult colonoscopy and the clinical utility of a long small-caliber scope as rescue | A |
| 393 | Kaso, M.; Takahashi, Y.; Nakayama, T. | 2019 | Factors related to cervical cancer screening among women of childrearing age: a cross-sectional study of a nationally representative sample in Japan | A |
| 394 | Kaur, R.; Mishra, P.; Taneja, D. K. | 2012 | Cardiovascular health promotion in schools of Delhi, India: A baseline evaluation of environment and policies | A |
| 395 | Kawada, A.; Hosaka, H.; Shimoyama, Y.; Kawamura, O.; Kuribayashi, S.; Moki, F.; Kusano, M. | 2013 | Correlation between the esophagogastric junction (EGJ) status and upper gi symptoms identified using a modified frequency scale for gerd (MFSSG) questionnaire, and usefulness of narrow band imaging (NBI) of the EGJ | A |
| 396 | Kawada, T.; Muto, K.; Kanai, T.; Kuratomi, Y. | 2009 | Prostate-specific antigen screening of workers under the age of 40 in Japan | A |
| 397 | Kawano, Y.; Taniai, N.; Yoshioka, M.; Matsushita, A.; Mizuguchi, Y.; Shimizu, T.; Ueda, J.; Yoshida, H.; Uchida, E. | 2012 | A case of resected by laparoscopic procedure with accessory spleen at the liver hilum which was difficult to distinguish to the hepatocellular carcinoma | A |
| 398 | Kelly, D.; Hughes, K.; Bellis, M. A. | 2014 | Work hard, party harder: Drug use and sexual behaviour in young british casual workers in Ibiza, Spain | A |
| 399 | Kelly, J. L.; Elkin, S. L.; Fluxman, J.; Polkey, M. I.; Soljak, M. A.; Hopkinson, N. S. | 2013 | Breathlessness and skeletal muscle weakness in patients undergoing lung health screening in primary care | A |
| 400 | Kelsall, H.; Radi, S.; Sim, M. | 2011 | Cardiovascular and diabetes risk factors in workers of Aboriginal and Torres Strait Islander, Maori, or Pacific Islander descent in a large health check program in Victorian workplaces | A |
| 401 | Kemper, H. C.; , Koppes, L. L.; , de Vente, W.; , van Lenthe, F. J.; , van Mechelen, W.; , Twisk, J. W.; and Post, G. B. | 2002 | Effects of health information in youth and young adulthood on risk factors for chronic diseases--20-year study results from the Amsterdam Growth and Health Longitudinal Study | A |

| | | | | |
|---|---|---|---|---|
| 402 | Kempf, K.; Martin, S.; Döhring, C.; Dugi, K.; Von Wolmar, C. W.; Haastert, B.; Schneider, M. | 2013 | Bi employee study I: Role of overweight and obesity on cardiometabolic risk | A |
| 403 | Keng, J. C.; Goriawala, A.; Rashid, S.; Schmocker, S.; Easson, A.; Kennedy, E. | 2018 | Home to stay: An integrated monitoring system using a mobile app to support patients at home following colorectal surgery | A |
| 404 | Khadjesari, Z.; Freemantle, N.; Linke, S.; Hunter, R.; Murray, E. | 2014 | Health on the web: Randomised controlled trial of online screening and brief alcohol intervention delivered in a workplace setting | A |
| 405 | Khadjesari, Z.; Newbury-Birch, D.; Murray, E.; Shenker, D.; Marston, L.; Kaner, E. | 2015 | Online health check for reducing alcohol intake among employees: A feasibility study in six workplaces across england | A |
| 406 | Khan, M. H.; Islam, F.; Chowdhury, N. M. R. A.; Masako, U.; Chowdhury, S. M.; Delem, M. D.; Rahman, A. | 2016 | Maternal and newborn health situation of Rohingya migrants in cox's Bazar, Bangladesh | A |
| 407 | Khan, R. A.; Souru, C.; Vaghese, S.; Yasir, Z.; Khandekar, R. | 2017 | Vision Screening of Ophthalmic Nursing Staff in a Tertiary Eye Care Hospital: Outcomes and ocular healthcare-seeking behaviours | A |
| 408 | Khan, R.; Rehman, R.; Baig, M.; Hussain, M.; Khan, M.; Syed, F. | 2015 | Dimensions of physical wellness among medical students of public and private medical colleges in Pakistan | A |
| 409 | Kim, C. J.; , Oh, K. W.; , Rhee, E. J.; , Kim, K. H.; , Jo, S. K.; , Jung, C. H.; , Won, J. C.; , Park, C. Y.; , Lee, W. Y.; , Park, S. W.; and Kim, S. W. | 2009 | Relationship between body composition and bone mineral density (BMD) in perimenopausal Korean women | A |
| 410 | Kim, D. S.; Chung, H. U.; Kwon, Y. M.; Yu, S. D. | 2011 | Effecting factors to mercury level in school children in the Yeongnam Region, Korea | A |
| 411 | Kim, H.; , Suzuki, T.; , Yoshida, H.; , Yoshida, Y.; , Sugiura, M.; , Iwasa, H.; , Kwon, J.; and Furuna, T. | 2007 | [Characteristics of urban community-dwelling elderly women with multiple symptoms of the geriatric syndrome and related factors] | A |
| 412 | Kim, H.; Lee, K. J.; Lee, S. O.; Kim, S. | 2004 | Cervical cancer screening in Korean American women: findings from focus group interviews | A |
| 413 | Kim, H.; Suzuki, T.; Yoshida, H.; Yoshida, Y.; Sugiura, M.; Iwasa, H.; Kwon, J.; Furuna, T. | 2007 | Characteristics of urban community-dwelling elderly women with multiple symptoms of the geriatric syndrome and related factors | A |
| 414 | Kim, I. J.; Hye, S. B.; Bohyun, K.; Jin, K. M.; Kim, S. S.; Jeon, Y. K.; Ho, K. J.; Lee, M. | 2018 | Comparison of adiposity indices for the prediction of fatty liver disease in metabolically healthy obese and metabolically unhealthy obese men | A |
| 415 | Kim, J. M.; Kim, M. H.; Ju, Y. S.; Hwang, S. S.; Ha, M.; Kim, B. K.; Zoh, K. E.; Paek, D. | 2018 | Reanalysis of epidemiological investigation of cancer risk among people residing near nuclear power plants in South Korea | A |
| 416 | Kim, J. R.; Kim, W. J.; Kim, S. M.; Oh, M. Y.; Kim, S. J.; Pak, K.; Kim, H.; Jeon, Y. K.; Kim, S. S.; Kim, B. H.; Kim, I. J. | 2013 | Incremental diagnostic value of metabolic tumor volume measured by f-18 FDG PET/CT additive to suvmax for characterization of thyroid FDG incidentaloma; After stratification of serum TSH level | A |
| 417 | Kim, J. W.; Ahn, S. T.; Oh, M. M.; Moon, D. G.; Han, K.; Park, H. S. | 2019 | Incidence of Prostate Cancer according to Metabolic Health Status: a Nationwide Cohort Study | A |
| 418 | Kim, J. W.; Cheon, J.; Park, H. S. | 2018 | Association between bladder cancer and metabolic health status: Analysis of a nationwide database | A |

| | | | | |
|---|---|---|---|---|
| 419 | Kim, K. E.; Lee, Y. J.; Shin, Y. H.; Shin, H. J. | 2015 | Correlation between C-reactive protein levels and affecting factors for adiposity in apparently healthy Korean adolescents | A |
| 420 | Kim, M. C.; Kim, K. H.; Jang, J. S.; Kwon, H. C.; Kim, B. G.; Rattner, D. W. | 2012 | Patient perception of natural orifice transluminal endoscopic surgery in an endoscopy screening program in Korea | A |
| 421 | Kim, N.; , Lee, S. W.; , Choi, S. I.; , Park, C. G.; , Yang, C. H.; , Kim, H. S.; , Rew, J. S.; , Moon, J. S.; , Kim, S.; , Park, S. H.; , Jung, H. C.; , Chung, I. S.; and Gerd Study, Grp | 2008 | The prevalence of and risk factors for erosive oesophagitis and non-erosive reflux disease: a nationwide multicentre prospective study in Korea | A |
| 422 | Kim, S. H.; Yun, J. M.; Chang, C. B.; Piao, H.; Yu, S. J.; Shin, D. W. | 2016 | Prevalence of upper gastrointestinal bleeding risk factors among the general population and osteoarthritis patients | A |
| 423 | Kim, S. K.; , Bae, J. C.; , Baek, J. H.; , Hur, K. Y.; , Lee, M. K.; and Kim, J. H. | 2018 | Is decreased lung function associated with chronic kidney disease? A retrospective cohort study in Korea | A |
| 424 | Kim, S.; Park, S. K.; Ryoo, J. H.; Choi, J. M.; Hong, H. P.; Park, J. H.; Suh, Y. J.; Byoun, Y. S. | 2015 | Incidental risk for diabetes according to serum ferritin concentration in Korean men | A |
| 425 | Kim, T.; Song, B.; Lee, H.; Min, Y.; Min, B.; Lee, J.; Rhee, P.; Kim, J. | 2017 | Association between Helicobacter pylori infection and various markers of systemic inflammation in asymptomatic healthy adults | A |
| 426 | Kim, Y. G.; Han, K. D.; Choi, J. I.; Boo, K. Y.; Kim, D. Y.; Lee, K. N.; Shim, J.; Kim, J. S.; Kim, Y. H. | 2020 | Frequent drinking is a more important risk factor for new-onset atrial fibrillation than binge drinking: A nationwide population-based study | A |
| 427 | Kimura, M.; , Taketani, T.; and Kurozawa, Y. | 2019 | High incidence of status epilepticus and ongoing seizures on arrival to the hospital due to high prevalence of febrile seizures in Izumo, Japan: A questionnaire -based study | A |
| 428 | Kimura, M.; Taketani, T.; Kurozawa, Y. | 2018 | Parental questionnaire study showed that annular ligament displacement was common in three-year-old children and almost a half had reoccurring episodes | A |
| 429 | Kimura, M.; Taketani, T.; Kurozawa, Y. | 2019 | High incidence of status epilepticus and ongoing seizures on arrival to the hospital due to high prevalence of febrile seizures in Izumo, Japan: A questionnaire-based study | A |
| 430 | King, K.; Creighton, S.; Nalabanda, A.; Evans, R. | 2013 | Routine opt-out HIV screening in adult mental health services: Results from pilot intervention to improve screening | A |
| 431 | Kini, A. D.; Venkatsh, D.; Prakash, V. S.; Jaisri, G. | 2011 | Comparison of heat rate variability patterns between normal subjects and patients with coronary artery disease | A |
| 432 | Kini, A. D.; Venkatesh, D.; Prakash, V. S.; Jaisri, G. | 2011 | Study of relationship between age, anthropometric parameters and heart rate variability | A |
| 433 | Kishi, H.; Shibata, Y.; Osaka, D.; Abe, S.; Inoue, S.; Tokairin, Y.; Igarashi, A.; Yamauchi, K.; Kimura, T.; Sato, M.; Aida, Y.; Kubota, I. | 2011 | Fev6 and FEV1/FEV6 in Japanese participants of community-based annual health check: The takahata study | A |
| 434 | Kishida, T.; , Inaba, R.; and Iwata, H. | 1997 | [Relationships between maximal oxygen uptake (VO2max) and physical activity, blood pressure and serum lipids] | A |

| | | | | |
|---|---|---|---|---|
| 435 | Kitamura, T.; Kawamura, T.; Tamakoshi, A.; Wakai, K.; Ando, M.; Ohno, Y. | 2009 | Rationale, design, and profiles of the New Integrated Suburban Seniority Investigation (NISSIN) Project: a study of an age-specific, community-based cohort of Japanese elderly | A |
| 436 | Klein, J.;  and von dem Knesebeck, O. | 2018 | Inequalities in health care utilization among migrants and non-migrants in Germany: a systematic review | A |
| 437 | Klein, J.; Von Dem Knesebeck, O. | 2018 | Inequalities in health care utilization among migrants and non-migrants in Germany: A systematic review 11 Medical and Health Sciences 1117 Public Health and Health Services | A |
| 438 | Knai, C.; Scott, C.; D'Souza, P.; James, L.; Mehrotra, A.; Petticrew, M.; Eastmure, E.; Durand, M. A.; Mays, N. | 2017 | The Public Health Responsibility Deal: making the workplace healthier? | A |
| 439 | Ko, Y. S.; Bae, J. H.; Sinn, D. H.; Gwak, G. Y.; Kang, W.; Paik, Y. H.; Choi, M. S.; Lee, J. H.; Koh, K. C.; Paik, S. W. | 2017 | The Clinical Significance of Serum Alpha-fetoprotein in Diagnosing Hepatocellular Carcinoma in a Health Screening Population | A |
| 440 | Kobayashi, D.; Noto, H.; Shimbo, T.; Ino, T.; Osugi, Y.; Takahashi, O.; Asai, K. | 2019 | Repeated measures of extremely high levels of high-density lipoprotein cholesterol and subsequent all-cause mortality and cardiovascular events: A longitudinal study | A |
| 441 | Kobayashi, K.; Imagama, S.; Ando, K.; Tsushima, M.; Machino, M.; Ota, K.; Tanaka, S.; Morozumi, M.; Kanbara, S.; Ishiguro, N.; Hasegawa, Y. | 2020 | Weakness of grip strength reflects future locomotive syndrome and progression of locomotive risk stage: A 10-year longitudinal cohort study | A |
| 442 | Koga, M.; Saito, H.; Mukai, M.; Kasayama, S.; Yamamoto, T. | 2009 | Factors contributing to increased serum urate in postmenopausal Japanese females | A |
| 443 | Kokubo, Y.; Imano, H.; Hata, J.; Murakoshi, N.; Aizawa, Y.; Iso, H.; Ninomiya, T.; Nakamura, F.; Miyamoto, Y.; Okamura, T.; Tomita, H.; Okumura, K. | 2016 | The development of a risk score for incident atrial fibrillation in Japanese general population cohorts: A collaborative meta-analysis | A |
| 444 | Koller, D.; Mielck, A. | 2009 | Regional and social differences concerning overweight, participation in health check-ups and vaccination. Analysis of data from a whole birth cohort of 6-year old children in a prosperous German city | A |
| 445 | Kolsek, M.;  and Klemenc Ketis, Z. | 2015 | ALCOHOL DRINKING AMONG THE STUDENTS OF THE UNIVERSITY OF MARIBOR, SLOVENIA | A |
| 446 | Komanasin, N.; Phyaluanglath, A.; Settasatian, C.; Settasatian, N.; Mongkolwongroj, P. | 2008 | Platelet glycoprotein IIIa Leu33Pro and Ia C807T/G873A polymorphisms and coronary artery disease in laos | A |
| 447 | Kompor, P.; Muang Karn, R.; Norkaew, J.; Kujapun, J.; Photipim, M.; Ponphimai, S.; Chavengkun, W.; Phong Paew, S.; Kaewpitoon, S.; Rujirakul, R.; Wakhuwathapong, P.; Phatisena, T.; Eaksanti, T.; Joosiri, A.; Polsripradistdist, P.; Padchasuwan, N.; Kaewpitoon, N. | 2016 | Population-Based Intervention for Liver Fluke Prevention and Control in Meuang Yang District, Nakhon Ratchasima Province, Thailand | A |
| 448 | Kon, S.; Konta, T.; Ichikawa, K.; Asahi, K.; Yamagata, K.; Fujimoto, S.; Tsuruya, K.; Narita, I.; Kasahara, M.; Shibagaki, Y.; Iseki, K.; Moriyama, T.; Kondo, M.; Watanabe, T. | 2017 | Association between renal function and cardiovascular and all-cause mortality in the community-based elderly population: results from the Specific Health Check and Guidance Program in Japan | A |

| | | | | |
|---|---|---|---|---|
| 449 | Kondo, N.; Kawachi, I.; Hirai, H.; Kondo, K.; Subramanian, S. V.; Hanibuchi, T.; Yamagata, Z. | 2009 | Relative deprivation and incident functional disability among older Japanese women and men: Prospective cohort study | A |
| 450 | Konno, S.; Munakata, M. | 2014 | Skill underutilization is associated with higher prevalence of hypertension: the Watari study | A |
| 451 | Konno, S.; Munakata, M. | 2015 | Moderately increased albuminuria is an independent risk factor of cardiovascular events in the general Japanese population under 75 years of age: The Watari study | A |
| 452 | Konno, S.; Munakata, M. | 2016 | Relationship between estimated glomerular filtration rate and incident microalbuminuria in the non-diabetic, non-hypertensive Japanese general population | A |
| 453 | Konta, T.; Ikeda, A.; Ichikawa, K.; Fujimoto, S.; Iseki, K.; Moriyama, T.; Yamagata, K.; Tsuruya, K.; Yoshida, H.; Asahi, K.; Kurahashi, I.; Ohashi, Y.; Watanabe, T. | 2012 | Blood pressure control in a japanese population with chronic kidney disease: A baseline survey of a nationwide cohort | A |
| 454 | Kotaki, K.; , Ikeda, H.; , Fukuda, T.; , Yuki, F.; , Hasuo, K.; , Kawano, Y.; and Kawasaki, M. | 2017 | Effectiveness of diagnostic screening tests in mass screening for COPD using a cooperative regional system in a region with heavy air pollution: a cross-sectional study | A |
| 455 | Koto, R.; Nakajima, A.; Horiuchi, H.; Yamanaka, H. | 2019 | Prevalence, patient characteristics, and treatment of gout and asymptomatic hyperuricemia in Japan: Cross-sectional study of a health insurance claims database | A |
| 456 | Koto, R.; Nakajima, A.; Horiuchi, H.; Yamanaka, H. | 2020 | Real-world treatment of gout and asymptomatic hyperuricemia: A cross-sectional study of Japanese health insurance claims data | A |
| 457 | Kotoura, Y.; Morihara, T.; Kida, Y.; Furukawa, R.; Tachiiri, H.; Kubo, T. | 2017 | Prevalence and characteristics of osteochondritis dissecans of the humeral capitellum among young baseball players | A |
| 458 | Krike, P.; Polaka, I.; Rudule, A.; Santare, D.; Park, J.; Murillo, R.; Herrero, R.; Leja, M. | 2015 | Participation rate in the gistar pilot study: The results from a single recruitment centre | A |
| 459 | Krikke, G. G.; Grooten, I. J.; Vrijkotte, T. G. M.; Van Eijsden, M.; Roseboom, T. J.; Painter, R. C. | 2016 | Vitamin B12 and folate status in early pregnancy and cardiometabolic risk factors in the offspring at age 5-6 years: Findings from the ABCD multi-ethnic birth cohort | A |
| 460 | Kulkarni, R. R. | 2014 | Morbidity pattern among elderly in urban field practice area at Ashok Nagar, Belgaum, Karnataka: A community based cross-sectional study | A |
| 461 | Kumagai, N.; Morita, I.; Nakagaki, H.; Toyama, A.; Kobayashi, M.; Shimozato, M.; Matasuhisa, K.; Watanabe, S.; Watanabe, T. | 2005 | Oral healthiness score for 8020 predicts loss of teeth in village residents | A |
| 462 | Kumagai, S.; Nofuji, Y.; Suwa, M.; Yamashita, S.; Kishimoto, H.; Matsuo, E.; Nishichi, R.; Sasaki, H. | 2011 | Serum BDNF predicts the prevalence of dyslipidemia in japanese male adults: A cross sectional study | A |

| | | | | |
|---|---|---|---|---|
| 463 | Kumata, H.; , Nishimura, R.; , Nakanishi, C.; , Inoue, C.; , Tezuka, Y.; , Endo, H.; , Miyagi, S.; , Tominaga, T.; , Unno, M.;  and Kamei, T. | 2018 | Surgical strategy for an adult patient with a catecholamine-producing ganglioneuroblastoma and a cerebral aneurysm: a case report | A |
| 464 | Kummer, Sonja; , Waller, Jo; , Ruparel, Mamta; , Cass, Judith; , Janes, Samuel M.;  and Quaife, Samantha L. | 2020 | Mapping the spectrum of psychological and behavioural responses to low-dose CT lung cancer screening offered within a lung health check | A |
| 465 | Küpper, T.; , Rieke, B.; , Neppach, K.; , Morrison, A.;  and Martin, J. | 2014 | Health hazards and medical treatment of volunteers aged 18-30 years working in international social projects of non-governmental organizations (NGO) | A |
| 466 | Kuriyama, N.; Ihara, M.; Koyama, T.; Mizuno, T.; Ozaki, E.; Kondo, M.; Tokuda, T.; Tamura, A.; Kazuo, T.; Nakagawa, M.; Watanabe, Y. | 2017 | Vasoactive mid-regional pro-adrenomedullin is associated with cognitive decline accompa-nying deep white matter lesions in the brain: A longitudinal brain MRI study | A |
| 467 | Kweon, O. J.; Lee, M. K.; Kim, H. J.; Chung, J. W.; Choi, S. H.; Kim, H. R. | 2016 | Neutropenia and neutrophil-to-lymphocyte ratio in a healthy Korean population: Race and sex should be considered | A |
| 468 | Kwok, C.;  and Fong, D. Y. | 2014 | Breast cancer screening practices among Hong Kong Chinese women | A |
| 469 | Kwok, C.; Endrawes, G.; Lee, C. F. | 2016 | Cultural Beliefs and attitudes about breast cancer and screening practices among Arabic women in Australia | A |
| 470 | Kwok, C.; Pillay, R.; Lee, C. F. | 2016 | Psychometric properties of the breast cancer screening beliefs questionnaire among women of Indian ethnicity living in Australia | A |
| 471 | Kwon, H.; Park, J. H.; Cho, B. | 2017 | Is Vitamin D an independent risk factor of nonalcoholic fatty liver disease? A cross-sectional study of the healthy population | A |
| 472 | Kwon, Y. H.; Kim, S. K.; Cho, J. H.; Kwon, H.; Park, S. E.; Oh, H. G.; Park, C. Y.; Lee, W. Y.; Oh, K. W.; Park, S. W.; Rhee, E. J. | 2018 | The association between persistent hypertriglyceridemia and the risk of diabetes development: The Kangbuk Samsung health study | A |
| 473 | Kyprianou, M.; Kapsou, M.; Raftopoulos, V.; Soteriades, E. S. | 2010 | Knowledge, attitudes and beliefs of Cypriot nurses on the handling of antineoplastic agents | A |
| 474 | La Rosa, E.; , Valensi, P.; , Cohen, R.; , Soufi, K.; , Robache, C.; , Cohen, R.;  and le Clésiau, H. | 2003 | [Socioeconomic determinism of obesity in the Seine-Saint-Denis area] | A |
| 475 | Lam, M. T.; Li, H. W. R.; Wong, C. Y. G.; Yeung, W. S. B.; Ho, P. C.; Ng, E. H. Y. | 2020 | Women's age and total motile normal morphology sperm count predict fecundability: a prospective cohort study | A |
| 476 | Lamsal, D. K. | 2017 | Blood pressure and its association with body mass index among the people attending in a hospital | A |
| 477 | Landecho, M. F.; Alegría-Murillo, L.; López-Fidalgo, J.; Colina, I.; Santesteban, V.; García-Unciti, M.; Beloqui, O.; Frühbeck, G.; Cuervo, M. | 2020 | Unravelling gender-specific factors that link obesity to albuminuria | A |
| 478 | Lang, S. J.; , Abel, G. A.; , Mant, J.;  and Mullis, R. | 2016 | Impact of socioeconomic deprivation on screening for cardiovascular disease risk in a primary prevention population: a cross-sectional study | A |

| | | | | |
|---|---|---|---|---|
| 479 | Langford, Aisha T.; , Sawyer, Devin R.; , Gioimo, Shari; , Brownson, Carol A.; and O'Toole, Mary L. | 2007 | Patient-centered goal setting as a tool to improve diabetes self-management | A |
| 480 | Langlie, J. K. | 1979 | Interrelationships among preventive health behaviors: A test of competing hypotheses | A |
| 481 | Larsen, I. K.; , Grotmol, T.; , Almendingen, K.; and Hoff, G. | 2006 | Lifestyle characteristics among participants in a Norwegian colorectal cancer screening trial | A |
| 482 | Larsen, L. B.; , Sondergaard, J.; , Thomsen, J. L.; , Halling, A.; , Sonderlund, A. L.; , Christensen, J. R.; and Thilsing, T. | 2019 | Step-wise approach to prevention of chronic diseases in the Danish primary care sector with the use of a personal digital health profile and targeted follow-up - an assessment of attendance | A |
| 483 | Latz, Isabel K. | 2020 | Associations between perceptions of U.S. immigration enforcement policies, physical health, psychological distress, and health care utilization in a Hispanic border community | A |
| 484 | Lazzarotto, S.; Baumstarck, K.; Loundou, A.; Hamidou, Z.; Aghababian, V.; Lero, T.; Auquier, P. | 2016 | Age-related hearing loss in individuals and their caregivers: Effects of coping on the quality of life among the dyads | A |
| 485 | Leber, W.; McMullen, H.; Bremner, S.; Kerry, S.; Millett, D.; Mguni, S.; Awosika, D.; Creighton, S.; Figueroa, J.; Hart, G.; Sampson, M.; Anderson, J.; Griffiths, C. | 2012 | Can point of care HIV testing in primary care increase identification of HIV? The RHIVA 2 cluster randomised controlled trial - Update | A |
| 486 | Lebrett, M. B.; Balata, H.; Evison, M.; Colligan, D.; Duerden, R.; Elton, P.; Greaves, M.; Howells, J.; Irion, K.; Karunaratne, D.; Lyons, J.; Mellor, S.; Myerscough, A.; Newton, T.; Sharman, A.; Smith, E.; Taylor, B.; Taylor, S.; Walsham, A.; Whittaker, J.; Barber, P. V.; Tonge, J.; Robbins, H. A.; Booton, R.; Crosbie, P. A. J. | 2020 | Analysis of lung cancer risk model (PLCO M2012 and LLP v2) performance in a community-based lung cancer screening programme | A |
| 487 | Ledson, M. J.; Grundy, S.; Arvanitis, R.; Timoney, M.; Gaynor, E.; Field, J. | 2017 | The liverpool healthy lung project (LHLP)-seeking out lung disease | A |
| 488 | Lee, B.; Lee, S. W.; Kang, H. R.; Kim, D. I.; Sun, H. Y.; Kim, J. H. | 2018 | Relationship between lower urinary tract symptoms and cardiovascular risk scores including Framingham risk score and ACC/AHA risk score | A |
| 489 | Lee, D. Y.; Lee, E. S.; Kim, J. H.; Park, S. E.; Park, C. Y.; Oh, K. W.; Park, S. W.; Rhee, E. J.; Lee, W. Y. | 2016 | Predictive value of triglyceride glucose index for the risk of incident diabetes: A 4-year retrospective longitudinal study | A |
| 490 | Lee, G. R.; , Griffin, A.; , Halton, K.; and Fitzgibbon, M. C. | 2017 | Generating method-specific Reference Ranges - A harmonious outcome? | A |
| 491 | Lee, H. Y.; Moon, J. E.; Sun, H. Y.; Doo, S. W.; Yang, W. J.; Song, Y. S.; Lee, S. R.; Park, B. W.; Kim, J. H. | 2019 | Association between lower urinary tract symptoms and cardiovascular risk scores in ostensibly healthy women | A |
| 492 | Lee, J. K.; Choi, H. G.; Kim, J. Y.; Nam, J.; Kang, H. T.; Koh, S. B.; Oh, S. S. | 2016 | Self-resilience as a protective factor against development of post-traumatic stress disorder symptoms in police officers | A |
| 493 | Lee, J.; Lee, J. H.; Jung, S. M.; Suh, Y. S.; Koh, J. H.; Lee, J. Y.; Min, H. K.; Kwok, S. K.; Ju, J. H.; Park, K. S.; Park, S. H. | 2014 | Visceral fat obesity in patients with primary gout in Korea | A |

| | | | | |
|---|---|---|---|---|
| 494 | Lee, J.; Lee, J. Y.; Lee, J. H.; Jung, S. M.; Suh, Y. S.; Koh, J. H.; Kwok, S. K.; Ju, J. H.; Park, K. S.; Park, S. H. | 2015 | Visceral fat obesity is highly associated with primary gout in a metabolically obese but normal weighted population: A case control study | A |
| 495 | Lee, J.; Smith, J. P. | 2012 | The effect of health promotion on diagnosis and management of diabetes | A |
| 496 | Lee, M. Y.; Cho, Y. K.; Bang, K. B.; Lee, D. S.; Yu, J. H.; Lee, H. A.; Park, E. H.; Sohn, C. I. | 2014 | The incidence rate of alcoholic fatty liver related to alcohol consumption: A 4-year retrospective cohort study | A |
| 497 | Lee, R. W.; Nair, A.; Stacey, C.; Fitzgerald, D.; Quaife, S.; Sasieni, P.; Janes, S.; Baldwin, D. | 2019 | Developing NHS England's national targeted lung health check pilot | A |
| 498 | Lee, S. M.; , Leem, J.; , Park, J. H.; , Yoon, K. H.; , Woo, J. S.; , Lee, J. M.; , Kim, J. B.; , Kim, W.; and Lee, S. | 2017 | Close look at the experiences of patients enrolled in a clinical trial of acupuncture treatment for atrial fibrillation in Korea: a qualitative study nested within a randomised controlled trial | A |
| 499 | Lee, Y. C.; , Chien, K. L.; and Chen, H. H. | 2007 | Lifestyle risk factors associated with fatigue in graduate students | A |
| 500 | Lehto, R.; , Mäki, P.; , Ray, C.; , Laatikainen, T.; and Roos, E. | 2016 | Childcare use and overweight in Finland: Cross-sectional and retrospective associations among 3- and 5-year-old children | A |
| 501 | Leppik, B. | 2013 | Annual walking day-move towards a healthier lifestyle | A |
| 502 | Lesný, P.; Krásnicanová, H.; Vejvalka, J. | 2005 | Web based software for growth diagnostics | A |
| 503 | Lester, H.; Reilly, S.; Planner, C.; Hann, M. | 2010 | The primary care experience of people with serious mental illness in England | A |
| 504 | Lewis, M. A.; Harshbarger, C.; Bann, C.; Burrus, O.; Peinado, S.; Garner, B. R.; Khavjou, O.; Shrestha, R. K.; Karns, S.; Borkowf, C. B.; Zulkiewicz, B. A.; Ortiz, A.; Galindo, C. A.; DallaPiazza, M.; Holm, P.; Marconi, V. C.; Somboonwit, C.; Swaminathan, S. | 2020 | Positive Health Check evaluation: A type 1 hybrid design randomized trial to decrease HIV viral loads in patients seen in HIV primary care | A |
| 505 | Li, C.; , Zeng, L.; , Dibley, M. J.; , Wang, D.; , Pei, L.; and Yan, H. | 2015 | Evaluation of socio-economic inequalities in the use of maternal health services in rural western China | A |
| 506 | Li, J.; , Cao, Y. F.; , Sun, X. Y.; , Han, L.; , Li, S. N.; , Gu, W. Q.; , Song, M.; , Jiang, C. T.; , Yang, X. L.; and Fang, Z. Z. | 2019 | Plasma tyrosine and its interaction with low high-density lipoprotein cholesterol and the risk of type 2 diabetes mellitus in Chinese | A |
| 507 | Li, L.; , Wan, C.; and Wen, F. Q. | 2014 | An unexpected role for serum uric acid as a biomarker for severity of asthma exacerbation | A |
| 508 | Li, L.; Wan, C.; Wen, F. | 2014 | An unexpected role for serum uric acid as a biomarker for severity of asthma exacerbation | A |
| 509 | Li, M.; Zhou, H.; Guan, Y.; Peng, H.; Wang, S.; Zhang, P.; Su, B. | 2016 | Positive hepatitis B surface antibody is associated with reduced risk of diabetes mellitus in retired female Chinese workers | A |
| 510 | Li, W. C.; Chen, I. C.; Chang, Y. C.; Loke, S. S.; Wang, S. H.; Hsiao, K. Y. | 2013 | Waist-to-height ratio, waist circumference, and body mass index as indices of cardiometabolic risk among 36,642 Taiwanese adults | A |

| | | | | |
|---|---|---|---|---|
| 511 | Li, Y. M. | 2008 | Concordance of a self assessment tool and measurement of bone mineral density in identifying the risk of osteoporosis in elderly Taiwanese women | A |
| 512 | Li, Y.; Yatsuya, H.; Iso, H.; Tamakoshi, K.; Toyoshima, H. | 2010 | Incidence of metabolic syndrome according to combinations of lifestyle factors among middle-aged Japanese male workers | A |
| 513 | Liaw, S. T.; Wade, V.; Furler, J. S.; Hasan, I.; Lau, P.; Kelaher, M.; Xuan, W.; Harris, M. F. | 2019 | Cultural respect in general practice: a cluster randomised controlled trial | A |
| 514 | Liaw, S. T.; Wade, V.; Furler, J.; Hasan, I.; Lau, P.; Kelaher, M.; Xuan, W.; Harris, M. | 2019 | Cultural respect in general practice: A mixed method cluster randomised controlled trial | A |
| 515 | Lidin, M. | 2015 | A cardio metabolic high risk individual the process and results of participating in a structured lifestyle intervention program for one year | A |
| 516 | Lidin, M.; , Ekblom-Bak, E.; , Karlsson, M. R.;  and Hellenius, M. L. | 2018 | Long-term effects of a Swedish lifestyle intervention programme on lifestyle habits and quality of life in people with increased cardiovascular risk | A |
| 517 | Lidin, M.; , Hellenius, M. L.; , Rydell-Karlsson, M.;  and Ekblom-Bak, E. | 2018 | Long-term effects on cardiovascular risk of a structured multidisciplinary lifestyle program in clinical practice | A |
| 518 | Lidin, M.; Ekblom-Bak, E.; Hellènius, M. | 2012 | Sedentary time decrease in patients with cardiovascular risk factors participating in a structured lifestyle intervention program | A |
| 519 | Lidin, M.; Ekblom-Bak, E.; Rydell Karlsson, M.; Hellénius, M. L. | 2018 | Long-term effects of a Swedish lifestyle intervention programme on lifestyle habits and quality of life in people with increased cardiovascular risk | A |
| 520 | Lidin, M.; Hellenius, M. L. H.; Rydell-Karlsson, M. R. K. | 2018 | Experience from individuals with high cardiovascular risk participating in a structured lifestyle program | A |
| 521 | Liel, C.; Ulrich, S. M.; Lorenz, S.; Eickhorst, A.; Fluke, J.; Walper, S. | 2020 | Risk factors for child abuse, neglect and exposure to intimate partner violence in early childhood: Findings in a representative cross-sectional sample in Germany | A |
| 522 | Liersch, S.; , Kruger, K.; , Oedingen, C.; , Spreenberg, A.; , Bergemann, T.;  and Krauth, C. | 2020 | Evaluation of the pediatric-centered integrated care AOK Junior: protocol for a mixed-method study | A |
| 523 | Lin, Wan-Shiuan; , Lee, Ting-Ting; , Yang, Yuan-Hao;  and Mills, Mary Etta | 2019 | Environmental factors affecting self-management of chronic hepatitis B from the patients' perspective | A |
| 524 | Lippi, G.; Bovo, C.; Buonocore, R.; Mitaritonno, M.; Cervellin, G. | 2017 | Red blood cell distribution width in patients with limb, chest and head trauma | A |
| 525 | Liu, H.; Liu, Y.; Wang, L.; Xu, D.; Lin, B.; Zhong, R.; Gong, S.; Podda, M.; Invernizzi, P. | 2010 | Prevalence of primary biliary cirrhosis in adults referring hospital for annual health check-up in Southern China | A |
| 526 | Liu, H. Y.; , Liu, Y. F.; , Wang, L. X.; , Xu, D. X.; , Lin, B. L.; , Zhong, R. Q.; , Gong, S. T.; , Podda, M.;  and Invernizzi, P. | 2010 | Prevalence of primary biliary cirrhosis in adults referring hospital for annual health check-up in Southern China | A |

| | | | | |
|---|---|---|---|---|
| 527 | Liu, Jinan; , Xu, Lingzhong; , Cao, Xiuling; , Wang, Xingzhou; , Sun, Hui; , Tang, Cheng; , Yang, Zhenghui; , Song, Xiaofei; and Lv, Ming | 2008 | Analysis of satisfaction about new cooperative medical scheme and its influencing factors in Weihai, China | A |
| 528 | Liu, K.; Chen, X.; Wang, S.; Wan, S.; Zhou, Y.; Pan, P.; Wen, B.; Zhang, X.; Liao, H.; Shi, D.; Shi, R. | 2015 | Arterial stiffness, central pulsatile hemodynamic load and orthostatic hypotension: A cross-sectional survey of community residents in chengdu | A |
| 529 | Liu, K.; Xu, J.; Tao, L.; Yang, K.; Sun, Y.; Guo, X. | 2020 | Platelet counts are associated with arterial stiffness in Chinese Han population: a longitudinal study | A |
| 530 | Liu, P. J.; , Ma, F.; , Lou, H. P.; and Liu, Y. P. | 2013 | The utility of fat mass index vs. body mass index and percentage of body fat in the screening of metabolic syndrome | A |
| 531 | Liu, P. J.; Ma, F.; Lou, H. P.; Zhu, Y. N.; Chen, Y. | 2014 | Relationship between serum uric acid levels and hepatic steatosis in non-obese postmenopausal women | A |
| 532 | Liu, P. J.; Ma, F.; Lou, H. P.; Zhu, Y. N.; Chen, Y. | 2014 | Relationship between serum uric acid levels and metabolic syndrome in Chinese postmenopausal women | A |
| 533 | Liu, P.; Ma, F.; Lou, H.; Zhu, Y.; Chen, Y. | 2014 | [Relationship between normal serum uric acid levels and nonalcoholic fatty liver disease in postmenopausal women] | A |
| 534 | Lokesh, S.; , Green, S. R.; , Hemachandar, R.; , Kadavanu, T. M.; , Ramachandrappa, A.; , Tiwari, S. R.; , Rajkumar, A. L.; and Govindasamy, E. | 2016 | Trace Elements in Chronic Haemodialysis Patients and Healthy Individuals-A Comparative Study | A |
| 535 | Lorch, R.; , Hocking, J.; , Temple-Smith, M.; , Law, M.; , Yeung, A.; , Wood, A.; , Vaisey, A.; , Donovan, B.; , Fairley, C. K.; , Kaldor, J.; and Guy, R. | 2013 | The chlamydia knowledge, awareness and testing practices of Australian general practitioners and practice nurses: survey findings from the Australian chlamydia control effectiveness pilot (ACCEPt) | A |
| 536 | Lotrean, L. M.; Ailoaiei, R.; Popa, M.; de Vries, H. | 2015 | Knowledge regarding early detection of cancer among romanian women having relatives with cancer | A |
| 537 | Lovett, R.; Dance, P.; Guthrie, J.; Brown, R.; Tongs, J. | 2014 | Walan Girri: developing a culturally mediated case management model for problematic alcohol use among urban Indigenous people | A |
| 538 | Lowe, C.; , Blinkhorn, A. S.; , Worthington, H. V.; and Craven, R. | 2007 | Testing the effect of including oral health in general health checks for elderly patients in medical practice--a randomized controlled trial | A |
| 539 | Lu, L.; Zou, G.; Zeng, Z.; Han, L.; Guo, Y.; Ling, L. | 2014 | Health-related quality of life and its correlates among chinese migrants in small-and medium-sized enterprises in two cities of guangdong | A |
| 540 | Macfarlane, E.; Laut, S.; Vine, D.; Knock, P.; Obeyesekera, S.; Dhairyawan, R. | 2014 | "My memory is not what it was" pilot of initial screening for cognitive and mood disorders within an HIV clinic | A |
| 541 | Maesato, H.; Itoh, M. | 2018 | The effects of brief intervention at awork place | A |
| 542 | Magnin, M.; Jeannot, E.; Voahangy, R.; Stoll, B. | 2018 | A realistic evaluation approach highlighted the success factors and difficulties of an innovative and comprehensive malnutrition programme in Madagascar | A |

| | | | | |
|---|---|---|---|---|
| 543 | Mäki, P.; Lehtinen-Jacks, S.; Vuorela, N.; Levälahti, E.; Koskela, T.; Saari, A.; Mölläri, K.; Mahkonen, R.; Salo, J.; Laatikainen, T. | 2017 | Register of primary health care visits (avohilmo) as data source for monitoring the prevalence of overweight among Finnish children | A |
| 544 | Makita, S.; , Abiko, A.; , Nagai, M.; , Yonezawa, S.; , Koshiyama, M.; , Ohta, M.; and Nakamura, M. | 2013 | Influence of daily alcohol consumption on serum adiponectin levels in men | A |
| 545 | Makizako, H.; Kubozono, T.; Kiyama, R.; Takenaka, T.; Kuwahata, S.; Tabira, T.; Kanoya, T.; Horinouchi, K.; Shimada, H.; Ohishi, M. | 2019 | Associations of social frailty with loss of muscle mass and muscle weakness among community-dwelling older adults | A |
| 546 | Malhotra, N.; Keshan, M. K.; Agarwal, A.; Kumar, R. A.; Trailokya, A.; Dalvi, K.; Talele, S. | 2016 | DemographIc Assessment and EValuation of DEgree of Lipid Control in High Risk Indian DySlipidemia PatiEnts (DIVERSE Study) | A |
| 547 | Mandai, M.; Kaso, M.; Takahashi, Y.; Nakayama, T. | 2018 | Loneliness among mothers raising children under the age of 3 years and predictors with special reference to the use of SNS: A community-based cross-sectional study | A |
| 548 | Mandic, S.; , Wilson, H.; , Clark-Grill, M.; and O'Neill, D. | 2018 | A Physical Activity Learning Module Improves Medical Students' Skills and Confidence for Advising Patients about Physical Activity | A |
| 549 | Manikam, Logan; , Shah, Rakhee; , Reed, Kate; , Santini, Gupreet; and Lakhanpaul, Monica | 2017 | Using a co-production prioritization exercise involving south asian children, young people and their families to identify health priorities requiring further research and public awareness | A |
| 550 | Mant, D.; , Fuller, A.; , Northover, J.; , Astrop, P.; , Chivers, A.; , Crockett, A.; , Clements, S.; and Lawrence, M. | 1992 | Patient compliance with colorectal cancer screening in general practice | A |
| 551 | Mar, J.; Álvarez-Sabín, J.; Oliva, J.; Becerra, V.; Casado, M.; Yébenes, M.; González-Rojas, N.; Arenillas, J. F.; Martínez-Zabaleta, M. T.; Rebollo, M.; Lago, A.; Segura, T.; Castillo, J.; Gállego, J.; Jiménez-Martínez, C.; López-Gastón, J. I.; Moniche, F.; Casado-Naranjo, I.; López-Fernández, J. C.; González-Rodríguez, C.; Escribano, B.; Masjuan, J. | 2013 | The costs of stroke in Spain by aetiology: The CONOCES study protocol | A |
| 552 | Marcinkiewicz, A.; , Hanke, W.; , Kałużny, P.; , Lipińska-Ojrzanowska, A.; , Wiszniewska, M.; and Walusiak-Skorupa, J. | 2018 | Can Periodical Examinations of Employees Be Useful in Detection of Glycaemia Impairment and Improving Patients' Adherence to Medical Recommendations? | A |
| 553 | Marcinkiewicz, A.; , Wojda, M.; , Walusiak-Skorupa, J.; , Hanke, W.; and Rydzynski, K. | 2017 | ANALYSIS OF TASKS OF OCCUPATIONAL HEALTH SERVICES ACCOMPLISHED IN POLAND, 1997-2014. DO WE EXPLOIT THE FULL POTENTIAL OF PROPHYLACTIC EXAMINATIONS OF WORKERS? | A |
| 554 | Martin, G. | 2003 | Annual health reviews for patients with severe learning disabilities: Five years of a combined GP/CLDN clinic | A |
| 555 | Martins, E.; , Malpeli, A.; , Asens, D.; , Telese, L.; , Fasano, V.; , Vargas, V.; , Tavella, M.; and Lerner, J. E. C. | 2018 | Contribution of diet to lead exposure among children aged 1 to 7 years in La Plata, Buenos Aires | A |

| | | | | |
|---|---|---|---|---|
| 556 | Martins, E.; , Varea, A.; , Hernandez, K.; , Sala, M.; , Girardelli, A.; , Fasano, V.;  and Disalvo, L. | 2016 | Blood lead levels in children aged between 1 and 6 years old in La Plata, Argentina. Identification of risk factors for lead exposure | A |
| 557 | Marwaha, R. K.; Tandon, N.; Ganie, M. A.; Kanwar, R.; Sastry, A.; Garg, M. K.; Bhadra, K.; Singh, S. | 2012 | Status of thyroid function in Indian adults: Two decades after universal salt Iodization | A |
| 558 | Mason, Michael J.; , Schmidt, Christopher; , Abraham, Anisha; , Walker, Leslie;  and Tercyak, Kenneth | 2009 | Adolescents' social environment and depression: Social networks, extracurricular activity, and family relationship influences | A |
| 559 | Matee, M. I.; Simon, E.; Christensen, M. F.; Kirk, K.; Andersen, L.; Samaranayake, L. P.; Scheutz, F. | 1995 | Association between carriage of oral yeasts and malnutrition among Tanzanian infants aged 6-24 months | A |
| 560 | Matsuoka, J.; Kobayashi, Y.; Kajiki, S.; Uehara, M.; Sasaki, N.; Odagami, K.; Hiraoka, K.; Nakanishi, S.; Igarashi, Y.; Mori, K. | 2017 | Developing a checklist for collecting information from overseas hospitals | A |
| 561 | Matthias Lidin, M.; Hellenius, M. L.; Rydell Carlsson, M.; Ekblom-Bak, E. | 2018 | A 1-year structured lifestyle program in clinical practice reduces cardiovascular risk for individuals with high cardiovascular risk | A |
| 562 | McAullay, D.; McAuley, K.; Bailie, R.; Mathews, V.; Jacoby, P.; Gardner, K.; Sibthorpe, B.; Strobel, N.; Edmond, K. | 2018 | Sustained participation in annual continuous quality improvement activities improves quality of care for Aboriginal and Torres Strait Islander children | A |
| 563 | McCarthy, K. J.; Braganza, S.; Fiori, K.; Gbeleou, C.; Kpakpo, V.; Lopez, A.; Schechter, J.; Goodwin, A. S.; Jones, H. E. | 2017 | Identifying inequities in maternal and child health through risk stratification to inform health systems strengthening in Northern Togo | A |
| 564 | McCormick, P. J.; Levin, M. A.; Reich, D. L. | 2013 | Design and implementation of a fully automated prospective randomized effectiveness trial | A |
| 565 | McCulloch, B.; , McDermott, R.; , Miller, G.; , Leonard, D.; , Elwell, M.;  and Muller, R. | 2003 | Self-reported diabetes and health behaviors in remote in idigenous communities in Northern Queensland, Australia | A |
| 566 | McGeown, M.;  and Fitzpatrick, P. | 2017 | Dental Attendance Among Adults at High Risk for Oral Cancer | A |
| 567 | McLean, K.; Little, K.; Hiscock, H.; Scott, D.; Goldfeld, S. | 2019 | Health needs and timeliness of assessment of Victorian children entering out-of-home care: An audit of a multidisciplinary assessment clinic | A |
| 568 | McMenamin, J. P. | 1994 | Screening for alcohol use disorder in a general practice | A |
| 569 | McMenamin, J. P. | 1997 | Detecting young adults with alcohol use disorder in a general practice | A |
| 570 | McMillan, S.; Scott, P.; Ormerod, D.; Jones, R. | 2012 | On the road: Delivering sexual health services to vulnerable populations in hard-to-reach areas | A |
| 571 | McMullen, H.; Griffiths, C.; Leber, W.; Greenhalgh, T. | 2015 | Explaining high and low performers in complex intervention trials: A new model based on diffusion of innovations theory | A |
| 572 | McMullen, H.; Leber, W.; Griffiths, C.; Mguni, S.; Anderson, J.; Millett, D.; Greenhalgh, T.; Figueroa, J. | 2016 | What is the experience of patients testing HIV-positive through rapid testing in general practice? A qualitative study | A |
| 573 | Meena, S.; Leanne, Q.; Scheil, W.; Shearing, T.; Nori, A.; Spurrier, N.; Nottage, C. | 2019 | Prevalence of ear disease and hearing loss in aboriginal children living in metropolitan South Australia | A |

| | | | | |
|---|---|---|---|---|
| 574 | Melosini, L.; Bugliaro, F.; Carli, M.; Novelli, F.; Frateiacci, S.; Paggiaro, P. | 2012 | Disease knowledge, patient's expectations and asthma control in patients with moderate to severe asthma: An Italian survey | A |
| 575 | Mendez, F. J.;  and Gomez-Conesa, A. | 2001 | Postural hygiene program to prevent low back pain | A |
| 576 | Meng, Q. T.; , Wang, S.; , Wang, Y.; , Wan, S. X.; , Liu, K.; , Zhou, X. Y.; , Zhong, G. Y.; , Zhang, X.;  and Chen, X. P. | 2014 | Arterial stiffness is a potential mechanism and promising indicator of orthostatic hypotension in the general population | A |
| 577 | Mesa, M. S. | 2018 | Health Care Disparities Between Men and Women With Type 2 Diabetes | A |
| 578 | Michikawa, T.; Nishiwaki, Y.; Kikuchi, Y.; Nakano, M.; Takamizawa, M.; Koike, M.; Kikuchi, N.; Mukoyami, Y.; Nakazawa, A.; Nishigaki, Y.; Takebayashi, T. | 2008 | A survey on urinary incontinence among middle aged and older people | A |
| 579 | Mikolasch, T.; Ruparel, M.; Dickson, J.; Horst, C.; Tisi, S.; Quaife, S.; Navani, N.; Taylor, M.; Ahmed, A.; Shaw, P.; Burke, S.; Soo, M. J.; Bhowmik, A.; Porter, J.; Janes, S. | 2018 | Interstital lung disease rates and risk factors in a UK lung cancer screening trial | A |
| 580 | Millar, H. L. | 2010 | Prevalence of metabolic syndrome in a UK psychiatric population | A |
| 581 | Miller, C. S.;  and Hart, G. | 1995 | REDUCED DISEASE PREVALENCE AT A SEXUALLY-TRANSMITTED DISEASES CLINIC DURING A MASS-MEDIA CAMPAIGN | A |
| 582 | Millett, D.; Creighton, S. | 2010 | HIV testing as part of NHS health checks: Report from a community testing initiative | A |
| 583 | Milne, N.; Ali, A. | 2015 | Improving quality: The impact of formal impaired glucose regulation reviews in the primary care setting | A |
| 584 | Minnerly, S.; Steece, R. S. | 2014 | The effectiveness of a point of care test for syphilis in local health clinics | A |
| 585 | Mishra, G. A.; Pimple, S.; Singal, A.; Kulkarni, V.; Shaikh, H.; Majmudar, P. | 2019 | Tobacco control and oral cancer screening among public transport bus drivers, conductors and other staff in Mumbai, India | A |
| 586 | Misumi, Y.; Sao, R.; Ishii, M.; Hida, N.; Okamoto, H. | 2010 | An assessment of annual lung cancer screening in yokohama: The 27 years' cumulative data | A |
| 587 | Mitani, A.; , Hakamata, Y.; , Hosoi, M.; , Horie, M.; , Murano, Y.; , Saito, A.; , Yanagimoto, S.; , Tsuji, S.; , Yamamoto, K.; and Nagase, T. | 2017 | The incidence and risk factors of asymptomatic primary spontaneous pneumothorax detected during health check-ups | A |
| 588 | Mitani, S.; Ozasa, K.; Shigeta, M.; Kuriyama, N.; Ozaki, E.; Mizuno, T.; Yokota, S.; Watanabe, Y. | 2010 | A cross-sectional study of familial clustering in hyperhomocysteinemia | A |
| 589 | Miyakita, T.; Ueda, A. | 2000 | Evaluation of hearing disorders and social support among the middle aged and elderly in the community. I. Analysis on the relationship between hearing difficulties and social participation, and self-rated health | A |

| | | | | |
|---|---|---|---|---|
| 590 | Miyashita, Y.; Akaleephan, C.; Asgari-Jirhandeh, N.; Sungyuth, C. | 2017 | Cross-border movement of older patients: A descriptive study on health service use of Japanese retirees in Thailand | A |
| 591 | Mizoo, T.; Taira, N.; Nishiyama, K.; Nogami, T.; Iwamoto, T.; Motoki, T.; Shien, T.; Matuoka, J.; Doihara, H.; Ishihara, S.; Kawai, N.; Kawasaki, K.; Ishibe, Y.; Ogasawara, Y. | 2012 | Effect of lifestyle and single nucleotide polymorphisms on breast cancer risk: A case-control study in Japanese women | A |
| 592 | Mizoue, T.; Kochi, T.; Akter, S.; Eguchi, M.; Kurotani, K.; Tsuruoka, H.; Kuwahara, K.; Ito, R.; Kabe, I.; Nanri, A. | 2015 | Low serum 25-hydroxyvitamin D concentrations are associated with increased likelihood of having depressive symptoms among Japanese workers | A |
| 593 | Mochizuki, K.; Hariya, N.; Miyauchi, R.; Misaki, Y.; Ichikawa, Y.; Goda, T. | 2014 | Self-reported faster eating associated with higher ALT activity in middle-aged, apparently healthy Japanese women | A |
| 594 | Mogere, D. M.; Loum, C. S.; Kaseje, D. | 2015 | Referral recommendations made by Community Health Workers on the - Patient's referral slips. Bridging households to sustainable healthcare; Kenyan Experience | A |
| 595 | Mogi, M.; , Kohara, K.; , Tabara, Y.; , Tsukuda, K.; , Igase, M.; and Horiuchi, M. | 2018 | Correlation between the 24-h urinary angiotensinogen or aldosterone level and muscle mass: Japan shimanami health promoting program study | A |
| 596 | Mogilevkina, I.;  and Odlind, V. | 2003 | Contraceptive practices and intentions of Ukrainian women | A |
| 597 | Moi, H. | 2007 | Handilab C chlamydia for home testing is not what it claims | A |
| 598 | Momo, K.; , Yasu, T.; , Yasui, H.;  and Kuroda, S. | 2019 | Risk factors affecting the failed low-density lipoprotein level achievement rate in working-age male population at high cardiovascular risk | A |
| 599 | Moon, H.; , Ko, H. J.;  and Kim, A. S. | 2019 | The Relationship Between Serum 25-Hydroxyvitamin D Levels and Physical Performance in Community-Dwelling Older Adults | A |
| 600 | Moore, C.; Sanchez, M.; Mueller, D.; Reed, M.; Heithoff, K. | 2013 | A program to assist transitions from hospital to home | A |
| 601 | Morgaine, K. C.; Firth, H. M.; Herbison, G. P.; Feyer, A. M.; McBride, D. I. | 2005 | Obtaining health information from farmers: Interviews versus postal questionnaires in a New Zealand case study | A |
| 602 | Mori, Y.; Komiya, H.; Utsunomiya, K. | 2014 | Relationship between abdominal obesity and manifestation of risk factors for the metabolic syndrome in Japanese: A 5-year cohort study using data from local resident health check-up programs | A |
| 603 | Morita, E.; Naito, M.; Hishida, A.; Wakai, K.; Mori, A.; Asai, Y.; Okada, R.; Kawai, S.; Hamajima, N. | 2011 | No association between the frequency of forest walking and blood pressure levels or the prevalence of hypertension in a cross-sectional study of a Japanese population | A |
| 604 | Morrison, R.; Reynolds, C. A.; Jegasothy, E.; Brailsford, S. R. | 2015 | Deferral of donors due to piercing events - Getting to the point of the matter | A |
| 605 | Mourad, J.; Michault, A.; Le Jeune, S.; Lopez-Sublet, M.; Le Clesiau, H. | 2010 | Evaluation of vascular age and its contributors in the general population | A |

| | | | | |
|---|---|---|---|---|
| 606 | Muller, A.; , Lamoureux, E.; , Bullen, C.; and Keeffe, J. E. | 2006 | Factors associated with regular eye examinations in people with diabetes: Results from the Victorian population health survey | A |
| 607 | Mulshine, J. | 2019 | ES05.05 Still Struggling for Traction — from Proving Lung Cancer Screening Works to Global Practical Implementation, Including Engagement of the Target Population | A |
| 608 | Munns, A.; Watts, R.; Hegney, D.; Walker, R. | 2016 | Effectiveness and experiences of families and support workers participating in peer-led parenting support programs delivered as home visiting programs: A comprehensive systematic review | A |
| 609 | Muraki, I.; Tanigawa, T.; Yamagishi, K.; Umesawa, M.; Hayama-Terada, M.; Kitamura, A.; Ohira, T.; Imano, H.; Cui, R.; Kiyama, M.; Iso, H. | 2018 | Associations of sleep-disordered breathing in relation to incident atrial fibrillation and change in serum nt-probnp levels among japanese: The circulatory risk in communities study (circs) | A |
| 610 | Murashima, M.; , Kikuchi, Y.; , Nomiyama, T.; , Kumagai, N.; , Omae, K.; and Watanabe, S. | 2004 | [Intake and excretion of cadmium and iron balance and influence of dietary habits among young women] | A |
| 611 | Murayama, Y.; Ohba, H.; Yasunaga, M.; Nonaka, K.; Takeuchi, R.; Nishi, M.; Sakuma, N.; Uchida, H.; Shinkai, S.; Fujiwara, Y. | 2015 | The effect of intergenerational programs on the mental health of elderly adults | A |
| 612 | Murdock, R.; Mundowa, G.; Clarke, L.; Roberts, J.; Yannaghas, H.; Raffee, S.; Jenner, J.; Gilleece, Y. | 2018 | Does annual attendance at a specialist service for women living with HIV address both sexual and reproductive health issues? | A |
| 613 | Murlidhar, V.; Kanhere, V. | 2005 | Asbestosis in an asbestos composite mill at Mumbai: A prevalence study | A |
| 614 | Murray, E.; Khadjesari, Z.; Linke, S.; Hunter, R.; Freemantle, N. | 2013 | Health on the web: randomised trial of work-based online screening and brief intervention for hazardous and harmful drinking | A |
| 615 | Nagahama, S.; , Kurotani, K.; , Pham, N. M.; , Nanri, A.; , Kuwahara, K.; , Dan, M.; , Nishiwaki, Y.; and Mizoue, T. | 2014 | Self-reported eating rate and metabolic syndrome in Japanese people: cross-sectional study | A |
| 616 | Nagahama, S.; Kashino, I.; Hu, H.; Nanri, A.; Kurotani, K.; Kuwahara, K.; Dan, M.; Michikawa, T.; Akter, S.; Mizoue, T.; Murakami, Y.; Nishiwaki, Y. | 2018 | Haemoglobin A1c and hearing impairment: longitudinal analysis using a large occupational health check-up data of Japan | A |
| 617 | Nagamatsu, Y.; , Barroga, E.; , Sakyo, Y.; , Igarashi, Y.; and Hirano, Y. O. | 2020 | Risks and perception of non-communicable diseases and health promotion behavior of middle-aged female immigrants in Japan: a qualitative exploratory study | A |
| 618 | Nagata, C.; Shimizu, H.; Takami, R.; Hayashi, M.; Takeda, N.; Yasuda, K. | 1999 | Hot flushes and other menopausal symptoms in relation to soy product intake in Japanese women | A |
| 619 | Nagata, C.; Shimizu, H.; Takami, R.; Hayashi, M.; Takeda, N.; Yasuda, K. | 2003 | Association of blood pressure with intake of soy products and other food groups in Japanese men and women | A |
| 620 | Nagura, A.; Yamada, H.; Ohashi, K.; Ishikawa, H.; Ichino, N.; Taromaru, N.; Ando, Y.; Taromaru, N.; Yamazaki, M.; Watarai, R.; Inoue, T.; Hamajima, N.; Teradaira, R. | 2014 | Circulating microRNAs, miR197, let7d, miR150, miR92, is influenced by smoking status in general population | A |

| | | | | |
|---|---|---|---|---|
| 621 | Nahar-Roy, M.; Mishra, B.; Swami, S.; Sharma, K. K.; Gupta, R. | 2014 | Gender bias in preventive cardiovascular health checks at a tertiary care hospital | A |
| 622 | Nakai, Y.; Makizako, H.; Kiyama, R.; Tomioka, K.; Taniguchi, Y.; Kubozono, T.; Takenaka, T.; Ohishi, M. | 2019 | Association between chronic pain and physical frailty in community-dwelling older adults | A |
| 623 | Nakajima, S.; Komada, Y.; Sasai-Sakuma, T.; Okajima, I.; Harada, Y.; Watanabe, K.; Inoue, Y. | 2017 | Higher sleep reactivity and insomnia mutually aggravate depressive symptoms: a cross-sectional epidemiological study in Japan | A |
| 624 | Nakamura, K.; , Watanabe, Y.; , Kitamura, K.; , Kabasawa, K.; and Someya, T. | 2019 | Psychological distress as a risk factor for dementia after the 2004 Niigata-Chuetsu earthquake in Japan | A |
| 625 | Nakamura, Y.; Oki, I.; Tanihara, S.; Ojima, T.; Kuwano, T.; Tsukada, M.; Momose, M.; Kobayashi, M.; Yanagawa, H. | 1999 | Relationship between breast milk and atopic dermatitis in children | A |
| 626 | Nakamura, Y.; Oki, I.; Tanihara, S.; Ojima, T.; Ito, Y.; Yamazaki, O.; Iwama, M.; Tabata, Y.; Katsuyama, K.; Sasai, Y.; Nakagawa, M.; Matsushita, A.; Hossaka, K.; Sato, J.; Hidaka, Y.; Uda, H.; Nakamata, K.; Yanagawa, H. | 2000 | Relationship between breast milk feeding and atopic dermatitis in children | A |
| 627 | Nakanishi, Noriyuki;  and Tatara, Kozo | 2000 | Correlates and prognosis in relation to participation in social activities among older people living in a community in Osaka, Japan | A |
| 628 | Nakanishi, N.; Hino, Y.; Ida, O.; Fukuda, H.; Shinsho, F.; Tatara, K. | 1999 | Associations between self-assessed masticatory disability and health of community-residing elderly people | A |
| 629 | Nakano, H.; Shibata, Y.; Inoue, S.; Igarashi, A.; Yamauchi, K.; Abe, S.; Sato, M.; Aida, Y.; Nunomiya, K.; Kimura, T.; Nemoto, T.; Watanabe, T.; Konta, T.; Ueno, Y.; Kato, T.; Kayama, T.; Kubota, I. | 2013 | Relationships between values of antibodies to several connective tissue disease autoantigens and pulmonary function in a Japanese general population: The Takahata study | A |
| 630 | Nakano, K.; Ono, K.; Yasumura, S. | 2002 | A survey of infection control among community home care service providers | A |
| 631 | Nakano, M.; Upadhyaya, S.; Chudal, R.; Skokauskas, N.; Luntamo, T.; Sourander, A.; Kaneko, H. | 2019 | Risk factors for impaired maternal bonding when infants are 3 months old: A longitudinal population based study from Japan | A |
| 632 | Nakatoh, S. | 2014 | [Epidemiology of bone and joint disease - the present and future - . The present situation and problems associated with medical screening systems for osteoporosis] | A |
| 633 | Nakazawa, A.; Shigeta, M.; Ozasa, K. | 2004 | Smoking cigarettes of low nicotine yield does not reduce nicotine intake as expected: A study of nicotine dependency in Japanese males | A |
| 634 | Nallely, M. M.; , Esmeralda, V.; , Merari, A. C. M.; , Gisela, G. F. D.; , Josefina, R. V.; , Minarda, D. A.;  and Carlos, R. L. J. | 2014 | IS INGESTION OF THASUS GIGAS (XAMUES) AN ALIMENTARY CULTURE OR AN AUXILIARY TREATMENT FOR TYPE II DIABETES? | A |
| 635 | Nam, H.; Jun, S. | 2015 | Associations between brain global hypometabolism and various clinical parameters in healthy subjects | A |

| | | | | |
|---|---|---|---|---|
| 636 | Nam, H. Y.; Jun, S.; Pak, K.; Kim, I. J. | 2017 | Concurrent low brain and high liver uptake on FDG PET are associated with cardiovascular risk factors | A |
| 637 | Nam, H. Y.; Jun, S. | 2017 | Association between active brown adipose tissue and coronary artery calcification in healthy men | A |
| 638 | Narumi, T.; Shishido, T.; Kiribayashi, N.; Kadowaki, S.; Nishiyama, S.; Takahashi, H.; Arimoto, T.; Miyashita, T.; Miyamoto, T.; Watanabe, T.; Shibata, Y.; Konta, T.; Ueno, Y.; Kato, T.; Kayama, T.; Kubota, I. | 2012 | Impact of insulin resistance on silent and ongoing myocardial damage in normal subjects: The takahata study | A |
| 639 | Narumi, T.; Shishido, T.; Kadowaki, S.; Otaki, Y.; Honda, Y.; Honda, S.; Hasegawa, H.; Nishiyama, S.; Takahashi, H.; Arimoto, T.; Miyashita, T.; Miyamoto, T.; Watanabe, T.; Kubota, I. | 2012 | Insulin resistance is a risk factor for ongoing myocardial damage in general subjects: The takahata study | A |
| 640 | Näsänen-Gilmore, P.; Sipola-Leppänen, M.; Tikanmäki, M.; Matinolli, H. M.; Eriksson, J. G.; Järvelin, M. R. J.; Vääräsmäki, M.; Hovi, P.; Kajantie, E. | 2016 | Late preterm birth protects against allergies in adulthood | A |
| 641 | Näsänen-Gilmore, P.; Sipola-Leppänen, M.; Tikanmäki, M.; Matinolli, H.; Eriksson, J. G.; Järvelin, M.; Vääräsmäki, M.; Hovi, P.; Kajantie, E. | 2017 | Late preterm birth protects against atopies in adulthood | A |
| 642 | Nassaji, M.; Ghorbani, R.; Tamadon, M. R.; Bitaraf, M. | 2015 | Association between body mass index and urinary tract infection in adult patients | A |
| 643 | Nasu, K.; , Nakamura, M.; , Aoki, N.; , Iitoi, Y.; , Kondo, I.; and Kikuchi, Y. | 2003 | [Red blood cell deformability in relation to gender, age, blood pressure, obesity, serum lipids, alcohol consumption and smoking] | A |
| 644 | Nath, D.; Shivashekar, M.; Vinodhini, V. M. | 2019 | Fibrinogen levels in obese and normal individuals | A |
| 645 | Nattabi, B.; Matthews, V.; Bailie, J.; Rumbold, A.; Scrimgeour, D.; Schierhout, G.; Ward, J.; Guy, R.; Kaldor, J.; Thompson, S. C.; Bailie, R. | 2017 | Wide variation in sexually transmitted infection testing and counselling at Aboriginal primary health care centres in Australia: Analysis of longitudinal continuous quality improvement data | A |
| 646 | Naumanen, P. | 2006 | The health promotion model as assessed by ageing workers | A |
| 647 | Nawata, A.; Omori, G.; Koga, Y.; Endoh, K.; Endo, N. | 2018 | Relevance of sarcopenia and knee extensor muscle strength in the matsudai knee osteoarthritis survey | A |
| 648 | Neervoort, F.; von Rosenstiel, I.; Bongers, K.; Demetriades, M.; Shacola, M.; Wolffers, I. | 2013 | Effect of a school feeding programme on nutritional status and anaemia in an urban slum: A preliminary evaluation in Kenya | A |
| 649 | Nelson, J.; King, M.; Scofield, S.; Kirkham, K.; Priestley, C. | 2015 | “In and out”-measuring outcomes for pregnancy prevention in females attending sexual health clinics | A |
| 650 | Nemoto, T.; Shibata, Y.; Osaka, D.; Abe, S.; Inoue, S.; Tokairin, Y.; Igarashi, A.; Yamauchi, K.; Kimura, T.; Kishi, H.; Nishiwaki, M.; Aida, Y.; Nunomiya, K.; Sato, M.; Watanabe, T.; Konta, T.; Kawata, S.; Kato, T.; Kayama, T.; Kubota, I. | 2011 | Impact of cigarette smoking on maximal expiratory flows in a general population: The Takahata study | A |

| | | | | |
|---|---|---|---|---|
| 651 | Nemoto, T.; Shibata, Y.; Sato, K.; Nakano, H.; Sato, M.; Aida, Y.; Nunomiya, K.; Kimura, T.; Yamauchi, K.; Igarashi, A.; Inoue, S.; Abe, S.; Kubota, I. | 2013 | The use of MEF50/MEF25 may over-estimate the presence of small airway disease: The Takahata study | A |
| 652 | Ness, A. R.; Khaw, K. T.; Bingham, S.; Day, N. E. | 1996 | Vitamin C status and undiagnosed angina | A |
| 653 | Neumann, S.; , Webendorfer, S.; , Lang, S.; , Germann, C.; and Oberlinner, C. | 2015 | Diabetes- Screening and Prevention in a Large Company in the chemical Industry | A |
| 654 | Ngo, C. Q.; Manabe, T.; Vu, G. V.; Chu, H. T.; Vu, T. T. T.; Tran, T. T.; Doan, L. T. P.; Takasaki, J.; Kudo, K. | 2019 | Difficulties in tuberculosis infection control in a general hospital of Vietnam: a knowledge, attitude, and practice survey and screening for latent tuberculosis infection among health professionals | A |
| 655 | Nielsen, J.; Hulman, A.; Witte, D. R. | 2018 | Spousal cardiometabolic risk factors and incidence of type 2 diabetes: a prospective analysis from the English Longitudinal Study of Ageing | A |
| 656 | Niessen, M. A. J.; Colkesen, B. E.; Van Pelt, D.; Hoppener, M. R.; Mathijssen, J. J.; Dijkgraaf, M. G. W.; Van Kalken, C.; Kraaijenhagen, R. A. | 2010 | Short term reduction in absenteeism after implementation of a personalized prevention program | A |
| 657 | Nigatu, Y. T.; , Roelen, C. A. M.; , Reijneveld, S. A.; and Bultmann, U. | 2015 | Overweight and Distress Have a Joint Association With Long-Term Sickness Absence Among Dutch Employees | A |
| 658 | Niijima, K.; , Enta, K.; , Hori, H.; , Sashihara, S.; , Mizoue, T.; and Morimoto, Y. | 2007 | The usefulness of sleep apnea syndrome screening using a portable pulse oximeter in the workplace | A |
| 659 | Nikzadian, M.; Dashti, F.; Memar, M. O. | 2016 | Compare the attitude to genetic counseling and obstacles in pregnant women with consanguinity and non-family marriage attending prenatal care clinics Al-Hadi Hospital of Shoushtar in 2015 | A |
| 660 | Nilsson, P. M.; , Klasson, E. B.; and Nyberg, P. | 2001 | Life-style intervention at the worksite--reduction of cardiovascular risk factors in a randomized study | A |
| 661 | Niruba, R.; Dilara, K.; Shubhashini, A. S. | 2011 | Co-relation between Serum triacylglycerol and serum hepatic marker enzymes | A |
| 662 | Nishimura, R.; Sano, H.; Onda, Y.; Tsujino, D.; Ando, K.; Ebara, F.; Matsudaira, T.; Ishikawa, S.; Sakamoto, T.; Tajima, N.; Utsunomiya, K. | 2017 | Population-based cross-sectional study on insulin resistance and insulin-secretory capacity in Japanese school children | A |
| 663 | Nishizawa, H.; Shimomura, I. | 2019 | Population approaches targeting metabolic syndrome focusing on Japanese trials | A |
| 664 | Noda, H.; , Harada, M.; , Yokota, K.; , Umesawa, M.; , Yamagishi, K.; , Cui, R.; , Ikeda, A.; , Chei, C.; , Wakabayashi, Y.; , Inagawa, M.; , Toriumi, S.; , Hirose, K.; , Oshima, M.; , Shiina, Y.; , Tanigawa, T.; , Tanaka, K.; , Shimamoto, T.; and Iso, H. | 2006 | [Individualized health education with sports gym use and dietary advice for overweight and obese persons in a community. Kokuho Health-up model Program in Chikusei-shi (former Kyowa town)] | A |
| 665 | Noh, J. W.; Kim, E. J.; Seo, H. J.; Kim, S. G. | 2016 | Independent association between glycated hemoglobin and arterial stiffness in healthy men | A |

| | | | | |
|---|---|---|---|---|
| 666 | Nomura, S.; Blangiardo, M.; Tsubokura, M.; Ozaki, A.; Morita, T.; Hodgson, S. | 2016 | Postnuclear disaster evacuation and chronic health in adults in Fukushima, Japan: A long-term retrospective analysis | A |
| 667 | Norton, J.; Elford, J.; Sherr, L.; Miller, R.; Johnson, M. A. | 1997 | Repeat HIV testers at a London same-day testing clinic | A |
| 668 | Nunomiya, K.; Abe, S.; Inoue, S.; Yamauchi, K.; Kishi, H.; Aida, Y.; Nemoto, T.; Satoh, M.; Kubota, I.; Shibata, Y. | 2012 | Elevated level of plasma homocysteine predicts the decline of FEV1 in healthy male smokers: The Takahata cohort study | A |
| 669 | Nyberg-Oksanen, E.; Sydenham, V.; Morgan, S.; Georgsen, J.; Jansen, E. | 2015 | Managing donors and donation sessions to improve donor service and session productivity | A |
| 670 | Oakey-Neate, L.; Schrader, G.; Strobel, J.; Bastiampillai, T.; van Kasteren, Y.; Bidargaddi, N. | 2020 | Using algorithms to initiate needs-based interventions for people on antipsychotic medication: implementation protocol | A |
| 671 | Obling, K. H.; , Overgaard, K.; , Juul, L.; and Maindal, H. T. | 2013 | The MILE study: a motivational, individual and locally anchored exercise intervention among 30-49 year-olds with low levels of cardiorespiratory fitness: a randomised controlled study in primary care | A |
| 672 | Ochi, Masayuki; , Tabara, Yasuharu; , Kido, Tomoko; , Uetani, Eri; , Ochi, Namiko; , Igase, Michiya; , Miki, Tetsuro; and Kohara, Katsuhiko | 2010 | Quadriceps sarcopenia and visceral obesity are risk factors for postural instability in the middle-aged to elderly population | A |
| 673 | Oelke, N. D.; Rush, K. L.; Goma, F. M.; Barker, J.; Marck, P.; Pedersen, C. | 2015 | Understanding Perceptions and Practices for Zambian Adults in Western Province at Risk for Hypertension: An Exploratory Descriptive Study | A |
| 674 | Ofili, A. N.; , Asuzu, M. C.; and Okojie, O. H. | 2003 | Knowledge and practice of universal precautions among nurses in central hospital, Benin-City, Edo State, Nigeria | A |
| 675 | Ogunsiji, O. O.; Kwok, C.; Fan, L. C. | 2017 | Breast cancer screening practices of African migrant women in Australia: A descriptive cross-sectional study | A |
| 676 | Oguoma, V. M.; Nwose, E. U.; Bwititi, P. T. | 2014 | Cardiovascular disease risk prevention: Preliminary survey of baseline knowledge, Attitude and practices of a Nigerian rural community | A |
| 677 | Oguro, R.; Kamide, K.; Kokubo, Y.; Shimaoka, I.; Congrains, A.; Horio, T.; Hanada, H.; Ohishi, M.; Katsuya, T.; Okamura, T.; Miyata, T.; Kawano, Y.; Rakugi, H. | 2010 | Association of carotid atherosclerosis with genetic polymorphisms of the klotho gene in patients with hypertension | A |
| 678 | Ohki, K.; Toyokawa, S.; Kumura, F.; Kimura, Y.; Kano, K. | 2001 | The effects of a health promotion program on physical, mental, and dietetic health status in climacteric women | A |
| 679 | Ohmachi, I.; Arima, K.; Abe, Y.; Nishimura, T.; Goto, H.; Aoyagi, K. | 2015 | Factors influencing the preferred place of death in community-dwelling elderly people in Japan | A |
| 680 | Okabayashi, S.; Kawamura, T.; Wakai, K.; Ando, M.; Tsushita, K.; Ohira, H.; Ukawa, S.; Tamakoshi, A. | 2019 | Lifestyle and psychosocial factors and a decline in competence in daily living among Japanese early elderly people: From an age-specified community-based cohort study (NISSIN project) | A |
| 681 | Okasha, M.; , McCarron, P.; , McEwen, J.; and Smith, G. D. | 2001 | Age at menarche: secular trends and association with adult anthropometric measures | A |

| | | | | |
|---|---|---|---|---|
| 682 | Okuda, N.; , Okamura, T.; , Kadowaki, T.; , Tanaka, T.; and Ueshima, H. | 2004 | [Weight-control intervention in overweight subjects at high risk of cardiovascular disease: a trial of a public health practical training program in a medical school] | A |
| 683 | Osaka, D.; Shibata, Y.; Abe, S.; Inoue, S.; Tokairin, Y.; Igarashi, A.; Yamauchi, K.; Kimura, T.; Sato, M.; Kishi, H.; Takabatake, N.; Sata, M.; Watanabe, T.; Konta, T.; Kawata, S.; Kato, T.; Kubota, I. | 2010 | Relationship between habit of cigarette smoking and airflow limitation in healthy Japanese individuals: The Takahata study | A |
| 684 | Oshio, T. | 2018 | Widening disparities in health between educational levels and their determinants in later life: evidence from a nine-year cohort study | A |
| 685 | Ostermann, J.; Njau, B.; Hobbie, A.; Mtuy, T.; Thielman, N. | 2018 | HETEROGENEOUS PREFERENCES FOR HIV/AIDS COUNSELING AND TESTING AMONG TWO HIGH RISK POPULATIONS IN NORTHERN TANZANIA - RESULTS FROM A DISCRETE CHOICE EXPERIMENT | A |
| 686 | Otaki, Y.; , Watanabe, T.; , Konta, T.; , Watanabe, M.; , Fujimoto, S.; , Sato, Y.; , Asahi, K.; , Yamagata, K.; , Tsuruya, K.; , Narita, I.; , Kasahara, M.; , Shibagaki, Y.; , Iseki, K.; , Moriyama, T.; , Kondo, M.; and Watanabe, T. | 2018 | Effect of Hypertension on Aortic Artery Disease-Related Mortality-3.8-Year Nationwide Community-Based Prospective Cohort Study | A |
| 687 | Otaki, Y.; Watanabe, T.; Konta, T.; Watanabe, M. | 2019 | Impact of hyperuricemia on mortality related to aortic diseases | A |
| 688 | Oya, J.; Nakagami, T.; Sasaki, S.; Jimba, S.; Murakami, K.; Kasahara, T.; Wasada, T.; Sekiguchi, H.; Hasegawa, M.; Endo, Y.; Iwamoto, Y. | 2010 | Intake of n-3 polyunsaturated fatty acids and non-alcoholic fatty liver disease: A cross-sectional study in Japanese men and women | A |
| 689 | Ozgursoy Uran, B. N.; Yildirim, Y.; Tokem, Y.; Saritas Yuksel, E.; Arslan, G. | 2016 | Nursing management at crohn's disease: Functional health pattern model | A |
| 690 | Pahk, K.; , Kwon, Y.; , Kim, M. K.; , Park, S.; and Kim, S. | 2020 | Visceral fat metabolic activity evaluated by (18)F-FDG PET/CT is associated with osteoporosis in healthy postmenopausal Korean women | A |
| 691 | Palinkas, A.; , Sandor, J.; , Papp, M.; , Korosi, L.; , Falusi, Z.; , Pal, L.; , Belteczki, Z.; , Rihmer, Z.; and Dome, P. | 2019 | Associations between untreated depression and secondary health care utilization in patients with hypertension and/or diabetes | A |
| 692 | Palladino, R.; , Vamos, E. P.; , Chang, K. C.; , Khunti, K.; , Majeed, A.; and Millett, C. | 2020 | Evaluation of the Diabetes Screening Component of a National Cardiovascular Risk Assessment Programme in England: a Retrospective Cohort Study | A |
| 693 | Palmer, E. L.; Wingfield, D.; Jamrozik, K.; Partridge, M. R. | 2005 | A pilot study to assess the possible methods of determining the burden of obstructive sleep apnoea syndrome in primary care | A |
| 694 | Panaretto, K.; Coutts, J.; Johnson, L.; Morgan, A.; Leon, D.; Hayman, N. | 2010 | Evaluating performance of and organisational capacity to deliver brief interventions in Aboriginal and Torres Strait Islander medical services | A |
| 695 | Park, H. T.; Kim, T.; Park, H. M. | 2015 | Vasomotor symptoms and metabolic syndrome in Korean postmenopausal women | A |

| | | | | |
|---|---|---|---|---|
| 696 | Park, H. W.; Sohn, K. H. | 2017 | Health check-up data showed a possible relationship between serum uric acid level and FEV1/FVC ratio | A |
| 697 | Park, H. Y.; Hahm, C. R.; Jeon, K.; Um, S. W.; Suh, G. Y.; Chung, M. P.; Kim, H.; Kwon, O. J.; Koh, W. J. | 2009 | Solitary pulmonary nodules caused by Mycobacterium tuberculosis and Mycobacterium avium complex | A |
| 698 | Park, J. Y.; , Kim, J.; , Lee, J. H.; , Oh, K. S.; , Chung, S. W.; and Park, H. | 2019 | Does a Partial Rotator Cuff Tear Affect Pitching Ability? Results From an MRI Study | A |
| 699 | Park, S.; Chun, J.; Han, K. D.; Soh, H.; Kang, E. A.; Lee, H. J.; Im, J. P.; Kim, J. S. | 2019 | The risk of inflammatory bowel disease based on body mass index and waist circumference: A nationwide population-based study | A |
| 700 | Park, S.; Greene, M. C.; Melby, M. K.; Fujiwara, T.; Surkan, P. J. | 2019 | Postpartum Depressive Symptoms as a Mediator Between Intimate Partner Violence During Pregnancy and Maternal-Infant Bonding in Japan | A |
| 701 | Park, S. K.; Choi, W. J.; Oh, C. M.; Kim, M. G.; Ham, W. T.; Choi, J. M.; Ryoo, J. H. | 2015 | Clinical significance of serum ferritin level as an independent predictor of insulin resistance in Korean men | A |
| 702 | Park, S. K.; Jung, J. Y.; Choi, W. J.; Kim, Y. H.; Kim, H. S.; Ham, W. T.; Shin, H.; Ryoo, J. H. | 2014 | Elevated fasting serum insulin level predicts future development of hypertension | A |
| 703 | Park, S. M. | 2019 | Effects of work conditions on suicidal ideation among middle-aged adults in South Korea | A |
| 704 | Parker, S. M.; Stocks, N.; Nutbeam, D.; Thomas, L.; Denney-Wilson, E.; Zwar, N.; Karnon, J.; Lloyd, J.; Noakes, M.; Liaw, S. T.; Lau, A.; Osborne, R.; Harris, M. F. | 2018 | Preventing chronic disease in patients with low health literacy using eHealth and teamwork in primary healthcare: Protocol for a cluster randomised controlled trial | A |
| 705 | Patel, N.; Solanki, S.; Patel, P.; Patel, S.; Lakhani, C.; Amarapurkar, D. | 2015 | Hyperhomocysteinemia in non-cirrhotic, insulin resistant and obese patients with non-alcoholic fatty liver disease | A |
| 706 | Patrick, F.; Young, A. H.; Williams, S. C. R.; Perkins, A. M. | 2018 | Prescreening clinical trial volunteers using an online personality questionnaire | A |
| 707 | Pavithra, V.; , Sathisha, T. G.; , Kasturp, K.; , Mallika, D. S.; , Amos, S. J.; and Ragunatha, S. | 2015 | Serum Levels of Metal Ions in Female Patients with Breast Cancer | A |
| 708 | Paz-Zulueta, M.; , Alvarez-Paredes, L.; , Diaz, J. C. R.; , Paras-Bravo, P.; , Becerra, M. E. A.; , Ingelmo, J. M. R.; , Garcia, M. M. R.; , Portilla, J.; and Santibanez, M. | 2018 | Prevalence of high-risk HPV genotypes, categorised by their quadrivalent and nine-valent HPV vaccination coverage, and the genotype association with high-grade lesions | A |
| 709 | Pearsall, R.; Hughes, S.; Geddes, J.; Pelosi, A. | 2014 | Understanding the problems developing a healthy living programme in patients with serious mental illness: A qualitative study | A |
| 710 | Perera, Bhathika; , Audi, Salma; , Solomou, Solomis; , Courtenay, Ken; and Ramsay, Hugh | 2019 | Mental and physical health conditions in people with intellectual disabilities: Comparing local and national data | A |
| 711 | Perez, C. M.; Ball, S. L.; Wagner, A. P.; Clare, I. C. H.; Holland, A. J.; Redley, M. | 2015 | The incidence of healthcare use, ill health and mortality in adults with intellectual disabilities and mealtime support needs | A |
| 712 | Perticaroli, P.; Mengucci, R.; Carletti, M.; Magnini, P.; Marcellini, M.; Pettinari, A.; Durastanti, M. | 2013 | Asbestos-related diseases in former asbestos-cement workers in Senigallia | A |

| | | | | |
|---|---|---|---|---|
| 713 | Petersen, J.; Malyutina, S.; Ryabikov, A.; Kontsevaya, A.; Kudryavtsev, A. V.; Eggen, A. E.; McKee, M.; Cook, S.; Hopstock, L. A.; Schirmer, H.; Leon, D. A. | 2020 | Uncontrolled and apparent treatment resistant hypertension: A cross-sectional study of Russian and Norwegian 40-69 year olds | A |
| 714 | Pfeiffer, W.; Scholl, J.; Renz, E.; Ciré, L.; Kentner, M. | 2001 | How healthy are company leaders? A cross-sectional study of the cardiovascular risk profile of managers | A |
| 715 | Pibernik-Okanovic, M.; Grgurevic, M.; Ajdukovic, D.; Novak, B.; Begic, D.; Metelko, Z. | 2009 | Screening performance of a short versus long version of the patient health questionnaire-depression in outpatients with diabetes | A |
| 716 | Pibernik-Okanovic, M.; Prasek, M.; Poljicanin-Filipovic, T.; Pavlic-Renar, I.; Metelko, Z. | 2004 | Effects of an empowerment-based psychosocial intervention on quality of life and metabolic control in type 2 diabetic patients | A |
| 717 | Pickens, R. C.; Cochran, A.; Tezber, K.; Berry, R.; Bhattacharya, E.; Koo, D.; King, L.; Iannitti, D. A.; Martinie, J. B.; Baker, E. H.; Ocuin, L. M.; Hunt, J.; Vrochides, D. | 2019 | Using a mobile application for real-time collection of patient reported outcomes in hepatopancreatobiliary surgery within an ERAS® pathway | A |
| 718 | Pinnock, C.; , Yip, J. L. Y.; , Khawaja, A. P.; , Luben, R.; , Hayat, S.; , Broadway, D. C.; , Foster, P. J.; , Khaw, K. T.;  and Wareham, N. | 2016 | Topical Beta-Blockers and Cardiovascular Mortality: Systematic Review and Meta-Analysis with Data from the EPIC-Norfolk Cohort Study | A |
| 719 | Pinto, C. G. D.; , Marega, M.; , de Carvalho, J. A. M.; , Carmona, F. G.; , Lopes, C. E. F.; , Ceschini, F. L.; , Bocalini, D. S.;  and Figueira, A. J. | 2015 | Physical activity as a protective factor for development of non-alcoholic fatty liver in men | A |
| 720 | Pinzon, E. M.; , Epidemiol, M.; , Bravo, S. M.; , Epidemiol, M.; , Mendez, F.; , Clavijo, G. M.;  and Leon, M. E. | 2008 | Prevalence and factors related with the presence of oral manifestations in HIV/AIDS patients who attended health institutions in Cali, Colombia | A |
| 721 | Platen, P.;  and Schaar, B. | 2003 | How to carry out a health-oriented marathon training programme for running and inline skating | A |
| 722 | Plenel, E.; Duchesne, L.; Lert, F.; Molina, J. M.; Velter, A. | 2019 | Drop in HIV diagnoses among MSM in Paris: Combined effect of PrEP, testing and political support to the communities | A |
| 723 | Pollett, S.; Calderon, M.; Heitzinger, K.; Solari, V.; Montano, S. M.; Zunt, J. | 2013 | Prevalence and predictors of cervicitis in female sex workers in Peru: An observational study | A |
| 724 | Poudel-Tandukar, K.; Sato, M.; Ejima, Y.; Nanri, A.; Matsushita, Y.; Imaizumi, K.; Mizoue, T. | 2012 | Relationship of serum fatty acid composition and desaturase activity to C-reactive protein in Japanese men and women | A |
| 725 | Priest, S.; , Austin, M. P.; , Barnett, B.;  and Buist, A. | 2008 | A psychosocial risk assessment model (PRAM) for use with pregnant and postpartum women in primary care settings | A |
| 726 | Prost, A.; Griffiths, C. J.; Anderson, J.; Wight, D.; Hart, G. J. | 2009 | Feasibility and acceptability of offering rapid HIV tests to patients registering with primary care in London (UK): A pilot study | A |
| 727 | Provost, D.; , Iwatsubo, Y.; , Riviere, S.; , Mevel, M.; , Didier, A.; , Brochard, P.; , Imbernon, E.;  and Raherison, C. | 2015 | The impact of allergic rhinitis on the management of asthma in a working population | A |
| 728 | Pugh, E.; Pakianathan, M.; McCormick, C.; Webb, H.; Korley, K.; Patel, S. | 2012 | The sexual health behaviour of HIV positive patients in an urban UK cohort | A |

| | | | | |
|---|---|---|---|---|
| 729 | Pyzalski, J.; Wojtaszczyk, P. | 2004 | Activities of occupational medicine physicians in the area of workplace health promotion | A |
| 730 | Quaife, S. L.; Ruparel, M.; Beeken, R. J.; McEwen, A.; Isitt, J.; Nolan, G.; Sennett, K.; Baldwin, D. R.; Duffy, S. W.; Janes, S. M.; Wardle, J. | 2016 | The Lung Screen Uptake Trial (LSUT): Protocol for a randomised controlled demonstration lung cancer screening pilot testing a targeted invitation strategy for high risk and 'hard-to-reach' patients | A |
| 731 | Quaife, S. L.; Ruparel, M.; Dickson, J. L.; Beeken, R. J.; McEwen, A.; Baldwin, D. R.; Bhowmik, A.; Navani, N.; Sennett, K.; Duffy, S. W.; Wardle, J.; Waller, J.; Janes, S. M. | 2020 | Lung Screen Uptake Trial (LSUT): Randomized Controlled Clinical Trial Testing Targeted Invitation Materials | A |
| 732 | Rahman, Mushtaqur; , Simmons, Rebecca K.; , Harding, Anne-Helen; , Wareham, Nicholas J.; and Griffin, Simon J. | 2008 | A simple risk score identifies individuals at high risk of developing type 2 diabetes: A prospective cohort study | A |
| 733 | Rajan, S.; Kulkarni, S.; Kewalramani, N.; Madas, S.; Kulkarni, N. | 2016 | Spirometry in normal Indians: Towards establishing a new prediction equation | A |
| 734 | Ramadoss, P.; Raghupathi, N. | 2013 | Prevalence of chronic obstructive pulmonary disease and its relationship to smoke exposure among subjects attending a tertiary care center in south India for a routine health check | A |
| 735 | Ramagiri, R.; Kannuri, N. K.; Lewis, M. G.; Murthy, G. V. S.; Gilbert, C. | 2020 | Evaluation of whether health education using video technology increases the uptake of screening for diabetic retinopathy among individuals with diabetes in a slum population in Hyderabad | A |
| 736 | Ramaswamy, G.; Natarajan, S.; Janakiraman, L. | 2016 | Bone mineral content & density in Indian children with congenital adrenal hyperplasia | A |
| 737 | Ranjan, R.; Agarwal, N.; Kapur, P.; Marwah, A.; Parveen, R. | 2019 | Factors influencing participation of healthy volunteers in clinical trials: Findings from a cross-sectional study in Delhi, North India | A |
| 738 | Räsänen, V.; , Leinonen, V.; and Zaproudina, N. | 2005 | Indigenous healers' explanations of low back pain and its relief | A |
| 739 | Rauf, M.; Houlbrooke, A.; Birks, P.; Barnett, J.; Alison, B.; Owen, G.; Leyakathali Khan, S.; Hussain, I. | 2020 | Lung health check: should there be a general health check? | A |
| 740 | Reach, G.; , Michault, A.; , Bihan, H.; , Paulino, C.; , Cohen, R.; and Le Clesiau, H. | 2011 | Patients' impatience is an independent determinant of poor diabetes control | A |
| 741 | Rector, J. L.; Burns, V. E.; Jarczok, M. N.; Loerbroks, A.; Moss, P.; Fischer, J. E.; Hoffman, K.; Bosch, J. A. | 2013 | Consistent associations between measures of distress and cmv reactivation in a large occupational sample | A |
| 742 | Rectorc, J. L.; Burns, V. E.; Jarczok, M. N.; Loerbroks, A.; Moss, P.; Fischer, J. E.; Hoffman, K.; Bosch, J. A. | 2013 | Personality as a predictor of cytomegalovirus infection | A |
| 743 | Reddy, D. K.; , Jaladhar, P.; and Shetty, S. | 2018 | CORRELATION OF NECK CIRCUMFERENCE AND DYSLIPIDAEMIA | A |
| 744 | Reding, D. J.; Fischer, V.; Lappe, K.; Gunderson, P. | 1994 | Health education delivery by Wisconsin veterinarians | A |
| 745 | Reid, K. A.; , Smiley, E.; and Cooper, S. -A | 2011 | Prevalence and associations of anxiety disorders in adults with intellectual disabilities | A |

| | | | | |
|---|---|---|---|---|
| 746 | Reviriego, J.; Vázquez, L. A.; Goday, A.; Cabrera, M.; García-Margallo, M. T.; Calvo, E. | 2016 | Prevalence of impaired fasting glucose and type 1 and 2 diabetes mellitus in a large nationwide working population in Spain | A |
| 747 | Rha, J. H.; Park, H. K.; Park, J. J.; Yoo, N. Y. | 2012 | Is antiplatelet treatment effective on the progression of white matter hyperintensity?-5 year follow up, propensity score adjusted, observational study | A |
| 748 | Rhee, E. J.; , Choi, J. H.; , Yoo, S. H.; , Bae, J. C.; , Kim, W. J.; , Choi, E. S.; , Park, S. E.; , Park, C. Y.; , Park, S. W.; , Oh, K. W.; , Park, S. W.; , Kim, S. W.; and Lee, W. Y. | 2011 | The association of unintentional changes in weight, body composition, and homeostasis model assessment index with glycemic progression in non-diabetic healthy subjects | A |
| 749 | Rhee, E. J.; , Oh, K. W.; , Yun, E. J.; , Jung, C. H.; , Park, C. Y.; , Lee, W. Y.; , Oh, E. S.; , Baek, K. H.; , Kang, M. I.; , Park, S. W.; and Kim, S. W. | 2007 | The association of Pro12Ala polymorphism of peroxisome proliferator-activated receptor-gamma gene with serum osteoprotegerin levels in healthy Korean women | A |
| 750 | Rieck, T.; , Feig, M.; , Delere, Y.; and Wichmann, O. | 2014 | Utilization of administrative data to assess the association of an adolescent health check-up with human papillomavirus vaccine uptake in Germany | A |
| 751 | Riedner, G.; Hoffmann, O.; Rusizoka, M.; Mmbando, D.; Maboko, L.; Grosskurth, H.; Todd, J.; Hayes, R.; Hoelscher, M. | 2006 | Decline in sexually transmitted infection prevalence and HIV incidence in female barworkers attending prevention and care services in Mbeya Region, Tanzania | A |
| 752 | Riganis, A. | 2020 | Informatics and Cognitive Assessment: A RUDAS Scale Paradigm | A |
| 753 | Riglin, J.; Buttery, A.; Husk, J.; Grant, R.; Martin, F.; Potter, J. | 2011 | Examining older people's experiences of falls prevention services | A |
| 754 | Riley, V. A.; Gidlow, C.; Ellis, N. J.; Povey, R. J.; Barnes, O.; Clark-Carter, D. | 2019 | Improving cardiovascular disease risk communication in the UK national health service health check programme | A |
| 755 | Risal, P.; , Adhikari, B.; , Shrestha, R.; , Manandhar, S.; , Bhatt, R. D.; and Hada, M. | 2019 | Analysis of Factors Associated with Thyroid Dysfunction: A Hospital Based Study | A |
| 756 | Roberts, J.; Mundowa, G.; Jennings, R.; Yannaghas, H.; Gilleece, Y. | 2019 | New models of care with specialist women's clinics can provide better support for women living with HIV | A |
| 757 | Robertson, G.; , Fleming, A.; , Williams, M. C.; , Trucco, E.; , Quinn, N.; , Hogg, R.; , McKay, G. J.; , Kee, F.; , Young, I.; , Pellegrini, E.; , Newby, D. E.; , van Beek, E. J. R.; , Peto, T.; , Dhillon, B.; , van Hemert, J.; , MacGillivray, T. J.; and Northern Ireland Cohort, Longitu | 2020 | Association between hypertension and retinal vascular features in ultra-widefield fundus imaging | A |
| 758 | Robroek, S. J.; Brouwer, W.; Lindeboom, D.; Oenema, A.; Burdorf, A. | 2010 | Demographic, behavioral, and psychosocial correlates of using the website component of a worksite physical activity and healthy nutrition promotion program: a longitudinal study | A |
| 759 | Robroek, S. J.; Lindeboom, D. E.; Burdorf, A. | 2012 | Initial and sustained participation in an internet-delivered long-term worksite health promotion program on physical activity and nutrition | A |

| | | | | |
|---|---|---|---|---|
| 760 | Robroek, S. J.; Polinder, S.; Bredt, F. J.; Burdorf, A. | 2012 | Cost-effectiveness of a long-term Internet-delivered worksite health promotion programme on physical activity and nutrition: a cluster randomized controlled trial | A |
| 761 | Romppainen, K.; Saloniemi, A.; Kinnunen, U.; Liukkonen, V.; Virtanen, P. | 2014 | Does provision of targeted health care for the unemployed enhance re-employment? | A |
| 762 | Ruiz, Y.; Betancourt, E.; Miranda, C.; Hunter, R. F. | 2013 | Barriers for compliance to breast, colorectal, and cervical screening cancer test among Hispanics patients | A |
| 763 | Ruktanonchai, C. W.; , Ruktanonchai, N. W.; , Nove, A.; , Lopes, S.; , Pezzulo, C.; , Bosco, C.; , Alegana, V. A.; , Burgert, C. R.; , Ayiko, R.; , Charles, Asek; , Lambert, N.; , Msechu, E.; , Kathini, E.; , Matthews, Z.; and Tatem, A. J. | 2016 | Equality in Maternal and Newborn Health: Modelling Geographic Disparities in Utilisation of Care in Five East African Countries | A |
| 764 | Rungsinaporn, K.; and Phaisakamas, T. | 2008 | Frequency of abnormalities detected by upper abdominal ultrasound | A |
| 765 | Ruparel, M.; Dickson, J. L.; Quaife, S. L.; Bhowmik, A.; Taylor, M. N.; Ahmed, A.; Shaw, P. J.; Burke, S.; Soo, M. J.; Devaraj, A.; Navani, N.; Duffy, S. W.; Baldwin, D. R.; Waller, J.; Janes, S. M. | 2017 | Lung cancer risk profiles and eligibility of attendees in a lung cancer screening demonstration pilot | A |
| 766 | Ruparel, M.; Quaife, S. L.; Dickson, J. L.; Bhowmik, A.; Taylor, M. N.; Ahmed, A.; Shaw, P. J.; Burke, S.; Soo, M. J.; Devaraj, A.; Navani, N.; Duffy, S. W.; Baldwin, D. R.; Waller, J.; Janes, S. M. | 2017 | Identification and attendance of a high-risk cohort in a lung cancer screening demonstration pilot | A |
| 767 | Ruparel, M.; Quaife, S. L.; Dickson, J. L.; Horst, C.; Burke, S.; Taylor, M.; Ahmed, A.; Shaw, P.; Soo, M. J.; Nair, A.; Devaraj, A.; O'Dowd, E. L.; Bhowmik, A.; Navani, N.; Sennett, K.; Duffy, S. W.; Baldwin, D. R.; Sofat, R.; Patel, R. S.; Hingorani, A.; Janes, S. M. | 2019 | lugn cancer screening + CVD | A |
| 768 | Ruparel, M.; Quaife, S. L.; Dickson, J. L.; Tisi, S.; Hall, H.; Horst, C.; Taylor, M.; Ahmed, A.; Shaw, P.; Burke, S. J.; Soo, M.; Nair, A.; Devaraj, A.; Sennett, K.; Duffy, S. W.; Navani, N.; Bhowmik, A.; Baldwin, D. R.; Janes, S. M. | 2019 | Prevalence, symptom burden and under-diagnosis of COPD in a lung cancer screening cohort | A |
| 769 | Ruparel, M.; Quaife, S. L.; Dickson, J. L.; Horst, C.; Burke, S. J.; Taylor, M.; Ahmed, A.; Shaw, P.; Soo, M.; Nair, A.; Devaraj, A.; O'Dowd, E.; Bhowmik, A.; Navani, N.; Sennett, K.; Duffy, S. W.; Baldwin, D. R.; Sofatt, R.; Patel, R.; Hingorani, A.; Janes, S. M. | 2019 | Is lung cancer screening an opportunity to reduce cardiovascular mortality? | A |
| 770 | Ruparel, M.; Quaife, S. L.; Dickson, J. L.; Horst, C.; Tisi, S.; Hall, H.; Taylor, M.; Ahmed, A.; Shaw, P.; Burke, S.; Soo, M. J.; Nair, A.; Devaraj, A.; Sennett, K.; Duffy, S. W.; Navani, N.; Bhowmik, A.; Baldwin, D. R.; Janes, S. M. | 2020 | Lung Screen Uptake Trial: results from a single lung cancer screening round | A |

| | | | | |
|---|---|---|---|---|
| 771 | Ruparel, M.; Quaife, S. L.; Dickson, J. L.; Horst, C.; Tisi, S.; Hall, H.; Taylor, M. N.; Ahmed, A.; Shaw, P. J.; Burke, S.; Soo, M. J.; Nair, A.; Devaraj, A.; Sennett, K.; Hurst, J. R.; Duffy, S. W.; Navani, N.; Bhowmik, A.; Baldwin, D. R.; Janes, S. M. | 2020 | Prevalence, symptom burden, and underdiagnosis of chronic obstructive pulmonary disease in a lung cancer screening cohort | A |
| 772 | Rupasena, I. P. | 2017 | Analysis of attitudes of regular blood donors regarding blood donation and donor retention who donated blood into the national blood center, sri lanka | A |
| 773 | Rush, K. L.; Goma, F. M.; Barker, J. A.; Ollivier, R. A.; Ferrier, M. S.; Singini, D. | 2018 | Hypertension prevalence and risk factors in rural and urban Zambian adults in Western Province: A cross-sectional study | A |
| 774 | Rutter, M. K.; , Kane, K.; , Lunt, M.; , Cordingley, L.; , Littlewood, A.; , Young, H. S.; , Chew-Graham, C. A.; , Hilton, R.; , Symmons, D. P. M.; and Griffiths, C. E. M. | 2016 | Primary care-based screening for cardiovascular risk factors in patients with psoriasis | A |
| 775 | Ryoo, J. H.; , Kim, S. Y.; , Oh, C. M.; , Park, S. K.; , Kim, E.; , Park, S. J.; , Yu, J. I.; , Kim, M. G.; , Choi, Y. S.; and Ko, T. S. | 2015 | The incidental relationship between serum ferritin levels and hypertension | A |
| 776 | Ryoo, J. H.; Choi, J. M.; Moon, S. Y.; Suh, Y. J.; Shin, J. Y.; Shin, H. C.; Park, S. K. | 2013 | The clinical availability of non alcoholic fatty liver disease as an early predictor of the metabolic syndrome in Korean men: 5-Year's prospective cohort study | A |
| 777 | Ryu, K. J.; Park, H.; Kim, Y. J.; Yi, K. W.; Shin, J. H.; Hur, J. Y.; Kim, T. | 2018 | Moderate to severe vasomotor symptoms are risk factors for non-alcoholic fatty liver disease in postmenopausal women | A |
| 778 | Saari-Kemppainen, A. | 1995 | Use of antenatal care services in a controlled ultrasound screening trial | A |
| 779 | Sabates, R.; Feinstein, L. | 2006 | The role of education in the uptake of preventative health care: The case of cervical screening in Britain | A |
| 780 | Sadiq, A.; Maguire, K. A.; Morris, K. G. | 2015 | Structured reporting of adverse events in blood donors | A |
| 781 | Sagarad, S. V.; Sukhani, N.; Machanur, B.; Patil, S. | 2016 | Study to evaluate current trends in appropriate usage of tread mill exercise testing | A |
| 782 | Sakai, A.; , Ohira, T.; , Hosoya, M.; , Ohtsuru, A.; , Satoh, H.; , Kawasaki, Y.; , Suzuki, H.; , Takahashi, A.; , Kobashi, G.; , Ozasa, K.; , Yasumura, S.; , Yamashita, S.; , Kamiya, K.; , Abe, M.; and Fukushima Hlth Management Survey, G. | 2015 | White Blood Cell, Neutrophil, and Lymphocyte Counts in Individuals in the Evacuation Zone Designated by the Government After the Fukushima Daiichi Nuclear Power Plant accident: The Fukushima Health Management Survey | A |
| 783 | Sakai, A.; Ohira, T.; Hosoya, M.; Ohtsuru, A.; Satoh, H.; Kawasaki, Y.; Suzuki, H.; Takahashi, A.; Kobashi, G.; Ozasa, K.; Yasumura, S.; Yamashita, S.; Kamiya, K.; Abe, M. | 2015 | White blood cell, neutrophil, and lymphocyte counts in individuals in the evacuation zone designated by the government after the Fukushima Daiichi Nuclear Power Plant accident: the Fukushima Health Management Survey | A |
| 784 | Sakurada, K.; Konta, T.; Watanabe, M.; Ishizawa, K.; Ueno, Y.; Yamashita, H.; Kayama, T. | 2020 | Associations of Frequency of Laughter With Risk of All-Cause Mortality and Cardiovascular Disease Incidence in a General Population: Findings From the Yamagata Study | A |

| | | | | |
|---|---|---|---|---|
| 785 | Sakurai, R.; , Fujiwara, Y.; , Yasunaga, M.; , Nonaka, K.; , Suzuki, H.; , Oba, H.; , Fukaya, T.; , Watanabe, S.;  and Shinkai, S. | 2013 | [Association of confidence in motor function and fear of falling with physical ability in community-dwelling older people] | A |
| 786 | Sakurai, R.; Fujiwara, Y.; Yasunaga, M.; Nonaka, K.; Suzuki, H.; Oba, H.; Fukaya, T.; Watanabe, S.; Shinkai, S. | 2013 | Association of confidence in motor function and fear of falling with physical ability in community-dwelling older people | A |
| 787 | Saldanha, M.; Brown, K.; Heap, D.; Mech, C.; Deamude, M.; Kislinsky, K.; Mc-Clory, D.; Shah, A.; Bensen, W. G. | 2013 | A study to determine patient satisfaction with a nurse-led injection clinic in rheumatology | A |
| 788 | Saldanha, M.; Brown, K.; Heap, D.; Deamude, M.; Mech, C.; Bensen, W.; McClory, D.; Kislinkky, K. | 2014 | A study to determine patient satisfaction with a nurse injection clinic in rheumatology | A |
| 789 | Salinas, M.; , López-Garrigós, M.; , Flores, E.;  and Leiva-Salinas, C. | 2017 | Serum Uric Acid Laboratory Test Request Patterns in Primary Care: How Panels May Contribute to Overutilization and Treatment of Asymptomatic Patients | A |
| 790 | Salinas, M.; Flores, E.; Lopez-Garrigos, M.; Mar-Medina, C.; Herranz-Puebla, M.; Aguayo-Gredilla, F.; Prada De Medio, E.; Gascón, F.; Garcia-Chico, P.; Pesudo, S.; García-Menéndez, L.; Esteve, P.; Martin-Fernández de Basoa, M. C.; Garrido, J. C.; Domínguez, J.; Calvo-Nieves, M. D.; Pérez-Fuentes, A.; Guillen-Santos, R.; Cava, F.; Leiva-Salinas, C. | 2017 | Request pattern, preanalytical and analytical variability and economic costs of urinalysis in primary care | A |
| 791 | Salinas, M.; López-Garrigós, M.; Flores, E.; Giménez-Marín, Á; Herrera-Contreras, I.; Roldán-Fontana, E.; Rabadán, L.; Buño, A.; Gallego-Ramírez, M. C.; Poncela-Garcia, M. V.; Ortuño, M.; Colomo, L. F.; Villamandos, V.; Plata, M. C.; Tapia, A.; Sole-Llop, M. E.; Miralles, A.; Rodríguez-Rodríguez, M. A.; Máiz-Suárez, L.; Leiva-Salinas, C. | 2017 | Serum uric acid laboratory test in primary care: A high requested inexpensive test with potentially costly adverse effects | A |
| 792 | Salomaa, V. V.; , Strandberg, T. E.; , Vanhanen, H.; , Naukkarinen, V.; , Sarna, S.;  and Miettinen, T. A. | 1991 | GLUCOSE-TOLERANCE AND BLOOD-PRESSURE - LONG-TERM FOLLOW-UP IN MIDDLE-AGED MEN | A |
| 793 | Salomaa, V. V.; Strandberg, T. E.; Vanhanen, H.; Naukkarinen, V.; Sarna, S.; Miettinen, T. A. | 1991 | Glucose tolerance and blood pressure: Long term follow up in middle aged men | A |
| 794 | Sanders, D.; Fowler, G.; Mant, D.; Fuller, A.; Jones, L.; Marzillier, J. | 1989 | Randomized controlled trial of anti-smoking advice by nurses in general practice | A |
| 795 | Santman-Berends, Imga; , Brouwer, H.; , Ten Wolthuis-Bronsvoort, A.; , De Bont-Smolenaars, A. J. G.; , Haarman-Zantinge, S.;  and Van Schaik, G. | 2018 | Development of an objective and uniform scoring method to evaluate the quality of rearing in Dutch dairy herds | A |
| 796 | Sarkar, S.; Balasundaram, S.; Backer, A. | 2015 | Consultation-liaison services in a tertiary hospital of pondicherry | A |
| 797 | Sasaki, M.; Harada, S.; Kawasaki, Y.; Tsubota, K.; Takebayashi, T.; Nishiwaki, Y.; Kawasaki, R. | 2019 | Dietary intake of saturated fatty acid and early age-related macular degeneration in a Japanese population | A |
| 798 | Sasaki, M.; Harada, S.; Tsubota, K.; Yasukawa, T.; Takebayashi, T.; Nishiwaki, Y.; Kawasaki, R. | 2020 | Dietary saturated fatty acid intake and early age-related macular degeneration in a Japanese population | A |

| | | | | |
|---|---|---|---|---|
| 799 | Sato, K.; , Shibata, Y.; , Abe, S.; , Inoue, S.; , Igarashi, A.; , Yamauchi, K.; , Ai-da, Y.; , Nunomiya, K.; , Nakano, H.; , Sato, M.; , Kimura, T.; , Nemoto, T.; , Watanabe, T.; , Konta, T.; , Ueno, Y.; , Kato, T.; , Kayama, T.; and Kubota, I. | 2014 | Association between Plasma Adiponectin Levels and Decline in Forced Expiratory Volume in 1 s in a General Japanese Population: The Takahata Study | A |
| 800 | Sato, K.; Shibata, Y.; Abe, S.; Inoue, S.; Igarashi, A.; Yamauchi, K.; Aida, Y.; Nunomiya, K.; Nakano, H.; Sato, M.; Kimura, T.; Nemoto, T.; Kubota, I. | 2014 | Association of plasma adiponectin level with pulmonary function in a general Japanese population | A |
| 801 | Sato, M.; , Shibata, Y.; , Abe, S.; , Inoue, S.; , Igarashi, A.; , Yamauchi, K.; , Aida, Y.; , Kishi, H.; , Nunomiya, K.; , Nakano, H.; , Sato, K.; , Watanabe, T.; , Konta, T.; , Ueno, Y.; , Kato, T.; , Kayama, T.; and Kubota, I. | 2013 | Retrospective Analysis of the Relationship between Decline in FEV1 and Abdominal Circumference in Male Smokers: the Takahata Study | A |
| 802 | Savinainen, M. S.; Nyberg, ; Merivirta, | 2013 | Efficiency of occupational health co-operation in small forestry enterprises | A |
| 803 | Sawant, A. M.; Shetty, D.; Mankeshwar, R.; Ashavaid, T. F. | 2008 | Prevalence of dyslipidemia in young adult Indian population | A |
| 804 | Sawyer, Alyssa; , Kaim, Amy; , Le, Huynh-Nhu; , McDonald, Denise; , Mittinty, Murthy; , Lynch, John; and Sawyer, Michael | 2019 | The effectiveness of an app-based nurse-moderated program for new mothers with depression and parenting problems (eMums plus): Pragmatic randomized controlled trial | A |
| 805 | Sawyer, M. G.; Reece, C. E.; Bowering, K.; Jeffs, D.; Sawyer, A. C. P.; Peters, J. D.; Mpundu-Kaambwa, C.; Clark, J. J.; McDonald, D.; Mittinty, M. N.; Lynch, J. W. | 2016 | Usage, adherence and attrition: How new mothers engage with a nurse-moderated web-based intervention to support maternal and infant health. A 9-month observational study | A |
| 806 | Sawyer, M. G.; Reece, C. E.; Bowering, K.; Jeffs, D.; Sawyer, A. C. P.; Mittinty, M.; Lynch, J. W. | 2017 | Nurse-Moderated Internet-Based Support for New Mothers: Non-Inferiority, Randomized Controlled Trial | A |
| 807 | Sayan, S.; , Pekin, T.; and Yildizhan, B. | 2018 | Relationship between vasomotor symptoms and metabolic syndrome in postmenopausal women | A |
| 808 | Scheel, J. R.; Parker, S.; Hippe, D. S.; Patrick, D. L.; Nakigudde, G.; Anderson, B. O.; Gralow, J. R.; Thompson, B.; Molina, Y. | 2019 | Role of Family Obligation Stress on Ugandan Women's Participation in Preventive Breast Health | A |
| 809 | Schluter, P. J.; Askew, D. A.; Spurling, G. K.; Lee, M.; Hayman, N. | 2017 | Aboriginal and Torres Strait Islander oral health and its impact among adults: A cross-sectional study | A |
| 810 | Schluter, P. J.; Kokaua, J.; Lee, M. | 2020 | Severe early childhood caries: a modern (neglected) epidemic? | A |
| 811 | Schmid, K.; Jüngert, B.; Hager, M.; Drexler, H. | 2009 | Is there a need for special preventive medical check-ups in employees exposed to experimental animal dust? | A |
| 812 | Schneider, M.; Martin, S.; Hilgers, R. D.; Dugi, K.; Wolmar, C. W. V.; Haastert, B.; Kempf, K. | 2012 | Boehringer Ingelheim employee study: A prospective epidemiological cohort study diabetes prevalence and association between impaired glucose metabolism and risk factors | A |

| | | | | |
|---|---|---|---|---|
| 813 | Scholl, J.; Bots, M. L.; Peters, S. A. E. | 2015 | Contribution of cardiorespiratory fitness, relative to traditional cardiovascular disease risk factors, to common carotid intima-media thickness | A |
| 814 | Scholl, J.; Kurz, P. U. | 2018 | New risk category OVERFAT as a good predictor of cardiometabolic risk | A |
| 815 | Schouten, L. S.; , Bultmann, U.; , Heymans, M. W.; , Joling, C. I.; , Twisk, J. W. R.; and Roelen, C. A. M. | 2016 | Shortened version of the work ability index to identify workers at risk of long-term sickness absence | A |
| 816 | Schreiber Pedersen, L.; Lose, G.; Hoybye, M.; Juergensen, M.; Waldmann, A.; Rudnicki, M. | 2017 | Triggers and barriers for help-seeking behavior among women with urinary incontinence: A cross-national study in Germany and Denmark | A |
| 817 | Schtze, H.; Rix, E. F.; Laws, R. A.; Passey, M.; Fanaian, M.; Harris, M. F. | 2012 | How feasible are lifestyle modification programs for disease prevention in general practice? | A |
| 818 | Schultz, R. | 2012 | Prevalences of overweight and obesity among children in remote Aboriginal communities in central Australia | A |
| 819 | Schuster, M.; Claus, M.; Webendörfer, S.; Oberlinner, C. | 2018 | The association of age and chronotype in day and rotating shift workers of a large German chemical company | A |
| 820 | Seki, N.; Tonai, K.; Hamano, S.; Hirai, H.; Noda, N.; Nakano, T.; Fukuyama, S.; Matsumoto, K.; Takada, S.; Inoue, H.; Nakanishi, Y. | 2013 | Prevalence of asthma, COPD, and COPD with variable airflow obstruction in a general Japanese population: The hisayama study | A |
| 821 | Selekler, H. M.; , Gokmen, G.; , Alvur, T. M.; and Steiner, T. J. | 2015 | Productivity losses attributable to headache, and their attempted recovery, in a heavy-manufacturing workforce in Turkey: implications for employers and politicians | A |
| 822 | Selekler, M. H.; Gökmen, G.; Steiner, T. J. | 2013 | Productivity impact of headache on a heavy-manufacturing workforce in Turkey | A |
| 823 | Selekler, M. H.; Steiner, T. J. | 2013 | Productivity impact of headache on a heavy-manufacturing workforce in Turkey | A |
| 824 | Selvakumar, M.; , Karthikeyan, R.; , Punithavathi, K.; , Amuthavalli, K.; and Anandan, H. | 2017 | Master Health Check-up Attendees in High Risk Group for Sexually Transmitted Infections over a Period of 15-Month in a Tertiary Care Hospital: A Retrospective Study | A |
| 825 | Senbanjo, Richard; and Strang, John | 2011 | The needle and the damage done: Clinical and behavioural markers of severe femoral vein damage among groin injectors | A |
| 826 | Senbanjo, R.; Tipping, T.; Hunt, N.; Strang, J. | 2012 | Injecting drug use via femoral vein puncture: Preliminary findings of a point-of-care ultrasound service for opioid-dependent groin injectors in treatment | A |
| 827 | Seo, H. J.; Kim, S. G.; Kim, C. S.; Chang, Y. K.; Park, I. G. | 2006 | The incidence and risk factors of hypertension that developed in a male-workers' cohort for 3 years | A |
| 828 | Seraji, M.; and Rakhshani, F. | 2016 | Evaluation of the Effect of Educating Self Care Behavior of Heart failure Patients on Economy of Health | A |

| | | | | |
|---|---|---|---|---|
| 829 | Seraji, M.; Rakhshani, F.; Khorasani, E. | 2017 | Cost saving due to self-care behavior of cardiovascular patients | A |
| 830 | Sezgin, G.; Georgiou, A. | 2019 | Vitamin d test ordering practices of Australian general practitioners between 2007 and 2017: Evidence from electronic health records | A |
| 831 | Shahim, B.; , Hasselberg, S.; , Boldt-Christmas, O.; , Gyberg, V.; , Mellbin, L.; and Ryden, L. | 2018 | Effectiveness of different outreach strategies to identify individuals at high risk of diabetes in a heterogeneous population: a study in the Swedish municipality of Sodertalje | A |
| 832 | Shankar, M. | 2013 | Models of care & activities to prevent osteoporos is and fractures in bangalore (South India): A reflection and response to call for action | A |
| 833 | Shanmugam, L.; Green, S. R.; Radhakrishnan, H.; Kadavanu, T. M.; Ramachandrappa, A.; Tiwari, S. R.; Rajkumar, A. L.; Govindasamy, E. | 2016 | Trace elements in chronic haemodialysis patients and healthy individuals-A comparative study | A |
| 834 | Shannon, O. M.; Stephan, B. C. M.; Granic, A.; Lentjes, M.; Hayat, S.; Mulligan, A.; Brayne, C.; Khaw, K. T.; Bundy, R.; Aldred, S.; Hornberger, M.; Paddick, S. M.; Muniz-Tererra, G.; Minihane, A. M.; Mathers, J. C.; Siervo, M. | 2019 | Mediterranean diet adherence and cognitive function in older UK adults: The European Prospective Investigation into Cancer and Nutrition-Norfolk (EPIC-Norfolk) Study | A |
| 835 | Sheriff, June N.; and Chenoweth, Lynn | 2006 | Promoting healthy ageing for those over 65 with the health check log: A pilot study | A |
| 836 | Sherwin, J. C.; Khawaja, A. P.; Broadway, D.; Luben, R.; Hayat, S.; Dalzell, N.; Wareham, N. J.; Khaw, K. T.; Foster, P. J. | 2012 | Uncorrected refractive error in older British adults: The EPIC-Norfolk eye study | A |
| 837 | Shiba, Y.; Anzai, S.; Ueki, S.; Haga, H. | 2015 | Factors related to social participation in Japanese: Comparison of Urban and Rural | A |
| 838 | Shibata, Y.; , Abe, S.; , Inoue, S.; , Igarashi, A.; , Yamauchi, K.; , Aida, Y.; , Kishi, H.; , Nunomiya, K.; , Nakano, H.; , Sato, M.; , Sato, K.; , Kimura, T.; , Nemoto, T.; , Watanabe, T.; , Konta, T.; , Ueno, Y.; , Kato, T.; , Kayama, T.; and Kubota, I. | 2013 | Relationship between Plasma Fibrinogen Levels and Pulmonary Function in the Japanese Population: The Takahata Study | A |
| 839 | Shibata, Y.; Inoue, S.; Abe, S.; Igarashi, A.; Kubota, I. | 2013 | Elevated serum iron as a marker for spirometric resistance to cigarette smoke: The Takahata study | A |
| 840 | Shibata, Y.; Watanabe, T.; Osaka, D.; Abe, S.; Inoue, S.; Tokairin, Y.; Igarashi, A.; Yamauchi, K.; Kimura, T.; Kishi, H.; Aida, Y.; Nunomiya, K.; Nemoto, T.; Sato, M.; Konta, T.; Kawata, S.; Kato, T.; Kayama, T.; Kubota, I. | 2011 | Impairment of pulmonary function is an independent risk factor for atrial fibrillation: The takahata study | A |
| 841 | Shidei, H.; , Maeda, H.; , Isaka, T.; , Matsumoto, T.; , Yamamoto, T.; , Nagashima, Y.; and Kanzaki, M. | 2020 | Mediastinal paraganglioma successfully resected by robot-assisted thoracoscopic surgery with en bloc chest wall resection: a case report | A |
| 842 | Shimizu, Y.; Kawashiri, S. Y.; Yamanashi, H.; Koyamatsu, J.; Fukui, S.; Kondo, H.; Tamai, M.; Nakamichi, S.; Maeda, T. | 2019 | Reticulocyte levels have an ambivalent association with hypertension and atherosclerosis in the elderly: A cross-sectional study | A |
| 843 | Shimizu, Y.; Nabeshima-Kimura, Y.; Kawashiri, S. Y.; Noguchi, Y.; Nagata, Y.; Maeda, T.; Hayashida, N. | 2020 | Anti-thyroid peroxidase antibody and thyroid cysts among the general Japanese population: A cross-sectional study | A |

| | | | | |
|---|---|---|---|---|
| 844 | Shimizu, Y.; Sato, S.; Noguchi, Y.; Koyamatsu, J.; Yamanashi, H.; Nagayoshi, M.; Kadota, K.; Kawashiri, S. Y.; Nagata, Y.; Maeda, T. | 2017 | Triglycerides and blood pressure in relation to circulating CD34-positive cell levels among community-dwelling elderly Japanese men: A cross-sectional study | A |
| 845 | Shimizu, Y.; Sato, S.; Noguchi, Y.; Koyamatsu, J.; Yamanashi, H.; Higashi, M.; Nagayoshi, M.; Kawashiri, S. Y.; Nagata, Y.; Takamura, N.; Maeda, T. | 2018 | Association between tongue pressure and subclinical carotid atherosclerosis in relation to platelet levels in hypertensive elderly men: A cross-sectional study | A |
| 846 | Shimizu, Y.; Yamanashi, H.; Noguchi, Y.; Koyamatsu, J.; Nagayoshi, M.; Kiyoura, K.; Fukui, S.; Tamai, M.; Kawashiri, S. Y.; Arima, K.; Maeda, T. | 2018 | Association of hemoglobin concentration with handgrip strength in relation to hepatocyte growth factor levels among elderly Japanese men aged 60-69 years: A cross-sectional study | A |
| 847 | Shimizu, Y.; Yamanashi, H.; Kitamura, M.; Furugen, R.; Iwasaki, T.; Fukuda, H.; Hayashida, H.; Kawasaki, K.; Kiyoura, K.; Kawashiri, S. Y.; Saito, T.; Kawakami, A.; Maeda, T. | 2019 | Association between human T cell leukemia virus type-1 (HTLV-1) infection and advanced periodontitis in relation to atherosclerosis among elderly Japanese: A cross-sectional study | A |
| 848 | Shimizu, Y.; Yamanashi, H.; Kitamura, M.; Furugen, R.; Iwasaki, T.; Fukuda, H.; Hayashida, H.; Kawasaki, K.; Kiyoura, K.; Kawashiri, S. Y.; Saito, T.; Kawakami, A.; Maeda, T. | 2019 | Association between human T cell leukemia virus 1 (HTLV-1) infection and advanced periodontitis in relation to hematopoietic activity among elderly participants: A cross-sectional study | A |
| 849 | Shimizu, Y.; Yamanashi, H.; Noguchi, Y.; Koyamatsu, J.; Nagayoshi, M.; Kiyoura, K.; Fukui, S.; Tamai, M.; Kawashiri, S. Y.; Arima, K.; Maeda, T. | 2019 | Short stature-related single-nucleotide polymorphism (SNP) activates endothelial repair activity in elderly Japanese | A |
| 850 | Shin, H. Y.; Park, S.; Park, S. M. | 2018 | Gaps in health behaviours and use of preventive services between patients with diabetes and the general population: A population-based cross-sectional study | A |
| 851 | Shin, J. Y.; Hwang, J. H.; Jeong, J. Y.; Kim, S. H.; Moon, J. D.; Roh, S. C.; Kim, Y. W.; Kim, Y.; Leem, J. H.; Ju, Y. S.; Hong, Y. S.; Ha, E. H.; Lee, Y. H.; Lee, D. H.; Kim, D. H. | 2009 | The association of central obesity with type 2 diabetes among Koreans according to the serum gamma-glutamyltransferase level: Korean genome and epidemiology study | A |
| 852 | Shin, Y. H.; Kim, K. E.; Lee, Y. J.; Nam, J. H.; Hong, Y. M.; Shin, H. J. | 2014 | Associations of matrix metalloproteinase (MMP)-8; MMP-9; and their inhibitor, tissue inhibitor of metalloproteinase-1, with obesity-related biomarkers in apparently healthy adolescent boys | A |
| 853 | Shinkai, S.; Yoshida, H.; Taniguchi, Y.; Murayama, H.; Nishi, M.; Amano, H.; Nofuji, Y.; Seino, S.; Fujiwara, Y. | 2016 | Public health approach to preventing frailty in the community and its effect on healthy aging in Japan | A |
| 854 | Shinkoda, H.; , Suetsugu, Y.; , Asami, E.; , Kato, N.; , Kohyama, J.; , Uchimura, N.; , Chishaki, A.; , Nishioka, K.; , Okubo, I.; , Matsumoto, K.; , Nanbu, Y.; and Kaku, T. | 2012 | [Analysis of parent-child sleeping and living habits related to later bedtimes in children] | A |
| 855 | Shiotani, A.; , Miyanishi, T.; , Uedo, N.; and Iishi, H. | 2005 | Helicobacter pylori infection is associated with reduced circulating ghrelin levels independent of body mass index | A |
| 856 | Shreeves, K.; Buzzacott, P.; Hornsby, A.; Caney, M. | 2018 | Violations of safe diving practices among 122 diver fatalities | A |
| 857 | Shrestha, D.; Dhakal, A. K.; Shakya, A.; Shah, S. C.; Shakya, H.; Mehata, S.; Sadeh, A. | 2015 | Pattern of sleep in infants and toddlers visiting at a teaching hospital in Lalitpur | A |

| | | | | |
|---|---|---|---|---|
| 858 | Shuba, N.; , Praba, V.; and Prithiviraaj, P. | 2018 | ASSOCIATION OF PLATELET INDICES WITH DISEASE ACTIVITY IN RHEUMATOID ARTHRITIS | A |
| 859 | Siconolfi, D. E.; Kapadia, F.; Halkitis, P. N.; Moeller, R. W.; Storholm, E. D.; Barton, S. C.; Solomon, T. M.; Jones, D. | 2013 | Sexual health screening among racially/ethnically diverse young gay, bisexual, and other men who have sex with men | A |
| 860 | Silva, A.; Brito, I.; Amado, J. | 2013 | Do you have a fantastic lifestyle? nights out and youth lifestyles | A |
| 861 | Sim, M.; Gwini, S.; Roberts, M.; Kelsall, H. | 2012 | The workhealth programme: Cardiovascular disease and diabetes risk factors in 400,000 Victorian workers | A |
| 862 | Sim, M.; Kelsall, H.; Mohebbi, M.; Botlero, R. | 2013 | Follow up study of work health check participants: Evaluation of lifestyle changes and other outcomes | A |
| 863 | Simmons, R. K.; , Harding, A. H.; , Wareham, N. J.; , Griffin, S. J.; and Team, E. PIC-Norfolk Project | 2007 | Do simple questions about diet and physical activity help to identify those at risk of Type 2 diabetes? | A |
| 864 | Simmons, R. K.; , Sharp, S.; , Boekholdt, S. M.; , Sargeant, L. A.; , Khaw, K. T.; , Wareham, N. J.; and Griffin, S. J. | 2008 | Evaluation of the Framingham risk score in the European Prospective Investigation of Cancer-Norfolk cohort - Does adding glycated hemoglobin improve the prediction of coronary heart disease events? | A |
| 865 | Simmons, R. K.; Harding, A. H.; Jakes, R. W.; Welch, A.; Wareham, N. J.; Griffin, S. J. | 2006 | How much might achievement of diabetes prevention behaviour goals reduce the incidence of diabetes if implemented at the population level? | A |
| 866 | Simmons, R. K.; Harding, A. H.; Wareham, N. J.; Griffin, S. J. | 2007 | Do simple questions about diet and physical activity help to identify those at risk of Type 2 diabetes? | A |
| 867 | Simmons, R. K.; Sharp, S.; Boekholdt, S. M.; Sargeant, L. A.; Khaw, K. T.; Wareham, N. J.; Griffin, S. J. | 2008 | Evaluation of the Framingham risk score in the European prospective investigation of cancer-Norfolk cohort: Does adding glycated hemoglobin improve the prediction of coronary heart disease events? | A |
| 868 | Singh, A.; Purohit, B. M.; Masih, N. | 2016 | Geriatric oral health predicaments in New Delhi, India | A |
| 869 | Singh, S. B.; Pokharel, P. K.; Raut, P.; Mehta, K. | 2015 | Study of the effects of pesticide exposure among the workers of tea estates | A |
| 870 | Sjøgaard, G. | 2012 | Evidence of worksite physical exercise training to promote health in jobs ranging from low to high occupational physical demands | A |
| 871 | Sjogaard, G.; , Justesen, J. B.; , Murray, M.; , Dalager, T.; and Sogaard, K. | 2014 | A conceptual model for worksite intelligent physical exercise training - IPET - intervention for decreasing life style health risk indicators among employees: a randomized controlled trial | A |
| 872 | Sjøgaard, G.; Justesen, B. | 2014 | Intelligent physical exercise training intervention at the workplace for health promotion among office workers: A randomized controlled trial | A |
| 873 | Smith, P.; May, C.; Santamaria, N.; Hendrie, D.; Sheehan, M.; Hung, J.; Chan, K.; Vickery, A.; Williamson, J.; Thompson, P. | 2011 | Randomised comparison of remote monitoring versus enhanced standard care for heart failure | A |

| | | | | |
|---|---|---|---|---|
| 874 | Smith, S.; , Yeomans, D.; , Bushe, C. J.; , Eriksson, C.; , Harrison, T.; , Holmes, R.; , Mynors-Wallis, L.; , Oatway, H.; and Sullivan, G. | 2007 | A well-being programme in severe mental illness. Baseline findings in a UK cohort | A |
| 875 | Sofue, T.; , Okano, Y.; , Matsushita, N.; , Moritoki, M.; , Nishijima, Y.; , Fujioka, H.; , Yamasaki, Y.; , Yamanaka, M.; , Nishiyama, A.; , Minamino, T.; , Shimizu, M.; , Yokoi, T.; , Mitsunaka, H.; , Akiyama, K.; , Akashi, Y.; , Obayashi, H.; , Ishizu, T.; , Matsubara, K.; , Takahashi, N.; and Kagawa Assoc Chronic, Kidney | 2019 | The effects of a participatory structured group educational program on the development of CKD: a population-based study | A |
| 876 | Sohn, H. S.; , Kim, J. R.; , Ryu, S. Y.; , Lee, Y. J.; , Lee, M. J.; , Min, H. J.; , Lee, J.; , Choi, H. Y.; , Song, Y. J.; and Ki, M. | 2016 | Risk Factors for Hepatitis C Virus (HCV) Infection in Areas with a. High Prevalence of HCV in the Republic of Korea in 2013 | A |
| 877 | Somashekhar, S. P.; , Vijay, R.; , Ananthasivan, R.; and Prasanna, G. | 2016 | Noninvasive and Low-Cost Technique for Early Detection of Clinically Relevant Breast Lesions Using a Handheld Point-of-Care Medical Device (iBreastExam): Prospective Three-Arm Triple-Blinded Comparative Study | A |
| 878 | Son, J.; Koh, H.; Son, J. | 2016 | The association between intraocular pressure and different combination of metabolic syndrome components | A |
| 879 | Soneson, E.; Childs-Fegredo, J.; Anderson, J. K.; Stochl, J.; Fazel, M.; Ford, T.; Humphrey, A.; Jones, P. B.; Howarth, E. | 2018 | Acceptability of screening for mental health difficulties in primary schools: a survey of UK parents | A |
| 880 | Song, J. U.; Hwang, J.; Ahn, J. K. | 2017 | Serum uric acid is positively associated with pulmonary function in Korean health screening examinees | A |
| 881 | Song, M.; Ware, R.; Doan, T. N.; Harley, D. | 2020 | Psychotropic medication use in adults with intellectual disability in Queensland, Australia, from 1999 to 2015: a cohort study | A |
| 882 | Sowmya, K.; Prabu Kumar, C. M.; Vishnu, | 2015 | Measurement of neck circumference as an indicator of abdominal obesity | A |
| 883 | Spiryda, L. B.; Brown, S.; Gwynne, M.; Handley, A. | 2014 | Outcomes of the cervix project: Increasing knowledge, attitudes, and practices of university of south carolina undergraduates regarding human papil-lomavirus (HPV), screening for HPV, and vaccination against HPV | A |
| 884 | Spyckerelle, Y.; , Steinmetz, J.; , Fournier, B.; , Giordanella, J. P.; , Boulange, M.; and De Talence, N. | 2000 | Borderline hypothyroidism: epidemiological aspects in women aged 45-70 years | A |
| 885 | Srinivas, M.; Srinivasan, V.; Mohan, M. B.; Varghese, J.; Venkataraman, J. | 2015 | A study of gender-wise risk association between fatty liver and metabolic syndrome components (Asia-Pacific criteria) in a South Indian urban cohort | A |
| 886 | Stafylis, C.; Bristow, C. C.; Natoli, L. J.; Salow, K. R.; Davidson, E.; Granados, Y.; McGrath, M.; Klausner, J. D. | 2019 | Field evaluation of a dual rapid Human Immunodeficiency Virus and treponemal syphilis rapid test in community-based clinics in Los Angeles and New York | A |

| | | | | |
|---|---|---|---|---|
| 887 | Stary, A.; , Steyrer, K.; , Heller-Vitouch, C.; , Müller, I.; and Mårdh, P. A. | 1991 | Screening for Chlamydia trachomatis in military personnel by urine testing | A |
| 888 | Stattin, P.; Johansson, R.; Lodnert, R.; Andrén, O.; Bill-Axelsson, A.; Bratt, O.; Damber, J. E.; Hellström, M.; Hugosson, J.; Lundgren, R.; Törnblom, M.; Varenhorst, E.; Johansson, J. E. | 2005 | Geographical variation in incidence of prostate cancer in Sweden: Survey from the National Prostate Cancer Register | A |
| 889 | Steinmetz, J.; , Spyckerelle, Y.; , Henny, J.; , Giordanella, J. P.; and Emmanuelli, J. | 2001 | [Screening for colorectal cancer. Study of a population attending a public health clinic] | A |
| 890 | Stevens, B.; Debattista, J.; Rutkin, W. | 2013 | Use of incentives to encourage sexually transmissible infection testing amongst sex-on-premises venue patrons | A |
| 891 | Stewart, K. F. J.; , Wesselius, A.; , Schols, Amwj; and Zeegers, M. P. | 2018 | Stages of behavioural change after direct-to-consumer disease risk profiling: study protocol of two integrated controlled pragmatic trials | A |
| 892 | Stoupa, A.; , Goischke, A.; , Garcin, C.; , Elie, C.; , Viaud, M.; , Thery, A.; , Richard, G.; and Polak, M. | 2015 | Can school health check-ups serve as screening tool for growth anomalies and obesity in children? | A |
| 893 | Strand, L. B.; Tsai, M. K.; Gunnell, D.; Janszky, I.; Wen, C. P.; Chang, S. S. | 2016 | Self-reported sleep duration and coronary heart disease mortality: A large cohort study of 400,000 Taiwanese adults | A |
| 894 | Strandberg, T. E.; , Salomaa, U. V.; , Naukkarinen, V. A.; , Vanhanen, H. T.; , Sarna, S. J.; and Miettinen, T. A. | 1995 | CARDIOVASCULAR MORBIDITY AND MULTIFACTORIAL PRIMARY PREVENTION - 15-YEAR FOLLOW-UP OF THE HELSINKI BUSINESSMEN STUDY | A |
| 895 | Strandberg, T. E.; , Salomaa, V. V.; , Vanhanen, H. T.; , Naukkarinen, V. A.; , Sarna, S. J.; and Miettinen, T. A. | 1995 | MORTALITY IN PARTICIPANTS AND NON-PARTICIPANTS OF A MULTIFACTORIAL PREVENTION STUDY OF CARDIOVASCULAR-DISEASES - A 28-YEAR FOLLOW-UP OF THE HELSINKI BUSINESSMEN STUDY | A |
| 896 | Strandberg, T. E.; Salomaa, V. V.; Vanhanen, H. T.; Pitkala, K. | 2001 | Blood pressure and mortality during an up to 32-year follow-up | A |
| 897 | Strawbridge, W. J.; Shema, S. J.; Cohen, R. D.; Kaplan, G. A. | 2001 | Religious attendance increases survival by improving and maintaining Good health behaviors, mental health, and social relationships | A |
| 898 | Su, T. C.; Liao, C. C.; Chien, K. L.; Hsu, S. H. J.; Sung, F. C. | 2014 | An overweight or obese status in childhood predicts subclinical atherosclerosis and prehypertension/hypertension in young adults | A |
| 899 | Subhashree, A. R.; Parameaswari, P. J.; Shanthi, B.; Carnagarin, R.; Parijatham, B. O. | 2012 | The reference intervals for the haematological parameters in healthy adult population of Chennai, Southern India | A |
| 900 | Sugiyama, N.; Igarashi, Y.; Kohda, E.; Kohno, A.; Suzuki, S.; Tanaka, T.; Chiyasu, S.; Yokokura, T. | 2005 | Use of compressed images for medical checkups: Comparison between radiologists' subjective quality assessment and diagnostic usefulness | A |
| 901 | Suh, S.; Bae, J. C.; Jin, S. M.; Jee, J. H.; Park, M. K.; Kim, D. K.; Kim, J. H. | 2017 | Serum calcium changes and risk of type 2 diabetes mellitus in Asian population | A |

| | | | | |
|---|---|---|---|---|
| 902 | Sulaiman, N.; Albadawi, S.; Abusnana, S.; Fikri, M.; Madani, A.; Mairghani, M.; Alawadi, F.; Zimmet, P.; Shaw, J. | 2015 | Novel approach to systematic random sampling in population surveys: Lessons from the United Arab Emirates National Diabetes Study (UAEDIAB) | A |
| 903 | Sun, S.; Wu, Y.; Batteson, R.; van Keep, M. | 2019 | Cost-effectiveness model of antihemophilic factor (recombinant) versus emicizumab treatment of patients with severe hemophilia a without inhibitors | A |
| 904 | Sun, S. X.; Wu, Y.; McDermott, M.; Van Keep, M. | 2019 | Cost-effectiveness model of recombinant FVIII versus emicizumab treatment of patients with severe hemophilia a without inhibitors | A |
| 905 | Sung, K. C.; and Rhee, E. J. | 2007 | Glycated haemoglobin as a predictor for metabolic syndrome in non-diabetic Korean adults | A |
| 906 | Sung, K. C.; Byrne, C. D.; Ryu, S.; Lee, J. Y.; Lee, S. H.; Kim, J. Y.; Kim, S. H.; Wild, S. H.; Guallar, E. | 2017 | Baseline and change in uric acid concentration over time are associated with incident hypertension in large Korean cohort | A |
| 907 | Sung, K. C.; Rhee, E. J.; Kim, H.; Park, J. B.; Kim, Y. K.; Rosenson, R. S. | 2012 | Prevalence of low LDL-cholesterol levels and elevated high-sensitivity C-reactive protein levels in apparently healthy Korean adults | A |
| 908 | Surtees, P. G.; Wainwright, N. W. J.; Luben, R.; Day, N. E.; Khaw, K. T. | 2005 | Prospective cohort study of hostility and the risk of cardiovascular disease mortality | A |
| 909 | Suzuki, K.; Iwata, A.; Ooike, Y.; Nakao, T.; Kodashima, S.; Aizawa, K.; Mizuno, Y.; Suzuki, T.; Yamazaki, T.; Tsuji, S. | 2011 | Association between cognitive deterioration and lifestyle-related diseases-study on normal populations attending comprehensive medical checkups | A |
| 910 | Suzuki, M.; Banno, K.; Usui, T.; Funasaka, N.; Segawa, T.; Kirihata, T.; Kamisako, H.; Ueda, K.; Munakata, A. | 2018 | Seasonal changes in plasma levels of thyroid hormones and the effects of the hormones on cellular ATP content in common bottlenose dolphin | A |
| 911 | Suzuki, T.; Kim, H.; Yoshida, H.; Ishizaki, T. | 2004 | Randomized controlled trial of exercise intervention for the prevention of falls in community-dwelling elderly Japanese women | A |
| 912 | Suzuki, T.; Yoshida, H.; Yoshida, S.; Nonaka, K. | 2010 | Association of quantitative calcaneal ultrasound with long-term care service utilization in elderly women: A cross-sectional population-based study | A |
| 913 | Swaminathan, A.; Sambandam, R.; Bhaskaran, M. | 2011 | Evaluation of the auditory effects of hyperlipidaemia and diabetes mellitus by using audiometry | A |
| 914 | Swaminathan, K.; Sabapathy Prakash, B. | 2013 | Diabetes in Madurai, South India: A pilot study to assess disease burden | A |
| 915 | Tai, T. S.; Hsu, C. C.; Pai, H. C.; Liu, W. H.; Hsu, Y. H. | 2013 | The association between hyperuricemia and betel nut chewing in Taiwanese men: a cross-sectional study | A |
| 916 | Tajfard, M.; , Mobarhan, M. G.; , Rahimi, H. R.; , Mouhebati, M.; , Esmaeily, H.; , Ferns, G. A.; , Latiff, L. A.; , Taghipour, A.; , Mokhber, N.; and Abdul-Aziz, A. F. | 2014 | Anxiety, Depression, Coronary Artery Disease and Diabetes Mellitus; An Association Study in Ghaem Hospital, Iran | A |

| | | | | |
|---|---|---|---|---|
| 917 | Tajirika-Shirai, R.; Takimoto, H.; Yokoyama, T.; Kaneko, H.; Kubota, T.; Miyasaka, N. | 2018 | Effect of individualised dietary education at medical check-ups on maternal and fetal outcomes in pregnant Japanese women | A |
| 918 | Takada, H.; Odaira, K.; Hashimoto, S.; Konagaya, M. | 2014 | Actual condition survey for solitudinous patients with subacutemyelo-opticoneuropathy in Japan | A |
| 919 | Takahashi, M.; Suzuki, K.; Shirai, K. | 2015 | An increase of cardio-ankle vascular index/year was the risk of the onset of ischemic changes in ECG in the urban Japanese residents | A |
| 920 | Takahashi, T.; Watanabe, T.; Shishido, T.; Sugai, T.; Taku, T.; Yokoyama, M.; Kinoshita, D.; Nishiyama, S.; Takahashi, H.; Arimoto, T.; Miyamoto, T.; Kubota, I. | 2016 | The impact of serum iron levels on cardiovascular mortality in general population | A |
| 921 | Takashima, S.; , Usui, S.; , Kurokawa, K.; , Kitano, T.; , Kato, T.; , Murai, H.; , Furusho, H.; , Oda, H.; , Maruyama, M.; , Nagata, Y.; , Usuda, K.; , Kubota, K.; , Takeshita, Y.; , Sakai, Y.; , Honda, M.; , Kaneko, S.; and Takamura, M. | 2016 | Altered gene expression in T-cell receptor signalling in peripheral blood leucocytes in acute coronary syndrome predicts secondary coronary events | A |
| 922 | Takayama, K.; , Kaneko, H.; , Ito, Y.; , Kataoka, K.; , Iwase, T.; , Yasuma, T.; , Matsuura, T.; , Tsunekawa, T.; , Shimizu, H.; , Suzumura, A.; , Ra, E.; , Akahori, T.; and Terasaki, H. | 2018 | Novel Classification of Early-stage Systemic Hypertensive Changes in Human Retina Based on OCTA Measurement of Choriocapillaris | A |
| 923 | Takayama, K.; Ito, Y.; Kaneko, H.; Kataoka, K.; Iwase, T.; Tsunekawa, T.; Shimizu, H.; Suzumura, A.; Akahori, T.; Terasaki, H. | 2018 | Optical coherence tomography angiography of choriocapillaris: Normative macular choriocapillaris vasculature and novel classification of early-stage systemic hypertensive changes: A cross-sectional study | A |
| 924 | Takyu, H.; Iwamoto, I.; Imai, I. | 2009 | Can displaying blood transfusion information help increase blood donor participation? | A |
| 925 | Tam, V. V.; , Larsson, M.; , Pharris, A.; , Diedrichs, B.; , Nguyen, H. P.; , Chuc, T. K. N.; , Ho, P. D.; , Marrone, G.; and Thorson, A. | 2012 | Peer support and improved quality of life among persons living with HIV on antiretroviral treatment: A randomised controlled trial from north-eastern Vietnam | A |
| 926 | Tamakoshi, A.; Kawamura, T.; Wakai, K.; Ando, M. | 2008 | Written informed consent for participation in a study and reduction in consent rate | A |
| 927 | Tang, X.; and Liu, Q. | 2018 | Prediction of the development of metabolic syndrome by the Markov model based on a longitudinal study in Dalian City | A |
| 928 | Taniguchi, N.; Ohsawa, N.; Fukuda, Y.; Akutagawa, S.; Gotou, I.; Min, K. Y.; Hanafusa, T. | 2003 | The role of general practitioners in stepwise notification - From anxiety test results of the chest x-rays abnormality patients | A |
| 929 | Tao, L. X.; Yang, K.; Liu, X. T.; Cao, K.; Zhu, H. P.; Luo, Y. X.; Guo, J.; Wu, L. J.; Li, X.; Guo, X. H. | 2016 | Longitudinal associations between triglycerides and metabolic syndrome components in a Beijing adult population, 2007-2012 | A |
| 930 | Tenenbaum, A.; , Sayada, M.; and Azogui-Levy, S. | 2017 | An illustrated guide to dental screening: a school survey | A |
| 931 | Terada, K.; , Satonaka, A.; , Wada, M.; , Terada, Y.; and Suzuki, N. | 2018 | Nutritional aspects of year-long wheelchair dance intervention in bedridden individuals with severe athetospastic cerebral palsy rated to GMFCS level V | A |

| | | | | |
|---|---|---|---|---|
| 932 | Terada, K.; Satonaka, A.; Wada, M.; Terada, Y.; Suzuki, N. | 2018 | Nutritional aspects of a year-long wheelchair dance intervention in bedridden individuals with severe athetospastic cerebral palsy rated to GMFCS level v | A |
| 933 | Thaduangta, B.; , Choomjit, P.; , Mongkolveswith, S.; , Supasitthimethee, U.; , Funilkul, S.; , Triyason, T.; and Ieee | 2016 | Smart Healthcare: Basic Health Check-up and Monitoring System for Elderly | A |
| 934 | Thomas, S. L.; Boreland, F.; Lyle, D. M. | 2013 | Improving participation by Aboriginal children in blood lead screening services in Broken Hill, NSW | A |
| 935 | Thompson, C. N.; , Anders, K. L.; , Nhi, L. T. Q.; , Tuyen, H. T.; , Minh, P. V.; , Tu, L. T. P.; , Nhu, T. D. H.; , Nhan, N. T. T.; , Ly, T. T. T.; , Duong, V. T.; , Vi, L. L.; , Thuy, N. T. V.; , Hieu, N. T.; , Chau, N. V. V.; , Campbell, J. I.; , Thwaites, G.; , Simmons, C.; and Baker, S. | 2014 | A cohort study to define the age-specific incidence and risk factors of Shigella diarrhoeal infections in Vietnamese children: a study protocol | A |
| 936 | Thomsen, J. L.; Parner, E. T. | 2006 | Methods for analysing recurrent events in health care data. Examples from admissions in Ebeltoft Health Promotion Project | A |
| 937 | Thu, W. P. P.; Tng, H. Y.; Logan, S.; Aris, I. M.; Cauley, J. A.; Yong, E. L. | 2017 | Sleep apnea and bone health in mid-life women | A |
| 938 | Tisi, S.; Dickson, J.; Horst, C.; Hall, H.; Mullin, A.; Farrelly, L.; Gyertson, K.; Levermore, C.; Clarke, C.; Allen, B.; Hamilton, S.; Hartmann, A.; Nair, A.; Devaraj, A.; Hackshaw, A.; Janes, S. | 2020 | SUMMIT study: protocolised management of pulmonary incidental findings in a lung cancer screening cohort | A |
| 939 | Tizek, L.; , Schielein, M.; , Spinner, C. D.; , Watzele, R.; , Kratzer, P.; , Böhner, A.; , Seifert, F.; , Biedermann, T.; and Zink, A. | 2019 | [New perspectives on health prevention. Prevalence of hypertension, hypacusis and balance disorders at the Munich Oktoberfest 2016] | A |
| 940 | Tizek, L.; , Schielein, M. C.; , Seifert, F.; , Biedermann, T.; , Bohner, A.; and Zink, A. | 2019 | Skin diseases are more common than we think: screening results of an unreferred population at the Munich Oktoberfest | A |
| 941 | Toda, H.; Nomura, S.; Gilmour, S.; Tsubokura, M.; Oikawa, T.; Lee, K.; Kiyabu, G. Y.; Shibuya, K. | 2017 | Assessment of medium-term cardiovascular disease risk after Japan's 2011 Fukushima Daiichi nuclear accident: A retrospective analysis | A |
| 942 | Todoroki, M.; , Minami, J.; , Ishimitsu, T.; , Ohrui, M.; and Matsuoka, H. | 2003 | Relation between the angiotensin-converting enzyme insertion/deletion polymorphism and blood pressure in Japanese male subjects | A |
| 943 | Tominaga, R.; , Fukuma, S.; , Yamazaki, S.; , Sekiguchi, M.; , Otani, K.; , Kikuchi, S.; , Sasaki, S.; , Kobayashi, S.; , Fukuhara, S.; and Konno, S. | 2016 | Relationship Between Kyphotic Posture and Falls in Community-Dwelling Men and Women The Locomotive Syndrome and Health Outcome in Aizu Cohort Study | A |
| 944 | Tomlin, A. M.; Dovey, S. M.; Tilyard, M. W. | 2013 | Patient outcomes from 10 years of annual diabetes reviews in New Zealand | A |
| 945 | Tong, S. F.; , Low, W. Y.; , Ismail, S. B.; , Trevena, L.; and Wilcock, S. | 2013 | Determinants of doctors' decisions to inquire about sexual dysfunction in Malaysian primary care settings | A |

| | | | | |
|---|---|---|---|---|
| 946 | Tong, S. F.; Khoo, E. M.; Low, W. Y.; Lee, B. C.; Lee, V. K. M.; Ng, C. J.; Zukifli, Z.; Tan, H. M. | 2010 | The health seeking behaviour of men with erectile dysfunction: Results of the subang aging male (SAM) study | A |
| 947 | Torniainen-Holm, M.; Pankakoski, M.; Lehto, T.; Saarelma, O.; Mustonen, P.; Joutsenniemi, K.; Suvisaari, J. | 2016 | The effectiveness of email-based exercises in promoting psychological wellbeing and healthy lifestyle: a two-year follow-up study | A |
| 948 | Tran, C. T.; Pham, T. H.; Tran, K. T.; Nguyen, T. K. C.; Larsson, M. | 2017 | Caretakers' barriers to pediatric antiretroviral therapy adherence in Vietnam - A qualitative and quantitative study | A |
| 949 | Tran-Duy, A.; McDermott, R.; Knight, J.; Hua, X.; Barr, E. L. M.; Arabena, K.; Palmer, A.; Clarke, P. M. | 2020 | Development and Use of Prediction Models for Classification of Cardiovascular Risk of Remote Indigenous Australians | A |
| 950 | Trullàs, J. C.; Roca, E.; Guillermo, A.; Bové, L.; Gibert, J. | 2018 | Ultra Pirineu 2017: Characteristics of elite and non-elite runners and effects on health of a mountain marathon: Serialmed-UP pilot study | A |
| 951 | Tseng, P. H.; , Lee, P. L.; , Hsu, W. C.; , Ma, Y.; , Lee, Y. C.; , Chiu, H. M.; , Ho, Y. L.; , Chen, M. F.; , Wu, M. S.; and Peng, C. K. | 2017 | A Higher Proportion of Metabolic Syndrome in Chinese Subjects with Sleep-Disordered Breathing: A Case-Control Study Based on Electrocardiogram-Derived Sleep Analysis | A |
| 952 | Tsuda, H.; Ito, Y. M.; Todo, Y.; Iba, T.; Tasaka, K.; Sutou, Y.; Hirai, K.; Dozono, K.; Dobashi, Y.; Manabe, M.; Sakamoto, T.; Yamamoto, R.; Ueda, K.; Akatsuka, M.; Kiyozuka, Y.; Nagai, N.; Imai, M.; Kobiki, K.; Fujita, H.; Itamochi, H.; Oshita, T.; Kawarada, T.; Hatae, M.; Yokoyama, Y. | 2018 | Measurement of endometrial thickness in premenopausal women in office gynecology | A |
| 953 | Tsugane, S.; Tsubono, Y.; Okubo, S.; Hayashi, M.; Kakizoe, T. | 1996 | A pilot study for a randomized controlled trial to prevent gastric cancer in high-risk Japanese population: Study design and feasibility evaluation | A |
| 954 | Tsukinoki, R.; Morimoto, K.; Nakayama, K. | 2005 | Association between lifestyle factors and plasma adiponectin levels in Japanese men | A |
| 955 | Tsukuya, G.; , Matsumoto, K.; , Fukuyama, S.; , Crawford, B.; , Nakanishi, Y.; , Ichinose, M.; , Machida, K.; , Samukawa, T.; , Ninomiya, T.; , Kiyohara, Y.; , Inoue, H.; and Hisayama Pulm Physiology Study, Grp | 2015 | Validation of a COPD screening questionnaire and establishment of diagnostic cut-points in a Japanese general population: The Hisayama study | A |
| 956 | Tsuruya, K.; Yoshida, H.; Nagata, M.; Kitazono, T.; Hirakata, H.; Iseki, K.; Moriyama, T.; Yamagata, K.; Yoshida, H.; Fujimoto, S.; Asahi, K.; Kurahashi, I.; Ohashi, Y.; Watanabe, T. | 2014 | Association of the triglycerides to high-density lipoprotein cholesterol ratio with the risk of chronic kidney disease: Analysis in a large Japanese population | A |
| 957 | Tzilos Wernette, G.; Plegue, M.; Mmeje, O.; Sen, A.; Countryman, K.; Ngo, Q.; Prosser, L.; Zlotnick, C. | 2019 | Reducing sexual health risks and substance use in the prenatal setting: A study protocol for a randomized controlled trial | A |
| 958 | Uchil, D.; Pipalia, D.; Chawla, M.; Patel, R.; Maniar, S.; Narayani,; Juneja, A. | 2009 | Non-alcoholic fatty liver disease (NAFLD)--the hepatic component of metabolic syndrome | A |

| | | | | |
|---|---|---|---|---|
| 959 | Ueda, K.; Kashiba, A.; Miyai, N.; Mure, K.; Arita, M. | 2017 | Effects of a home blood pressure monitoring by mobile phone-based selfmanagement support system in mild hypertension: The Katsuragi study | A |
| 960 | Ukawa, S.; Tamakoshi, A.; Tsushita, K.; Wakai, K.; Ando, M.; Ohira, H.; Okabayashi, S.; Matsushita, M.; Zhao, W.; Oikawa, J.; Kawamura, T. | 2014 | Body mass index is a predictor for developing hypertension among the younger Japanese elderly: Findings from the new integrated suburban seniority investigation project | A |
| 961 | Ukawa, S.; Tamakoshi, A.; Wakai, K.; Ando, M.; Kawamura, T. | 2015 | Body mass index is associated with hypertension in Japanese young elderly individuals: Findings of the new integrated suburban seniority investigation | A |
| 962 | Umaipalan, A.; Creighton, S.; Millett, D. | 2011 | Outreach HIV testing | A |
| 963 | Unger, M. D.; , Cuppari, L.; , Titan, S. M.; , Magalhaes, M. C. T.; , Sassaki, A. L.; , dos Reis, L. M.; , Jorgetti, V.; and Moyses, R. M. A. | 2010 | Vitamin D status in a sunny country: Where has the sun gone? | A |
| 964 | Ura, C.; Okamura, T.; Inagaki, H.; Ogawa, M.; Niikawa, H.; Edahiro, A.; Sugiyama, M.; Miyamae, F.; Sakuma, N.; Furuta, K.; Hatakeyama, A.; Ogisawa, F.; Konno, M.; Suzuki, T.; Awata, S. | 2020 | Characteristics of detected and undetected dementia among community-dwelling older people in Metropolitan Tokyo | A |
| 965 | Usfar, A.; Catharina, M. | 2017 | The need of food subsidy programs to fulfill nutrient adequacies: The case of Indonesia | A |
| 966 | Usui, T.; Kanda, E.; Iseki, C.; Iseki, K.; Kashihara, N.; Nangaku, M. | 2018 | Observation period for changes in proteinuria and risk prediction of end-stage renal disease in general population | A |
| 967 | Vagholkar, S.; Zwar, N.; Jayasinghe, U. W.; Denney-Wilson, E.; Patel, A.; Campbell, T.; Harris, M. F. | 2014 | Influence of cardiovascular absolute risk assessment on prescribing of antihypertensive and lipid-lowering medications: A cluster randomized controlled trial | A |
| 968 | Vaillo, Y. A.; Galdón Garrido, Ma J.; Durá Ferrandis, E.; Carretero Gómez, S.; Hernández, J. T. | 2004 | Age, Health Beliefs and Breast Cancer Screening Program Participation in Valencian Community, Spain | A |
| 969 | Valačić, V. B.; Tkalčić, M.; Smojver-Ažić, S.; Živčić-Bećirević, I. | 2000 | Family asthma program: Medical and psychological approach to asthmatic children and their parents | A |
| 970 | Valappil, A. V.; Chaudhary, N. V.; Praveenkumar, R.; Gopalakrishnan, B.; Girija, A. S. | 2012 | Low cholesterol as a risk factor for primary intracerebral hemorrhage: A case-control study | A |
| 971 | Valero, C.; Olmos, J. M.; Martínez, J.; Hernández, J. L.; Castillo, J.; Riancho, J. A.; Macías, J. G. | 2011 | Vitamin D variations over a four year period in a general population | A |
| 972 | Valla, L.; Wentzel-Larsen, T.; Hofoss, D.; Slinning, K. | 2015 | Prevalence of suspected developmental delays in early infancy: Results from a regional population-based longitudinal study | A |
| 973 | Vallianou, N. G.; Georgousopoulou, E.; Evangelopoulos, A. A.; Bountziouka, V.; Bonou, M. S.; Vogiatzakis, E. D.; Avgerinos, P. C.; Barbetseas, J.; Panagiotakos, D. B. | 2017 | Inverse Relationship between Adherence to the Mediterranean Diet and Serum Cystatin C Levels | A |

| | | | | |
|---|---|---|---|---|
| 974 | van Bon-Martens, M. J.; , Klingenberg, H.; , Dijkstra, H. A.; and Peeters, P. H. | 2001 | [Breast complaints during the Dutch nationwide breast cancer screening program: increased risk of referral and of breast cancer] | A |
| 975 | Van de Poppe, D. J.; , Hulzebos, E.; , Takken, T.; and Low-Land Fitness Registry Study, Gr | 2019 | Reference values for maximum work rate in apparently healthy Dutch/Flemish adults: data from the LowLands fitness registry | A |
| 976 | van der Put, A. C.; and van der Lippe, T. | 2020 | Work Environment and Worksite Health Promotion in Nine European Countries | A |
| 977 | Van Rhenen, W.; , Blonk, R. W.; , van der Klink, J. J.; , van Dijk, F. J.; and Schaufeli, W. B. | 2005 | The effect of a cognitive and a physical stress-reducing programme on psychological complaints | A |
| 978 | Van Tam, V.; , Larsson, M.; , Pharris, A.; , Diedrichs, B.; , Nguyen, H. P.; , Nguyen, C. T.; , Ho, P. D.; , Marrone, G.; and Thorson, A. | 2012 | Peer support and improved quality of life among persons living with HIV on antiretroviral treatment: a randomised controlled trial from north-eastern Vietnam | A |
| 979 | Vanhala, M.; , Kumpula, L. S.; , Soininen, P.; , Kangas, A. J.; , Ala-Korpela, M.; , Kautiainen, H.; , Mantyselka, P.; and Saltevo, J. | 2011 | High serum adiponectin is associated with favorable lipoprotein subclass profile in 6.4-year follow-up | A |
| 980 | Vargas, L. A. S.; Garces, M. R. E. A.; Dupont, P. T.; Meixueiro, A.; Jacome, A. A. A.; Roesch, F. B.; Cid, H. V.; Troche, J. M. R. | 2013 | Prevalence of antibodies related to celiac disease (CD) in patients with irritable bowel syndrome (IBS) according to rome iii criteria. A case-control study | A |
| 981 | Vatakencherry, R. M. J.; Saraswathy, L. | 2016 | Vitamin D-predictor of cardiovascular disease risk factor | A |
| 982 | Veale, M.; Ajwani, S.; Johnson, M.; Nash, L.; Patterson, T.; George, A. | 2016 | The early childhood oral health program: a qualitative study of the perceptions of child and family health nurses in South Western Sydney, Australia | A |
| 983 | Veljkovic, D.; Serbic-Nonkovic, O.; Radonjic, Z.; Stevanovic, V.; Erceg, M.; Nikolic, L.; Martic, J.; Redzic, D.; Aleksic, M.; Jovanovic, A.; Plavanski, L.; Mutavdzic, M.; Milanovic, O. | 2013 | Patients blood management in pediatric hospital | A |
| 984 | Viitasalo, K.; , Hemio, K.; , Puttonen, S.; , Hyvarinen, H. K.; , Leiviska, J.; , Harma, M.; , Peltonen, M.; and Lindstrom, J. | 2015 | Prevention of diabetes and cardiovascular diseases in occupational health care: Feasibility and effectiveness | A |
| 985 | Viitasalo, K.; , Lindstrom, J.; , Hemio, K.; , Puttonen, S.; , Koho, A.; , Harma, M.; and Peltonen, M. | 2012 | Occupational health care identifies risk for type 2 diabetes and cardiovascular disease | A |
| 986 | Villadsen, S. F.; Negussie, D.; GebreMariam, A.; Tilahun, A.; Girma, T.; Friis, H.; Rasch, V. | 2016 | Antenatal care strengthening for improved health behaviours in Jimma, Ethiopia, 2009-2011: An effectiveness study | A |
| 987 | Vincelet, C.; , Bruckert, E.; , Le Corff, J.; , Boisson, M.; and Foucault, C. | 2004 | [The interest of cholesterol levels in young children. Study in a population of 4,697 children aged 4] | A |
| 988 | Vinker, S.; Kaplan, B.; Nakar, S.; Samuels, G.; Shapira, G.; Kitai, E. | 2001 | Urinary incontinence in women: Prevalence, characteristics and effect on quality of life. A primary care clinic study | A |
| 989 | Vistisen, D.; , Witte, D. R.; , Tabak, A. G.; , Herder, C.; , Brunner, E. J.; , Kivimaki, M.; and Faerch, K. | 2014 | Patterns of Obesity Development before the Diagnosis of Type 2 Diabetes: The Whitehall II Cohort Study | A |

| | | | | |
|---|---|---|---|---|
| 990 | Vuylsteke, B.; , Vandenbruaene, M.; , Vandenbalcke, P.; , Van Dyck, E.; and Laga, M. | 1999 | Chlamydia trachomatis prevalence and sexual behaviour among female adolescents in Belgium | A |
| 991 | Wada, K.; , Tanaka, T.; , Kumagai, G.; , Kudo, H.; , Asari, T.; , Chiba, D.; , Ota, S.; , Kamei, K.; , Takeda, O.; , Nakaji, S.; and Ishibashi, Y. | 2018 | A study of the factors associated with cervical spinal disc degeneration, with a focus on bone metabolism and amino acids, in the Japanese population: a cross sectional study | A |
| 992 | Wada, T.; , Matsumoto, H.; and Hagino, H. | 2019 | Customized exercise programs implemented by physical therapists improve exercise-related self-efficacy and promote behavioral changes in elderly individuals without regular exercise: a randomized controlled trial | A |
| 993 | Wainwright, Nicholas W. J.; , Levy, Sheldon; , Pico, Jose; , Luben, Robert N.; , Surtees, Paul G.; and Khaw, Kay-Tee | 2014 | Social adversity experience and blood pressure control following antihypertensive medication use in a community sample of older adults | A |
| 994 | Wakasugi, M.; Narita, I.; Iseki, K.; Moriyama, T.; Yamagata, K.; Tsuruya, K.; Yoshida, H.; Fujimoto, S.; Asahi, K.; Kurahashi, I.; Ohashi, Y.; Watanabe, T. | 2012 | Weight gain after 20 years of age is associated with prevalence of chronic kidney disease | A |
| 995 | Waku, S.; Iida, N.; Ishihara, T. | 2000 | Significance of brain natriuretic peptide measurement as a diagnostic indicator of cardiac function | A |
| 996 | Walden, C.; Kronstadt, D.; Martin, L. | 2012 | Pink rats stay: Unique aspects of a hands-on training program designed to build confidence and proficiency | A |
| 997 | Waldmann, E.; Heinze, G.; Britto-Arias, M.; Sallinger, D.; Gessl, I.; Ferlitsch, A.; Trauner, M.; Ferlitsch, M. | 2014 | Lifestyle, environment or gender-what has bigger impact on the incidence of colorectal Neoplasia? | A |
| 998 | Walsh, N.; Currid, M. | 2018 | Breaking boundaries; where intellectual disability (ID) & Older Person Services collaborate | A |
| 999 | Walton, S.; and Bedford, H. | 2017 | Immunization of looked-after children and young people: A review of the literature | A |
| 1000 | Wam, E. C.; Sama, L. F.; Ali, I. M.; Ebile, W. A.; Aghangu, L. A.; Tume, C. B. | 2016 | Seroprevalence of Toxoplasma gondii IgG and IgM antibodies and associated risk factors in women of child-bearing age in Njinikom, NW Cameroon | A |
| 1001 | Wändell, Per E.; , de Waard, Anne-Karien M.; , Holzmann, Martin J.; , Gornitzki, Carl; , Lionis, Christos; , de Wit, Niek; , Søndergaard, Jens; , Sønderlund, Anders L.; , Kral, Norbert; , Seifert, Bohumil; , Korevaar, Joke C.; , Schellevis, François G.; and Carlsson, Axel C. | 2018 | Barriers and facilitators among health professionals in primary care to prevention of cardiometabolic diseases: A systematic review | A |
| 1002 | Wang, J. H. Y.; Ma, G. X.; Liang, W.; Gehan, E.; Tan, Y.; MaKambi, K.; Wang, Y.; Vernon, S. W.; Tu, S. P.; Mandelblatt, J. | 2015 | Effects of a culturally targeted physician communication intervention on colorectal cancer screening among older Chinese American patients | A |
| 1003 | Wang, Y. L.; Xia, K.; Zhang, J.; Wang, X. N.; Guo, F. | 2005 | Early evaluation of exercise tolerance test on diastolic functional changes of left ventricle in patients with type 2 diabetes | A |

| | | | | |
|---|---|---|---|---|
| 1004 | Wang, Y. Q.; Yang, P. T.; Yuan, H.; Cao, X.; Zhu, X. L.; Xu, G.; Mo, Z. H.; Chen, Z. H. | 2015 | Low bone mineral density is associated with increased arterial stiffness in participants of a health records based study | A |
| 1005 | Wang, Y. T.; Tseng, P. H.; Chen, C. L.; Han, D. S.; Chi, Y. C.; Tseng, F. Y.; Yang, W. S. | 2017 | Human serum RNase-L level is inversely associated with metabolic syndrome and age | A |
| 1006 | Wardhani, V.; Holipah, H.; Dewanto, A.; Triastuti, E. | 2014 | A community empowerment model to promote positive health behavior and diabetes mellitus self care | A |
| 1007 | Watanabe, Kazuhiro; , Otsuka, Yasumasa; , Inoue, Akiomi; , Sakurai, Kenji; , Ui, Akiko;  and Nakata, Akinori | 2016 | Interrelationships between job resources, vigor, exercise habit, and serum lipids in Japanese employees: A multiple group path analysis using medical checkup data | A |
| 1008 | Weber, C. | 2011 | [Diagnostics and therapy of localized prostate cancer: a problem-oriented account] | A |
| 1009 | Weidmann, C.; Schneider, S.; Weck, E.; Klüter, H. | 2010 | Are altruistically motivated first-time donors more likely to come back? Initial motivation and return of German first-time donors | A |
| 1010 | Wernette, G. T.; , Plegue, M.; , Mmeje, O.; , Sen, A.; , Countryman, K.; , Ngo, Q.; , Prosser, L.;  and Zlotnick, C. | 2019 | Reducing sexual health risks and substance use in the prenatal setting: A study protocol for a randomized controlled trial | A |
| 1011 | Wessels, M.; Te Lintelo, M.; Te Velde, R.; Putter, H.; Vriezinga, S.; Hopman, E.; Mearin, L. | 2016 | Measuring diet compliance in coeliac children and adolescents: Is there an easier way than the dietary interview? | A |
| 1012 | Westergren, T.; Berntsen, S.; Nilsen, T.; Haraldstad, K.; Kittang, O. B.; Fegran, L. | 2014 | How do children with asthma interact with parents about medications? | A |
| 1013 | Whicher, C. A.; , O'Neill, S.;  and Holt, R. I. G. | 2020 | Diabetes in the UK: 2019 | A |
| 1014 | Wichstrom, L.; , Berg-Nielsen, T. S.; , Angold, A.; , Egger, H. L.; , Solheim, E.;  and Sveen, T. H. | 2012 | Prevalence of psychiatric disorders in preschoolers | A |
| 1015 | Widdup, J.; Comino, E. J.; Webster, V.; Knight, J. | 2012 | Universal for whom? Evaluating an urban Aboriginal population's access to a mainstream universal health home visiting program | A |
| 1016 | Wienzek-Lischka, S.; Toepel, G.; Hackstein, H.; Bein, G. | 2012 | Motivation for blood donation - How can the willingness to donate blood be increased? Representative survey of a university blood donation service | A |
| 1017 | Wildin, H. | 2013 | Clinical outcomes of depot and well being clinic-results of physical health checks | A |
| 1018 | Witty, K.; White, A. K. | 2010 | 'Tackling men's health'-establishing a men's health intervention within a large sports stadium | A |
| 1019 | Wolf, G.; Pieper, R.; Obe, G. | 1999 | Chromosomal alterations in peripheral lymphocytes of female cabin attendants | A |
| 1020 | Wongkrajang, P.; Chinswangwatanakul, W.; Mokkhamakkun, C.; Chuangsuwanich, N.; Wesarachkitti, B.; Thaowto, B.; Laiwejpithaya, S.; Komkhum, O. | 2018 | Establishment of new complete blood count reference values for healthy Thai adults | A |

| | | | | |
|---|---|---|---|---|
| 1021 | Wood, D. A.; , Kinmonth, A. L.; , Davies, G.; , Yarwood, J.; , Thompson, S. G.; , Pyke, S. D. M.; , Kok, Y.; , Cramb, R.; , Leguen, C.; , Coles, C.; , Durrington, P.; , Marteau, T.; , Odwyer, A.; , Steele, D. W.; and Bicknell, C. | 1994 | BRITISH FAMILY HEART-STUDY - ITS DESIGN AND METHOD, AND PREVALENCE OF CARDIOVASCULAR RISK-FACTORS | A |
| 1022 | Wray, David; and Medwell, Jane | 2015 | Exploring a national book-gifting scheme: Parents' and children's reactions | A |
| 1023 | Wu, B. | 2007 | Dental service utilization among urban and rural older adults in China - A brief communication | A |
| 1024 | Wu, D. M.; Pai, L.; Sung, P. K.; Tsai, J. T.; Hsu, L. L.; Lee, M. C.; Sun, C. A. | 1999 | A preliminary study on the individual aggregation in cigarette smoking, alcohol drinking, and betel-nut chewing in a health check-up population | A |
| 1025 | Wu, F.; , Narimatsu, H.; , Li, X.; , Nakamura, S.; , Sho, R.; , Zhao, G.; , Nakata, Y.; and Xu, W. | 2017 | Non-communicable diseases control in China and Japan | A |
| 1026 | Wu, H. Y.; Hsu, S. P.; Peng, Y. S.; Chien, K. L.; Hung, K. Y. | 2018 | Influences of alcohol consumption on incident chronic kidney disease and proteinuria | A |
| 1027 | Wu, H. Y.; Lin, H. J.; Chen, M. R.; Peng, Y. S.; Tu, Y. K.; Hung, K. Y.; Chien, K. L. | 2016 | Effects of alcohol consumption on chronic kidney disease: A seven year cohort study | A |
| 1028 | Wu, P.; Chen, Q.; Chen, L.; Zhang, P.; Xiao, J.; Chen, X.; Liu, M.; Wang, S. | 2017 | Dose-response relationship between alanine aminotransferase levels within the reference interval and metabolic syndrome in Chinese adults | A |
| 1029 | Wu, Y. W.; Tseng, P. H.; Lee, Y. C.; Wang, S. Y.; Chiu, H. M.; Tu, C. H.; Wang, H. P.; Lin, J. T.; Wu, M. S.; Yang, W. S. | 2014 | Association of esophageal inflammation, obesity and gastroesophageal reflux disease: From FDG PET/CT perspective | A |
| 1030 | Wuestner, A.; Luderer, Ch; Kress, W.; Heller, R.; Zierz, S.; Meyer, G.; Sackmann, R.; Hoffmann, K. | 2016 | Methods of qualitative research in the analysis of development and care in children with rare diseases | A |
| 1031 | Xu, S.; Ming, J.; Xing, Y.; Jia, A.; Cai, J.; Jing, C.; Ji, Q. | 2016 | Diabetes awareness and screening in high-risk populations in China: A nationwide, population-based survey | A |
| 1032 | Yabe, D.; Kuwata, H.; Fujiwara, Y.; Sakaguchi, M.; Moyama, S.; Makabe, N.; Murotani, K.; Asano, H.; Ito, S.; Mishima, H.; Takase, H.; Ota, N.; Seino, Y.; Hamamoto, Y.; Kurose, T.; Seino, Y. | 2019 | Dietary instructions focusing on meal-sequence and nutritional balance for prediabetes subjects: An exploratory, cluster-randomized, prospective, open-label, clinical trial | A |
| 1033 | Yakov, T.; Sulayman, A.; Yahyo, Z.; Hurshid, I.; Sayde, D.; Muhabbat, A. | 2017 | Evaluation of colorectal cancer early detection status in Uzbekistan | A |
| 1034 | Yamagishi, F.; and Toyota, M. | 2009 | [Research and control of relapse tuberculosis cases] | A |
| 1035 | Yamaguchi, N.; Takahashi, T.; Ueno, T.; Hiyama, S.; Ogawa, M.; Matsumura, T.; Sasanuma, H.; Takeshita, K. | 2018 | Pain deterioration within 1 year predicts future decline of walking ability: A 7-year prospective observational study of elderly female patients with knee osteoarthritis living in a rural district | A |

| | | | | |
|---|---|---|---|---|
| 1036 | Yamakado, M.; Ichihara, K.; Matsumoto, Y.; Ishikawa, Y.; Kato, K.; Komatsubara, Y.; Takaya, N.; Tomita, S.; Kawano, R.; Takada, K.; Watanabe, K. | 2015 | Derivation of gender and age-specific reference intervals from fully normal Japanese individuals and the implications for health screening | A |
| 1037 | Yamamoto, A.; Takagishi, K.; Kobayashi, T.; Shitara, H.; Ichinose, T.; Takasawa, E.; Shimoyama, D.; Osawa, T. | 2015 | The impact of faulty posture on rotator cuff tears with and without symptoms | A |
| 1038 | Yamaoka, Y.; Fujiwara, T.; Tamiya, N. | 2016 | Association Between Maternal Postpartum Depression and Unintentional Injury Among 4-Month-Old Infants in Japan | A |
| 1039 | Yamasue, K.; Hayashi, T.; Ohshige, K.; Tochikubo, O.; Souma, T. | 2008 | Masked hypertension in elderly managerial employees and retirees | A |
| 1040 | Yanagisawa, A.; , Suzuki, K.; , Kimura, A.; , Ito, Y.; , Hamajima, N.; and Inoue, T. | 2009 | Possible protective effect of serum beta-carotene levels on the association between interleukin-1B C-31T polymorphism and hypertension in a Japanese population | A |
| 1041 | Yang, W. S.; , Chen, P. C.; , Hsu, H. C.; , Su, T. C.; , Lin, H. J.; , Chen, M. F.; , Lee, Y. T.; and Chien, I. L. | 2018 | Differential effects of saturated fatty acids on the risk of metabolic syndrome: a matched case-control and meta-analysis study | A |
| 1042 | Ying, B.; Li, D.; Zou, M.; Hu, X.; Lv, R.; Wang, L. | 2011 | Reference intervals of and factors contributing to serum Cystatin C | A |
| 1043 | Yip, J. L.; , Broadway, D. C.; , Luben, R.; , Garway-Heath, D. F.; , Hayat, S.; , Dalzell, N.; , Lee, P. S.; , Bhaniani, A.; , Wareham, N. J.; , Khaw, K. T.; and Foster, P. J. | 2011 | Physical activity and ocular perfusion pressure: the EPIC-Norfolk eye study | A |
| 1044 | Yokobayashi, Kenichi; , Kawachi, Ichiro; , Kondo, Katsunori; , Kondo, Naoki; , Nagamine, Yuiko; , Tani, Yukako; , Shirai, Kokoro; and Tazuma, Susumu | 2017 | Association between social relationship and glycemic control among older Japanese: JAGES cross-sectional study | A |
| 1045 | Yokota, F.; , Ahmed, A.; , Islam, R.; , Nishikitani, M.; , Kikuchi, K.; , Nohara, Y.; , Okajima, H.; , Kitaoka, H.; and Nakashima, N. | 2018 | The Relationships and Risk Factors Associated with Hypertension, Diabetes, and Proteinuria among Adults from Bheramara Upazila, Bangladesh: Findings from Portable Health Clinic Data, 2013-2016 | A |
| 1046 | Yokoyama, M.; Watanabe, T.; Otaki, Y.; Takahashi, H.; Arimoto, T.; Shishido, T.; Miyamoto, T.; Konta, T.; Shibata, Y.; Daimon, M.; Ueno, Y.; Kato, T.; Kayama, T.; Kubota, I. | 2016 | Association of the aspartate aminotransferase to alanine aminotransferase ratio with BNP level and cardiovascular mortality in the general population: The Yamagata study 10-year follow-up | A |
| 1047 | Yokoyama, Y.; Hakulinen, T.; Sugimoto, M.; Silventoinen, K.; Kalland, M. | 2018 | Maternal subjective well-being and preventive health care system in Japan and Finland | A |
| 1048 | Yonei, Y. | 2013 | [Significance of anti-aging medical checkups for the elderly] | A |
| 1049 | Yong, M.; Germann, C.; Lang, S.; Oberlinner, C. | 2015 | Chronotype, social jetlag, and anthropometric measures | A |
| 1050 | Yordanova, G.; Iotova, V.; Lateva, M.; Galcheva, S.; Stoycheva, R.; Mladenov, V.; Boyadjiev, V.; Bazdarska, Y.; Ivanova, A. | 2015 | One year screening program for stature deviations-strategy and outcome | A |
| 1051 | Yoshida, H.; , Nishi, M.; , Watanabe, N.; , Fujiwara, Y.; , Fukaya, T.; , Ogawa, K.; , Kim, M. J.; , Lee, S.; and Shinkai, S. | 2012 | [Predictors of frailty development in a general population of older adults in Japan using the Frailty Index for Japanese elderly patients] | A |

| | | | | |
|---|---|---|---|---|
| 1052 | Yoshida, J.; Eguchi, E.; Nagaoka, K.; Ito, T.; Ogino, K. | 2018 | Association of night eating habits with metabolic syndrome and its components: a longitudinal study | A |
| 1053 | Yoshinaga, M.; Ushinohama, H.; Sato, S.; Horigome, H.; Hata, T.; Tauchi, N.; Nishihara, E.; Sumitomo, N.; Ozawa, A.; Ichida, F.; Shiraishi, H.; Nomura, Y.; Kucho, Y.; Takahashi, H.; Ohno, S.; Nagashima, M. | 2018 | Electrocardiographic screening of 1-month-old infants to prevent sudden infant death | A |
| 1054 | Yoshino, G.; An, T.; Nakano, S.; Kuboki, K. | 2012 | Subclinical atherosclerosis in elderly subjects with metabolic syndrome | A |
| 1055 | Young, H.; , Burke, L.; and Gabhainn, S. N. | 2018 | Sexual intercourse, age of initiation and contraception among adolescents in Ireland: findings from the Health Behaviour in School-aged Children (HBSC) Ireland study | A |
| 1056 | Yu, I. W.; Wu, M. P.; Lin, H. W. | 2012 | Self-reported health status among taiwanese hypertensive elderly | A |
| 1057 | Yu, T. Y.; Jee, J. H.; Bae, J. C.; Jin, S. M.; Baek, J. H.; Lee, M. K.; Kim, J. H. | 2016 | Serum uric acid: A strong and independent predictor of metabolic syndrome after adjusting for body composition | A |
| 1058 | Yu, T. Y.; Jin, S. M.; Jee, J. H.; Bae, J. C.; Lee, M. K.; Kim, J. H. | 2019 | The protective effects of increasing serum uric acid level on development of metabolic syndrome | A |
| 1059 | Yuasa, A.; Ii, Y.; Yamamoto, Y.; Kitazaki, S.; Saito, K.; Fujimoto, Y. | 2015 | Outcome research to investigate the actual status of MRSA infection and usage of anti-MRSA drugs in real clinical settings in Japan : A retrospective database study | A |
| 1060 | Zhang, C.; Xie, C. B.; Du, Q.; Hu, H. H.; Liu, X. Q.; Long, J.; Zhong, J. L.; Jiang, W. | 2020 | Prevalence and genotype distribution of human papillomavirus from 9,182 individuals participated health examination | A |
| 1061 | Zhang, H. Y.; Youk, T. M.; Yoo, K. C.; Lee, H. K.; Song, H. J.; Yang, K. Y.; Lee, H. Y.; Cho, Y. E.; Noh, S. H.; Choi, J. B.; Nam, G. H. | 2018 | Reference temperature data of normal Korean lower extremity | A |
| 1062 | Zhang, Y. F.; , Ji, M. M.; , Zou, J. J.; , Yuan, T.; , Deng, J.; , Yang, L. N.; , Li, M. Z.; , Qin, H.; , Chen, J. H.; and Lin, Q. | 2018 | Effect of a Conditional Cash Transfer Program on Nutritional Knowledge and Food Practices among Caregivers of 3-5-Year-Old Left-Behind Children in the Rural Hunan Province | A |
| 1063 | Zhu, H.; Liang, X.; Pan, X. F.; Huang, C.; Kuang, J.; Lv, W.; Zeng, Q.; Mai, W.; Huang, Y. | 2020 | A prospective cohort study of home blood pressure monitoring based on an intelligent cloud platform (the HBPM-iCloud study): rationale and design | A |
| 1064 | Ziebland, S.; , Thorogood, M.; , Yudkin, P.; , Jones, L.; and Coulter, A. | 1998 | Lack of willpower or lack of wherewithal? "Internal" and "external" barriers to changing diet and exercise in a three year follow-up of participants in a health check | A |
| 1065 | Zohoori, F. V.; Shah, K.; Mason, J.; Shucksmith, J. | 2012 | Identifying Factors to Improve Oral Cancer Screening Uptake: A Qualitative Study | A |
| 1066 | Zouzia, E.; Kakalou, E.; Papastamopoulos, V.; Kakkavas, T.; Dimas, D.; Rosenberg, T.; Skoutelis, A. | 2016 | CVD risk assessment using various tools in an HIV cohort in Greece | A |

| | | | | |
|---|---|---|---|---|
| 1067 | Zulkiewicz, B. A.; Burrus, O.; Harshbarger, C.; Ortiz, A.; Garner, B. R.; Lewis, M. A. | 2020 | Identifying Implementation Strategies That Address Barriers and Facilitate Implementation of Digital Interventions in HIV Primary Care Settings: Results from the Pilot Implementation of Positive Health Check | A |
| 1068 | Abraham, A.; Lau, C. H.; Wong, L.; Kim, J. H. | 2013 | Health risk screening practices by providers and associated factors among university students in hong kong | B |
| 1069 | Ahmed, F.; Rao, A.; Shenoy, R.; Suprabha, B. S. | 2018 | Knowledge, attitude, and behavior of nurses toward delivery of Primary Oral Health Care in Dakshina Kannada, India | B |
| 1070 | Aida, J.; Ando, Y.; Oosaka, M.; Niimi, K.; Morita, M. | 2008 | Contributions of social context to inequality in dental caries: A multilevel analysis of Japanese 3-year-old children | B |
| 1071 | Al-Hanawi, M. K.; Mwale, M. L.; Kamninga, T. M. | 2020 | The effects of health insurance on health-seeking behaviour: Evidence from the Kingdom of Saudi Arabia | B |
| 1072 | Alexander, Karyn E.; , Brijnath, Bianca;  and Mazza, Danielle | 2013 | 'Can they really identify mental health problems at the age of three?' parent and practitioner views about screening young children’s social and emotional development | B |
| 1073 | Aljaber, A.;  and Al-Surimi, K. | 2015 | Promoting oral health practice among patients with diabetes attending primary health care clinics | B |
| 1074 | Angsuwathana, S.; , Leerasiri, P.; , Rattanachaiyanont, M.; , Tanmahasamut, P.; , Dangrat, C.; , Indhavivadhana, S.;  and Techatrisak, K. | 2007 | Health check-up program for pre/postmenopausal women at Siriraj Menopause Clinic | B |
| 1075 | Artac, M.; , Dalton, A. R.; , Babu, H.; , Bates, S.; , Millett, C.; and Majeed, A. | 2013 | Primary care and population factors associated with NHS Health Check coverage: a national cross-sectional study | B |
| 1076 | Baker, C.; Loughren, E. A.; Crone, D.; Kallfa, N. | 2015 | A process evaluation of the NHS Health Check care pathway in a primary care setting | B |
| 1077 | Baxter, H.; , Lowe, K.; , Houston, H.; , Jones, G.; , Felce, D.; and Kerr, M. | 2006 | Previously unidentified morbidity in patients with intellectual disability | B |
| 1078 | Bender, A. M.; , Jorgensen, T.;  and Pisinger, C. | 2015 | Is self-selection the main driver of positive interpretations of general health checks? The Inter99 randomized trial | B |
| 1079 | Bennett, P.; Blackall, M.; Clapham, M.; Little, S.; Player, D. | 1988 | A multi-disciplinary approach to the prevention of coronary heart disease | B |
| 1080 | Bennett, P.; Blackall, M.; Clapham, M.; Little, S.; Player, D.; Williams, K. | 1989 | South Birmingham Coronary Prevention Project: A district approach to the prevention of heart disease | B |
| 1081 | Benova, L.; Owolabi, O.; Radovich, E.; Wong, K. L. M.; Macleod, D.; Langlois, E. V.; Campbell, O. M. R. | 2019 | Provision of postpartum care to women giving birth in health facilities in sub-Saharan Africa: A cross-sectional study using Demographic and Health Survey data from 33 countries | B |
| 1082 | Bernstorff, M.; , Deichgraeber, P.; , Bruun, N. H.; , Dalsgaard, E. M.; , Fenger-Gron, M.;  and Lauritzen, T. | 2019 | A Randomised Trial Examining Cardiovascular Morbidity and All-Cause Mortality 24 years Following General Health Checks: the Ebeltoft Health Promotion Project (EHPP) | B |

| | | | | |
|---|---|---|---|---|
| 1083 | Bestges, B. B.; Lösche, C. C.; Krummenauer, F. | 2013 | How is ophthalmic undersupply demonstrated in socially disadvantaged people? Cross-sectional pilot investigation on the parametrisation of endpoints for patient-centred care research | B |
| 1084 | Bleakley, A.; , Merzel, C.; , Messeri, P.; , Gift, T.; , Malotte, C. K.; , Middlestadt, S.; and VanDevanter, N. | 2008 | Check Out That Body: A Community Awareness Campaign in New York City | B |
| 1085 | Boyle, J.; Powell, C. | 2017 | Qualitative audit of GP learning disability annual health checks for young people | B |
| 1086 | Bradshaw, S.; Hellwig, L.; Peate, D.; Wilson, A. | 2015 | Promoting the uptake of preventative Aboriginal child health policy in Western Australia | B |
| 1087 | Broholm-Jorgensen, M.; , Guassora, A. D.; , Reventlow, S.; , Dalton, S. O.; and Tjornhoj-Thomsen, T. | 2017 | Balancing trust and power: a qualitative study of GPs perceptions and strategies for retaining patients in preventive health checks | B |
| 1088 | Bünger, J.; Lanzerath, I.; Ruhnau, P.; Görlitz, A.; Fischer, C.; Kott, J.; Ellrott, T.; Flege, A.; Tschentscher, H.; Reutemann, S. K.; Meier, W.; Schwaldat, M.; Niklas, A.; Pudel, V.; Hilgers, R.; Hallier, E. | 2003 | Company health care: Evaluation of concepts for reducing cardiovascular risks | B |
| 1089 | Carey, I. M.; , Hosking, F. J.; , Harris, T.; , DeWilde, S.; , Beighton, C.; and Cook, D. G. | 2017 | Health Services and Delivery Research | B |
| 1090 | Carlos-Oliva, D.; , Vitale, M. P.; , Granana, N.; , Rouvier, M. E.; and Zeltman, C. | 2020 | Neurodevelopmental development with the use of the Ages and Stages Questionnaire (ASQ-3) in monitoring children's health | B |
| 1091 | Cavanagh, D. | 2019 | Health checks and after health checks study - A qualitative longitudinal study. Annual health checks for people with intellectual disabilities: An exploration of experiences, follow up and self management of health conditions | B |
| 1092 | Chandak, A.; Yeravdekar, R.; Shukla, S. | 2017 | Trends in students' outlook for annual health checkup at an indian university | B |
| 1093 | Chapman, Hazel M.; , Lovell, Andrew; and Bramwell, Ros | 2018 | Do health consultations for people with learning disabilities meet expectations? A narrative literature review | B |
| 1094 | Cheong, A. T.; , Liew, S. M.; , Khoo, E. M.; , Zaidi, N. F. M.; and Chinna, K. | 2017 | Are interventions to increase the uptake of screening for cardiovascular disease risk factors effective? A systematic review and meta-analysis | B |
| 1095 | Cox, Cheryl L.; , Zhu, Liang; , Finnegan, Lorna; , Steen, Brenda D.; , Hudson, Melissa M.; , Robison, Leslie L.; and Oeffinger, Kevin C. | 2012 | Survivor profiles predict health behavior intent: The Childhood Cancer Survivor Study | B |
| 1096 | Diez, M.; Picavet, P.; Ricci, R.; Dequenne, M.; Renard, M.; Bongartz, A.; Farnir, F. | 2015 | Health screening to identify opportunities to improve preventive medicine in cats and dogs | B |
| 1097 | Felce, David; , Baxter, Helen; , Lowe, Kathy; , Dunstan, Frank; , Houston, Helen; , Jones, Glyn; , Felce, Janet; and Kerr, Michael | 2008 | The impact of repeated health checks for adults with intellectual disabilities | B |

| | | | | |
|---|---|---|---|---|
| 1098 | Flitcroft, L.; Chen, W. S.; Meyer, D. | 2020 | The Demographic Representativeness and Health Outcomes of Digital Health Station Users: Longitudinal Study | B |
| 1099 | Geyti, C.; Maindal, H. T.; Dalsgaard, E. M.; Christensen, K. S.; Sandbæk, A. | 2018 | Mental health assessment in health checks of participants aged 30–49 years: A large-scale cohort study | B |
| 1100 | Gibb, S.; Milne, B.; Shackleton, N.; Taylor, B. J.; Audas, R. | 2019 | How universal are universal preschool health checks? An observational study using routine data from New Zealand's B4 School Check | B |
| 1101 | Hagen, B.; and Strauch, S. | 2011 | The J1 Adolescent Health Check-Up Analysis of Data From the German KiGGS Survey | B |
| 1102 | Hamilton, R.; Harrison, M.; Naji, S.; Robertson, C. | 2009 | Service innovation: The first year of lifestyle clinics for psychiatric out-patients | B |
| 1103 | Hock, S.; , Graul, C.; , Herb, S.; , Nötzel, G.; and Kieslich, M. | 2017 | [Failure to Attend Child Preventive Examination as a Possible Indication of Risk to Children's Welfare: Retrospective Analysis of 605 Cases Reported to the Child Protection Services] | B |
| 1104 | Hoj, K.; , Skriver, M. V.; , Hansen, A. L. S.; , Christensen, B.; , Maindal, H. T.; and Sandbaek, A. | 2014 | Effect of including fitness testing in preventive health checks on cardiorespiratory fitness and motivation: study protocol of a randomized controlled trial | B |
| 1105 | Hoj, K.; , Skriver, M. V.; , Maindal, H. T.; , Christensen, B.; and Sandbaek, A. | 2018 | The effect of cardiorespiratory fitness assessment in preventive health checks: a randomised controlled trial | B |
| 1106 | Hyseni, L.; Guzman-Castillo, M.; Kypridemos, C.; Collins, B.; Schwaller, E.; Capewell, S.; Boland, A.; Dickson, R.; O'Flaherty, M.; Lloyd-Williams, F. | 2018 | Engaging with stakeholders to inform the development of a computer model for the NHS Health Check programme: a qualitative study | B |
| 1107 | Hyseni, L.; Guzman-Castillo, M.; Kypridemos, C.; Collins, B.; Schwaller, E.; Capewell, S.; Boland, A.; Dickson, R.; O'Flaherty, M.; Gallacher, K.; Hale, P.; Lloyd-Williams, F. | 2020 | Engaging with stakeholders to inform the development of a decision-support tool for the NHS health check programme: qualitative study | B |
| 1108 | Ismail, H.; Kelly, S. | 2015 | Lessons learned from England's Health Checks Programme: using qualitative research to identify and share best practice | B |
| 1109 | Jahangard-Rafsanjani, Z.; Hakimzadeh, N.; Gholami, K. H.; Sarayani, A. | 2015 | A community pharmacy-based cardiovascular risk screening service implemented in a resource-limited country | B |
| 1110 | Jennings, W.; , Spurling, G. K.; and Askew, D. A. | 2014 | Yarning about health checks: barriers and enablers in an urban Aboriginal medical service | B |
| 1111 | Kanstrup, H.; Refsgaard, J.; Engberg, M.; Lassen, J. F.; Larsen, M. L.; Lauritzen, T. | 2002 | Cholesterol reduction following health screening in general practice | B |
| 1112 | Kapphahn, C. J.; Wilson, K. M.; Klein, J. D. | 1999 | Adolescent girls' and boys' preferences for provider gender and confidentiality in their health care | B |
| 1113 | Kilkenny, M. F.; Casburn, K.; Reyneke, M.; Johnson, R.; Lalor, E.; Cadilhac, D. | 2014 | Influence of area-level socioeconomic status and risk factors for stroke: Impact of the New South Wales (NSW) Know your numbers program | B |

| | | | | |
|---|---|---|---|---|
| 1114 | Komase, Y.; Abe, T.; Kasahara, K.; Kaneko, T.; Takahashi, H.; Nishikawa, M.; Kuwahira, I. | 2008 | Survey on the handling of chronic obstructive pulmonary disease by annual health check in Japan | B |
| 1115 | Krska, J.; du Plessis, R.; Chellaswamy, H. | 2016 | Implementation of NHS Health Checks in general practice: variation in delivery between practices and practitioners | B |
| 1116 | Landers, G.; , Snyder, A.; and Zhou, M. | 2013 | Comparing Preventive Visits of Children in Foster Care with other Children in Medicaid | B |
| 1117 | Lehtinen-Jacks, S.; Kyttälä, P.; Erkkola, M.; Lindfors, P.; Kronberg-Kippilä, C.; Lahti-Koski, M.; Hakulinen, T.; Räsänen, S. M.; Virtanen, S. M. | 2018 | Healthier diets for families with toddlers: A cluster randomized pilot trial in Finnish child health clinics | B |
| 1118 | Lennox, N.; , Ware, R.; , Carrington, S.; , O'Callaghan, M.; , Williams, G.; , McPherson, L.; and Bain, C. | 2012 | Ask: a health advocacy program for adolescents with an intellectual disability: a cluster randomised controlled trial | B |
| 1119 | Lennox, N.; Bain, C.; Carrington, S.; O'Callaghan, M.; McPherson, L.; Ware, R. | 2016 | Improving health advocacy and health outcomes in adolescents with intellectual disabilities | B |
| 1120 | Lennox, N.; McPherson, L.; Bain, C.; O'Callaghan, M.; Carrington, S.; Ware, R. S. | 2016 | A health advocacy intervention for adolescents with intellectual disability: a cluster randomized controlled trial | B |
| 1121 | Leyk, D.; , Ruther, T.; , Wunderlich, M.; , Sievert, A. P.; , Erley, O. M.; and Lollgen, H. | 2008 | Utilization and Implementation of Sports Medical Screening Examinations | B |
| 1122 | Lloyd-Williams, F.; Hyseni, L.; Guzman-Castillo, M.; Kypridemos, C.; Collins, B.; Capewell, S.; Schwaller, E.; O'Flaherty, M. | 2020 | Evaluating stakeholder involvement in building a decision support tool for NHS health checks: co-producing the WorkHORSE study | B |
| 1123 | Lo, T. Y.; Wong, C. K. H.; Lau, P. S.; Lau, C. H.; Chan, W. W. Y.; Wong, T. C. | 2014 | The usefulness of primary care physician-led motivational interview in a substance abuse check-up clinic: A retrospective study | B |
| 1124 | Macdonald, S.; Morrison, J.; Melville, C. A.; Baltzer, M.; MacArthur, L.; Cooper, S. A. | 2018 | Embedding routine health checks for adults with intellectual disabilities in primary care: practice nurse perceptions | B |
| 1125 | Magnusson, M.; , Persson, K.; and Sundelin, C. | 2001 | The effectiveness of routine health examinations at 2, 6, 9 and 12 months of age: experiences based on data from a Swedish county | B |
| 1126 | Maksimova, T. M.; Belov, V. B.; Lushkina, N. P.; Barabanova, N. A. | 2004 | A comparative evaluation of the health of children based on the results of medical check-ups and questionnaires | B |
| 1127 | Marcinkiewicz, A.; Hanke, W.; Kałużny, P.; Lipińska-Ojrzanowska, A.; Wiszniewska, M.; Walusiak-Skorupa, J. | 2018 | Can periodical examinations of employees be useful in detection of glycaemia impairment and improving patients' adherence to medical recommendations? | B |
| 1128 | Martínez Caceres, C.; Giménez Lascano, G.; Vidal, G.; Di Laudadio, S.; Fernandez, N.; Ramat, M.; Calvo, E.; Cané, P.; Lancelle, L.; Orsei, S.; Aréchaga, M.; Martínez Ulloa, J.; Pagliaro, D. | 2002 | From speech to action. About how family doctors look after the patient's health. A quantitative study | B |
| 1129 | Mc Namara, K. P.; , Krass, I.; , Peterson, G. M.; , Alzubaidi, H.; , Grenfell, R.; , Freedman, B.; and Dunbar, J. A. | 2020 | Implementing screening interventions in community pharmacy to promote interprofessional coordination of primary care - A mixed methods evaluation | B |

| | | | | |
|---|---|---|---|---|
| 1130 | McMillan, B.; , Fox, S.; , Lyons, M.; , Bourke, S.; , Mistry, M.; , Ruddock, A.; , Brown, B.; , Tang, M. Y.; and Van Marwijk, H. | 2018 | Using patient and public involvement to improve the research design and funding application for a project aimed at fostering a more collaborative approach to the NHS health check: the CaVIAR project (better Care Via Improved Access to Records) | B |
| 1131 | Mengoni, Silvana E.; and Redman, Sandra | 2019 | Health monitoring of young children with down syndrome: A parent-report study | B |
| 1132 | Mengoni, S. E.; and Redman, S. | 2020 | Health monitoring of young children with Down syndrome: A parent-report study | B |
| 1133 | Mengoni, S.; Redman, S. | 2019 | Health service provision for babies and young children with down syndrome: A parent-report study | B |
| 1134 | Metzner, F.; , Ravens-Sieberer, U.; , Schwinn, A.; , Lietz, J.; and Pawils, S. | 2015 | [Health Promotion and Child Protection in the Paediatric Practice - Paediatricians as Protagonists in an Invitation and Reporting System for Child Health Check-ups] | B |
| 1135 | Miller, M. K.; Dowd, D.; Linebarger, J.; Jahnke, S.; Wickcliffe, J. | 2014 | Accessing healthcare: Experiences of urban youth | B |
| 1136 | Nguyen, M.; Dunne, M.; Tariq, A. | 2019 | PNS183 UNIVERSAL HEALTH INSURANCE COVERAGE FOR CHILDREN UNDER AGE 6: FACTORS ASSOCIATED WITH THE USE OF PRIVATE OVER PUBLIC HEALTH SERVICES IN VIETNAM | B |
| 1137 | Nicholas, J. M.; , Burgess, C.; , Dodhia, H.; , Miller, J.; , Fuller, F.; , Cajeat, E.; and Gulliford, M. C. | 2013 | Variations in the organization and delivery of the oNHS health check' in primary care | B |
| 1138 | Nikander, K.; , Kosola, S.; , Kaila, M.; and Hermanson, E. | 2018 | Who benefit from school doctors' health checks: a prospective study of a screening method | B |
| 1139 | Norman, P. | 1993 | Predicting the uptake of health checks in general practice: Invitation methods and patients' health beliefs | B |
| 1140 | Norman, Paul; and Fitter, Mike | 1991 | Predicting attendance at health screening: Organizational factors and patients' health beliefs | B |
| 1141 | Orts, L. M.; , Lokke, A.; , Bjerregaard, A. L.; , Maindal, H. T.; , Norman, K.; , Bech, B. H.; and Sandbaek, A. | 2019 | The effect on participation rates of including focused spirometry information in a health check invitation: a cluster-randomised trial in Denmark | B |
| 1142 | Orts, L. M.; , Lokke, A.; , Bjerregaard, A. L.; , Maindal, H. T.; and Sandbaek, A. | 2016 | Effect on attendance by including focused information on spirometry in preventive health checks: study protocol for a randomized controlled trial | B |
| 1143 | Paldanius, S.; , Seilo, N.; , Kunttu, K.; , Autio, R.; and Kaila, M. | 2020 | Screening University Students for Health Checks With an Electronic Health Questionnaire in Finland: Protocol for a Retrospective, Register-Based Cohort Study | B |
| 1144 | Perry, J.; Felce, D.; Kerr, M.; Bartley, S.; Tomlinson, J.; Felce, J. | 2014 | Contact with primary care: the experience of people with intellectual disabilities | B |

| | | | | |
|---|---|---|---|---|
| 1145 | Price, H. C.; Woodman, S.; Oxborrow, L. | 2016 | NICE care processes in the housebound population living with diabetes in New Milton, Hampshire | B |
| 1146 | Qidwai, W.; , Nanji, K.; , Khoja, T. A. M.; , Rawaf, S.; , Al Kurashi, N. Y.; , Alnasir, F.; , Al Shafaee, M. A.; , Al Shetti, M.; , Bashir, M.; , Saad, N. E. S.; , Alkaisi, S.; , Halasa, W.; , Al-Duwaisan, H.; , Al-Ali, A.;  and Farahat, T. M. | 2015 | Health Promotion, Disease Prevention and Periodic Health Checks: Perceptions and Practice among Family Physicians in Eastern Mediterranean Region | B |
| 1147 | Rasmussen, S. R.; , Thomsen, J. L.; , Kilsmark, J.; , Hvenegaard, A.; , Engberg, M.; , Lauritzen, T.;  and Sogaard, J. | 2007 | Preventive health screenings and health consultations in primary care increase life expectancy without increasing costs | B |
| 1148 | Rider, G. N.; McMorris, B. J.; Gower, A. L.; Coleman, E.; Eisenberg, M. E. | 2018 | Youth and provider perspectives on improving health care experiences for transgender and gender nonconforming adolescents: A mixed methods study | B |
| 1149 | Riley, R.; Coghill, N.; Montgomery, A.; Feder, G.; Horwood, J. | 2015 | The provision of NHS health checks in a community setting: an ethnographic account | B |
| 1150 | Shaw, R. L.; Lowe, H.; Holland, C.; Pattison, H.; Cooke, R. | 2016 | GPs' perspectives on managing the NHS Health Check in primary care: A qualitative evaluation of implementation in one area of England | B |
| 1151 | Si, S.; Moss, J.; Karnon, J.; Stocks, N. | 2018 | Cost-effectiveness evaluation of the 45-49 year old health check versus usual care in Australian general practice: A modelling study | B |
| 1152 | Sondergaard, A.; , Christensen, B.;  and Maindal, H. T. | 2012 | Diversity and ambivalence in general practitioners' attitudes towards preventive health checks - a qualitative study | B |
| 1153 | Sox, Harold C. | 2013 | The health checkup: Was it ever effective? Could it be effective? | B |
| 1154 | Stol, Yrrah H.; , Asscher, Eva C. A.;  and Schermer, Maartje H. N. | 2017 | What is a good health check? An interview study of health check providers’ views and practices | B |
| 1155 | Stone, T. J.; Brangan, E.; Chappell, A.; Harrison, V.; Horwood, J. | 2020 | Telephone outreach by community workers to improve uptake of NHS Health Checks in more deprived localities and minority ethnic groups: a qualitative investigation of implementation | B |
| 1156 | Sugiyama, T.; , Tamiya, N.; , Watanabe, T.; , Wakui, T.; , Shibayama, T.; , Moriyama, Y.; , Yamaoka, Y.;  and Noguchi, H. | 2018 | Association of care recipients' care-need level with family caregiver participation in health check-ups in Japan | B |
| 1157 | Tomokawa, S.; Asakura, T.; Njenga, S. M.; Njomo, D. W.; Takeuch, R.; Akiyama, T.; Kazama, H.; Mutua, A.; Barnett, W.; Henzan, H.; Shimada, M.; Ichinose, Y.; Kamiya, Y.; Kaneko, S.; Miyake, K.; Kobayashi, J. | 2020 | Examining the appropriateness and reliability of the strategy of the Kenyan Comprehensive School Health Program | B |
| 1158 | Uronen, L.; , Heimonen, J.; , Puukka, P.; , Martimo, K. P.; , Hartiala, J.;  and Salanterä, S. | 2017 | Health check documentation of psychosocial factors using the WAI | B |
| 1159 | Usher-Smith, J. A.; , Pritchard, J.; , Poole, S.;  and Griffin, S. J. | 2015 | Offering statins to a population attending health checks with a 10-year cardiovascular disease risk between 10% and 20 | B |

| | | | | |
|---|---|---|---|---|
| 1160 | Van Der Meer, J. B. W.; Mackenbach, J. P. | 1999 | The care and course of diabetes: Differences according to level of education | B |
| 1161 | Wangdahl, J.; , Westerling, R.; , Lytsy, P.; and Martensson, L. | 2019 | Perspectives on health examination for asylum seekers in relation to health literacy - focus group discussions with Arabic and Somali speaking participants | B |
| 1162 | Ware, R. S.; Lennox, N. G. | 2016 | Characteristics influencing attendance at a primary care health check for people with intellectual disability: An individual participant data meta-analysis | B |
| 1163 | Yen, S. M.; Kung, P. T.; Tsai, W. C. | 2014 | Factors associated with free adult preventive health care utilization among physically disabled people in Taiwan: nationwide population-based study | B |
| 1164 | | 1991 | Prevalence of risk factors for heart disease in OXCHECK trial: implications for screening in primary care. Imperial Cancer Research Fund OXCHECK Study Group | C |
| 1165 | Abbas, S. Z.; Pollard, T. M.; Wynn, P.; Learmonth, A.; Joyce, K.; Bambra, C. | 2015 | The effectiveness of using the workplace to identify and address modifiable health risk factors in deprived populations | C |
| 1166 | Artac, M.; Dalton, A. R. H.; Majeed, A.; Car, J.; Huckvale, K.; Millett, C. | 2013 | Uptake of the NHS health check programme in an urban setting | C |
| 1167 | Artac, M.; Dalton, A. R. H.; Majeed, A.; Car, J.; Millett, C. | 2013 | Effectiveness of a national cardiovascular disease risk assessment program (NHS Health Check): Results after one year | C |
| 1168 | Artac, M.; Dalton, A. R. H.; Babu, H.; Bates, S.; Millett, C.; Majeed, A. | 2013 | Primary care and population factors associated with NHS Health Check coverage: A national cross-sectional study | C |
| 1169 | Attwood, S.; Morton, K.; Sutton, S. | 2016 | Exploring equity in uptake of the NHS Health Check and a nested physical activity intervention trial | C |
| 1170 | Banack, H.; Lowensteyn, I.; Grover, S.; Marchand, S.; Grover, S. | 2011 | Promoting health lifestyles in community pharmacies: The My Health Check Up program | C |
| 1171 | Bender, A. M.; , Jorgensen, T.; and Pisinger, C. | 2017 | Do high participation rates improve effects of population-based general health checks? | C |
| 1172 | Bender, A. M.; , Jorgensen, T.; and Pisinger, C. | 2019 | Higher mortality in women living in high-participation areas of a population-based health check and lifestyle intervention study | C |
| 1173 | Bender, A. M.; , Kawachi, I.; , Jorgensen, T.; and Pisinger, C. | 2015 | Neighborhood social capital is associated with participation in health checks of a general population: a multilevel analysis of a population-based lifestyle intervention- the Inter99 study | C |
| 1174 | Bender, A. M.; , Kawachi, I.; , Jorgensen, T.; and Pisinger, C. | 2015 | Neighborhood Deprivation Is Strongly Associated with Participation in a Population-Based Health Check | C |
| 1175 | Broholm-Jorgensen, M.; , Kamstrup-Larsen, N.; , Guassora, A. D.; , Reventlow, S.; , Dalton, S. O.; and Tjornhoj-Thomsen, T. | 2019 | 'It can't do any harm': A qualitative exploration of accounts of participation in preventive health checks | C |

| | | | | |
|---|---|---|---|---|
| 1176 | Broholm-Jørgensen, M.; Guassora, A. D.; Reventlow, S.; Dalton, S. O.; Tjørnhøj-Thomsen, T. | 2017 | Balancing trust and power: a qualitative study of GPs perceptions and strategies for retaining patients in preventive health checks | C |
| 1177 | Brown, K. | 1997 | Problems found in the over-75s by the annual health check | C |
| 1178 | Brunner-Ziegler, S.; Rieder, A.; Stein, K. V.; Koppensteiner, R.; Hoffmann, K.; Dorner, T. E. | 2013 | Predictors of participation in preventive health examinations in Austria | C |
| 1179 | Bukman, A. J.; , Teuscher, D.; , Ben Meftah, J.; , Groenenberg, I.; , Crone, M. R.; , van Dijk, S.; , Bos, M. B.; and Feskens, E. J. M. | 2016 | Exploring strategies to reach individuals of Turkish and Moroccan origin for health checks and lifestyle advice: a mixed-methods study | C |
| 1180 | Burgess, C. P.; Berry, H. L.; Gunthorpe, W.; Bailie, R. S. | 2008 | Development and preliminary validation of the 'caring for country' questionnaire: Measurement of an Indigenous Australian health determinant | C |
| 1181 | Chan, A.; , Amoroso, C.; and Harris, M. | 2008 | New 45-49 year health checks - GP uptake of MBS item 717 | C |
| 1182 | Chang, K. C.; , Soljak, M.; , Lee, J. T.; , Woringer, M.; , Johnston, D.; , Khunti, K.; , Majeed, A.; and Millett, C. | 2015 | Coverage of a national cardiovascular risk assessment and management programme (NHS Health Check): Retrospective database study | C |
| 1183 | Chang, K. C. M.; Lee, J. T.; Vamos, E. P.; Soljak, M.; Johnston, D.; Khunti, K.; Majeed, A.; Millett, C. | 2016 | Impact of the National Health Service Health Check on cardiovascular disease risk: A difference-in-differences matching analysis | C |
| 1184 | Chang, K. C. M.; Vamos, E. P.; Palladino, R.; Majeed, A.; Lee, J. T.; Millett, C. | 2019 | Impact of the NHS Health Check on inequalities in cardiovascular disease risk: a difference-in-differences matching analysis | C |
| 1185 | Cheong, A. T.; Khoo, E. M.; Tong, S. F.; Liew, S. M. | 2016 | To check or not to check? A qualitative study on how the public decides on health checks for cardiovascular disease prevention | C |
| 1186 | Cheong, A. T.; Khoo, E. M.; Liew, S. M.; Chinna, K. | 2018 | What are the determinants for individuals to undergo cardiovascular disease health checks? A cross sectional survey | C |
| 1187 | Chew, C. A.; , Wilkin, D.; and Glendenning, C. | 1994 | Annual assessment of patients aged 75 years and over: general practitioners' and practice nurses' views and experiences | C |
| 1188 | Chiou, C. J.; Chang, H. Y. | 2002 | Do the elderly benefit from annual physical examination? An example from Kaohsiung City, Taiwan | C |
| 1189 | Chiou, C. J.; Guo, S. E.; Chen, H. C. | 1994 | The utilization of health care by the elderly with chronic health problems in Kaohsiung City | C |
| 1190 | Chua, G. N.; Bond, C.; Ryan, M.; Porteous, T. | 2017 | Using a discrete choice experiment to value a community pharmacy service: How valid are the findings? | C |
| 1191 | Chua, G. N.; Ryan, M.; Porteous, T.; Bond, C. | 2016 | Assessing external validity of discrete choice experiments using a case study in community pharmacy: Can attitude tell us more? | C |
| 1192 | Coghill, N.; , Garside, L.; , Montgomery, A. A.; , Feder, G.; and Horwood, J. | 2018 | NHS health checks: a cross-sectional observational study on equity of uptake and outcomes | C |

| | | | | |
|---|---|---|---|---|
| 1193 | Collins, B.; Kypridemos, C.; Parvulescu, P.; Cookson, R.; Capewell, S.; O'Flaherty, M. | 2017 | Distributive equity in the real world: Would targeting the National Health Service Health Check programme to deprived groups be more cost effective? | C |
| 1194 | Coulter, A.; Fowler, G.; Jones, L.; Lawrence, M.; Mant, D.; Muir, J.; Neil, A.; Roe, L.; Rusted, N.; Schofield, T.; Thorogood, M.; Yudkin, P. | 1991 | Prevalence of risk factors for heart disease in OXCHECK trial: Implications for screening in primary care | C |
| 1195 | Dalton, A. R.; , Bottle, A.; , Okoro, C.; , Majeed, A.; and Millett, C. | 2011 | Uptake of the NHS Health Checks programme in a deprived, culturally diverse setting: cross-sectional study | C |
| 1196 | Dam, A.; , Datta, N.; , Mohanty, U. R.; , Karn, R.; , Singh, D.; and Kumar, S. | 2010 | Preventive palliation in the elderly - organizing health camps for the rural aged | C |
| 1197 | Earp, K.; Al-Joudeh, A.; Delaney, H. | 2018 | Screening for non-alcoholic fatty liver disease in primary care using simple fibrosis markers | C |
| 1198 | Edwards, L. A.; Taylor, D. J.; Campbell, P.; Shah, R.; Edgar, D. F.; Crabb, D. P. | 2019 | Feeling the pressure: A cross-sectional study exploring feasibility of a healthcare Pop-Up for intraocular pressure measurements in shopping centres in England | C |
| 1199 | Edwards, L.; Taylor, D. J.; Shah, R.; Campbell, P.; Edgar, D. F.; Crabb, D. P. | 2018 | Feeling the pressure - Initial results from a shopping centre/mall Pop-Up for screening intra-ocular pressure (IOP) across England | C |
| 1200 | Ellis, N.; , Gidlow, C.; , Cowap, L.; , Randall, J.; , Iqbal, Z.; and Kumar, J. | 2015 | A qualitative investigation of non-response in NHS health checks | C |
| 1201 | Engberg, M.; Christensen, B.; Karlsmose, B.; Lous, J.; Lauritzen, T. | 2002 | General health screenings to improve cardiovascular risk profiles: A randomized controlled trial in general practice with 5-year follow-up | C |
| 1202 | Enzell, K. | 1984 | Mortality among persons with depressive symptoms and among responders and non-responders in a health check-up. An investigation of persons born in 1905 and followed up from age 66 to 75 | C |
| 1203 | Fanaian, M.; , Laws, R. A.; , Passey, M.; , McKenzie, S.; , Wan, Q.; , Davies, G. P.; , Lyle, D.; and Harris, M. F. | 2010 | Health improvement and prevention study (HIPS) evaluation of an intervention to prevent vascular disease in general practice | C |
| 1204 | Fletcher, A. E.; , Jones, D. A.; , Bulpitt, C. J.; and Tulloch, A. J. | 2002 | The MRC trial of assessment and management of older people in the community: objectives, design and interventions ISRCTN23494848 | C |
| 1205 | Gidlow, C. J.; , Ellis, N. J.; , Cowap, L.; , Riley, V.; , Crone, D.; , Cottrell, E.; , Grogan, S.; , Chambers, R.; and Clark-Carter, D. | 2019 | A qualitative study of cardiovascular disease risk communication in NHS Health Check using different risk calculators: protocol for the RIsk COmmunication in NHS Health Check (RICO) study | C |
| 1206 | Gillett, M.; Brennan, A.; Watson, P.; Khunti, K.; Davies, M.; Mostafa, S.; Gray, L. J. | 2015 | The cost-effectiveness of testing strategies for type 2 diabetes: A modelling study | C |
| 1207 | Gilliland, F. D.; , Mahler, R.; , Hunt, C.; and Davis, S. M. | 1999 | Preventive health care among rural American Indians in New Mexico | C |
| 1208 | Godefrooij, Merijn B.; , van de Kerkhof, Rolf M.; , Wouda, Paul J.; , Vening, Robert A.; , Knottnerus, J. André; , Dinant, Geert-Jan; and Spigt, Mark G. | 2012 | Development, implementation and yield of a cardiometabolic health check | C |

| | | | | |
|---|---|---|---|---|
| 1209 | Griffiths, C.; , Cooke, S.; and Toon, P. | 1994 | Registration health checks: inverse care in the inner city? | C |
| 1210 | Groenenberg, I.; , Crone, M. R.; , van Dijk, S.; , Gebhardt, W. A.; , Ben Meftah, J.; , Middelkoop, B. J.; , Stiggelbout, A. M.; and Assendelft, W. J. | 2015 | 'Check it out!' Decision-making of vulnerable groups about participation in a two-stage cardiometabolic health check: a qualitative study | C |
| 1211 | Groenenberg, I.; Crone, M.; van Dijk, S.; Meftah, J. B.; Hettinga, D.; Middelkoop, B.; Stiggelbout, A.; Assendelft, P. | 2016 | The PreventieConsult and hard-to-reach patients | C |
| 1212 | Gulliford, M. C.; Khoshaba, B.; McDermott, L.; Cornelius, V.; Ashworth, M.; Fuller, F.; Miller, J.; Dodhia, H.; Wright, A. J. | 2018 | Cardiovascular risk at health checks performed opportunistically or following an invitation letter. Cohort study | C |
| 1213 | Hansen, C. B.; Pavlovic, K. M. H.; Sondergaard, J.; Thilsing, T. | 2020 | Does GP empathy influence patient enablement and success in lifestyle change among high risk patients? | C |
| 1214 | Hardeman, W.; , Mitchell, J.; , Pears, S.; , Van Emmenis, M.; , Theil, F.; , Gc, V. S.; , Vasconcelos, J. C.; , Westgate, K.; , Brage, S.; , Suhrcke, M.; , Griffin, S. J.; , Kinmonth, A. L.; , Wilson, E. C. F.; , Prevost, A. T.; , Sutton, S.; and Team, V. B. I. Res | 2020 | Evaluation of a very brief pedometer-based physical activity intervention delivered in NHS Health Checks in England: The VBI randomised controlled trial | C |
| 1215 | Harte, E.; , MacLure, C.; , Martin, A.; , Saunders, C. L.; , Meads, C.; , Walter, F. M.; , Griffin, S. J.; , Mant, J.; and Usher-Smith, J. A. | 2018 | Reasons why people do not attend NHS Health Checks: a systematic review and qualitative synthesis | C |
| 1216 | Hayman, N. | 2010 | Strategies to Improve Indigenous Access for Urban and Regional Populations to Health Services | C |
| 1217 | Hoebel, J.; Richter, M.; Lampert, T. | 2013 | Social status and participation in health checks in men and women in Germany: Results from the German Health Update (GEDA), 2009 and 2010 | C |
| 1218 | Hoebel, J.; Starker, A.; Jordan, S.; Richter, M.; Lampert, T. | 2014 | Determinants of health check attendance in adults: findings from the cross-sectional German Health Update (GEDA) study | C |
| 1219 | Hozawa, A.; , Kuriyama, S.; , Watanabe, I.; , Kakizaki, M.; , Ohmori-Matsuda, K.; , Sone, T.; , Nagai, M.; , Sugawara, Y.; , Nitta, A.; , Li, Q. A.; , Ohkubo, T.; , Murakami, Y.; and Tsuji, I. | 2010 | Participation in health check-ups and mortality using propensity score matched cohort analyses | C |
| 1220 | Ichikawa, D.; Saito, T.; Oyama, H. | 2017 | Impact of predicting health-guidance candidates using massive health check-up data: A data-driven analysis | C |
| 1221 | Jenkinson, C. E.; , Asprey, A.; , Clark, C. E.; and Richards, S. H. | 2015 | Patients' willingness to attend the NHS cardiovascular health checks in primary care: a qualitative interview study | C |
| 1222 | Johannes Scholl, J.; Kurz, P. U. | 2018 | Successful diabetes prevention - The Prevention First longitudinal study (PFLS) | C |
| 1223 | Johnson, R. R.; Casburn, K.; Kilkenny, M.; Cadilhac, D. | 2014 | Actions for change-Know your numbers program identifies multiple risk factors for stroke and actions participants take | C |

| | | | | |
|---|---|---|---|---|
| 1224 | Journath, G.; Hammar, N.; Linnersjo, A.; Vikstrom, M.; Walldius, G.; Krakau, I.; Lindgren, P.; De Faire, U.; Hellenius, M. L. | 2015 | Long-term effects of a cardiovascular prevention program in primary health care in Sweden | C |
| 1225 | Kamstrup-Larsen, N.; , Dalton, S. O.; , Broholm-Jorgensen, M.; , Larsen, L. B.; , Thomsen, J. L.; , Johansen, C.; and Tolstrup, J. S. | 2019 | Using general practitioners to recruit individuals with low socioeconomic position to preventive health checks is feasible: a cross sectional study | C |
| 1226 | Kamstrup-Larsen, N.; , Dalton, S. O.; , Broholm-Jørgensen, M.; , Larsen, L. B.; , Thomsen, J. L.; , Johansen, C.; and Tolstrup, J. S. | 2019 | Using general practitioners to recruit individuals with low socioeconomic position to preventive health checks is feasible: a cross sectional study | C |
| 1227 | Kamstrup-Larsen, N.; , Dalton, S. O.; , Gronbaek, M.; , Broholm-Jorgensen, M.; , Thomsen, J. L.; , Larsen, L. B.; , Johansen, C.; and Tolstrup, J. | 2019 | The effectiveness of general practice-based health checks on health behaviour and incidence on non-communicable diseases in individuals with low socioeconomic position: a randomised controlled trial in Denmark | C |
| 1228 | Kan, M.; Yoshida, H.; Fujiwara, Y.; Watanabe, N.; Tsuchiya, Y.; Shinkai, S. | 2006 | Longitudinal analysis of factors associated with participation in community-based mass screening for the frail elderly in need of care | C |
| 1229 | Katsura, T.; , Fujimoto, M.; , Shizawa, M.; , Hoshino, A.; , Usui, K.; , Yokoyama, E.; and Hara, M. | 2017 | A retrospective cohort study on the risk assessment of newly certificated long-term care need of elderly individuals in a community: Basic checklist and specific health checkup | C |
| 1230 | Kennedy, O.; , Su, F. Z.; , Pears, R.; , Walmsley, E.; and Roderick, P. | 2019 | Evaluating the effectiveness of the NHS Health Check programme in South England: a quasi-randomised controlled trial | C |
| 1231 | Khunti, K.; , Morris, D. H.; , Weston, C. L.; , Gray, L. J.; , Webb, D. R.; and Davies, M. J. | 2013 | Joint prevalence of diabetes, impaired glucose regulation, cardiovascular disease risk and chronic kidney disease in South Asians and White Europeans | C |
| 1232 | Kilkenny, M. F.; Dunstan, L.; Busingye, D.; Purvis, T.; Reyneke, M.; Orgill, M.; Cadilhac, D. A. | 2017 | Knowledge of risk factors for diabetes or cardiovascular disease (CVD) is poor among individuals with risk factors for CVD | C |
| 1233 | Kilkenny, M. F.; Johnson, R.; Andrew, N. E.; Purvis, T.; Hicks, A.; Colagiuri, S.; Cadilhac, D. A. | 2014 | Comparison of two methods for assessing diabetes risk in a pharmacy setting in Australia | C |
| 1234 | Kumar, J.; Chambers, R.; Mawby, Y.; Leese, C.; Iqbal, Z.; Picariello, L.; Richardson, D. | 2011 | Delivering more with less? Making the NHS Health Check work in financially hard times: real time learning from Stoke-on-Trent | C |
| 1235 | Kypridemos, C.; , Collins, B.; , McHale, P.; , Bromley, H.; , Parvulescu, P.; , Capewell, S.; and O'Flaherty, M. | 2018 | Future cost-effectiveness and equity of the NHS Health Check cardiovascular disease prevention programme: Microsimulation modelling using data from Liverpool, UK | C |
| 1236 | Labeit, A.; Peinemann, F.; Baker, R. | 2013 | Utilisation of preventative health check-ups in the UK: Findings from individual-level repeated cross-sectional data from 1992 to 2008 | C |
| 1237 | Lambert, A. M.; Burden, A. C.; Chambers, J.; Marshall, T. | 2012 | Cardiovascular screening for men at high risk in Heart of Birmingham Teaching Primary Care Trust: The 'Deadly Trio' programme | C |

| | | | | |
|---|---|---|---|---|
| 1238 | Langham, S.; Thorogood, M.; Normand, C.; Muir, J.; Jones, L.; Fowler, G. | 1996 | Costs and cost effectiveness of health checks conducted by nurses in primary care: The Oxcheck study | C |
| 1239 | Lauritzen, T.; , Leboeuf-Yde, C.; , Lunde, I. M.; and Nielsen, K. D. | 1995 | Ebeltoft project: baseline data from a five-year randomized, controlled, prospective health promotion study in a Danish population | C |
| 1240 | Lauritzen, T.; , Nielsen, K. D.; , Leboeuf-Yde, C.; and Lunde, I. M. | 1997 | [The health project Ebeltoft: health check ups and discussions in general practice. Basic data from a 5-year, prospective, randomized, controlled population study] | C |
| 1241 | Li, L. J.; , Zhou, J. X.; , Chen, H. T.; , Song, Y. L.; and Xue, Y. M. | 2012 | Effect of HbA(1c) combined FPG on screening diabetes in health check-up | C |
| 1242 | Liaw, T.; Lawrence, M.; Rendell, J. | 1996 | The effect of a computer-generated patient-held medical record summary and/or a written personal health record on patients' attitudes, knowledge and behaviour concerning health promotion | C |
| 1243 | Mandic, S.; Clark-Grill, M.; Wilson, H. | 2010 | Free health screening programs in the workplace and community: Who is likely to participate? | C |
| 1244 | Mant, D. | 1991 | PREVALENCE OF RISK-FACTORS FOR HEART-DISEASE IN OXCHECK TRIAL - IMPLICATIONS FOR SCREENING IN PRIMARY CARE | C |
| 1245 | Maria Woringer, M.; Watt, H. W.; Cecil, E. C.; Chang, K. C.; Majeed, A. M.; Soljak, M. S. | 2015 | Outreach providers administering the NHS health check CVD prevention programme target people at higher CVD risk | C |
| 1246 | Maske, U. E.; Jaedtke, M.; Luppa, M.; Riedel-Heller, S.; Kleiber, D.; Busch, M. A. | 2019 | Diagnosed depression and utilization of health care services and preventive measures in the general adult population in Germany: Results from a nationwide cross-sectional telephone survey | C |
| 1247 | Maske, U.; Maren, J.; Hapke, U.; Kleiber, D.; Busch, M. A. | 2016 | Diagnosed depression and utilization of healthcare and preventive services in the general adult population in Germany | C |
| 1248 | McNaughton, R. J.; , Oswald, N. T. A.; , Shucksmith, J. S.; , Heywood, P. J.; and Watson, P. S. | 2011 | Making a success of providing NHS Health Checks in community pharmacies across the Tees Valley: a qualitative study | C |
| 1249 | McNaughton, R. J.; Shucksmith, J. | 2015 | Reasons for (non)compliance with intervention following identification of 'high-risk' status in the NHS Health Check programme | C |
| 1250 | Mitsuhashi, Y.; , Kishi, R.; , Eguchi, T.; , Miyake, H.; and Maeda, N. | 2003 | [Factors associated with participation in medical checkups of the elderly at home comparison of 3 regions with different social backgrounds] | C |
| 1251 | Moriarty, Y.; , Townson, J.; , Quinn-Scoggins, H.; , Padgett, L.; , Owen, S.; , Smits, S.; , Playle, R.; , Dimitropoulou, P.; , Sewell, B.; , Kolovou, V.; , Buckle, P.; , Carter, B.; , Edwards, A.; , Hepburn, J.; , Matthews, M.; , Mitchell, C.; , Neal, R. D.; , Robling, M.; , Wood, F.; and Brain, K. | 2019 | Improving cancer symptom awareness and help-seeking among adults living in socioeconomically deprived communities in the UK using a facilitated health check: A protocol for the Awareness and Beliefs About Cancer (ABACus) Randomised Control Trial | C |

| | | | | |
|---|---|---|---|---|
| 1252 | Movahedi, M.; Farajzadegan, Z.; Khadivi, R. | 2019 | Middle-aged health checks program outputs in non-communicable diseases screening in Iran | C |
| 1253 | Mytton, O. T.; Jackson, C.; Steinacher, A.; Goodman, A.; Langenberg, C.; Griffin, S.; Wareham, N.; Woodcock, J. | 2018 | The current and potential health benefits of the National Health Service Health Check cardiovascular disease prevention programme in England: A microsimulation study | C |
| 1254 | Nichols, M.; Waters, E.; Cleary, J.; Proimos, J.; Allender, S. | 2014 | Feasibility of using routinely collected height and weight measurements of preschool children for monitoring overweight and obesity in Australia | C |
| 1255 | Nielen, M.; Van Der Meer, V.; Assendelft, P.; Schellevis, F. | 2011 | An evidence-based cardiometabolic health check in general practice | C |
| 1256 | Oexmann, M. J.; Ascanio, R.; Egan, B. M. | 2001 | Efficacy of a church-based intervention on cardiovascular risk reduction | C |
| 1257 | Palladino, R.; Vamos, E.; Chang, K. C. M.; Millett, C. | 2017 | Impact of a national diabetes risk assessment and screening programme in England: A quasi-experimental study | C |
| 1258 | Park, Sang-Mi;  and Moon, Sang-Sik | 2016 | Elderly Koreans who consider suicide: Role of healthcare use and financial status | C |
| 1259 | Park, S. M.;  and Hong, S. P. | 2020 | The association of health care access and utilization with self-perceived health in South Korea: the significance of age | C |
| 1260 | Pathy, M. S. J.; , Bayer, A.; , Harding, K.;  and Dibble, A. | 1992 | RANDOMIZED TRIAL OF CASE FINDING AND SURVEILLANCE OF ELDERLY PEOPLE AT HOME | C |
| 1261 | Petersen, J.; Kontsevaya, A.; McKee, M.; Richardson, E.; Cook, S.; Malyutina, S.; Kudryavtsev, A. V.; Leon, D. A. | 2020 | Primary care use and cardiovascular disease risk in Russian 40-69 year olds: a cross-sectional study | C |
| 1262 | Petter, J.; , Reitsma-van Rooijen, M. M.; , Korevaar, J. C.;  and Nielen, M. M. J. | 2015 | Willingness to participate in prevention programs for cardiometabolic diseases | C |
| 1263 | Robson, J.; , Dostal, I.; , Madurasinghe, V.; , Sheikh, A.; , Hull, S.; , Boomla, K.; , Griffiths, C.;  and Eldridge, S. | 2017 | NHS Health Check comorbidity and management: an observational matched study in primary care | C |
| 1264 | Robson, J.; Dostal, I.; Madurasinghe, V.; Sheikh, A.; Hull, S.; Boomla, K.; Page, H.; Griffiths, C.; Eldridge, S. | 2015 | The NHS Health Check programme: Implementation in east London 2009-2011 | C |
| 1265 | Robson, J.; Dostal, I.; Sheikh, A.; Eldridge, S.; Madurasinghe, V.; Griffiths, C.; Coupland, C.; Hippisley-Cox, J. | 2016 | The NHS Health Check in England: An evaluation of the first 4 years | C |
| 1266 | Rommel, A.; Frank, L.; Lampert, T. | 2016 | Utilization of preventive care among people with migrant background | C |
| 1267 | Schlichthorst, M.; Sanci, L. A.; Pirkis, J.; Spittal, M. J.; Hocking, J. S. | 2016 | Why do men go to the doctor? Socio-demographic and lifestyle factors associated with healthcare utilisation among a cohort of Australian men | C |
| 1268 | Scholl, J. G.; Kurz, P. U. | 2018 | A modern health check-up with lifestyle coaching improves overall cardiovascular health | C |

| | | | | |
|---|---|---|---|---|
| 1269 | Scholl, J.; Kurz, P. | 2015 | Long term benefits of a contemporary health check-up with evidence-based risk communication and motivational coaching-the PF study | C |
| 1270 | Scholl, J.; Kurz, P. | 2015 | A contemporary health check-up as a one-time intervention with individual lifestyle coaching can substantially lower diabetes risk in patients with pre-diabetes-the PF study | C |
| 1271 | Schuetz, C. A.; , Alperin, P.; , Guda, S.; , van Herick, A.; , Cariou, B.; , Eddy, D.; , Gumprecht, J.; , Nicolucci, A.; , Schwarz, P.; , Wareham, N. J.; , Witte, D. R.; and Smith, U. | 2013 | A standardized vascular disease health check in europe: a cost-effectiveness analysis | C |
| 1272 | Shah, S.; Nelapatla, R.; Agarwal, K.; Pandya, B. K. | 2015 | World kidney day: Five year analysis of health awareness events in UK | C |
| 1273 | Shah, S.; Pandya, B. | 2013 | World kidney day (WKD): Four year analysis of health events in UK | C |
| 1274 | Shimoda, A.; Ichikawa, D.; Oyama, H. | 2018 | Using machine-learning approaches to predict non-participation in a nationwide general health check-up scheme | C |
| 1275 | Shin, H. Y.; , Kang, H. T.; , Lee, J. W.; and Lim, H. J. | 2018 | The Association between Socioeconomic Status and Adherence to Health Check-up in Korean Adults, Based on the 2010-2012 Korean National Health and Nutrition Examination Survey | C |
| 1276 | Simmons, R. K.; , Griffin, S. J.; , Witte, D. R.; , Borch-Johnsen, K.; , Lauritzen, T.; and Sandbaek, A. | 2017 | Effect of population screening for type 2 diabetes and cardiovascular risk factors on mortality rate and cardiovascular events: a controlled trial among 1,912,392 Danish adults | C |
| 1277 | Simmons, R. K.; Griffin, S. J.; Witte, D. R.; Borch-Johnsen, K.; Lauritzen, T.; Sandbæk, A. | 2017 | Effect of population screening for type 2 diabetes and cardiovascular risk factors on mortality rate and cardiovascular events: a controlled trial among 1,912,392 Danish adults | C |
| 1278 | Skaaby, T.; , Jorgensen, T.; and Linneberg, A. | 2017 | Effects of invitation to participate in health surveys on the incidence of cardiovascular disease: a randomized general population study | C |
| 1279 | Skaaby, T.; , Jorgensen, T.; and Linneberg, A. | 2018 | A randomized general population study of the effects of repeated health checks on incident diabetes | C |
| 1280 | Smith, P.; , Smits, S.; , Owen, S.; , Wood, F.; , McCutchan, G.; , Carter, B.; , Edwards, A.; , Robling, M.; , Townson, J.; and Brain, K. | 2018 | Feasibility and acceptability of a cancer symptom awareness intervention for adults living in socioeconomically deprived communities | C |
| 1281 | Srichang, N.; Jiamjarasrangsi, W.; Aekplakorn, W.; Supakankunti, S. | 2011 | Cost and effectiveness of screening methods for abnormal fasting plasma glucose among Thai adults participating in the annual health check-up at king chulalongkorn memorial hospital | C |
| 1282 | Tada, A.; Matsukubo, T. | 2003 | Relationship between oral health behaviors and general health behaviors in a Japanese adult population | C |
| 1283 | Taneichi, H.; Sairenchi, T.; Wada, K.; Muto, T. | 2012 | Burden of cardiovascular risk factors on the frequency of medical consultations among Japanese national health insurance beneficiaries | C |
| 1284 | Thorogood, M.; Coulter, A.; Jones, L.; Yudkin, P.; Muir, J.; Mant, D. | 1993 | Factors affecting response to an invitation to attend for a health check | C |

| | | | | |
|---|---|---|---|---|
| 1285 | Visram, S.; Carr, S. M.; Geddes, L. | 2015 | Can lay health trainers increase uptake of NHS Health Checks in hard-to-reach populations? A mixed-method pilot evaluation | C |
| 1286 | Waller, D.; Agass, M.; Mant, D.; Coulter, A.; Fuller, A.; Jones, L. | 1990 | Health checks in general practice: Another example of inverse care? | C |
| 1287 | Walsh, N. | 2019 | Peripheral bone density measurement: An interdisciplinary initiative for improving health outcomes for people with intellectual disabilities in the republic of Ireland (ROI) | C |
| 1288 | Welch, L. S.; , Dement, J.; , Ringen, K.; , Cranford, K.; and Quinn, P. S. | 2017 | Impact of Secondary Prevention in an Occupational High-Risk Group | C |
| 1289 | Williams, J.; Miners, A.; Harris, R.; Mandal, S.; Simmons, R.; Ireland, G.; Hickman, M.; Gore, C.; Vickerman, P. | 2019 | The cost-effectiveness of one-time birth cohort screening for hepatitis C as part of the National Health Service health check programme for 40 to 74 year olds in England | C |
| 1290 | Wilson, R.; Kuh, D.; Stafford, M. | 2019 | Variations of health check attendance in later life: results from a British birth cohort study | C |
| 1291 | Woringer, M.; Cecil, E.; Watt, H.; Chang, K.; Hamid, F.; Khunti, K.; Dubois, E.; Evason, J.; Majeed, A.; Soljak, M. | 2017 | Evaluation of community provision of a preventive cardiovascular programme - the National Health Service Health Check in reaching the under-served groups by primary care in England: cross sectional observational study | C |
| 1292 | Woringer, M.; Nielsen, J. J.; Zibarras, L.; Evason, J.; Kassianos, A. P.; Harris, M.; Majeed, A.; Soljak, M. | 2017 | Development of a questionnaire to evaluate patients' awareness of cardiovascular disease risk in England's National Health Service Health Check preventive cardiovascular programme | C |
| 1293 | Bender, A. M.; Jørgensen, T.; Pisinger, C. | 2015 | Is self-selection the main driver of positive interpretations of general health checks? The Inter99 randomized trial | D |
| 1294 | Cohen, M.; Azaiza, F. | 2007 | Health-promoting behaviors and health locus of control from a multicultural perspective | D |
| 1295 | Nahar, P.; , van Marwijk, H.; , Gibson, L.; , Musinguzi, G.; , Anthierens, S.; , Ford, E.; , Bremner, S. A.; , Bowyer, M.; , Le Reste, J. Y.; , Sodi, T.; and Bastiaens, H. | 2020 | A protocol paper: community engagement interventions for cardiovascular disease prevention in socially disadvantaged populations in the UK: an implementation research study | D |
| 1296 | Bunten, A.; Porter, L.; Gold, N.; Bogle, V. | 2020 | A systematic review of factors influencing NHS health check uptake: invitation methods, patient characteristics, and the impact of interventions | E |
| 1297 | Cook, E. J.; Sharp, C.; Randhawa, G.; Guppy, A.; Gangotra, R.; Cox, J. | 2016 | Who uses NHS health checks? Investigating the impact of ethnicity and gender and method of invitation on uptake of NHS health checks | E |
| 1298 | Forster, A. S.; Burgess, C.; McDermott, L.; Wright, A. J.; Dodhia, H.; Conner, M.; Miller, J.; Rudisill, C.; Cornelius, V.; Gulliford, M. C. | 2014 | Enhanced invitation methods to increase uptake of NHS health checks: Study protocol for a randomized controlled trial | E |
| 1299 | Gardiner, E.; Davies, M. J.; Hiles, S.; Khunti, K. | 2010 | Response rates to population based screening for type 2 diabetes and pre-diabetes: A systematic review | E |

| | | | | |
|---|---|---|---|---|
| 1300 | Aalto, M.; Alho, H.; Halme, J. T.; Seppä, K. | 2009 | AUDIT and its abbreviated versions in detecting heavy and binge drinking in a general population survey | Duplicate |
| 1301 | Aarup, M.; Sokolowski, I.; Lous, J. | 2008 | The prevalence of obesity and overweight among 3 year-old children in the municipality of Aalborg and identification of risk factors | Duplicate |
| 1302 | Ab Rahman, N.; Sivasampu, S.; Mohamad Noh, K.; Khoo, E. M. | 2016 | Health profiles of foreigners attending primary care clinics in Malaysia | Duplicate |
| 1303 | Aguado Taberné, C.; Martínez de la Iglesia, J.; Espejo Espejo, J.; Yun Casalilla, A.; Muñoz Alamo, M.; Ruiz Moral, R. | 1996 | Clinical situation, knowledge and risky behavior of patients with human immunodeficiency virus infection attending a health center | Duplicate |
| 1304 | Aida, Y.; Shibata, Y.; Osaka, D.; Abe, S.; Inoue, S.; Tokairin, Y.; Igarashi, A.; Yamauchi, K.; Nemoto, T.; Nunomiya, K.; Kishi, H.; Sato, M.; Kubota, I. | 2011 | Relationship between serum uric acid and spirometric values in healthy Japanese individuals: The takahata study | Duplicate |
| 1305 | Alageel, S.; Gulliford, M. C.; Wright, A.; Khoshaba, B.; Burgess, C. | 2020 | Engagement with advice to reduce cardiovascular risk following a health check programme: A qualitative study | Duplicate |
| 1306 | Alexander, K. E.; Brijnath, B.; Mazza, D. | 2013 | 'Can they really identify mental health problems at the age of three?' Parent and practitioner views about screening young children's social and emotional development | Duplicate |
| 1307 | Avila, J. C.; Kuo, Y. F.; Rodriguez, A. M.; Wong, R.; Kaul, S. | 2017 | Preventive services use among female survivors of adolescent and young adult cancer | Duplicate |
| 1308 | Bailie, R. S.; Si, D.; Connors, C. M.; Kwedza, R.; O'Donoghue, L.; Kennedy, C.; Cox, R.; Liddle, H.; Hains, J.; Dowden, M. C.; Burke, H. P.; Brown, A.; Weeramanthri, T.; Thompson, S. | 2011 | Variation in quality of preventive care for well adults in Indigenous community health centres in Australia | Duplicate |
| 1309 | Banim, P. R.; Luben, R.; Khaw, K. T.; Wareham, N.; Hart, A. | 2011 | Physical activity and the risk of developing pancreatic cancer - Data from a UK prospective study (EPIC-Norfolk) | Duplicate |
| 1310 | Bender, A. M.; Jørgensen, T.; Pisinger, C. | 2017 | Do high participation rates improve effects of population-based general health checks? | Duplicate |
| 1311 | Bender, A. M.; Jørgensen, T.; Pisinger, C. | 2019 | Higher mortality in women living in high-participation areas of a population-based health check and lifestyle intervention study | Duplicate |
| 1312 | Bender, A. M.; Kawachi, I.; Jørgensen, T.; Pisinger, C. | 2015 | Neighborhood social capital is associated with participation in health checks of a general population: a multilevel analysis of a population-based lifestyle intervention- the Inter99 study | Duplicate |
| 1313 | Bender, A. M.; Kawachi, I.; Jørgensen, T.; Pisinger, C. | 2015 | Neighborhood deprivation is strongly associated with participation in a population-based health check | Duplicate |
| 1314 | Blake, H.; , Hussain, B.; , Hand, J.; , Juma, A.; and Evans, C. | 2019 | Employers' views of the "Healthy Hub Roadshow": a workplace HIV testing intervention in England | Duplicate |
| 1315 | Blake, H.; , Hussain, B.; , Hand, J.; , Juma, A.; and Evans, C. | 2019 | Employers' views of the "Healthy Hub Roadshow": a workplace HIV testing intervention in England(*) | Duplicate |
| 1316 | Blake, H.; Hussain, B.; Hand, J.; Juma, A.; Evans, C. | 2019 | Employers’ views of the “Healthy Hub Roadshow”: a workplace HIV testing intervention in England* | Duplicate |

| | | | | |
|---|---|---|---|---|
| 1317 | Bleakley, A.; Merzel, C.; Messeri, P.; Gift, T.; Kevin Malotte, C.; Middlestadt, S.; VanDevanter, N. | 2008 | Check out that body: A community awareness campaign in New York City | Duplicate |
| 1318 | Boongird, P.; , Chamnan, P.; , Laptikultham, S.; , Krittayapoositpot, P.; , Nitiyanant, W.; , Aekplakorn, W.; , Mangklabruks, A.; and Investigators, Hcur | | Dose-response relationship between physical exercise and risk of physician-diagnosed dementia in 206 073 Thai community-dwelling men and women: HCUR study | Duplicate |
| 1319 | Brand, T.; , Kleer, D.; , Samkange-Zeeb, F.; and Zeeb, H. | 2015 | Prevention among migrants. Participation, migrant sensitive strategies and programme characteristics | Duplicate |
| 1320 | Brangan, E.; Stone, T. J.; Chappell, A.; Harrison, V.; Horwood, J. | 2019 | Patient experiences of telephone outreach to enhance uptake of NHS Health Checks in more deprived communities and minority ethnic groups: A qualitative interview study | Duplicate |
| 1321 | Bressington, D. T.; Mui, J.; Cheung, E. F. C.; Petch, J.; Clark, A. B.; Gray, R. | 2013 | The prevalence of metabolic syndrome amongst patients with severe mental illness in the community in Hong Kong - a cross sectional study | Duplicate |
| 1322 | Brockmann, P. E.; Diaz, B.; Damiani, F.; Villarroel, L.; Núñez, F.; Bruni, O. | 2016 | Impact of television on the quality of sleep in preschool children | Duplicate |
| 1323 | Broholm-Jørgensen, M.; Kamstrup-Larsen, N.; Guassora, A. D.; Reventlow, S.; Dalton, S. O.; Tjørnhøj-Thomsen, T. | 2019 | 'It can't do any harm': A qualitative exploration of accounts of participation in preventive health checks | Duplicate |
| 1324 | Brown, K.; , Boot, D.; , Groom, L.; and Williams, E. | 1997 | Problems found in the over-75s by the annual health check | Duplicate |
| 1325 | Brown, K.; , Boot, D.; , Groom, L.; and Williams, E. I. | 1997 | Problems found in the over-75s by the annual health check | Duplicate |
| 1326 | Browning, C.; Chapman, A.; Cowlishaw, S.; Li, Z.; Thomas, S. A.; Yang, H.; Zhang, T. | 2011 | The Happy Life Club™ study protocol: a cluster randomised controlled trial of a type 2 diabetes health coach intervention | Duplicate |
| 1327 | Browning, C.; Chapman, A.; Yang, H.; Liu, S.; Zhang, T.; Enticott, J. C.; Thomas, S. A. | 2016 | Management of type 2 diabetes in China: The Happy Life Club, a pragmatic cluster randomised controlled trial using health coaches | Duplicate |
| 1328 | Brütting, J.; Druschke, D.; Spitzer, S.; Seibt, R. | 2018 | Health status of long-term sick leave and working female teachers in Germany: A cross-sectional study | Duplicate |
| 1329 | Bukman, A. J.; Teuscher, D.; Ben Meftah, J.; Groenenberg, I.; Crone, M. R.; van Dijk, S.; Bos, M. B.; Feskens, E. J. | 2016 | Exploring strategies to reach individuals of Turkish and Moroccan origin for health checks and lifestyle advice: a mixed-methods study | Duplicate |
| 1330 | Cabrera, M.; Sánchez-Chaparro, M. A.; Valdivielso, P.; Quevedo-Aguado, L.; Catalina-Romero, C.; Fernández-Labandera, C.; Ruiz-Moraga, M.; González-Santos, P.; Calvo-Bonacho, E. | 2014 | Prevalence of atherogenic dyslipidemia: Association with risk factors and cardiovascular risk in Spanish working population. "ICARIA" study | Duplicate |
| 1331 | Cao, X.; Wang, D.; Zhou, J.; Chen, Z. | 2017 | Comparison of lipoprotein derived indices for evaluating cardio-metabolic risk factors and subclinical organ damage in middle-aged Chinese adults | Duplicate |
| 1332 | Carlos-Oliva, D.; , Vitale, M. P.; , Grañana, N.; , Rouvier, M. E.; and Zeltman, C. | 2020 | [Neurodevelopmental development with the use of the Ages and Stages Questionnaire (ASQ-3) in monitoring children's health] | Duplicate |

| | | | | |
|---|---|---|---|---|
| 1333 | Carlos-Oliva, D.; Paula Vitale, M.; Grañana, N.; Eugenia Rouvier, M.; Zeltman, C. | 2020 | Neurodevelopmental development with the use of the Ages and Stages Questionnaire (ASQ-3) in monitoring children's health | Duplicate |
| 1334 | Carstensen, K.; , Kousgaard, M. B.; and Burau, V. | 2019 | Sustaining an intervention for physical health promotion in community mental health services: A multisite case study | Duplicate |
| 1335 | Carstensen, K.; Brostrøm Kousgaard, M.; Burau, V. | 2019 | Sustaining an intervention for physical health promotion in community mental health services: A multisite case study | Duplicate |
| 1336 | Chan, A.; Amorese, C.; Harris, M. | 2008 | New 45-49 year health checks: GP uptake of MBS item 717 | Duplicate |
| 1337 | Chen, W.; Li, T.; Zou, G.; Li, X.; Shi, L.; Feng, S.; Shi, J.; Zhou, F.; Han, S.; Ling, L. | 2016 | Study protocol: a cluster randomized controlled trial to assess the effectiveness of a multi-pronged behavioural intervention to improve use of personal protective equipment among migrant workers exposed to organic solvents in small and medium-sized enterprises | Duplicate |
| 1338 | Chew, C. A.; , Wilkin, D.; and Glendenning, C. | 1994 | ANNUAL ASSESSMENT OF PATIENTS AGED 75 YEARS AND OVER - GENERAL-PRACTITIONERS AND PRACTICE NURSES VIEWS AND EXPERIENCES | Duplicate |
| 1339 | Chew, C. A.; Wilkin, D.; Glendenning, A. | 1994 | Annual assessment of patients aged 75 years and over: General practitioners' and practice nurses' views and experiences | Duplicate |
| 1340 | Coghill, N.; Garside, L.; Montgomery, A. A.; Feder, G.; Horwood, J. | 2018 | NHS health checks: a cross- sectional observational study on equity of uptake and outcomes | Duplicate |
| 1341 | Coley, N.; Rosenberg, A.; van Middelaar, T.; Soulier, A.; Barbera, M.; Guillemont, J.; Steensma, J.; Igier, V.; Eskelinen, M.; Soininen, H.; Moll van Charante, E.; Richard, E.; Kivipelto, M.; Andrieu, S.; Sindi, S.; Solomon, A.; Hartmann, T.; Brayne, C.; van Gool, P.; Beishuizen, C.; Jongstra, S.; van Wanrooij, L.; Hoevenaar-Blom, M.; Ngandu, T.; Mangiasche, F.; Meiller, Y.; van de Groep, B.; Braynefor, C. | 2019 | Older Adults' Reasons for Participating in an eHealth Prevention Trial: A Cross-Country, Mixed-Methods Comparison | Duplicate |
| 1342 | Dalager, T.; Justesen, J. B.; Murray, M.; Boyle, E.; Sjøgaard, G. | 2016 | Implementing intelligent physical exercise training at the workplace: health effects among office workers-a randomized controlled trial | Duplicate |
| 1343 | Dalal, K.; Dawad, S. | 2009 | Non-utilization of public health care facilities: examining the reasons through a national study of women in India | Duplicate |
| 1344 | Dalton, A. R. H.; Bottle, A.; Okoro, C.; Majeed, A.; Millett, C. | 2011 | Uptake of the NHS Health Checks programme in a deprived, culturally diverse setting: Cross-sectional study | Duplicate |
| 1345 | Damman, O. C.; van der Beek, A. J.; Timmermans, D. R. | 2015 | Employees are ambivalent about health checks in the occupational setting | Duplicate |
| 1346 | Davison, K. L.; Reynolds, C. A.; Andrews, N.; Brailsford, S. R.; Kohli, H.; Miflin, G.; Wickenden, C.; Hay, F.; Allan, J.; Field, S.; Carter, M.; Maguire, K.; Ratchford, J.; Davison, K.; Brailsford, S.; Reynolds, C.; Andrews, N. | 2015 | Getting personal with blood donors - the rationale for, methodology of and an overview of participants in the UK blood donor survey | Duplicate |

| | | | | |
|---|---|---|---|---|
| 1347 | de Waard, A. K. M.; Wändell, P. E.; Holzmann, M. J.; Korevaar, J. C.; Hollander, M.; Gornitzki, C.; de Wit, N. J.; Schellevis, F. G.; Lionis, C.; Søndergaard, J.; Seifert, B.; Carlsson, A. C. | 2018 | Barriers and facilitators to participation in a health check for cardiometabolic diseases in primary care: A systematic review | Duplicate |
| 1348 | de Waard, A. M.; , Wändell, P. E.; , Holzmann, M. J.; , Korevaar, J. C.; , Hollander, M.; , Gornitzki, C.; , de Wit, N. J.; , Schellevis, F. G.; , Lionis, C.; , Søndergaard, J.; , Seifert, B.; and Carlsson, A. C. | 2018 | Barriers and facilitators to participation in a health check for cardiometabolic diseases in primary care: A systematic review | Duplicate |
| 1349 | Dhanapalaratnam, R.; Fanaian, M.; Harris, M. F. | 2011 | Lifestyle intervention - a study on maintenance in general practice | Duplicate |
| 1350 | Díaz, J. J.; Saldarriaga, V. | 2019 | Encouraging use of prenatal care through conditional cash transfers: Evidence from JUNTOS in Peru | Duplicate |
| 1351 | Dolák, F.; Šedová, L.; Nováková, D.; Olišarová, V. | 2016 | Approach to prevention of obesity of Roma population in the Region of South Bohemia with focus on selected eating behaviors | Duplicate |
| 1352 | Durão, S.; Ajumobi, O.; Kredo, T.; Naude, C.; Levitt, N. S.; Steyn, K.; Bradshaw, D.; Young, T. | 2015 | Evidence insufficient to confirm the value of population screening for diabetes and hypertension in low- and middle-income settings | Duplicate |
| 1353 | Enocson, A.; , Jolly, K.; , Jordan, R. E.; , Fitzmaurice, D. A.; , Greenfield, S. M.; , Adab, P.; and Team, Bliss Res | 2018 | Case-finding for COPD in primary care: a qualitative study of patients' perspectives | Duplicate |
| 1354 | Enocson, A.; Jolly, K.; Jordan, R. E.; Fitzmaurice, D. A.; Greenfield, S. M.; Adab, P. | 2018 | Case-finding for COPD in primary care: A qualitative study of patients’ perspectives | Duplicate |
| 1355 | Fanaian, M.; Laws, R. A.; Passey, M.; McKenzie, S.; Wan, Q.; Davies, G. P.; Lyle, D.; Harris, M. F. | 2010 | Health improvement and prevention study (HIPS) - evaluation of an intervention to prevent vascular disease in general practice | Duplicate |
| 1356 | Fletcher, A. E.; Jones, D. A.; Bulpitt, C. J.; Tulloch, A. J. | 2002 | The MRC trial of assessment and management of older people in thecommunity: Objectives, design and interventions [ISRCTN23494848] | Duplicate |
| 1357 | Fletcher, A. E.; Jones, D. A.; Bulpitt, C. J.; Tulloch, A. J. | 2002 | The MRC trial of assessment and management of older people in the community: objectives, design and interventions [ISRCTN23494848] | Duplicate |
| 1358 | Fujibayashi, K.; Yokokawa, H.; Gunji, T.; Sasabe, N.; Okumura, M.; Iijima, K.; Haniu, T.; Hisaoka, T.; Fukuda, H. | 2015 | Utility of 75-g oral glucose tolerance test results and hemoglobin a1c values for predicting the incidence of diabetes mellitus among middle-aged Japanese men: A large-scale retrospective cohort study performed at a single hospital | Duplicate |
| 1359 | Fujii, R.; Yamada, H.; Yamazaki, M.; Munetsuna, E.; Ando, Y.; Ohashi, K.; Ishikawa, H.; Shimoda, H.; Sakata, K.; Ogawa, A.; Kobayashi, S.; Suzuki, K. | 2019 | Circulating microRNAs (miR-126, miR-197, and miR-223) are associated with chronic kidney disease among elderly survivors of the Great East Japan Earthquake | Duplicate |
| 1360 | Fujiwara, Y.; Nishi, M.; Watanabe, N.; Lee, S.; Inoue, K.; Yoshida, H.; Sakuma, N.; Kureta, Y.; Ishii, K.; Uchida, H.; Kakuno, F.; Shinkai, S. | 2006 | An intergenerational health promotion program involving older adults in urban areas. "Research of Productivity by Intergenerational Sympathy (REPRINTS)": first-year experience and short-term effects | Duplicate |
| 1361 | Genc, M.; Ruusuvaara, L.; Mardh, P. A. | 1993 | An economic evaluation of screening for Chlamydia trachomatis in adolescent males | Duplicate |

| | | | | |
|---|---|---|---|---|
| 1362 | Gilliland, F. D.; Mahler, R.; Hunt, W. C.; Davis, S. M. | 1999 | Preventive health care among rural American Indians in New Mexico | Duplicate |
| 1363 | Godefrooij, M. B.; Van de Kerkhof, R. M.; Wouda, P. J.; Vening, R. A.; Knottnerus, J. A.; Dinant, G. J.; Spigt, M. G. | 2012 | Development, implementation and yield of a cardiometabolic health check | Duplicate |
| 1364 | Granollers Mercader, S.; Pont Ribas, A. | 1993 | Nurse care in primary health care: diagnosis and follow-up of health problems | Duplicate |
| 1365 | Griffiths, C.; Sturdy, P.; Brewin, P.; Bothamley, G.; Eldridge, S.; Martineau, A.; MacDonald, M.; Ramsay, J.; Tibrewal, S.; Levi, S.; Zumla, A.; Feder, G. | 2007 | Educational outreach to promote screening for tuberculosis in primary care: a cluster randomised controlled trial | Duplicate |
| 1366 | Groenenberg, Iris; , Crone, Mathilde R.; , van Dijk, Sandra; , Gebhardt, Winnifred A.; , Ben Meftah, Jamila; , Middelkoop, Barend J. C.; , Stiggelbout, Anne M.; and Assendelft, Willem J. J. | 2015 | ‘Check it out!’ Decision-making of vulnerable groups about participation in a two-stage cardiometabolic health check: A qualitative study | Duplicate |
| 1367 | Groenenberg, I.; , Crone, M. R.; , van Dijk, S.; , Gebhardt, W. A.; , Ben Meftah, J.; , Middelkoop, B. J. C.; , Stiggelbout, A. M.; and Assendelft, W. J. J. | 2015 | 'Check it out!' Decision-making of vulnerable, groups about participation in a two-stage cardiometabolic health check: A qualitative study | Duplicate |
| 1368 | Groenenberg, I.; Crone, M. R.; van Dijk, S.; Ben Meftah, J.; Middelkoop, B. J.; Assendelft, W. J.; Stiggelbout, A. M. | 2015 | Response and participation of underserved populations after a three-step invitation strategy for a cardiometabolic health check | Duplicate |
| 1369 | Groenenberg, I.; Crone, M. R.; van Dijk, S.; Gebhardt, W. A.; Ben Meftah, J.; Middelkoop, B. J. C.; Stiggelbout, A. M.; Assendelft, W. J. J. | 2015 | 'Check it out!' Decision-making of vulnerable groups about participation in a two-stage cardiometabolic health check: A qualitative study | Duplicate |
| 1370 | Grubb, N. R.; Elder, D.; Broadhurst, P.; Reoch, A.; Tassie, E.; Neilson, A. | 2019 | Atrial fibrillation case finding in over 65s with cardiovascular risk factors – Results of initial Scottish clinical experience | Duplicate |
| 1371 | Hagen, B.; Strauch, S. | 2011 | The J1 adolescent health check-up: Analysis of data from the German KiGGS Survey | Duplicate |
| 1372 | Häkkänen, Paula; , Ketola, Eeva; and Laatikainen, Tiina | 2018 | Screening and treatment of obesity in school health care—The gap between clinical guidelines and reality | Duplicate |
| 1373 | Häkkänen, P.; Ketola, E.; Laatikainen, T. | 2018 | Screening and treatment of obesity in school health care - the gap between clinical guidelines and reality | Duplicate |
| 1374 | Hao, L.; Wang, Z.; Wang, Y.; Wang, J.; Zeng, Z. | 2020 | Association between Cardiorespiratory Fitness, Relative Grip Strength with Non-Alcoholic Fatty Liver Disease | Duplicate |
| 1375 | Hardeman, W.; Mitchell, J.; Pears, S.; Van Emmenis, M.; Theil, F.; Gc, V. S.; Vasconcelos, J. C.; Westgate, K.; Brage, S.; Suhrcke, M.; Griffin, S. J.; Kinmonth, A. L.; Wilson, E. C. F.; Prevost, A. T.; Sutton, S. | 2020 | Evaluation of a very brief pedometer-based physical activity intervention delivered in NHS Health Checks in England: The VBI randomised controlled trial | Duplicate |
| 1376 | Harris, M. F.; , Fanaian, M.; , Jayasinghe, U. W.; , Passey, M. E.; , McKenzie, S. H.; , Powell Davies, G.; , Lyle, D. M.; , Laws, R. A.; , Schütze, H.; and Wan, Q. | 2012 | A cluster randomised controlled trial of vascular risk factor management in general practice | Duplicate |

| | | | | |
|---|---|---|---|---|
| 1377 | Harris, M. F.; Fanaian, M.; Jayasinghe, U. W.; Passey, M. E.; McKenzie, S. H.; Davies, G. P.; Lyle, D. M.; Laws, R. A.; Schütze, H.; Wan, Q. | 2012 | A cluster randomised controlled trial of vascular risk factor management in general practice | Duplicate |
| 1378 | Hasegawa, M.; , Akter, S.; , Hu, H. H.; , Kashino, I.; , Kuwahara, K.; , Okazaki, H.; , Sasaki, N.; , Ogasawara, T.; , Eguchi, M.; , Kochi, T.; , Miyamoto, T.; , Nakagawa, T.; , Honda, T.; , Yamamoto, S.; , Murakami, T.; , Shimizu, M.; , Uehara, A.; , Yamamoto, M.; , Imai, T.; , Nishihara, A.; , Tomita, K.; , Nagahama, S.; , Hori, A.; , Konishi, M.; , Kabe, I.; , Mizoue, T.; , Kunugita, N.; , Dohi, S.; , Mizoue, T.; , Akter, S.; , Hu, H.; , Inoue, Y.; , Fukunaga, A.; , Kashino, I.; , Islam, Z.; , Konishi, M.; , Nanri, A.; , Kurotani, K.; , Kuwahara, K.; , Watanabe, Y.; , Imai, T.; , Miyamoto, T.; , Hasegawa, M.; , Shirozu, M.; , Kabe, I.; , Shimizu, C.; , Gonmori, N.; , Ogasawara, A.; , Kato, N.; , Tomizawa, A.; , Kunugita, N.; , Sone, T.; , Fukasawa, K.; , Hori, A.; , Nishiura, C.; , Kinugawa, C.; , Kuroda, R.; , Yamamoto, K.; , Ohtsu, M.; , Sakamoto, N.; , Osaki, Y.; , Totsuzaki, T.; , Endo, M.; , Itoh, T.; , Kawashima, M.; , Masuda, M.; , Kitahara, K.; , Yokoya, T.; , Fukai, K.; , Odagami, K.; , Kobayashi, Y.; and Japan Epidemiology Collaboration, O. | 2020 | Five-year cumulative incidence of overweight and obesity, and longitudinal change in body mass index in Japanese workers: The Japan Epidemiology Collaboration on Occupational Health Study | Duplicate |
| 1379 | Heianza, Y.; , Hara, S.; , Arase, Y.; , Saito, K.; , Fujiwara, K.; , Tsuji, H.; , Kodama, S.; , Hsieh, S. D.; , Mori, Y.; , Shimano, H.; , Yamada, N.; , Kosaka, K.; and Sone, H. | 2011 | HbA(1c) 5.7-6.4% and impaired fasting plasma glucose for diagnosis of prediabetes and risk of progression to diabetes in Japan (TOPICS 3): a longitudinal cohort study | Duplicate |
| 1380 | Heianza, Y.; Hara, S.; Arase, Y.; Saito, K.; Fujiwara, K.; Tsuji, H.; Kodama, S.; Hsieh, S. D.; Mori, Y.; Shimano, H.; Yamada, N.; Kosaka, K.; Sone, H. | 2011 | HbA1c 5·7-6·4 and impaired fasting plasma glucose for diagnosis of prediabetes and risk of progression to diabetes in Japan (TOPICS 3): A longitudinal cohort study | Duplicate |
| 1381 | Heida, A.; Dijkstra, A.; Groen, H.; Muller Kobold, A.; Verkade, H.; van Rheenen, P. | 2015 | Comparing the efficacy of a web-assisted calprotectin-based treatment algorithm (IBD-live) with usual practices in teenagers with inflammatory bowel disease: Study protocol for a randomized controlled trial | Duplicate |
| 1382 | Henderson, L. J.; Smulders, T. V.; Roughan, J. V. | 2020 | Identifying obstacles preventing the uptake of tunnel handling methods for laboratory mice: An international thematic survey | Duplicate |
| 1383 | Hengel, B.; Guy, R.; Garton, L.; Ward, J.; Rumbold, A.; Taylor-Thomson, D.; Silver, B.; McGregor, S.; Dyda, A.; Knox, J.; Kaldor, J.; Maher, L. | 2015 | Barriers and facilitators of sexually transmissible infection testing in remote Australian Aboriginal communities: Results from the Sexually Transmitted Infections in Remote Communities, Improved and Enhanced Primary Health Care (STRIVE) Study | Duplicate |

| | | | | |
|---|---|---|---|---|
| 1384 | Hergils, L.; Hergils, Å | 2000 | Univiersal neonatal hearing screening - Parental attitudes and concern | Duplicate |
| 1385 | Hewer, L. A.; Whyatt, D. | 2006 | Improving the implementation of an early literacy program by child health nurses through addressing local training and cultural needs | Duplicate |
| 1386 | Ho, S. S. M.; , Choi, K. C.; , Wong, C. L.; , Chan, C. W. H.; , Chan, H. Y. L.; , Tang, W. P. Y.; , Lam, W. W. T.; , Shiu, A. T. Y.; , Goggins, W. B.; and So, W. K. W. | 2014 | Uptake of breast screening and associated factors among Hong Kong women aged ≥50 years: A population-based survey | Duplicate |
| 1387 | Hoebel, J.; Richter, M.; Lampert, T. | 2013 | Social status and participation in health checks in men and women in germany | Duplicate |
| 1388 | Høj, Kirsten; , Vinther Skriver, Mette; , Maindal, Helle Terkildsen; , Christensen, Bo; and Sandbæk, Annelli | 2018 | The effect of cardiorespiratory fitness assessment in preventive health checks: A randomised controlled trial | Duplicate |
| 1389 | Høj, K.; Vinther Skriver, M.; Terkildsen Maindal, H.; Christensen, B.; Sandbæk, A. | 2018 | The effect of cardiorespiratory fitness assessment in preventive health checks: a randomised controlled trial | Duplicate |
| 1390 | Holzmann, M. J.; Carlsson, A. C.; Hammar, N.; Ivert, T.; Walldius, G.; Jungner, I.; Wändell, P.; Ärnlöv, J. | 2016 | Chronic kidney disease and 10-year risk of cardiovascular death | Duplicate |
| 1391 | Hozawa, A.; Kuriyama, S.; Watanabe, I.; Kakizaki, M.; Ohmori-Matsuda, K.; Sone, T.; Nagai, M.; Sugawara, Y.; Nitta, A.; Li, Q.; Ohkubo, T.; Murakami, Y.; Tsuji, I. | 2010 | Participation in health check-ups and mortality using propensity score matched cohort analyses | Duplicate |
| 1392 | Ingole, J. R.; Patel, R. D.; Ingole, S. I. | 2015 | Opportunistic screening of vitamin b12 deficiency in it professionals presenting for routine health check-up | Duplicate |
| 1393 | Johnson, N.; Lancaster, T.; Fuller, A.; Hodgson, S. V. | 1995 | The prevalence of a family history of cancer in general practice | Duplicate |
| 1394 | Junod, B.; Gutzwiller, F. | 1981 | Swiss National Research Program on the Prevention of Cardiovascular Disease: examination of initial health | Duplicate |
| 1395 | Kamstrup-Larsen, N.; Broholm-Jørgensen, M.; Dalton, S. O.; Larsen, L. B.; Thomsen, J. L.; Tolstrup, J. S. | 2019 | Why do general practitioners not refer patients to behaviour-change programmes after preventive health checks? A mixed-method study | Duplicate |
| 1396 | Kamstrup-Larsen, N.; Dalton, S. O.; Grønbæk, M.; Broholm-Jørgensen, M.; Thomsen, J. L.; Larsen, L. B.; Johansen, C.; Tolstrup, J. | 2019 | The effectiveness of general practice-based health checks on health behaviour and incidence on non-communicable diseases in individuals with low socioeconomic position: A randomised controlled trial in Denmark | Duplicate |
| 1397 | Karefylakis, C.; Näslund, I.; Edholm, D.; Sundbom, M.; Karlsson, F. A.; Rask, E. | 2014 | Prevalence of anemia and related deficiencies 10 years after gastric bypass. A retrospective study | Duplicate |
| 1398 | Kennedy, O.; Su, F.; Pears, R.; Walmsley, E.; Roderick, P. | 2019 | Evaluating the effectiveness of the NHS Health Check programme in South England: A quasi-randomised controlled trial | Duplicate |
| 1399 | Kim, S. H.; Yun, J. M.; Shin, D. W. | 2017 | Prevalence of upper gastrointestinal bleeding risk factors in the osteoar-thritis patients | Duplicate |

| | | | | |
|---|---|---|---|---|
| 1400 | Kishi, H.; Shibata, Y.; Osaka, D.; Abe, S.; Inoue, S.; Tokairin, Y.; Igarashi, A.; Yamauchi, K.; Kimura, T.; Sato, M.; Aida, Y.; Watanabe, T.; Konta, T.; Kawata, S.; Kato, T.; Kubota, I. | 2011 | FEV6 and FEV1/FEV6 in Japanese participants of the community-based annual health check: The Takahata study | Duplicate |
| 1401 | Kon, S.; Konta, T.; Ichikawa, K.; Asahi, K.; Yamagata, K.; Fujimoto, S.; Tsuruya, K.; Narita, I.; Kasahara, M.; Shibagaki, Y.; Iseki, K.; Moriyama, T.; Kondo, M.; Watanabe, T. | 2018 | Association between renal function and cardiovascular and all-cause mortality in the community-based elderly population: Results from the specific health check and guidance program in japan | Duplicate |
| 1402 | Kummer, S.; Waller, J.; Ruparel, M.; Cass, J.; Janes, S. M.; Quaife, S. L. | 2020 | Mapping the spectrum of psychological and behavioural responses to low-dose CT lung cancer screening offered within a Lung Health Check | Duplicate |
| 1403 | Kwok, C.; Fong, D. Y. T. | 2014 | Breast cancer screening practices among hong kong chinese women | Duplicate |
| 1404 | Kypridemos, C.; Collins, B.; McHale, P.; Bromley, H.; Parvulescu, P.; Capewell, S.; O'Flaherty, M. | 2018 | Future cost-effectiveness and equity of the NHS Health Check cardiovascular disease prevention programme: Microsimulation modelling using data from Liverpool, UK | Duplicate |
| 1405 | Langford, A. T.; , Sawyer, D. R.; , Gloimo, S.; and Brownson, C. A. | 2007 | Patient-centered goal setting as a tool to improve diabetes self-management | Duplicate |
| 1406 | Langford, A. T.; Sawyer, D. R.; Gioimo, S.; Brownson, C. A.; O'Toole, M. L. | 2007 | Patient-centered: Goal setting as a tool to improve diabetes self-management | Duplicate |
| 1407 | Larsen, L. B.; , Sondergaard, J.; , Thomsen, J. L.; , Halling, A.; , Sønderlund, A. L.; , Christensen, J. R.; and Thilsing, T. | 2019 | Step-wise approach to prevention of chronic diseases in the Danish primary care sector with the use of a personal digital health profile and targeted follow-up - an assessment of attendance | Duplicate |
| 1408 | Larsen, L. B.; Sondergaard, J.; Thomsen, J. L.; Halling, A.; Sønderlund, A. L.; Christensen, J. R.; Thilsing, T. | 2019 | Digital Recruitment and Acceptance of a Stepwise Model to Prevent Chronic Disease in the Danish Primary Care Sector: Cross-Sectional Study | Duplicate |
| 1409 | Lauritzen, T.; , Leboeufyde, C.; , Lunde, I. M.; and Nielsen, K. D. B. | 1995 | EBELTOFT PROJECT - BASE-LINE DATA FROM A 5-YEAR RANDOMIZED, CONTROLLED, PROSPECTIVE HEALTH PROMOTION STUDY IN A DANISH POPULATION | Duplicate |
| 1410 | Lauritzen, T.; Leboeuf-Yde, C.; Lunde, I. M.; Nielsen, K. D. B. | 1995 | Ebeltoft project: Baseline data from a five-year randomized, controlled, prospective health promotion study in a Danish population | Duplicate |
| 1411 | Lauritzen, T.; Nielsen, K. D.; Leboeuf-Yde, C.; Lunde, I. M. | 1997 | The health project Ebeltoft: health check ups and discussions in general practice. Basic data from a 5-year, prospective, randomized, controlled population study | Duplicate |
| 1412 | Lee, G. R.; Griffin, A.; Halton, K.; Fitzgibbon, M. C. | 2017 | Generating method-specific Reference Ranges – A harmonious outcome? | Duplicate |
| 1413 | Lee, Y. C.; Chien, K. L.; Chen, H. H. | 2007 | Lifestyle risk factors associated with fatique in graduate students | Duplicate |
| 1414 | Lehto, R.; Mäki, P.; Ray, C.; Laatikainen, T.; Roos, E. | 2016 | Childcare use and overweight in Finland: Cross-sectional and retrospective associations among 3- and 5-year-old children | Duplicate |

| | | | | |
|---|---|---|---|---|
| 1415 | Leyk, D.; Rüther, T.; Wunderlich, M.; Sievert, A. P.; Erley, O. M.; Löllgen, H. | 2008 | Utilization and implementation of sports medical screening examinations - Survey of more than 10 000 long-distance runners | Duplicate |
| 1416 | Li, J.; Cao, Y. F.; Sun, X. Y.; Han, L.; Li, S. N.; Gu, W. Q.; Song, M.; Jiang, C. T.; Yang, X.; Fang, Z. Z. | 2019 | Plasma tyrosine and its interaction with low high-density lipoprotein cholesterol and the risk of type 2 diabetes mellitus in Chinese | Duplicate |
| 1417 | Li, L. J.; Zhou, J. X.; Chen, H. T.; Song, Y. L.; Xue, Y. M. | 2012 | Effect of HbA1c combined FPG on screening diabetes in health check-up | Duplicate |
| 1418 | Li, Y. Y.; , Yatsuya, H.; , Iso, H.; , Tamakoshi, K.; and Toyoshima, H. | 2010 | Incidence of metabolic syndrome according to combinations of lifestyle factors among middle-aged Japanese male workers | Duplicate |
| 1419 | Lidin, M.; , Hellénius, M. L.; , Rydell-Karlsson, M.; and Ekblom-Bak, E. | 2018 | Long-term effects on cardiovascular risk of a structured multidisciplinary lifestyle program in clinical practice | Duplicate |
| 1420 | Liersch, S.; Krüger, K.; Oedingen, C.; Spreenberg, A.; Bergemann, T.; Krauth, C. | 2020 | Evaluation of the pediatric-centered integrated care AOK Junior: protocol for a mixed-method study | Duplicate |
| 1421 | Lin, W. S.; , Lee, T. T.; , Yang, Y. H.; and Mills, M. E. | 2019 | Environmental factors affecting self-management of chronic hepatitis B from the patients' perspective | Duplicate |
| 1422 | Liu, P.; Ma, F.; Lou, H.; Liu, Y. | 2013 | The utility of fat mass index vs. body mass index and percentage of body fat in the screening of metabolic syndrome | Duplicate |
| 1423 | Løkkegaard, T.; Andersen, J. S.; Jacobsen, R. K.; Badsberg, J. H.; Jørgensen, T.; Pisinger, C. | 2015 | Psychological consequences of screening for cardiovascular risk factors in an un-selected general population: results from the Inter99 randomised intervention study | Duplicate |
| 1424 | Lowe, C.; Blinkhorn, A. S.; Worthington, H. V.; Craven, R. | 2007 | Testing the effect of including oral health in general health checks for elderly patients in medical practice - A randomized controlled trial | Duplicate |
| 1425 | Malseed, C.; Nelson, A.; Ware, R.; Lacey, I.; Lander, K. | 2014 | Deadly Choices™ community health events: a health promotion initiative for urban Aboriginal and Torres Strait Islander people | Duplicate |
| 1426 | Manikam, L.; Shah, R.; Reed, K.; Santini, G.; Lakhanpaul, M. | 2017 | Using a co-production prioritization exercise involving South Asian children, young people and their families to identify health priorities requiring further research and public awareness | Duplicate |
| 1427 | Mant, D.; , Fuller, A.; , Northover, J.; , Astrop, P.; , Chivers, A.; , Crockett, A.; , Clements, S.; and Lawrence, M. | 1992 | PATIENT COMPLIANCE WITH COLORECTAL-CANCER SCREENING IN GENERAL-PRACTICE | Duplicate |
| 1428 | Martins, E.; Malpeli, A.; Asens, D.; Telese, L.; Fasano, V.; Vargas, V.; Tavellaa, M.; ColmanLerner, J. E. | 2018 | Contribution of diet to lead exposure among children aged 1 to 7 years in la Plata, Buenos Aires | Duplicate |
| 1429 | Martins, E.; Varea, A.; Hernández, K.; Sala, M.; Girardelli, A.; Fasano, V.; Disalvo, L. | 2016 | Blood lead levels in children aged between 1 and 6 years old in la Plata, Argentina. Identification of risk factors for lead exposure | Duplicate |
| 1430 | Mason, M. J.; , Schmidt, C.; , Abraham, A.; , Walker, L.; and Tercyak, K. | 2009 | Adolescents' social environment and depression: social networks, extracurricular activity, and family relationship influences | Duplicate |
| 1431 | McCulloch, B.; McDermott, R.; Miller, G.; Leonard, D.; Elwell, M.; Muller, R. | 2003 | Self-reported diabetes and health behaviors in remote indigenous communities in northern Queensland, Australia | Duplicate |
| 1432 | McMenamin, J. P. | 1992 | HEALTH SCREENING IN A GENERAL-PRACTICE BY OPPORTUNISTIC RECRUITMENT | Duplicate |

| | | | | |
|---|---|---|---|---|
| 1433 | McNaughton, R. J.; Oswald, N. T.; Shucksmith, J. S.; Heywood, P. J.; Watson, P. S. | 2011 | Making a success of providing NHS Health Checks in community pharmacies across the Tees Valley: a qualitative study | Duplicate |
| 1434 | Meena, S.; Quirino, L.; Scheil, W.; Shearing, T.; Nori, A.; Spurrier, N.; Nottage, C. | 2019 | Prevalence of ear disease and hearing loss in aboriginal children living in metropolitan South Australia | Duplicate |
| 1435 | Meepring, S.; Chien, W. T.; Gray, R.; Bressington, D. | 2018 | Effects of the Thai Health Improvement Profile intervention on the physical health and health behaviours of people with schizophrenia: A quasi-experimental study | Duplicate |
| 1436 | Méndez, F. J.; Gómez-Conesa, A. | 2001 | Postural hygiene program to prevent low back pain | Duplicate |
| 1437 | Meng, Q.; Wang, S.; Wang, Y.; Wan, S.; Liu, K.; Zhou, X.; Zhong, G.; Zhang, X.; Chen, X. | 2014 | Arterial stiffness is a potential mechanism and promising indicator of orthostatic hypotension in the general population | Duplicate |
| 1438 | Miller, C. S.; Hart, G. | 1995 | Reduced disease prevalence at a sexually transmitted diseases clinic during a mass media campaign | Duplicate |
| 1439 | Mitsuhashi, Y.; Kishi, R.; Eguchi, T.; Miyake, H.; Maeda, N. | 2003 | Factors associated with participation in medical checkups of the elderly at home comparison of 3 regions with different social backgrounds | Duplicate |
| 1440 | Momo, K.; Yasu, T.; Yasui, H.; Kuroda, S. I. | 2019 | Risk factors affecting the failed low-density lipoprotein level achievement rate in working-age male population at high cardiovascular risk | Duplicate |
| 1441 | Müller, A.; Lamoureux, E.; Bullen, C.; Keeffe, J. E. | 2006 | Factors associated with regular eye examinations in people with diabetes: Results from the Victorian Population Health Survey | Duplicate |
| 1442 | Murashima, M.; Kikuchi, Y.; Nomiyama, T.; Kumagai, N.; Omae, K.; Watanabe, S. | 2004 | Intake and excretion of cadmium and iron balance and influence of dietary habits among young women | Duplicate |
| 1443 | Nagamatsu, Y.; Barroga, E.; Sakyo, Y.; Igarashi, Y.; Hirano O, Y. | 2020 | Risks and perception of non-communicable diseases and health promotion behavior of middle-aged female immigrants in Japan: A qualitative exploratory study | Duplicate |
| 1444 | Nakamura, K.; Watanabe, Y.; Kitamura, K.; Kabasawa, K.; Someya, T. | 2019 | Psychological distress as a risk factor for dementia after the 2004 Niigata–Chuetsu earthquake in Japan | Duplicate |
| 1445 | Nallely, M. M.; Esmeralda, V. V.; Merari, A. C. M.; Gisela, G. F. D.; Josefina, R. V.; Minarda, O. A.; Carlos, L. J. R. | 2014 | Is ingestion of Thasus Gigas (Xamues) an alimentary culture or an auxiliary treatment for type II diabetes? | Duplicate |
| 1446 | Neumann, S.; , Webendörfer, S.; , Lang, S.; , Germann, C.; and Oberlinner, C. | 2015 | [Diabetes screening and prevention in a large chemical company] | Duplicate |
| 1447 | Neumann, S.; Webendörfer, S.; Lang, S.; Germann, C.; Oberlinner, C. | 2015 | Diabetes screening and prevention in a large chemical company | Duplicate |
| 1448 | Nicholas, J. M.; Burgess, C.; Dodhia, H.; Miller, J.; Fuller, F.; Cajeat, E.; Gulliford, M. C. | 2013 | Variations in the organization and delivery of the 'NHS health check' in primary care | Duplicate |
| 1449 | Nigatu, Y. T.; Roelen, C. A. M.; Reijneveld, S. A.; Bültmann, U. | 2015 | Overweight and distress have a joint association with long-term sickness absence among Dutch employees | Duplicate |

| | | | | |
|---|---|---|---|---|
| 1450 | Nilsson, P. M.; Klasson, E. B.; Nyberg, P. | 2001 | Life-style intervention at the worksite - Reduction of cardiovascular risk factors in a randomized study | Duplicate |
| 1451 | Noda, H.; Harada, M.; Yokota, K.; Umesawa, M.; Yamagishi, K.; Cui, R.; Ikeda, A.; Chei, C.; Wakabayashi, Y.; Inagawa, M.; Toriumi, S.; Hirose, K.; Oshima, M.; Shiina, Y.; Tanigawa, T.; Tanaka, K.; Shimamoto, T.; Iso, H. | 2006 | Individualized health education with sports gym use and dietary advice for overweight and obese persons in a community. Kokuho Health-up model Program in Chikusei-shi (former Kyowa town) | Duplicate |
| 1452 | Norman, P. | 1993 | PREDICTING THE UPTAKE OF HEALTH CHECKS IN GENERAL-PRACTICE - INVITATION METHODS AND PATIENTS HEALTH BELIEFS | Duplicate |
| 1453 | Okasha, M.; McCarron, P.; McEwen, J.; Davey Smith, G. | 2001 | Age at menarche: Secular trends and association with adult anthropometric measures | Duplicate |
| 1454 | Okuda, N.; Okamura, T.; Kadowaki, T.; Tanaka, T.; Ueshima, H. | 2004 | Weight-control intervention in overweight subjects at high risk of cardiovascular disease: a trial of a public health practical training program in a medical school | Duplicate |
| 1455 | Ørts, L. M.; , Løkke, A.; , Bjerregaard, A. L.; , Maindal, H. T.; , Norman, K.; , Bech, B. H.;  and Sandbæk, A. | 2019 | The effect on participation rates of including focused spirometry information in a health check invitation: a cluster-randomised trial in Denmark | Duplicate |
| 1456 | Ørts, L. M.; Løkke, A.; Bjerregaard, A. L.; Maindal, H. T.; Sandbæk, A. | 2016 | Effect on attendance by including focused information on spirometry in preventive health checks: Study protocol for a randomized controlled trial | Duplicate |
| 1457 | Otaki, Y.; Watanabe, T.; Konta, T.; Watanabe, M.; Fujimoto, S.; Sato, Y.; Asahi, K.; Yamagata, K.; Tsuruya, K.; Narita, I.; Kasahara, M.; Shibagaki, Y.; Iseki, K.; Moriyama, T.; Kondo, M.; Watanabe, T. | 2018 | Effect of hypertension on aortic artery disease-related mortality: 3.8-year nationwide community-based prospective cohort study | Duplicate |
| 1458 | Pahk, K.; Kwon, Y.; Kim, M. K.; Park, S.; Kim, S. | 2020 | Visceral fat metabolic activity evaluated by 18F-FDG PET/CT is associated with osteoporosis in healthy postmenopausal Korean women | Duplicate |
| 1459 | Pálinkás, A.; Sándor, J.; Papp, M.; Kőrösi, L.; Falusi, Z.; Pál, L.; Bélteczki, Z.; Rihmer, Z.; Döme, P. | 2019 | Associations between untreated depression and secondary health care utilization in patients with hypertension and/or diabetes | Duplicate |
| 1460 | Panca, M.; Buszewicz, M.; Strydom, A.; Hassiotis, A.; Welch, C. A.; Hunter, R. M. | 2019 | Resource use and cost of annual health checks in primary care for people with intellectual disabilities | Duplicate |
| 1461 | Park, S. M.;  and Moon, S. S. | 2016 | Elderly Koreans who consider suicide: Role of health care use and financial status | Duplicate |
| 1462 | Park, S. M.; Moon, S. S. | 2016 | Elderly Koreans who consider suicide: Role of healthcare use and financial status | Duplicate |
| 1463 | Patel, N.; Solanki, S.; Patel, P.; Zala, F.; Matroja, K.; Lakhani, C.; Patel, S.; Amarapurkar, D. | 2015 | Hyperhomocysteinemia in non cirrhotic insulin resistant and obese patients with non alcoholic fatty liver disease | Duplicate |

| | | | | |
|---|---|---|---|---|
| 1464 | Pavithra, V.; Sathisha, T. G.; Kasturi, K.; Siva Mallika, D.; Jeevan Amos, S.; Ragunatha, S. | 2015 | Serum levels of metal ions in female patients with breast cancer | Duplicate |
| 1465 | Paz-Zulueta, M.; Álvarez-Paredes, L.; Rodríguez Díaz, J. C.; Parás-Bravo, P.; Andrada Becerra, M. E.; Rodríguez Ingelmo, J. M.; Ruiz García, M. M.; Portilla, J.; Santibañez, M. | 2018 | Prevalence of high-risk HPV genotypes, categorised by their quadrivalent and nine-valent HPV vaccination coverage, and the genotype association with high-grade lesions | Duplicate |
| 1466 | Petter, J.; Reitsma-van Rooijen, M. M.; Korevaar, J. C.; Nielen, M. M. | 2015 | Willingness to participate in prevention programs for cardiometabolic diseases | Duplicate |
| 1467 | Pinnock, C.; Yip, J. L. Y.; Khawaja, A. P.; Luben, R.; Hayat, S.; Broadway, D. C.; Foster, P. J.; Khaw, K.; Wareham, N. | 2016 | Topical Beta-Blockers and Cardiovascular Mortality: Systematic Review and Meta-Analysis with Data from the EPIC-Norfolk Cohort Study | Duplicate |
| 1468 | Pinto, C. G.; Marega, M.; Carvalho, J. A.; Carmona, F. G.; Lopes, C. E.; Ceschini, F. L.; Bocalini, D. S.; Figueira Junior, A. J. | 2015 | Physical activity as a protective factor for development of non-alcoholic fatty liver in men | Duplicate |
| 1469 | Pinzón, E. M.; Epidemiol, M.; Bravo, S. M.; Epidemiol, M.; Méndez, F.; Clavijo, G. M.; León, M. E. | 2008 | Prevalence and factors related with the presence of oral manifestations in HIV/AIDS patients who attended health institutions in Cali, Colombia | Duplicate |
| 1470 | Platen, P.; Schaar, B. | 2003 | How to carry out a health-orientated marathon training programme for running and inline skating | Duplicate |
| 1471 | Priest, S. R.; Austin, M. P.; Barnett, B. B.; Buist, A. | 2008 | A psychosocial risk assessment model (PRAM) for use with pregnant and postpartum women in primary care settings | Duplicate |
| 1472 | Rahman, M.; Simmons, R. K.; Harding, A. H.; Wareham, N. J.; Griffin, S. J. | 2008 | A simple risk score identifies individuals at high risk of developing Type 2 diabetes: A prospective cohort study | Duplicate |
| 1473 | Rasmussen, S. R.; Thomsen, J. L.; Kilsmark, J.; Hvenegaard, A.; Engberg, M.; Lauritzen, T.; Søgaard, J. | 2007 | Preventive health screenings and health consultations in primary care increase life expectancy without increasing costs | Duplicate |
| 1474 | Reach, G.; Michault, A.; Bihan, H.; Paulino, C.; Cohen, R.; Le Clésiau, H. | 2011 | Patients' impatience is an independent determinant of poor diabetes control | Duplicate |
| 1475 | Richter, M.; , Brand, H.;  and Rössler, G. | 2002 | [Socio-economic differences in the utilisation of screening programmes and health promotion measures in North Rhine-Westphalia, Germany] | Duplicate |
| 1476 | Richter, M.; Brand, H.; Rössler, G. | 2002 | Socio-economic differences in the utilisation of screening programmes and health promotion measures in North Rhine-Westphalia, Germany | Duplicate |
| 1477 | Rieck, T.; Feig, M.; Deleré, Y.; Wichmann, O. | 2014 | Utilization of administrative data to assess the association of an adolescent health check-up with human papillomavirus vaccine uptake in Germany | Duplicate |
| 1478 | Roberts, D. J.; de Souza, V. C. | 2016 | A venue-based analysis of the reach of a targeted outreach service to deliver opportunistic community NHS Health Checks to ‘hard-to-reach’ groups | Duplicate |

| | | | | |
|---|---|---|---|---|
| 1479 | Robertson, G.; Fleming, A.; Williams, M. C.; Trucco, E.; Quinn, N.; Hogg, R.; McKay, G. J.; Kee, F.; Young, I.; Pellegrini, E.; Newby, D. E.; Van Beek, E. J. R.; Peto, T.; Dhillon, B.; Van Hemert, J.; MacGillivray, T. J. | 2020 | Association between hypertension and retinal vascular features in ultra-widefield fundus imaging | Duplicate |
| 1480 | Robroek, Suzan J. W.; , Brouwer, Wendy; , Lindeboom, Dennis; , Oenema, Anke; and Burdorf, Alex | 2010 | Demographic, behavioral, and psychosocial correlates of using the website component of a worksite physical activity and healthy nutrition promotion program: A longitudinal study | Duplicate |
| 1481 | Robroek, Suzan J. W.; , Lindeboom, Dennis E. M.; and Burdorf, Alex | 2012 | Initial and sustained participation in an Internet-delivered long-term worksite health promotion program on physical activity and nutrition | Duplicate |
| 1482 | Robroek, Suzan J. W.; , Polinder, Suzanne; , Bredt, Folef J.; and Burdorf, Alex | 2012 | Cost-effectiveness of a long-term Internet-delivered worksite health promotion programme on physical activity and nutrition: A cluster randomized controlled trial | Duplicate |
| 1483 | Ruktanonchai, C. W.; Ruktanonchai, N. W.; Nove, A.; Lopes, S.; Pezzulo, C.; Bosco, C.; Alegana, V. A.; Burgert, C. R.; Ayiko, R.; Charles, A. S. E. K.; Lambert, N.; Msechu, E.; Kathini, E.; Matthews, Z.; Tatem, A. J. | 2016 | Equality in maternal and newborn health: Modelling geographic disparities in utilisation of care in five East African countries | Duplicate |
| 1484 | Ryoo, J. H.; Kim, S. Y.; Oh, C. M.; Park, S. K.; Kim, E.; Park, S. J.; In Yu, J.; Kim, M. G.; Choi, Y. S.; Ko, T. S. | 2015 | The incidental relationship between serum ferritin levels and hypertension | Duplicate |
| 1485 | Sato, K.; Shibata, Y.; Abe, S.; Inoue, S.; Igarashi, A.; Yamauchi, K.; Aida, Y.; Nunomiya, K.; Nakano, H.; Sato, M.; Kimura, T.; Nemoto, T.; Watanabe, T.; Konta, T.; Ueno, Y.; Kato, T.; Kayama, T.; Kubota, I. | 2014 | Association between plasma adiponectin levels and decline in forced expiratory volume in 1 s in a general Japanese population: The takahata study | Duplicate |
| 1486 | Sato, M.; Shibata, Y.; Abe, S.; Inoue, S.; Igarashi, A.; Yamauchi, K.; Aida, Y.; Kishi, H.; Nunomiya, K.; Nakano, H.; Sato, K.; Watanabe, T.; Konta, T.; Ueno, Y.; Kato, T.; Kayama, T.; Kubota, I. | 2012 | Retrospective analysis of the relationship between decline in FEV1 and abdominal circumference in male smokers: The Takahata study | Duplicate |
| 1487 | Sawyer, A.; Kaim, A.; Le, H. N.; McDonald, D.; Mittinty, M.; Lynch, J.; Sawyer, M. | 2019 | The Effectiveness of an App-Based Nurse-Moderated Program for New Mothers With Depression and Parenting Problems (eMums Plus): Pragmatic Randomized Controlled Trial | Duplicate |
| 1488 | Sayan, S.; Pekin, T.; Yıldızhan, B. | 2018 | Relationship between vasomotor symptoms and metabolic syndrome in postmenopausal women | Duplicate |
| 1489 | Schouten, Lianne S.; , Bültmann, Ute; , Heymans, Martijn W.; , Joling, Catelijne I.; , Twisk, Jos W. R.; and Roelen, Corné A. M. | 2016 | Shortened version of the work ability index to identify workers at risk of long-term sickness absence | Duplicate |
| 1490 | Schouten, L. S.; Bültmann, U.; Heymans, M. W.; Joling, C. I.; Twisk, J. W.; Roelen, C. A. | 2016 | Shortened version of the work ability index to identify workers at risk of long-term sickness absence | Duplicate |
| 1491 | Schutze, H.; , Rix, E. F.; , Laws, R. A.; , Passey, M.; , Fanaian, M.; and Harris, M. F. | 2012 | How feasible are lifestyle modification programs for disease prevention in general practice? | Duplicate |

| | | | | |
|---|---|---|---|---|
| 1492 | Schütze, H.; , Rix, E. F.; , Laws, R. A.; , Passey, M.; , Fanaian, M.; and Harris, M. F. | 2012 | How feasible are lifestyle modification programs for disease prevention in general practice? | Duplicate |
| 1493 | Selekler, H. M.; Gökmen, G.; Alvur, T. M.; Steiner, T. J. | 2015 | Productivity losses attributable to headache, and their attempted recovery, in a heavy-manufacturing workforce in Turkey: implications for employers and politicians | Duplicate |
| 1494 | Shahim, B.; Hasselberg, S.; Boldt-Christmas, O.; Gyberg, V.; Mellbin, L.; Rydén, L. | 2018 | Effectiveness of different outreach strategies to identify individuals at high risk of diabetes in a heterogeneous population: a study in the Swedish municipality of Södertälje | Duplicate |
| 1495 | Shiotani, A.; Miyanishi, T.; Uedo, N.; Iishi, H. | 2005 | Helicobacter pylori infection is associated with reduced circulating ghrelin levels independent of body mass index | Duplicate |
| 1496 | Sjøgaard, G.; Justesen, J. B.; Murray, M.; Dalager, T.; Søgaard, K. | 2014 | A conceptual model for worksite intelligent physical exercise training--IPET--intervention for decreasing life style health risk indicators among employees: a randomized controlled trial | Duplicate |
| 1497 | Skaaby, T.; Jørgensen, T.; Linneberg, A. | 2017 | Effects of invitation to participate in health surveys on the incidence of cardiovascular disease: A randomized general population study | Duplicate |
| 1498 | Skaaby, T.; Jørgensen, T.; Linneberg, A. | 2018 | A randomized general population study of the effects of repeated health checks on incident diabetes | Duplicate |
| 1499 | Smith, S.; Yeomans, D.; Bushe, C. J. P.; Eriksson, C.; Harrison, T.; Holmes, R.; Mynors-Wallis, L.; Oatway, H.; Sullivan, G. | 2007 | A well-being programme in severe mental illness. Baseline findings in a UK cohort | Duplicate |
| 1500 | Sofue, T.; Okano, Y.; Matsushita, N.; Moritoki, M.; Nishijima, Y.; Fujioka, H.; Yamasaki, Y.; Yamanaka, M.; Nishiyama, A.; Minamino, T. | 2019 | The effects of a participatory structured group educational program on the development of CKD: a population-based study | Duplicate |
| 1501 | Sohn, H. S.; Kim, J. R.; Ryu, S. Y.; Lee, Y. J.; Lee, M. J.; Min, H. J.; Lee, J.; Choi, H. Y.; Song, Y. J.; Ki, M. | 2016 | Risk factors for hepatitis C virus (HCV) infection in areas with a high prevalence of HCV in the Republic of Korea in 2013 | Duplicate |
| 1502 | Spyckerelle, Y.; Steinmetz, J.; Fournier, B.; Giordanella, J. P.; Boulangé, M.; De Talencé, N. | 2000 | Borderline hypothyroidism: Epidemiological aspects in women aged 45-70 years | Duplicate |
| 1503 | Steinmetz, J.; Spyckerelle, Y.; Henny, J.; Giordanella, J. P.; Emmanuelli, J. | 2001 | Screening for colorectal cancer | Duplicate |
| 1504 | Stol, Y. H.; Asscher, E. C. A.; Schermer, M. H. N. | 2017 | What is a good health check? An interview study of health check providers' views and practices | Duplicate |
| 1505 | Stoupa, A.; Goischke, A.; Garcin, C.; Elie, C.; Viaud, M.; Théry, A.; Richard, G.; Polak, M. | 2015 | Can school health check-ups serve as screening tool for growth anomalies and obesity in children? | Duplicate |
| 1506 | Strandberg, T. E.; Salomaa, V. V.; Vanhanen, H. T.; Naukkarinen, V. A.; Sarna, S. J.; Miettinen, T. A. | 1995 | Mortality in participants and non-participants of a multifactorial prevention study of cardiovascular diseases: A 28 year follow up of the Helsinki Businessmen Study | Duplicate |
| 1507 | Sugiyama, T.; Tamiya, N.; Watanabe, T.; Wakui, T.; Shibayama, T.; Moriyama, Y.; Yamaoka, Y.; Noguchi, H. | 2018 | Association of care recipients' care-need level with family caregiver participation in health check-ups in Japan | Duplicate |

| | | | | |
|---|---|---|---|---|
| 1508 | Swensen, E.; Reiten, T.; Eriksen, O. | 1994 | Intervention in a high-risk group for cardiovascular disease in Selfjord and Kviteseid. National ambitions transferred to municipal reality | Duplicate |
| 1509 | Tajfard, M.; Ghayour Mobarhan, M.; Rahimi, H. R.; Mouhebati, M.; Esmaeily, H.; Ferns, G. A.; Latiff, L. A.; Taghipour, A.; Mokhber, N.; Fazli Abdul-Aziz, A. | 2014 | Anxiety, depression, coronary artery disease and diabetes mellitus; An association study in Ghaem hospital, Iran | Duplicate |
| 1510 | Takashima, S. I.; Usui, S.; Kurokawa, K.; Kitano, T.; Kato, T.; Murai, H.; Furusho, H.; Oda, H.; Maruyama, M.; Nagata, Y.; Usuda, K.; Kubota, K.; Takeshita, Y.; Sakai, Y.; Honda, M.; Kaneko, S.; Takamura, M. | 2016 | Altered gene expression in T-cell receptor signalling in peripheral blood leucocytes in acute coronary syndrome predicts secondary coronary events | Duplicate |
| 1511 | Thompson, C. N.; Anders, K. L.; Nhi, le T. Q.; Tuyen, H. T.; Van Minh, P.; Tu, le T. P.; Nhu, Td o H.; Nhan, N. T.; Ly, T. T.; Duong, V. T.; Vi, L. L.; Van Thuy, N. T.; Hieu, N. T.; Van Chau, N. V.; Campbell, J. I.; Thwaites, G.; Simmons, C.; Baker, S. | 2014 | A cohort study to define the age-specific incidence and risk factors of Shigella diarrhoeal infections in Vietnamese children: a study protocol | Duplicate |
| 1512 | Tizek, L.; Schielein, M. C.; Seifert, F.; Biedermann, T.; Böhner, A.; Zink, A. | 2019 | Skin diseases are more common than we think: screening results of an unreferred population at the Munich Oktoberfest | Duplicate |
| 1513 | Tominaga, R.; Fukuma, S.; Yamazaki, S.; Sekiguchi, M.; Otani, K.; Kikuchi, S. I.; Sasaki, S.; Kobayashi, S.; Fukuhara, S.; Konno, S. I. | 2016 | Relationship between kyphotic posture and falls in community-dwelling men and women | Duplicate |
| 1514 | Tsukuya, G.; Matsumoto, K.; Fukuyama, S.; Crawford, B.; Nakanishi, Y.; Ichinose, M.; Machida, K.; Samukawa, T.; Ninomiya, T.; Kiyohara, Y.; Inoue, H. | 2015 | Validation of a COPD screening questionnaire and establishment of diagnostic cut-points in a Japanese general population: The Hisayama study | Duplicate |
| 1515 | Unger, M. D.; Cuppari, L.; Titan, S. M.; Magalhães, M. C. T.; Sassaki, A. L.; dos Reis, L. M.; Jorgetti, V.; Moysés, R. M. A. | 2010 | Vitamin D status in a sunny country: Where has the sun gone? | Duplicate |
| 1516 | Van de Poppe, D. J.; Hulzebos, E.; Takken, T. | 2019 | Reference values for maximum work rate in apparently healthy Dutch/Flemish adults: data from the LowLands fitness registry | Duplicate |
| 1517 | Van Rhenen, W.; , Blonk, R. W. B.; , van der Klink, J. J. L.; , van Dijk, F. J. H.; and Schaufeli, W. B. | 2005 | The effect of a cognitive and a physical stress-reducing programme on psychological complaints | Duplicate |
| 1518 | Van Rhenen, W.; Blonk, R. W. B.; van der Klink, J. J.; van Dijk, F. J.; Schaufeli, W. B. | 2005 | The effect of a cognitive and a physical stress-reducing programme on psychological complaints | Duplicate |
| 1519 | Van Tam, V.; Larsson, M.; Pharris, A.; Diedrichs, B.; Nguyen, H. P.; Nguyen, C. T. K.; Ho, P. D.; Marrone, G.; Thorson, A. | 2012 | Peer support and improved quality of life among persons living with HIV on antiretroviral treatment: A randomised controlled trial from north-eastern Vietnam | Duplicate |
| 1520 | Vanhala, M.; Kumpula, L. S.; Soininen, P.; Kangas, A. J.; Ala-Korpela, M.; Kautiainen, H.; Mäntyselkä, P.; Saltevo, J. | 2011 | High serum adiponectin is associated with favorable lipoprotein subclass profile in 6.4-year follow-up | Duplicate |
| 1521 | Viitasalo, K.; Hemiö, K.; Puttonen, S.; Hyvärinen, H. K.; Leiviskä, J.; Härmä, M.; Peltonen, M.; Lindström, J. | 2015 | Prevention of diabetes and cardiovascular diseases in occupational health care: Feasibility and effectiveness | Duplicate |

| | | | | |
|---|---|---|---|---|
| 1522 | Viitasalo, K.; Lindström, J.; Hemiö, K.; Puttonen, S.; Koho, A.; Härmä, M.; Peltonen, M. | 2012 | Occupational health care identifies risk for type 2 diabetes and cardiovascular disease | Duplicate |
| 1523 | Walsh, N.; Barr, O.; Lang, D.; Currid, M.; Hoey, C. | 2019 | Peripheral bone density measurement: An interdisciplinary initiative for improving health outcomes for people with intellectual disabilities in the republic of Ireland | Duplicate |
| 1524 | Walton, S.; Bedford, H. | 2017 | Immunization of looked-after children and young people: a review of the literature | Duplicate |
| 1525 | Wandell, P. E.; , de Waard, A. K. M.; , Holzmann, M. J.; , Gornitzki, C.; , Lionis, C.; , de Wit, N.; , Sondergaard, J.; , Sonderlund, A. L.; , Kral, N.; , Seifert, B.; , Korevaar, J. C.; , Schellevis, F. G.;  and Carlsson, A. C. | 2018 | Barriers and facilitators among health professionals in primary care to prevention of cardiometabolic diseases: A systematic review | Duplicate |
| 1526 | Wändell, P. E.; De Waard, A. K. M.; Holzmann, M. J.; Gornitzki, C.; Lionis, C.; De Wit, N.; Søndergaard, J.; Sønderlund, A. L.; Kral, N.; Seifert, B.; Korevaar, J. C.; Schellevis, F. G.; Carlsson, A. C. | 2018 | Barriers and facilitators among health professionals in primary care to prevention of cardiometabolic diseases: A systematic review | Duplicate |
| 1527 | Wångdahl, J.; Westerling, R.; Lytsy, P.; Mårtensson, L. | 2019 | Perspectives on health examination for asylum seekers in relation to health literacy - focus group discussions with Arabic and Somali speaking participants | Duplicate |
| 1528 | Whicher, C. A.; O'Neill, S.; Holt, R. I. G. | 2020 | Diabetes in the UK: 2019 | Duplicate |
| 1529 | Wichstrøm, Lars; , Berg-Nielsen, Turid Suzanne; , Angold, Adrian; , Egger, Helen Link; , Solheim, Elisabet;  and Sveen, Trude Hamre | 2012 | Prevalence of psychiatric disorders in preschoolers | Duplicate |
| 1530 | Wood, D. A.; Kinmonth, A. L.; Davies, G.; Yarwood, J.; Thompson, S. G.; Pyke, S. D. M.; Kok, Y.; Cramb, R.; Le Guen, C. | 1994 | British family heart study: Its design and method, and prevalence of cardiovascular risk factors | Duplicate |
| 1531 | Wu, P. P.; , Chen, Q. C.; , Chen, L. L.; , Zhang, P. P.; , Xiao, J.; , Chen, X. X.; , Liu, M.;  and Wang, S. M. | 2017 | Dose-Response Relationship between Alanine Aminotransferase Levels within the Reference Interval and Metabolic Syndrome in Chinese Adults | Duplicate |
| 1532 | Yanagisawa, A.; Suzuki, K.; Kimura, A.; Ito, Y.; Hamajima, N.; Inoue, T. | 2009 | Possible protective effect of serum β-carotene levels on the association between interleukin-1B C-31T polymorphism and hypertension in a Japanese population | Duplicate |
| 1533 | Yang, W. S.; Chen, P. C.; Hsu, H. C.; Su, T. C.; Lin, H. J.; Chen, M. F.; Lee, Y. T.; Chien, K. L. | 2018 | Differential effects of saturated fatty acids on the risk of metabolic syndrome: a matched case-control and meta-analysis study | Duplicate |
| 1534 | Yip, J. L. Y.; Broadway, D. C.; Luben, R.; Garway-Heath, D. F.; Hayat, S.; Dalzell, N.; Lee, P. S.; Bhaniani, A.; Wareham, N. J.; Khaw, K. T.; Foster, P. J. | 2011 | Physical activity and Ocular perfusion pressure: The EPIC-Norfolk eye study | Duplicate |

| | | | | |
|---|---|---|---|---|
| 1535 | Yokobayashi, K.; , Kawachi, I.; , Kondo, K.; , Kondo, N.; , Nagamine, Y.; , Tani, Y.; , Shirai, K.; , Tazuma, S.; and Grp, Jages | 2017 | Association between Social Relationship and Glycemic Control among Older Japanese: JAGES Cross-Sectional Study | Duplicate |
| 1536 | Yokobayashi, K.; Kawachi, I.; Kondo, K.; Kondo, N.; Nagamine, Y.; Tani, Y.; Shirai, K.; Tazuma, S.; Kondo, K.; Hanazato, M.; Hikichi, H.; Miyaguni, Y.; Sasaki, Y.; Nagamine, Y.; Ashida, T.; Kondo, N.; Takagi, D.; Tani, Y.; Aida, J.; Osaka, K.; Tsuboya, T.; Jeong, S.; Murata, C.; Saito,; Ojima, T.; Okada, E.; Saito, M.; Hirai, H.; Misawa, J.; Suzuki, K.; Takeda, T.; Yamamoto, T.; Nakade, M.; Cable, N.; Tamakoshi, A.; Fujino, Y.; Shobugawa, Y.; Hayashi, T. | 2017 | Association between social relationship and glycemic control among older Japanese: JAGES cross-sectional study | Duplicate |
| 1537 | Yoshida, H.; Nishi, M.; Watanabe, N.; Fujiwara, Y.; Fukaya, T.; Ogawa, K.; Kim, M. J.; Lee, S.; Shinkai, S. | 2012 | Predictors of frailty development in a general population of older adults in Japan using the frailty index for Japanese elderly patients | Duplicate |
| 1538 | Young, H.; Burke, L.; Nic Gabhainn, S. | 2018 | Sexual intercourse, age of initiation and contraception among adolescents in Ireland: findings from the Health Behaviour in School-aged Children (HBSC) Ireland study | Duplicate |
| 1539 | Zhang, Y.; Ji, M.; Zou, J.; Yuan, T.; Deng, J.; Yang, L.; Li, M.; Qin, H.; Chen, J.; Lin, Q. | 2018 | Effect of a conditional cash transfer program on nutritional knowledge and food practices among caregivers of 3–5-year-old left-behind children in the rural Hunan province | Duplicate |
| 1540 | Zhu, H. L.; , Liang, X. Y.; , Pan, X. F.; , Huang, C. Y.; , Kuang, J.; , Lv, W. B.; , Zeng, Q. C.; , Mai, W. Y.; and Huang, Y. L. | 2020 | A prospective cohort study of home blood pressure monitoring based on an intelligent cloud platform (the HBPM-iCloud study): rationale and design | Duplicate |
| 1541 | Ziebland, S.; Thorogood, M.; Yudkin, P.; Jones, L.; Coulter, A. | 1998 | Lack of willpower or lack of wherewithal? 'Internal' and 'external' barriers to changing diet and exercise in a three year follow-up of participants in a health check | Duplicate |
| 1542 | Zulkiewicz, B. A.; , Burrus, O.; , Harshbarger, C.; , Ortiz, A.; , Garner, B. R.; and Lewis, M. A. | 2021 | Identifying Implementation Strategies That Address Barriers and Facilitate Implementation of Digital Interventions in HIV Primary Care Settings: Results from the Pilot Implementation of Positive Health Check | Duplicate |

**Literature search: August 2020**
**Records assessed at full text level and the excluded reasons**

| # | Author | Year | Title | Excluded with reason |
|---|---|---|---|---|
| 1 | Edwards, L. A.; , Campbell, P.; , Taylor, D. J.; , Shah, R.; , Edgar, D. F.; and Crabb, D. P. | 2019 | Healthy shopper? Blood pressure testing in a shopping centre Pop-Up in England | A |
| 2 | Groenenberg, I.; , Crone, M. R.; , van Dijk, S.; , Ben Meftah, J.; , Middelkoop, B. J. C.; , Assendelft, W. J. J.; and Stiggelbout, A. M. | 2015 | Response and participation of underserved populations after a three-step invitation strategy for a cardiometabolic health check | A |
| 3 | Lowrie, R.; McConnachie, A.; Williamson, A. E.; Kontopantelis, E.; Forrest, M.; Lannigan, N.; Mercer, S. W.; Mair, F. S. | 2017 | Incentivised chronic disease management and the inverse equity hypothesis: Findings from a longitudinal analysis of Scottish primary care practice-level data | A |
| 4 | Spurling, G.; and Hayman, N. | 2010 | Self-rated health status in an urban indigenous primary care setting: implications for clinicians and public health policy | A |
| 5 | Cheong, A. T.; Chinna, K.; Khoo, E. M.; Liew, S. M. | 2017 | Determinants for cardiovascular disease health check questionnaire: A validation study | B |
| 6 | Conner, M.; Godin, G.; Norman, P.; Sheeran, P. | 2011 | Using the Question-Behavior Effect to Promote Disease Prevention Behaviors: Two Randomized Controlled Trials | B |
| 7 | Emslie, M.; Campbell, M.; Walker, K.; Campbell, A.; Farmer, J. | 1996 | Health check-ups in general practice: a patient perspective | B |
| 8 | Enriquez-Haass, Vilma Lucrecia | 2011 | Access and utilization of health services among Latino immigrant male day laborers in the United States | B |
| 9 | Larsen, C. G.; , Jorgensen, K. J.; and Gotzsche, P. C. | 2012 | Regular Health Checks: Cross-Sectional Survey | B |
| 10 | Shimoda, A.; Ichikawa, D.; Oyama, H. | 2018 | Prediction models to identify individuals at risk of metabolic syndrome who are unlikely to participate in a health intervention program | B |
| 11 | Sinclair, A.; Alexander, H. A. | 2012 | Using outreach to involve the hard-to-reach in a health check: What difference does it make? | B |
| 12 | Sommer, I.; Titscher, V.; Gartlehner, G. | 2018 | Participants' expectations and experiences with periodic health examinations in Austria - a qualitative study | B |
| 13 | Thomsen, J. L.; Parner, E. T.; Karlsmose, B.; Thulstrup, A. M.; Lauritzen, T.; Engberg, M. | 2005 | Effect of preventive health screening on long-term primary health care utilization. A randomized controlled trial | B |
| 14 | (no author details; but here is the origianl paper, this reference indicates the correction to this paper) | 2017 | Erratum: NHS Health Check comorbidity and management: An observational matched study in primary care (British Journal of General Practice (2017) DOI: 10.3399/bjgp16X688837) | C |

| | | | | |
|---|---|---|---|---|
| 15 | Burgess, C.; Wright, A. J.; Forster, A. S.; Dodhia, H.; Miller, J.; Fuller, F.; Cajeat, E.; Gulliford, M. C. | 2015 | Influences on individuals' decisions to take up the offer of a health check: a qualitative study | C |
| 16 | Cassidy, G.; Martin, D. M.; Martin, G. H. B.; Roy, A. | 2002 | Health checks for people with learning disabilities: Community learning disability teams working with general practitioners and primary health care teams | C |
| 17 | Corlett, S. A.; and Krska, J. | 2016 | Evaluation of NHS Health Checks provided by community pharmacies | C |
| 18 | Damman, O. C.; , van der Beek, A. J.; and Timmermans, D. R. M. | 2015 | Employees are ambivalent about health checks in the occupational setting | C |
| 19 | de Waard, A. K. M.; , Wandell, P. E.; , Holzmann, M. J.; , Korevaar, J. C.; , Hollander, M.; , Gornitzki, C.; , de Wit, N. J.; , Schellevis, F. G.; , Lionis, C.; , Sondergaard, J.; , Seifert, B.; , Carlsson, A. C.; and Grp, Spimeu Res | 2018 | Barriers and facilitators to participation in a health check for cardiometabolic diseases in primary care: A systematic review | C |
| 20 | Digiacomo, M.; Abbott, P.; Davison, J.; Moore, L.; Davidson, P. M. | 2010 | Facilitating uptake of Aboriginal Adult Health Checks through community engagement and health promotion | C |
| 21 | Dowell, A. C.; , Ochera, J. J.; , Hilton, S. R.; , Bland, J. M.; , Harris, T.; , Jones, D. R.; and Katbamna, S. | 1996 | Prevention in practice: results of a 2-year follow-up of routine health promotion interventions in general practice | C |
| 22 | Dryden, R.; , Williams, B.; , McCowan, C.; and Themessl-Huber, M. | 2012 | What do we know about who does and does not attend general health checks? Findings from a narrative scoping review | C |
| 23 | Dubey, V.; Mathew, R.; Iglar, K.; Moineddin, R.; Glazier, R. | 2006 | Improving preventive service delivery at adult complete health check-ups: The Preventive health Evidence-based Recommendation Form (PERFORM) cluster randomized controlled trial | C |
| 24 | Eastwood, Sophie V.; , Rait, Greta; , Bhattacharyya, Mimi; , Nair, Devaki R.; and Walters, Kate | 2013 | Cardiovascular risk assessment of South Asian populations in religious and community settings: A qualitative study | C |
| 25 | El-Osta, A.; Woringer, M.; Pizzo, E.; Verhoef, T.; Dickie, C.; Ni, M. Z.; Huddy, J. R.; Soljak, M.; Hanna, G. B.; Majeed, A. | 2017 | Does use of point-of-care testing improve cost-effectiveness of the NHS Health Check programme in the primary care setting? A cost-minimisation analysis | C |
| 26 | Fullard, E.; Fowler, G.; Gray, M. | 1987 | Promoting prevention in primary care: Controlled trial of low technology, low cost approach | C |
| 27 | Geue, C.; Lewsey, J. D.; MacKay, D. F.; Antony, G.; Fischbacher, C. M.; Muirie, J.; McCartney, G. | 2016 | Scottish Keep Well health check programme: an interrupted time series analysis | C |
| 28 | Gidlow, C.; Ellis, N.; Randall, J.; Cowap, L.; Smith, G.; Iqbal, Z.; Kumar, J. | 2015 | Method of invitation and geographical proximity as predictors of NHS Health Check uptake | C |
| 29 | Godefrooij, M.; Spigt, M.; van der Minne, W.; Jurrissen, G.; Dinant, G. J.; Knottnerus, A. | 2014 | Implementing cardiometabolic health checks in general practice: a qualitative process evaluation | C |

| | | | | |
|---|---|---|---|---|
| 30 | Gray, B. J.; Bracken, R. M.; Thomas, M.; Williams, S. P.; Williams, M.; Rice, S.; Stephens, J. W. | 2014 | 'Prosiect Sir Gâr': workplace-based cardiovascular disease and diabetes risk assessments | C |
| 31 | Hanlon, P.; , Carey, L.; , Tannahill, C.; , Kelly, M.; , Gilmour, H.; , Tannahill, A.; and McEwen, J. | 1998 | Behaviour change following a workplace health check: how much change occurs and who changes? | C |
| 32 | Hardy, S.; , Deane, K.; and Gray, R. | 2012 | The Northampton Physical Health and Wellbeing Project: the views of patients with severe mental illness about their physical health check | C |
| 33 | Hardy, Sheila; and Gray, Richard | 2012 | Is the use of an invitation letter effective in prompting patients with severe mental illness to attend a primary care physical health check? | C |
| 34 | Hawking, M. K. D.; Timmis, A.; Wilkins, F.; Potter, J. L.; Robson, J. | 2019 | Improving cardiovascular disease risk communication in NHS Health Checks: a qualitative study | C |
| 35 | Hills, O.; Shah, D. | 2020 | Online health information seeking, medical care beliefs and timeliness of medical check-ups among African Americans | C |
| 36 | Ismail, H.; Atkin, K. | 2016 | The NHS Health Check programme: insights from a qualitative study of patients | C |
| 37 | Kamstrup-Larsen, N.; , Broholm-Jorgensen, M.; , Dalton, S. O.; , Larsen, L. B.; , Thomsen, J. L.; and Tolstrup, J. S. | 2019 | Why do general practitioners not refer patients to behaviour-change programmes after preventive health checks? A mixed-method study | C |
| 38 | Khanal, S.; Lloyd, B.; Rissel, C.; Portors, C.; Grunseit, A.; Indig, D.; Ibrahim, I.; McElduff, S. | 2016 | Evaluation of the implementation of Get Healthy at Work, a workplace health promotion program in New South Wales, Australia | C |
| 39 | Kirkcaldy, A. J.; Robinson, J. E.; Perkins, E. S.; Forrest, D. | 2011 | Older men's experiences of community-based health checks in Knowsley, UK | C |
| 40 | Krska, J.; du Plessis, R.; Chellaswamy, H. | 2015 | Views and experiences of the NHS Health Check provided by general medical practices: cross-sectional survey in high-risk patients | C |
| 41 | Larsen, L. B.; , Sondergaard, J.; , Thomsen, J. L.; , Halling, A.; , Sonderlund, A. L.; , Christensen, J. R.; and Thilsing, T. | 2019 | Digital Recruitment and Acceptance of a Stepwise Model to Prevent Chronic Disease in the Danish Primary Care Sector: Cross-Sectional Study | C |
| 42 | Larsen, L. B.; , Thilsing, T.; and Pedersen, L. B. | 2020 | Patient preferences for preventive health checks in Danish general practice: a discrete choice experiment among patients at high risk of noncommunicable diseases | C |
| 43 | Larsen, L. B.; Sonderlund, A. L.; Sondergaard, J.; Thomsen, J. L.; Halling, A.; Hvidt, N. C.; Hvidt, E. A.; Mønsted, T.; Pedersen, L. B.; Roos, E. M.; Pedersen, P. V.; Thilsing, T. | 2018 | Targeted prevention in primary care aimed at lifestyle-related diseases: a study protocol for a non-randomised pilot study | C |

| | | | | |
|---|---|---|---|---|
| 44 | Larsen, L. B.; Sandbaek, A.; Thomsen, J. L.; Bjerregaard, A. L. | 2018 | Uptake of health checks by residents from the Danish social housing sector - a register-based cross-sectional study of patient characteristics in the 'Your Life - Your Health' program | C |
| 45 | Lokkegaard, T.; , Andersen, J. S.; , Jacobsen, R. K.; , Badsberg, J. H.; , Jorgensen, T.; and Pisinger, C. | 2015 | Psychological consequences of screening for cardiovascular risk factors in an un-selected general population: Results from the Inter99 randomised intervention study | C |
| 46 | Martin, A.; , Saunders, C. L.; , Harte, E.; , Griffin, S. J.; , MacLure, C.; , Mant, J.; , Meads, C.; , Walter, F. M.; and Usher-Smith, J. A. | 2018 | Delivery and impact of the NHS Health Check in the first 8 years: a systematic review | C |
| 47 | Meepring, Soontareeporn; , Chien, Wai Tong; , Gray, Richard; and Bressington, Daniel | 2018 | Effects of the Thai Health Improvement Profile intervention on the physical health and health behaviours of people with schizophrenia: A quasi-experimental study | C |
| 48 | Murray, K. A.; Murphy, D. J.; Clements, S. J.; Brown, A.; Connolly, S. B. | 2014 | Comparison of uptake and predictors of adherence in primary and secondary prevention of cardiovascular disease in a community-based cardiovascular prevention programme (MyAction Westminster) | C |
| 49 | Panca, M.; , Buszewicz, M.; , Strydom, A.; , Hassiotis, A.; , Welch, C. A.; and Hunter, R. M. | 2018 | Resource use and cost of annual health checks in primary care for people with intellectual disabilities | C |
| 50 | Perry, C.; , Thurston, M.; , Alford, S.; , Cushing, J.; and Panter, L. | 2016 | The NHS health check programme in England: a qualitative study | C |
| 51 | Persson, M.; Friberg, F. | 2009 | The dramatic encounter: Experiences of taking part in a health conversation | C |
| 52 | Piha, K.; Sumanen, H.; Lahelma, E.; Rahkonen, O. | 2017 | Socioeconomic differences in health check-ups and medically certified sickness absence: a 10-year follow-up among middle-aged municipal employees in Finland | C |
| 53 | Riley, R.; Coghill, N.; Montgomery, A.; Feder, G.; Horwood, J. | 2016 | Experiences of patients and healthcare professionals of NHS cardiovascular health checks: a qualitative study | C |
| 54 | Riley, V. A.; Gidlow, C.; Ellis, N. J. | 2018 | Understanding implementation and uptake in the National Health Service Health Check Programme | C |
| 55 | Roberts, D. J.; and de Souza, V. C. | 2016 | A venue-based analysis of the reach of a targeted outreach service to deliver opportunistic community NHS Health Checks to 'hard-to-reach' groups | C |
| 56 | Romeo, R.; Knapp, M.; Morrison, J.; Melville, C.; Allan, L.; Finlayson, J.; Cooper, S. A. | 2009 | Cost estimation of a health-check intervention for adults with intellectual disabilities in the UK | C |
| 57 | Rosen, D.; , Nakar, S.; , Cohen, A. D.; and Vinker, S. | 2014 | Low rate of non-attenders to primary care providers in Israel - a retrospective longitudinal study | C |

| | | | | |
|---|---|---|---|---|
| 58 | Sabates, R.; Feinstein, L. | 2008 | Do income effects mask social and behavioural factors when looking at universal health care provision? | C |
| 59 | Saramunee, K.; Krska, J.; Mackridge, A.; Richards, J.; Suttajit, S.; Phillips-Howard, P. | 2015 | General public's views on pharmacy public health services: Current situation and opportunities in the future | C |
| 60 | Spurling, G. K. P.; , Askew, D. A.; , Schluter, P. J.; and Hayman, N. E. | 2013 | Implementing computerised Aboriginal and Torres Strait Islander health checks in primary care for clinical care and research: a process evaluation | C |
| 61 | Stol, Y. H.; Asscher, E. C. A.; Schermer, M. H. N. | 2018 | Good health checks according to the general public; expectations and criteria: a focus group study | C |
| 62 | White, J.; Lucas, J.; Swift, L.; Barton, G. R.; Johnson, H.; Irvine, L.; Abotsie, G.; Jones, M.; Gray, R. J. | 2018 | Nurse-facilitated health checks for persons with severe mental illness: A cluster-randomized controlled trial | C |
| 63 | Wu, T. Y.; Chen, Y. A.; Liu, W. L.; Majeed, A. | 2015 | Differences in mortality rates between frequent and occasional participants of periodic health check-ups: An observational study and propensity analysis | C |
| 64 | Brangan, Emer; , Stone, Tracey J.; , Chappell, Amanda; , Harrison, Vivienne; and Horwood, Jeremy | 2018 | Patient experiences of telephone outreach to enhance uptake of nhs health checks in more deprived communities and minority ethnic groups: A qualitative interview study | C |
| 65 | O'Neill, C. | 1994 | The OXCHECK Study: a nursing perspective. Oxford and Collaborators Health Check | C |
| 66 | Abuduxike, G.; Aşut, Ö; Vaizoğlu, S. A.; Cali, S. | 2020 | Health-Seeking Behaviors and its Determinants: A Facility-Based Cross-Sectional Study in the Turkish Republic of Northern Cyprus | C |
| 67 | Kondo, Naoki; and Ishikawa, Yoshiki | 2018 | Affective stimuli in behavioural interventions soliciting for health check-up services and the service users' socioeconomic statuses: A study at Japanese pachinko parlours | D |
| 68 | Malseed, C.; , Nelson, A.; , Ware, R.; , Lacey, I.; and Lander, K. | 2014 | Deadly Choices (TM) community health events: a health promotion initiative for urban Aboriginal and Torres Strait Islander people | D |
| 69 | Barwell, P. | 2009 | Do invitations to attend Well Man Checks result in increased male health screening in primary health care? | E |
| 70 | McMenamin, J. P. | 1992 | Health screening in a general practice by opportunistic recruitment | E |
| 71 | Brand, T.; , Kleer, D.; , Samkange-Zeeb, F.; and Zeeb, H. | 2015 | [Prevention among migrants: Participation, migrant sensitive strategies and programme characteristics] | F |
| 72 | Junod, B.; and Gutzwiller, F. | 1981 | [Swiss National Research Program on the Prevention of Cardiovascular Disease: examination of initial health] | F |

| | | | | |
|---|---|---|---|---|
| 73 | Richter, M.; , Brand, H.; and Rossler, G. | 2002 | Socio-economic differences in the utilisation of screening programmes and health promotion measures in North Rhine-Westphalia, Germany | F |
| 74 | Steinert, Tilman; , Breier, Andrea; and Flammer, Erich | 2011 | Wahrnehmung von Vorsorgeuntersuchungen und Arztbesuchen bei Menschen mit Schizophrenie und Menschen ohne psychische Erkrankung mit vergleichbarem Sozialstatus | F |
| 75 | Swensen, E.; , Reiten, T.; and Eriksen, O. | 1994 | [Intervention in a high-risk group for cardiovascular disease in Selfjord and Kviteseid. National ambitions transferred to municipal reality] | F |
| 76 | Burgess, Caroline; , Nicholas, Jennifer; and Gulliford, Martin | 2012 | Impact of an electronic, computer-delivered questionnaire, with or without postal reminders, on survey response rate in primary care | G |
| 77 | Burnett, L.; Burden, A. F. | 2013 | Is uptake of the NHS health checks programme by Indo Asian people less than for other ethnic groups? How many people are found with diabetes? | G |
| 78 | Khayat, Kevin; and Salter, Brian | 1994 | Health promotion in the market-place: Patient satisfaction as the basis of a marketing strategy | G |
| 79 | Martha Cabrera Sierra, M.; Fernandez Meseguer, A.; Cortes Arcas, M. V.; Garcia Margallo, M. T.; Navarro Valle, R. I.; Sanz Martin, C.; Diez Alonso, L. A.; Gutierrez Gonzalez, F.; Rabanal Alonso, R. B.; Calvo Bonacho, E. | 2013 | Lifestyle intervention clinical trial in a labour population with high cardiovascular risk: 12 months follow-up preliminary results | G |
| 80 | Norelius, M.; Perk, J. | 2014 | Cardiovascular risk assessment at the pharmacy may identify the population at risk | G |
| 81 | Panaretto, K. S.; Button, S.; Carson, A.; Leon, D.; Schibasaki, R.; Wason, G.; Baker, D.; Ring, I. | 2012 | Are we there yet? Closing the gap in indigenous health in Australia: Monitoring clinical performance in aboriginal and Islander Community control health services in Queensland | G |
| 82 | Patel, R.; Lagord, C.; Waterall, J.; Moth, M.; Knapton, M.; Deanfield, J. | 2016 | Self-assessment of cardiovascular risk: Public use of the online JBS3 heart age tool | G |
| 83 | Peeters, A.; Pasupathi, K.; Sim, M. | 2013 | The impact of a workplace diabetes prevention screening and follow-up program on productivity | G |
| 84 | Saijpaul, R.; Gupta, S.; Saini, M. | 2017 | Biochemical analysis of employees attending cardiac camp of supreme court of India | G |
| 85 | Stone, M. A.; Taub, N.; Aujla, N.; Davies, M. J.; Farooqi, A.; Khunti, K. | 2011 | The MY-WAIST diabetes screening programme in primary care: Key quantitative findings from the final data set | G |
| 86 | Webendoerfer, S.; Claus, M.; Oberlinner, C. | 2018 | Health checks at the workplace | G |

| | | | | |
|---|---|---|---|---|
| 87 | | 2016 | Invitation method influences NHS Health Check uptake | H |
| 88 | Harvey, S. B.; Newton, A.; Moye, G. A. | 2005 | Physical health monitoring in schizophrenia: The use of an invitational letter in a primary care setting | H |
| 89 | Tan, M. M.; Lee, J. T.; Lorenc, T. | 2017 | Does routine screening for cardiovascular risk factors widen socioeconomic inequalities in health? : A systematic review | H |
| 90 | Lee, K.; Rutledge, M.; Rouse, A.; Burden, A. C. F. | 2013 | What methods did we use to achieve high take-up of the NHS health checks programme (NHSHCP)? | H |
| 91 | McDermott, L.; Cornelius, V.; Wright, A. J.; Burgess, C.; Forster, A. S.; Ashworth, M.; Khoshaba, B.; Clery, P.; Fuller, F.; Miller, J.; Dodhia, H.; Rudisill, C.; Conner, M. T.; Gulliford, M. C. | 2018 | Enhanced Invitations Using the Question-Behavior Effect and Financial Incentives to Promote Health Check Uptake in Primary Care | Duplicate study as included study, but in different format |

## Literature search update: May 2021
## Titles and abstracts screening with excluded reasons

| # | Author | Year | Title | Excluded with reason |
|---|---|---|---|---|
| 1 | Abu Awwad, D.: Hossain, S. Z.: Mackey, M.: Brennan, P.: Adam, S. | 2020 | Exploring the role of healthcare organisations in increasing women's participation in breast-screening in the United Arab Emirates | A |
| 2 | Baek, S.: Kim, G.: Park, H. W. | 2020 | A mobile delivered self-exercise program for female farmers | A |
| 3 | Bartlett, E. C.: Kemp, S. V.: Ridge, C. A.: Desai, S. R.: Mirsadraee, S.: Morjaria, J. B.: Shah, P. L.: Popat, S.: Nicholson, A. G.: Rice, A. J.: Jordan, S.: Begum, S.: Mani, A.: Derbyshire, J.: Morris, K.: Chen, M.: Peacock, C.: Addis, J.: Martins, M.: Kaye, S. B.: Padley, S. P. G.: Devaraj, A.: McDonald, F.: Robertus, J. L.: Lim, E.: Barnett, J.: Finch, J.: Dalal, P.: Yousaf, N.: Jamali, A.: Ivashniova, N.: Phillips, C.: Newsom-Davies, T.: Lee, R.: Vaghani, P.: Whiteside, S.: Vaughan-Smith, S. | 2020 | Baseline Results of the West London lung cancer screening pilot study – Impact of mobile scanners and dual risk model utilisation | A |
| 4 | Begum, J.: Nisar, M. K. | 2020 | Can dedicated community health hubs improve physical activity in a multi-ethnic rheumatology practice? | A |
| 5 | Berger, M.: Taylor, S.: Harriss, L.: Campbell, S.: Thompson, F.: Jones, S.: Makrides, M.: Gibson, R.: Amminger, G. P.: Sarnyai, Z.: McDermott, R. | 2020 | Cross-sectional association of seafood consumption, polyunsaturated fatty acids and depressive symptoms in two Torres Strait communities | A |
| 6 | Boedker, C.: Chong, K. M.: Mouritsen, J. | 2020 | The counter-performativity of calculative practices: Mobilising rankings of intellectual capital | A |
| 7 | Chu, N.: Wu, D.: Lin, Y. | 2020 | Association between chronic disease status and grip strength among middle-age and elderly population in Taiwan: The Chiayi community-based elderly health survey | A |
| 8 | Crosbie, P. A. J.: Gabe, R.: Simmonds, I.: Kennedy, M.: Rogerson, S.: Ahmed, N.: Baldwin, D. R.: Booton, R.: Cochrane, A.: Darby, M.: Franks, K.: Hinde, S.: Janes, S. M.: Macleod, U.: Messenger, M.: Moller, H.: Murray, R. L.: Neal, R. D.: Quaife, S. L.: Sculpher, M.: Tharmanathan, P.: Torgerson, D.: Callister, M. E. J. | 2020 | Yorkshire Lung Screening Trial (YLST): protocol for a randomised controlled trial to evaluate invitation to community-based low-dose CT screening for lung cancer versus usual care in a targeted population at risk | A |
| 9 | Cuschieri, S.: Mamo, J. | 2020 | Are normoglycaemic individuals at risk of depression? The depression-dysglycaemic phenotype from a European population-based cross-sectional study | A |

| | | | | |
|---|---|---|---|---|
| 10 | Desai, A.: Lau, C.: Sutradhar, R.: Lee, D.: Nathan, P.: Gupta, S. | 2020 | Morbidity, mortality, and healthcare use among mothers of children with cancer: A population-based study | A |
| 11 | Dharmayat, K.: Woringer, M.: Mastellos, N.: Cole, D.: Car, J.: Ray, S.: Khunti, K.: Majeed, A.: Ray, K. K.: Seshasai, S. R. K. | 2020 | Investigation of Cardiovascular Health and Risk Factors Among the Diverse and Contemporary Population in London (the TOGETHER Study): Protocol for Linking Longitudinal Medical Records | A |
| 12 | Gilworth, G.: Milton, S.: Chater, A.: Nazareth, I.: Roposch, A.: Green, J. | 2020 | Parents' expectations and experiences of the 6-week baby check: a qualitative study in primary care | A |
| 13 | Hara, M.: Koshida, R.: Araki, K.: Kondo, M.: Hirota, Y. | 2020 | Determinants of self-paid rotavirus vaccination status in Kanazawa, Japan, including socioeconomic factors, parents' perception, and children's characteristics | A |
| 14 | Imai, R.: Imaoka, M.: Nakao, H.: Hida, M.: Tazaki, F.: Omizu, T.: Ishigaki, T.: Nakamura, M. | 2020 | Association between chronic pain and pre-frailty in Japanese community-dwelling older adults: A cross-sectional study | A |
| 15 | Jung, M.: Ihm, S. H.: Han, S.: Jung, H. O.: Youn, H. J.: Ryu, K. H. | 2020 | Sex-specific effect of obesity on exercise capacity and diastolic function | A |
| 16 | Kang, M. K.: Park, J. G.: Kim, M. C. | 2020 | Association between Atrial Fibrillation and Advanced Liver Fibrosis in Patients with Non-Alcoholic Fatty Liver Disease | A |
| 17 | Keng, C. J. S.: Goriawala, A.: Rashid, S.: Goldstein, R.: Schmocker, S.: Easson, A.: Kennedy, E. | 2020 | Home to Stay: An Integrated Monitoring System Using a Mobile App to Support Patients at Home Following Colorectal Surgery | A |
| 18 | Kera, T.: Kawai, H.: Takahashi, J.: Hirano, H.: Watanabe, Y.: Fujiwara, Y.: Ihara, K.: Kim, H.: Obuchi, S. | 2020 | Association between ground reaction force in sit-to-stand motion and falls in community-dwelling older Japanese individuals | A |
| 19 | Kneuertz, P. J.: Jagadesh, N.: Perkins, A.: Fitzgerald, M.: Moffatt-Bruce, S. D.: Merritt, R. E.: D'Souza, D. M. | 2020 | Improving patient engagement, adherence, and satisfaction in lung cancer surgery with implementation of a mobile device platform for patient reported outcomes | A |
| 20 | Kosugi, T.: Eriguchi, M.: Yoshida, H.: Tasaki, H.: Nishimoto, M.: Kasahara, M.: Iseki, K.: Asahi, K.: Yamagata, K.: Konta, T.: Fujimoto, S.: Narita, I.: Shibagaki, Y.: Moriyama, T.: Kondo, M.: Watanabe, T.: Tsuruya, K. | 2020 | Association between CKD and new onset of dyslipidemia: Results from a longitudinal nationwide survey | A |
| 21 | Kuriyama, N.: Ozaki, E.: Koyama, T.: Matsui, D.: Watanabe, I.: Tomida, S.: Nagamitsu, R.: Hashiguchi, K.: Inaba, M.: Yamada, S.: Horii, M.: Mizuno, S.: Yoneda, Y.: Kurokawa, M.: Kobayashi, D.: Fukuda, S.: Iwasa, K.: Watanabe, Y.: Uehara, R. | 2020 | Evaluation of myostatin as a possible regulator and marker of skeletal muscle–cortical bone interaction in adults | A |

| | | | | |
|---|---|---|---|---|
| 22 | Liel, C.: Ulrich, S. M.: Lorenz, S.: Eickhorst, A.: Fluke, J.: Walper, S. | 2020 | Risk factors for child abuse, neglect and exposure to intimate partner violence in early childhood: Findings in a representative cross-sectional sample in Germany | A |
| 23 | Luisa, F. N.: Per, M.: Lynne, T. S.: Emilien, J. | 2020 | Overweight and obesity in 5-to 6-year-old schoolchildren in Switzerland from 2003 to 2018 | A |
| 24 | Middleton, M.: Somerset, S.: Evans, C.: Blake, H. | 2020 | Test@work texts: Mobile phone messaging to increase awareness of HIV and HIV testing in UK construction employees during the COVID-19 pandemic | A |
| 25 | Mweseli, R.: Sadaka, A.: Bartlett, E.: Deveraj, A.: Kemp, S.: Addis, J.: Derbyshire, J.: Chen, M.: Morris, K.: Hopkinson, N. | 2020 | Participation in a targeted lung health check program and smoking cessation | A |
| 26 | Narvaez, L. F.: Mahler, P.: Thadikkaran-Salomon, L.: Jeannot, E. | 2020 | Overweight and obesity in 5- To 6-year-old schoolchildren in Switzerland from 2003 to 2018 | A |
| 27 | O'Brien, K.: Agostino, J.: Ciszek, K.: Douglas, K. | 2020 | Parents' perceptions of their child's weight among kindergarten children | A |
| 28 | Ohara, Y.: Kawai, H.: Shirobe, M.: Motokawa, K.: Fujiwara, Y.: Kim, H.: Ihara, K.: Obuchi, S.: Edahiro, A.: Iwasaki, M.: Watanabe, Y.: Hirano, H. | 2020 | Association between anorexia and hyposalivation in community-dwelling older adults in Japan: a 6-year longitudinal study | A |
| 29 | Otaki, Y.: Watanabe, T. W.: Konta, T. K.: Watanabe, M. W.: Asahi, K. A.: Yamagata, K. Y.: Fujimoto, S. F.: Tsuruya, K. T.: Narita, I. N.: Kasahara, M. K.: Shibagaki, Y. S.: Iseki, K. I.: Moriyama, T. M.: Kondo, M. K.: Watanabe, T. W. | 2020 | Usefulness of Suita score to predict deaths from aortic aneurysm rupture and aortic dissection in general population | A |
| 30 | Otaki, Y.: Watanabe, T.: Konta, T.: Watanabe, M.: Asahi, K.: Yamagata, K.: Fujimoto, S.: Tsuruya, K.: Narita, I.: Kasahara, M.: Shibagaki, Y.: Iseki, K.: Moriyama, T.: Kondo, M.: Watanabe, T. | 2020 | Impact of calculated plasma volume status on all-cause and cardiovascular mortality: 4-year nationwide community-based prospective cohort study | A |
| 31 | Pasdar, Z.: Gamble, D. T.: Myint, P. K.: Luben, R. N.: Wareham, N. J.: Khaw, K. T.: Bhattacharya, S. | 2020 | Hypertensive disorders of pregnancy (HDP) and the risk of common cancers in women: Evidence from the European prospective investigation into cancer (EPIC)-norfolk prospective population-based study | A |
| 32 | Radhika, : Sekar, V.: Devi, D.: Ashok, G.: Chokkalingam, M. | 2020 | Analysis of coronary angiographic profile in positive exercise treadmill test in patients with diabetes mellitus | A |
| 33 | Rein, D.: Claus, M.: Frosch, W.: Marz, W.: Lorkowski, S.: Webendoerfer, S.: Schreiner, T. | 2020 | Changes in Erythrocyte Omega-3 Fatty Acids in German Employees upon Dietary Advice by Corporate Health | A |
| 34 | Runderawala, H.: Desai, N. | 2020 | The triglyceride and glucose index (TyG) as screening biomarker to identify non-alcoholic fatty liver disease | A |
| 35 | Tarpara, K. V.: Khan, E. M.: Basu, A. | 2020 | Age and gender based study of reference range of platelet count, MPV and PDW in healthy eastern Indian population | A |

| | | | | |
|---|---|---|---|---|
| 36 | Terui, T.: Yoshida, K.: Sasaki, M.: Murakami, M.: Goto, A. | 2020 | The Association Between Fathers' Self-Assessment of Their Own Parenting and Mothers' Recognition of Paternal Support: A Municipal-Based Cross-Sectional Study | A |
| 37 | Thuong, N. T. T. | 2020 | Impact of health insurance on healthcare utilisation patterns in Vietnam: a survey-based analysis with propensity score matching method | A |
| 38 | Ueda, K.: Kashiba, A.: Ooue, C.: Kimura, A.: Takeshita, T.: Arita, M. | 2020 | Effects of a home blood pressure monitoring by mobile phone-based and health service (continuous antihypertensive treatment) in mild hypertension : The wakayama health promotion study | A |
| 39 | Urtasun Erburu, A.: Jareño Roglán, E. J.: Albert I Ros, X.: García Tamarit, P.: Llinares Ramal, S.: Segura Barrachina, S.: Ausejo, R. L. C.: Puchades Marqués, E.: Fraile Zamarreño, A. | 2020 | 12-year evolution of breastfeeding indicators and associated factors in a recently accredited as BFHI Health Center | A |
| 40 | Wang, L.: Zhang, T.: Liu, Y. F.: Tang, F.: Xue, F. Z. | 2020 | Association of Serum Uric Acid with Metabolic Syndrome and Its Components: A Mendelian Randomization Analysis | A |
| 41 | Wei, L.: Cheng, X.: Luo, Y.: Yang, R.: Lei, Z.: Jiang, H.: Chen, L. | 2020 | Lean non-alcoholic fatty liver disease and risk of incident diabetes in a euglycaemic population undergoing health check-ups: A cohort study | A |
| 42 | Yahata, Y.: Fielding, J. E.: Kamiya, H.: Takimoto, N.: Ishii, J.: Fukusumi, M.: Sunagawa, T. | 2020 | Factors associated with knowledges and attitudes about measles and rubella immunization in a non-health care occupational setting in Japan | A |
| 43 | Yeap, S. S.: Thambiah, S. C.: Samsudin, I. N.: Appannah, G.: Zainuddin, N.: Mohamad-Ismuddin, S.: Shahifar, N.: Md-Said, S.: Zahari-Sham, S. Y.: Suppiah, S.: Hew, F. L. | 2020 | Different reference ranges affect the prevalence of osteoporosis and osteopenia in an urban adult Malaysian population | A |
| 44 | Zeng, Y.: He, H.: Wang, X.: Zhang, M.: An, Z. | 2020 | Climate and air pollution exposure are associated with thyroid function parameters: a retrospective cross-sectional study | A |
| 45 | Zhao, H.: Zhang, X. N.: Shi, Z.: Yin, L.: Zhang, W. L.: He, K.: Xue, H. Q.: Zhao, X. Y.: Shi, S. H. | 2020 | Association of level of leisure-time physical activity with risks of all-cause mortality and cardiovascular disease in an elderly Chinese population: a prospective cohort study | A |
| 46 | Ahankari, A. S.: Kabra, P.: Tata, L. J.: Hayter, M.: Fogarty, A. W. | 2021 | Two measures of systemic inflammation are positively associated with haemoglobin levels in adolescent girls living in rural India: a cross-sectional study | A |
| 47 | Balata, H.: Ruparel, M.: O'Dowd, E.: Ledson, M.: Janes, S.: Booton, R.: Baldwin, D.: Crosbie, P. | 2021 | MA05.06 Lung Cancer Screening – Cumulative Results from Five UK-Based Programmes | A |
| 48 | Balata, H.: Ruparel, M.: O'Dowd, E.: Ledson, M.: Janes, S.: Booton, R.: Baldwin, D.: Crosbie, P. | 2021 | Lung cancer screening - Cumulative results from five UK-based programmes | A |

| | | | | |
|---|---|---|---|---|
| 49 | Borrull-Guardeno, J.: Sebastia-Laguarda, C.: Donat-Colomer, F.: Sanchez-Martinez, V. | 2021 | Women's knowledge and attitudes towards cervical cancer prevention: A qualitative study in the Spanish context | A |
| 50 | Claus, M.: Antoni, C.: Hofmann, B. | 2021 | Factors associated with elevated alanine aminotransferase in employees of a German chemical company: results of a large cross-sectional study | A |
| 51 | Coombs, N. M.: Missen, K.: Allen, L. | 2021 | Beyond simulation - Extracurricular volunteering in nursing education: A focus group | A |
| 52 | Dickson, J. L.: Quaife, S. L.: Horst, C.: Tisi, S.: Hall, H.: Verghese, P.: Mullin, A.: Sarpong, R.: Teague, J.: Farrelly, L.: Bowyer, V.: Gyertson, K.: Pervez, H.: Bojang, F.: Levermore, C.: Anastasiadis, T.: Sennett, K.: Navani, N.: Hackshaw, A.: Janes, S. M. | 2021 | The summit study: Uptake from re-invitation | A |
| 53 | Han, L.: Doran, T.: Holt, R. I. G.: Hewitt, C.: Jacobs, R.: Prady, S. L.: Alderson, S. L.: Shiers, D.: Wang, H. I.: Bellass, S.: Gilbody, S.: Kitchen, C. E. W.: Lister, J.: Taylor, J.: Siddiqi, N. | 2021 | The impact of severe mental illness on healthcare use and health outcomes for people with type 2 diabetes | A |
| 54 | Harshbarger, C.: Burrus, O.: Rangarajan, S.: Bollenbacher, J.: Zulkiewicz, B.: Verma, R.: Galindo, C. A.: Lewis, M. A. | 2021 | Challenges of and Solutions for Developing Tailored Video Interventions That Integrate Multiple Digital Assets to Promote Engagement and Improve Health Outcomes: Tutorial | A |
| 55 | Herpertz-Dahlmann, B.: Bonin, E.: Dahmen, B. | 2021 | Can you find the right support for children, adolescents and young adults with anorexia nervosa: Access to age-appropriate care systems in various healthcare systems | A |
| 56 | Hozawa, A.: Tanno, K.: Nakaya, N.: Nakamura, T.: Tsuchiya, N.: Hirata, T.: Narita, A.: Kogure, M.: Nochioka, K.: Sasaki, R.: Takanashi, N.: Otsuka, K.: Sakata, K.: Kuriyama, S.: Kikuya, M.: Tanabe, O.: Sugawara, J.: Suzuki, K.: Suzuki, Y.: Kodama, E. N.: Fuse, N.: Kiyomoto, H.: Tomita, H.: Uruno, A.: Hamanaka, Y.: Metoki, H.: Ishikuro, M.: Obara, T.: Kobayashi, T.: Kitatani, K.: Takai-Igarashi, T.: Ogishima, S.: Satoh, M.: Ohmomo, H.: Tsuboi, A.: Egawa, S.: Ishii, T.: Ito, K.: Ito, S.: Taki, Y.: Minegishi, N.: Ishii, N.: Nagasaki, M.: Igarashi, K.: Koshiba, S.: Shimizu, R.: Tamiya, G.: Nakayama, K.: Motohashi, H.: Yasuda, J.: Shimizu, A.: Hachiya, T.: Shiwa, Y.: Tominaga, T.: Tanaka, H.: Oyama, K.: Tanaka, R.: Kawame, H.: Fukushima, A.: Ishigaki, Y.: Tokutomi, T.: Osumi, N.: Kobayashi, T.: Nagami, F.: Hashizume, H.: Arai, T.: Kawaguchi, Y.: Higuchi, S.: Sakaida, M.: Endo, R.: Nishizuka, S.: Tsuji, I.: Hitomi, J.: Nakamura, M.: Ogasawara, K.: Yaegashi, | 2021 | Study Profile of the Tohoku Medical Megabank Community-Based Cohort Study | A |

| | | | | |
|---|---|---|---|---|
| | N.: Kinoshita, K.: Kure, S.: Sakai, A.: Kobayashi, S.: Sobue, K.: Sasaki, M.: Yamamoto, M. | | | |
| 57 | Hulsegge, G.: Proper, K. I.: Loef, B.: Paagman, H.: Anema, J. R.: van Mechelen, W. | 2021 | The mediating role of lifestyle in the relationship between shift work, obesity and diabetes | A |
| 58 | Kaimoto, K.: Yamashita, M.: Suzuki, T.: Makizako, H.: Koriyama, C.: Kubozono, T.: Takenaka, T.: Ohishi, M.: Kanouchi, H.: The Tarumizu Study Diet, Group | 2021 | Association of Protein and Magnesium Intake with Prevalence of Prefrailty and Frailty in Community-Dwelling Older Japanese Women | A |
| 59 | Kitamura, A.: Seino, S.: Abe, T.: Nofuji, Y.: Yokoyama, Y.: Amano, H.: Nishi, M.: Taniguchi, Y.: Narita, M.: Fujiwara, Y.: Shinkai, S. | 2021 | Sarcopenia: prevalence, associated factors, and the risk of mortality and disability in Japanese older adults | A |
| 60 | Kobayashi, K.: Ando, K.: Nakashima, H.: Machino, M.: Kanbara, S.: Ito, S.: Inoue, T.: Yamaguchi, H.: Koshimizu, H.: Ishiguro, N.: Hasegawa, Y.: Imagama, S. | 2021 | Overcoming locomotive syndrome: The Yakumo Study | A |
| 61 | Kumalo, A.: Gambura, E.: Dodicho, T.: Ahmed, K. S.: Balcha, T.: Beshir, B.: Abraham, M. | 2021 | Prevalence of Intestinal Parasites and Salmonella typhi among Food Handlers Working in Catering Establishments of Public Institutes Found in Dawuro Zone, South-Western Ethiopia | A |
| 62 | Kuriyama, N.: Ozaki, E.: Koyama, T.: Matsui, D.: Watanabe, I.: Tomida, S.: Nagamitsu, R.: Hashiguchi, K.: Inaba, M.: Yamada, S.: Horii, M.: Mizuno, S.: Yoneda, Y.: Kurokawa, M.: Kobayashi, D.: Fukuda, S.: Iwasa, K.: Watanabe, Y.: Uehara, R. | 2021 | Evaluation of myostatin as a possible regulator and marker of skeletal muscle-cortical bone interaction in adults | A |
| 63 | Leach, A. J.: Morris, P. S.: Coates, H. L. C.: Nelson, S.: O'Leary, S. J.: Richmond, P. C.: Gunasekera, H.: Harkus, S.: Kong, K.: Brennon-Jones, C. G.: Brophy-Williams, S.: Currie, K.: Das, S. K.: Isaacs, D.: Jarosz, K.: Lehmann, D.: Pak, J.: Patel, H.: Perry, C.: Reath, J. S.: Sommer, J.: Torzillo, P. J. | 2021 | Otitis media guidelines for Australian Aboriginal and Torres Strait Islander children: summary of recommendations | A |
| 64 | Maruta, M.: Makizako, H.: Ikeda, Y.: Miyata, H.: Nakamura, A.: Han, G.: Shimokihara, S.: Tokuda, K.: Kubozono, T.: Ohishi, M.: Tabira, T. | 2021 | Association between apathy and satisfaction with meaningful activities in older adults with mild cognitive impairment: A population-based cross-sectional study | A |
| 65 | Matyas, L.: Dobbs, P.: Moittie, S. | 2021 | Acute fatal upper respiratory obstruction in a chimpanzee (Pan troglodytes) during anaesthesia Case report | A |
| 66 | Mills, K.: Paxton, B.: Walter, F. M.: Griffin, S. J.: Sutton, S.: Usher-Smith, J. A. | 2021 | Incorporating a brief intervention for personalised cancer risk assessment to promote behaviour change into primary care: a multi-methods pilot study | A |
| 67 | Min, K. D.: Kim, J. S.: Park, Y. H.: Shin, H. Y.: Kim, C.: Seo, S. W.: Kim, S. Y. | 2021 | New assessment for residential greenness and the association with cortical thickness in cognitively healthy adults | A |

| | | | | |
|---|---|---|---|---|
| 68 | Moral Peláez, I.: Brotons Cuixart, C.: Fernández Valverde, D.: Puig Palma, M.: Calvo Bonacho, E.: Martínez Muñoz, P.: Catalina Romero, C.: Quevedo Aguado, L. J. | 2021 | External validation of the European and American equations for calculating cardiovascular risk in a Spanish working population | A |
| 69 | Noguchi, T.: Nojima, I.: Inoue-Hirakawa, T.: Sugiura, H. | 2021 | Role of non–face-to-face social contacts in moderating the association between living alone and mental health among community-dwelling older adults: a cross-sectional study | A |
| 70 | Okoli, G. N.: Lam, O. L. T.: Abdulwahid, T.: Neilson, C. J.: Mahmud, S. M.: Abou-Setta, A. M. | 2021 | Seasonal influenza vaccination among cancer patients: A systematic review and meta-analysis of the determinants | A |
| 71 | Otaki, Y.: Watanabe, T.: Konta, T.: Watanabe, M.: Asahi, K.: Yamagata, K.: Fujimoto, S.: Tsuruya, K.: Narita, I.: Kasahara, M.: Shibagaki, Y.: Iseki, K.: Moriyama, T.: Kondo, M.: Watanabe, T. | 2021 | Impact of chronic kidney disease on aortic disease-related mortality: A four-year community-based cohort study | A |
| 72 | Sakurai, A.: Yamada, S. I.: Karasawa, I.: Kondo, E.: Kurita, H. | 2021 | Accuracy of a salivary examination kit for the screening of periodontal disease in a group medical check-up (Japanese-specific health check-up) | A |
| 73 | Shapira, U.: Brezinski, R. Y.: Rogowski, O.: Zeltser, D.: Berliner, S.: Shapira, I.: Shenhar-Tsarfaty, S.: Fireman, E. | 2021 | Association between elevated serum bilirubin levels with preserved lung function under conditions of exposure to air pollution | A |
| 74 | Shimizu, Y.: Arima, K.: Noguchi, Y.: Kawashiri, S. Y.: Yamanashi, H.: Tamai, M.: Nagata, Y.: Maeda, T. | 2021 | Possible mechanisms underlying the association between human T-cell leukemia virus type 1 (HTLV-1) and hypertension in elderly Japanese population | A |
| 75 | Somerset, S.: Evans, C.: Blake, H. | 2021 | Accessing voluntary hiv testing in the construction industry: A qualitative analysis of employee interviews from the test@work study | A |
| 76 | Takahashi, J.: Kawai, H.: Fujiwara, Y.: Watanabe, Y.: Hirano, H.: Kim, H.: Ihara, K.: Ejiri, M.: Ishii, K.: Oka, K.: Obuchi, S. | 2021 | Association between activity diversity and frailty among community-dwelling older Japanese: A cross-sectional study | A |
| 77 | Takami, A.: Watanabe, S.: Yamamoto, Y.: Miyachi, H.: Bamba, Y.: Ohata, M.: Mishima, S.: Kubota, H.: Nishiura, A.: Inaba, T.: Enomoto, M.: Mitsuhashi, T.: Nakanishi, K.: Miura, R.: Nonaka, E.: Shimbo, K.: Yatomi, Y.: Tohyama, K. | 2021 | Reference intervals of white blood cell parameters for healthy adults in japan | A |
| 78 | Takao, Toshihiro: Sumi, Naoki: Yamanaka, Yoshiyuki: Fujimoto, Sohachi: Kamada, Tomoari | 2021 | Associations between lifestyle behaviour changes and the optimal well-being of middle-aged Japanese individuals | A |
| 79 | Takizawa, M.: Kawachi, I.: Fujiwara, T.: Kizuki, M.: Nawa, N.: Kino, S. | 2021 | Association Between Maternal Working Status and Unintentional Injuries Among 3 to 4-Month-Old Infants in Japan | A |
| 80 | Takura, T.: Hirano Goto, K.: Honda, A. | 2021 | Development of a predictive model for integrated medical and long-term care resource consumption based on health | A |

| | | | | |
|---|---|---|---|---|
| | | | behaviour: application of healthcare big data of patients with circulatory diseases | |
| 81 | Tammemagi, M. | 2021 | ES12.02 Defining High Risk | A |
| 82 | Tan, Y. R.: Tan, E. H.: Jawahir, S.: Hanafiah, A. N. M.: Yunos, M. H. M. | 2021 | Demographic and socioeconomic inequalities in oral healthcare utilisation in Malaysia: evidence from a national survey | A |
| 83 | Tanaka, M.: Saito, M.: Takahashi, M.: Adachi, M.: Nakamura, K. | 2021 | Interformat Reliability of Web-Based Parent-Rated Questionnaires for Assessing Neurodevelopmental Disorders Among Preschoolers: Cross-sectional Community Study | A |
| 84 | Tsuboi, Y.: Yamada, H.: Munetsuna, E.: Fujii, R.: Yamazaki, M.: Ando, Y.: Mizuno, G.: Ishikawa, H.: Ohashi, K.: Hashimoto, S.: Hamajima, N.: Suzuki, K. | 2021 | Global DNA hypermethylation in peripheral blood mononuclear cells and cardiovascular disease risk: a population-based propensity score-matched cohort study | A |
| 85 | van der Steeg, G. E.: Takken, T. | 2021 | Reference values for maximum oxygen uptake relative to body mass in Duplicatetch/Flemish subjects aged 6-65 years: the LowLands Fitness Registry | A |
| 86 | Waade, J.: Seibt, U.: Honscha, W.: Rachidi, F.: Starke, A.: Speck, S.: Truyen, U. | 2021 | Multidrug-resistant enterobacteria in newborn dairy calves in Germany | A |
| 87 | Wakasugi, M.: Narita, I.: Iseki, K.: Asahi, K.: Yamagata, K.: Fujimoto, S.: Moriyama, T.: Konta, T.: Tsuruya, K.: Kasahara, M.: Shibagaki, Y.: Kondo, M.: Watanabe, T. | 2021 | The Effect of CKD on Associations between Lifestyle Factors and All-cause, Cancer, and Cardiovascular Mortality: A Population-based Cohort Study | A |
| 88 | Yahata, Y.: Fielding, J. E.: Kamiya, H.: Takimoto, N.: Ishii, J.: Fukusumi, M.: Sunagawa, T. | 2021 | Factors associated with knowledges and attitudes about measles and rubella immunization in a non-health care occupational setting in Japan | A |
| 89 | Yokokawa, H.: Fukuda, H.: Saita, M.: Goto, K.: Kaku, T.: Miyagami, T.: Takahashi, Y.: Hamada, C.: Hisaoka, T.: Naito, T. | 2021 | An association between visceral or subcutaneous fat accumulation and diabetes mellitus among Japanese subjects | A |
| 90 | Zulkiewicz, B. A.: Burrus, O.: Harshbarger, C.: Ortiz, A.: Garner, B. R.: Lewis, M. A. | 2021 | Identifying Implementation Strategies That Address Barriers and Facilitate Implementation of Digital Interventions in HIV Primary Care Settings: Results from the Pilot Implementation of Positive Health Check | A |
| 91 | Leach, A. J.: Morris, P. S.: Coates, H. L.: Nelson, S.: O'Leary, S. J.: Richmond, P. C.: Gunasekera, H.: Harkus, S.: Kong, K. L.: Brennon-Jones, C. G.: Brophy-Williams, S.: Currie, K.: Das, S. K.: Isaacs, D.: Jarosz, K.: Lehmann, D.: Pak, J.: Patel, H.: Perry, C.: Reath, J. S.: Sommer, J.: Torzillo, P. J. | 2021 | Otitis media guidelines for Australian Aboriginal and Torres Strait Islander children: summary of recommendations | A |

| | | | | |
|---|---|---|---|---|
| 92 | Zeng, Y.: He, H.: Wang, X.: Zhang, M.: An, Z. | 2021 | Climate and air pollution exposure are associated with thyroid function parameters: a retrospective cross-sectional study | A |
| 93 | Haya, M. A. N.: Ichikawa, S.: Shibagaki, Y.: Wakabayashi, H.: Takemura, Y. | 2020 | The "Healthy Akame!" community - government - university collaboration for health: a community-based participatory mixed-method approach to address health issue in rural Japan | A |
| 94 | Nomikos, N.: Naoum, P.: Naoum, V.: Athanasakis, K.: Kyriopoulos, J.: Pavi, E. | 2020 | PNS99 Personal Characteristics Associated with Subscription to Private Health Insurance in Greece − Results from the ″Health & Welfare″ Survey | A |
| 95 | Seilo, N.: Paldanius, S.: Autio, R.: Kunttu, K.: Kaila, M. | 2020 | Associations between e-health questionnaire responses, health checks and graduation: Finnish register-based study of 2011-2012 university entrants | A |
| 96 | Venkatesan, S.: Susila, S.: Suthanthiran, S.: Madhusudhan, S.: Paari, N. | 2020 | Identification and assessment of prediabetes - A rural Indian study (A correlative study between questionnaire and biochemical analysis) | A |
| 97 | Flynn, A. C.: Pryke, E.: Wadhera, M.: Poston, L.: White, S. L. | 2021 | A preconception intervention targeted at women with modifiable risk factors before pregnancy to improve outcomes; protocol for the Get Ready! feasibility trial | A |
| 98 | McShea, Lynzee: Giles, Karen: Murphy, Ashley: Ling, Jonathan | 2021 | An alternative approach for detecting hearing loss in adults with learning disabilities | A |
| 99 | Hakola, R.: Leino, T.: Luukkonen, R.: Kauppi, P. | 2020 | Occupational health check-ups and health-promoting programs and asthma | A |
| 100 | Ortiz, M. S.: Cabieses, B.: Oyarte, M.: Repetto, P. | 2020 | Disentangling socioeconomic inequalities of type 2 diabetes mellitus in Chile: A population-based analysis | A |
| 101 | Adeniyi, A.: Donnelly, L.: Janssen, P.: Jevitt, C.: von Bergman, H.: Brondani, M. | 2020 | A Qualitative Study of Health Care Providers' Views on Integrating Oral Health into Prenatal Care | B |
| 102 | Atuhaire, R.: Atuhaire, L. K.: Wamala, R.: Nansubuga, E. | 2020 | Interrelationships between early antenatal care, health facility delivery and early postnatal care among women in Uganda: a structural equation analysis | B |
| 103 | Geyti, C.: Christensen, K. S.: Dalsgaard, E. M.: Bech, B. H.: Gunn, J.: Maindal, H. T.: Sandbaek, A. | 2020 | Factors associated with non-initiation of mental healthcare after detection of poor mental health at a scheduled health check: A cohort study | B |
| 104 | Habukawa, C.: Nagamitsu, S.: Koyanagi, K.: Nishikii, Y.: Yanagimoto, Y.: Seiji, Y.: Suzuki, Y.: Go, S.: Murakami, K. | 2020 | Late bedtime reflects QTA30 anxiety symptoms in adolescents in a school checkup | B |
| 105 | Weniger, M.: Beesdo-Baum, K.: Roessner, V.: Hense, H.: Knappe, S. | 2021 | Successful prevention of mental health problems? From regular health check-ups to indicative preventive measures | B |

| | | | | |
|---|---|---|---|---|
| | | | for emotional and behavioral problems in preschool and primary school age: a prospective implementation study | |
| 106 | Atkins, L.: Stefanidou, C.: Chadborn, T.: Thompson, K.: Michie, S.: Lorencatto, F. | 2020 | Influences on NHS Health Check behaviours: a systematic review | C |
| 107 | Cheong, A. T.: Tong, S. F.: Chinna, K.: Khoo, E. M.: Liew, S. M. | 2020 | Gender differences in factors influencing intention to undergo cardiovascular disease health checks: A cross-sectional survey | C |
| 108 | Gaede, L.: Sitges, M.: Neil, J.: Selvi, E.: Woan, W.: Derks, R.: Mollmann, H. | 2020 | European heart health survey 2019 | C |
| 109 | Garriga, C.: Robson, J.: Coupland, C.: Hippisley-Cox, J. | 2020 | NHS Health Checks for people with mental ill-health 2013-2017: An observational study | C |
| 110 | Patel, R.: Barnard, S.: Thompson, K.: Lagord, C.: Clegg, E.: Worrall, R.: Evans, T.: Carter, S.: Flowers, J.: Roberts, D.: Nuttall, M.: Samani, N. J.: Robson, J.: Kearney, M.: Deanfield, J.: Waterall, J. | 2020 | Evaluation of the uptake and delivery of the NHS Health Check programme in England, using primary care data from 9.5 million people: A cross-sectional study | C |
| 111 | Duplicateddy, C.: Wong, G.: Gadsby, E. W.: Krska, J.: Hibberd, V. | 2021 | NHS Health Check programme: A protocol for a realist review | C |
| 112 | Grauman, Å: Hansson, M.: James, S.: Hauber, B.: Veldwijk, J. | 2021 | Communicating Test Results from a General Health Check: Preferences from a Discrete Choice Experiment Survey | C |
| 113 | Lee, H. Y.: Kim, S.: Neese, J.: Lee, M. H. | 2021 | Does health literacy affect the uptake of annual physical check-ups?: Results from the 2017 US health information national trends survey | C |
| 114 | Grauman, A.: Hansson, M.: James, S.: Hauber, B.: Veldwijk, J. | 2021 | Communicating Test Results from a General Health Check: Preferences from a Discrete Choice Experiment Survey | C |
| 115 | Bartlett, E. C.: Kemp, S. V.: Ridge, C. A.: Desai, S. R.: Mirsadraee, S.: Morjaria, J. B.: Shah, P. L.: Popat, S.: Nicholson, A. G.: Rice, A. J.: Jordan, S.: Begum, S.: Mani, A.: Derbyshire, J.: Morris, K.: Chen, M.: Peacock, C.: Addis, J.: Martins, M.: Kaye, S. B.: Padley, S. P. G.: Devaraj, A.: McDonald, F.: Robertus, J. L.: Lim, E.: Barnett, J.: Finch, J.: Dalal, P.: Yousaf, N.: Jamali, A.: Ivashniova, N.: Phillips, C.: Newsom-Davies, T.: Lee, R.: Vaghani, P.: Whiteside, S.: Vaughan-Smith, S. | 2020 | Baseline Results of the West London lung cancer screening pilot study - Impact of mobile scanners and dual risk model utilisation | Duplicate |
| 116 | Crosbie, P. A.: Gabe, R.: Simmonds, I.: Kennedy, M.: Rogerson, S.: Ahmed, N.: Baldwin, D. R.: Booton, R.: Cochrane, A.: Darby, M.: Franks, K.: Hinde, S.: Janes, S. M.: Macleod, U.: Messenger, | 2020 | Yorkshire Lung Screening Trial (YLST): protocol for a randomised controlled trial to evaluate invitation to community-based low-dose CT screening for lung cancer versus usual care in a targeted population at risk | Duplicate |

| | | | | |
|---|---|---|---|---|
| | M.: Moller, H.: Murray, R. L.: Neal, R. D.: Quaife, S. L.: Sculpher, M.: Tharmanathan, P.: Torgerson, D.: Callister, M. E. | | | |
| 117 | Gaede, L.: Sitges, M.: Neil, J.: Selvi, E.: Woan, W.: Derks, R.: Möllmann, H. | 2020 | European heart health survey 2019 | Duplicate |
| 118 | Kuronen, J.: Winell, K.: Kopra, J.: Räsänen, K. | 2020 | Quality improvement activity in occupational healthcare associated with reduced need for disability retirement: A bayesian mixed effects modelling study in finland | Duplicate |
| 119 | Lewis, M. A.: Harshbarger, C.: Bann, C.: Burrus, O.: Peinado, S.: Garner, B. R.: Khavjou, O.: Shrestha, R. K.: Karns, S.: Borkowf, C. B.: Zulkiewicz, B. A.: Ortiz, A.: Galindo, C. A.: DallaPiazza, M.: Holm, P.: Marconi, V. C.: Somboonwit, C.: Swaminathan, S.: Positive Hlth Check Study, Team | 2020 | Positive Health Check evaluation: A type 1 hybrid design randomized trial to decrease HIV viral loads in patients seen in HIV primary care | Duplicate |
| 120 | Liersch, S.: Krüger, K.: Oedingen, C.: Spreenberg, A.: Bergemann, T.: Krauth, C. | 2020 | Evaluation of the pediatric-centered integrated care AOK Junior: protocol for a mixed-method study | Duplicate |
| 121 | Liu, K.: Xu, J.: Tao, L.: Yang, K.: Sun, Y.: Guo, X. | 2020 | Platelet counts are associated with arterial stiffness in Chinese Han population: A longitudinal study | Duplicate |
| 122 | Pahk, K.: Kwon, Y.: Park, S.: Kim, S. | 2020 | Visceral fat metabolic activity evaluated by 18F-FDG PET/CT predicts osteoporosis in healthy postmenopausal Korean women | Duplicate |
| 123 | Palladino, R.: Vamos, E. P.: Chang, K. C.: Khunti, K.: Majeed, A.: Millett, C. | 2020 | Evaluation of the Diabetes Screening Component of a National Cardiovascular Risk Assessment Programme in England: a Retrospective Cohort Study | Duplicate |
| 124 | Park, Sang-Mi: Hong, Seok-Pyo | 2020 | The association of health care access and utilization with self-perceived health in South Korea: The significance of age | Duplicate |
| 125 | Rein, D.: Claus, M.: Frosch, W.: März, W.: Lorkowski, S.: Webendoerfer, S.: Schreiner, T. | 2020 | Changes in erythrocyte omega-3 fatty acids in german employees upon dietary advice by corporate health | Duplicate |
| 126 | Borrull-Guardeño, J.: Sebastiá-Laguarda, C.: Donat-Colomer, F.: Sánchez-Martínez, V. | 2021 | Women's knowledge and attitudes towards cervical cancer prevention: A qualitative study in the Spanish context | Duplicate |
| 127 | Borrull-Guardeño, Jessica: Sebastiá-Laguarda, Cruz: Donat-Colomer, Francisco: Sánchez-Martínez, Vanessa | 2021 | Women’s knowledge and attitudes towards cervical cancer prevention: A qualitative study in the spanish context | Duplicate |
| 128 | Herpertz-Dahlmann, Beate: Bonin, Eva: Dahmen, Brigitte | 2021 | Can you find the right support for children, adolescents and young adults with anorexia nervosa: Access to age-appropriate care systems in various healthcare systems | Duplicate |
| 129 | Maruta, Michio: Makizako, Hyuma: Ikeda, Yuriko: Miyata, Hironori: Nakamura, Atsushi: Han, Gwanghee: Shimokihara, Suguru: Tokuda, Keiichiro: Kubozono, Takuro: Ohishi, Mitsuru: Tabira, Takayuki | 2021 | Association between apathy and satisfaction with meaningful activities in older adults with mild cognitive impairment: A population-based cross-sectional study | Duplicate |

| | | | | |
|---|---|---|---|---|
| 130 | Tan, Y. R.: Tan, E. H.: Jawahir, S.: Mohd Hanafiah, A. N.: Mohd Yunos, M. H. | 2021 | Demographic and socioeconomic inequalities in oral healthcare utilisation in Malaysia: evidence from a national survey | Duplicate |
| 131 | Abu Awwad, D.: Hossain, S. Z.: Mackey, M.: Brennan, P.: Adam, S. | 2021 | Exploring the role of healthcare organisations in increasing women's participation in breast-screening in the United Arab Emirates | Duplicate |
| 132 | Adeniyi, A.: Donnelly, L.: Janssen, P.: Jevitt, C.: von Bergman, H.: Brondani, M. | 2021 | A Qualitative Study of Health Care Providers' Views on Integrating Oral Health into Prenatal Care | Duplicate |
| 133 | Herpertz-Dahlmann, B.: Bonin, E.: Dahmen, B. | 2021 | Can you find the right support for children, adolescents and young adults with anorexia nervosa: Access to age-appropriate care systems in various healthcare systems | Duplicate |
| 134 | Hulsegge, G.: Proper, K. I.: Loef, B.: Paagman, H.: Anema, J. R.: van Mechelen, W. | 2021 | The mediating role of lifestyle in the relationship between shift work, obesity and diabetes | Duplicate |
| 135 | Maruta, M.: Makizako, H.: Ikeda, Y.: Miyata, H.: Nakamura, A.: Han, G.: Shimokihara, S.: Tokuda, K.: Kubozono, T.: Ohishi, M.: Tabira, T. | 2021 | Association between apathy and satisfaction with meaningful activities in older adults with mild cognitive impairment: A population-based cross-sectional study | Duplicate |
| 136 | Alaei-Shahmiri, F.: Khamseh, M. E.: Manhoei, K.: Yadegari, H.: Kazemi, H.: Meshkini, M. | 2020 | The optimal vitamin D cut-off value associated with hyperglycemia in an Iranian population | Duplicate from August 2020 literature research |
| 137 | Alageel, S.: Gulliford, M. C.: Wright, A.: Khoshaba, B.: Burgess, C. | 2020 | Engagement with advice to reduce cardiovascular risk following a health check programme: A qualitative study | Duplicate from August 2020 literature research |
| 138 | An, T. D.: McDermott, R.: Knight, J.: Hua, X. Y.: Barr, E. L. M.: Arabena, K.: Palmer, A.: Clarke, P. M. | 2020 | Development and Use of Prediction Models for Classification of Cardiovascular Risk of Remote Indigenous Australians | Duplicate from August 2020 literature research |
| 139 | Balata, H.: Harvey, J.: Barber, P. V.: Colligan, D.: Duplicateerden, R.: Elton, P.: Evison, M.: Greaves, M.: Howells, J.: Irion, K.: Karunaratne, D.: Mellor, S.: Newton, T.: Sawyer, R.: Sharman, A.: Smith, E.: Taylor, B.: Taylor, S.: Tonge, J.: Walsham, A.: Whittaker, J.: Vestbo, J.: Booton, R.: Crosbie, P. A. | 2020 | Spirometry performed as part of the Manchester community-based lung cancer screening programme detects a high prevalence of airflow obstruction in individuals without a prior diagnosis of COPD | Duplicate from August 2020 literature research |
| 140 | Balata, H.: Traverse-Healy, L.: Blandin-Knight, S.: Armitage, C.: Barber, P.: Colligan, D.: Elton, P.: Kirwan, M.: Lyons, J.: McWilliams, L.: Novasio, J.: Sharman, A.: Slevin, K.: Taylor, S.: Tonge, J.: Waplington, S.: Yorke, J.: Evison, M.: Booton, R.: Crosbie, P. A. J. | 2020 | Attending community-based lung cancer screening influences smoking behaviour in deprived populations | Duplicate from August 2020 literature research |
| 141 | Bhasin, V.: Mehta, A.: Skopicki, H. A.: Parikh, P. B. | 2020 | Predictors of Aspirin Nonadherence in Adults with Prior Myocardial Infarction | Duplicate from August 2020 literature research |

| | | | | |
|---|---|---|---|---|
| 142 | Billstedt, E.: Nilsson, G.: Leffler, L.: Carlsson, L.: Olsson, I.: Fernell, E.: Gillberg, C. | 2020 | Cognitive functioning in a representative cohort of preschool children with febrile seizures | Duplicate from August 2020 literature research |
| 143 | Blake, H.: Somerset, S.: Evans, C. | 2020 | Development and fidelity testing of the test@work digital toolkit for employers on workplace health checks and opt-in HIV testing | Duplicate from August 2020 literature research |
| 144 | Boongird, P.: Chamnan, P.: Laptikultham, S.: Krittayapoositpot, P.: Nitiyanant, W.: Aekplakorn, W.: Mangklabruks, A. | 2020 | Dose–response relationship between physical exercise and risk of physician-diagnosed dementia in 206 073 Thai community-dwelling men and women: HCUR study | Duplicate from August 2020 literature research |
| 145 | Boongird, P.: Chamnan, P.: Laptikultham, S.: Krittayapoositpot, P.: Nitiyanant, W.: Aekplakorn, W.: Mangklabruks, A. | 2020 | Dose–response relationship between physical exercise and risk of physician-diagnosed dementia in 206 073 Thai community-dwelling men and women: HCUR study | Duplicate from August 2020 literature research |
| 146 | Boongird, P.: Chamnan, P.: Laptikultham, S.: Krittayapoositpot, P.: Nitiyanant, W.: Aekplakorn, W.: Mangklabruks, A.: Hcur Investigators | 2020 | Dose-response relationship between physical exercise and risk of physician-diagnosed dementia in 206 073 Thai community-dwelling men and women: HCUR study | Duplicate from August 2020 literature research |
| 147 | Bosman, L. C.: Twisk, J. W. R.: Geraedts, A. S.: Heymans, M. W. | 2020 | Effect of Partial Sick Leave on Sick Leave Duplicateration in Employees with Musculoskeletal Disorders | Duplicate from August 2020 literature research |
| 148 | Bunten, A.: Porter, L.: Gold, N.: Bogle, V. | 2020 | A systematic review of factors influencing NHS health check uptake: invitation methods, patient characteristics, and the impact of interventions | Duplicate from August 2020 literature research |
| 149 | Carlos-Oliva, D.: Paula Vitale, M.: Grañana, N.: Eugenia Rouvier, M.: Zeltman, C. | 2020 | Neurodevelopmental development with the use of the Ages and Stages Questionnaire (ASQ-3) in monitoring children’s health | Duplicate from August 2020 literature research |
| 150 | Carlos-Oliva, D.: Vitale, M. P.: Granana, N.: Rouvier, M. E.: Zeltman, C. | 2020 | Neurodevelopmental development with the use of the Ages and Stages Questionnaire (ASQ-3) in monitoring children's health | Duplicate from August 2020 literature research |
| 151 | Carlos-Oliva, D.: Vitale, M. P.: Grañana, N.: Rouvier, M. E.: Zeltman, C. | 2020 | [Neurodevelopmental development with the use of the Ages and Stages Questionnaire (ASQ-3) in monitoring children's health] | Duplicate from August 2020 literature research |
| 152 | Chattopadhyay, K.: Biswas, M.: Moore, R. | 2020 | NHS Health Check and healthy lifestyle in Leicester, England: analysis of a survey dataset | Duplicate from August 2020 literature research |
| 153 | Chinn, D. | 2020 | An empirical examination of the use of Easy Read health information in health consultations involving patients with intellectual disabilities | Duplicate from August 2020 literature research |

| | | | | |
|---|---|---|---|---|
| 154 | Dickson, J.: Quaife, S.: Horst, C.: Hall, H.: Tisi, S.: Mullin, A.: Farrelly, L.: Gyertson, K.: Nnorom, S.: Levermore, C.: Bojang, F.: Anastasiadis, T.: Sennett, K.: Clarke, C.: Allen, B.: Hamilton, S.: Hartmann, A.: Hackshaw, A.: Janes, S. | 2020 | The SUMMIT study: invitation strategy and screening uptake of the first 36,680 invited | Duplicate from August 2020 literature research |
| 155 | Flitcroft, L.: Chen, W. S.: Meyer, D. | 2020 | The demographic representativeness and health outcomes of digital health station users: Longitudinal study | Duplicate from August 2020 literature research |
| 156 | Fujii, R.: Yamada, H.: Munetsuna, E.: Yamazaki, M.: Ohashi, K.: Ishikawa, H.: Maeda, K.: Hagiwara, C.: Ando, Y.: Hashimoto, S.: Hamajima, N.: Suzuki, K. | 2020 | Associations of Circulating MicroRNAs (miR-17, miR-21, and miR-150) and Chronic Kidney Disease in a Japanese Population | Duplicate from August 2020 literature research |
| 157 | Fukushima, Keiko: Fukushima, Noritoshi: Sato, Hiroki: Yokota, Jinko: Uchida, Keiko | 2020 | Association between nutritional level, menstrual-related symptoms, and mental health in female medical students | Duplicate from August 2020 literature research |
| 158 | Hammarberg, K.: Hassard, J.: de Silva, R.: Johnson, L. | 2020 | Acceptability of screening for pregnancy intention in general practice: a population survey of people of reproductive age | Duplicate from August 2020 literature research |
| 159 | Hansen, C. B.: Pavlovic, K. M. H.: Sondergaard, J.: Thilsing, T. | 2020 | Does GP empathy influence patient enablement and success in lifestyle change among high risk patients? | Duplicate from August 2020 literature research |
| 160 | Hao, L.: Wang, Z. Z.: Wang, Y.: Wang, J.: Zeng, Z. P. | 2020 | Association between Cardiorespiratory Fitness, Relative Grip Strength with Non-Alcoholic Fatty Liver Disease | Duplicate from August 2020 literature research |
| 161 | Hao, L.: Wang, Z.: Wang, Y.: Wang, J.: Zeng, Z. | 2020 | Association between cardiorespiratory fitness, relative grip strength with non-alcoholic fatty liver disease | Duplicate from August 2020 literature research |
| 162 | Hardeman, W.: Mitchell, J.: Pears, S.: Van Emmenis, M.: Theil, F.: Gc, V. S.: Vasconcelos, J. C.: Westgate, K.: Brage, S.: Suhrcke, M.: Griffin, S. J.: Kinmonth, A. L.: Wilson, E. C. F.: Prevost, A. T.: Sutton, S. | 2020 | Evaluation of a very brief pedometer-based physical activity intervention delivered in NHS Health Checks in England: The VBI randomised controlled trial | Duplicate from August 2020 literature research |
| 163 | Hardeman, W.: Mitchell, J.: Pears, S.: Van Emmenis, M.: Theil, F.: Gc, V. S.: Vasconcelos, J. C.: Westgate, K.: Brage, S.: Suhrcke, M.: Griffin, S. J.: Kinmonth, A. L.: Wilson, E. C. F.: Prevost, A. T.: Sutton, S.: V. B. I. Res Team | 2020 | Evaluation of a very brief pedometer-based physical activity intervention delivered in NHS Health Checks in England: The VBI randomised controlled trial | Duplicate from August 2020 literature research |
| 164 | Hasegawa, M.: Akter, S.: Hu, H. H.: Kashino, I.: Kuwahara, K.: Okazaki, H.: Sasaki, N.: Ogasawara, T.: Eguchi, M.: Kochi, T.: Miyamoto, T.: Nakagawa, T.: Honda, T.: Yamamoto, S.: Murakami, T.: Shimizu, M.: Uehara, A.: Yamamoto, M.: Imai, T.: Nishihara, A.: Tomita, K.: Nagahama, S.: Hori, A.: Konishi, M.: | 2020 | Five-year cumulative incidence of overweight and obesity, and longitudinal change in body mass index in Japanese workers: The Japan Epidemiology Collaboration on Occupational Health Study | Duplicate from August 2020 literature research |

| | | | | |
|---|---|---|---|---|
| | Kabe, I.: Mizoue, T.: Kunugita, N.: Dohi, S.: Mizoue, T.: Akter, S.: Hu, H.: Inoue, Y.: Fukunaga, A.: Kashino, I.: Islam, Z.: Konishi, M.: Nanri, A.: Kurotani, K.: Kuwahara, K.: Watanabe, Y.: Imai, T.: Miyamoto, T.: Hasegawa, M.: Shirozu, M.: Kabe, I.: Shimizu, C.: Gonmori, N.: Ogasawara, A.: Kato, N.: Tomizawa, A.: Kunugita, N.: Sone, T.: Fukasawa, K.: Hori, A.: Nishiura, C.: Kinugawa, C.: Kuroda, R.: Yamamoto, K.: Ohtsu, M.: Sakamoto, N.: Osaki, Y.: Totsuzaki, T.: Endo, M.: Itoh, T.: Kawashima, M.: Masuda, M.: Kitahara, K.: Yokoya, T.: Fukai, K.: Odagami, K.: Kobayashi, Y.: Japan Epidemiology Collaboration, O. | | | |
| 165 | Hasegawa, T.: Asakura, M.: Asanuma, H.: Amaki, M.: Takahama, H.: Sugano, Y.: Kanzaki, H.: Yasuda, S.: Anzai, T.: Izumi, C.: Kitakaze, M. | 2020 | Difference in the prevalence of subclinical left ventricular impairment among left ventricular geometric pattern in a community-based population | Duplicate from August 2020 literature research |
| 166 | Henderson, L. J.: Smolders, T. V.: Roughan, J. V. | 2020 | Identifying obstacles preventing the uptake of tunnel handling methods for laboratory mice: An international thematic survey | Duplicate from August 2020 literature research |
| 167 | Henderson, L. J.: Smulders, T. V.: Roughan, J. V. | 2020 | Identifying obstacles preventing the uptake of tunnel handling methods for laboratory mice: An international thematic survey | Duplicate from August 2020 literature research |
| 168 | Hu, B.: Shi, X.: Duplicate, X.: Xu, M.: Wang, Q.: Zhao, H. | 2020 | Pattern of immune infiltration in lung cancer and its clinical implication | Duplicate from August 2020 literature research |
| 169 | Hulsegge, G.: van Mechelen, W.: Paagman, H.: Proper, K. I.: Anema, J. R. | 2020 | The moderating role of lifestyle, age, and years working in shifts in the relationship between shift work and being overweight | Duplicate from August 2020 literature research |
| 170 | Hulsegge, G.: van Mechelen, W.: Proper, K. I.: Paagman, H.: Anema, J. R. | 2020 | Shift work, and burnout and distress among 7798 blue-collar workers | Duplicate from August 2020 literature research |
| 171 | Hyseni, L.: Guzman-Castillo, M.: Kypridemos, C.: Collins, B.: Schwaller, E.: Capewell, S.: Boland, A.: Dickson, R.: O'Flaherty, M.: Gallacher, K.: Hale, P.: Lloyd-Williams, F. | 2020 | Engaging with stakeholders to inform the development of a decision-support tool for the NHS health check programme: qualitative study | Duplicate from August 2020 literature research |
| 172 | Ikemiyagi, H.: Ishida, A.: Kinjo, K.: Ohya, Y. | 2020 | A high normal ankle--brachial index is associated with electrocardiography-determined left ventricular hypertrophy: the Okinawa Peripheral Arterial Disease Study (OPADS) | Duplicate from August 2020 literature research |
| 173 | Iseki, K.: Konta, T.: Asahi, K.: Yamagata, K.: Fujimoto, S.: Tsuruya, K.: Narita, I.: Kasahara, M.: Shibagaki, Y.: Moriyama, T.: Kondo, M.: Iseki, C.: Watanabe, T. | 2020 | Impact of metabolic syndrome on the mortality rate among participants in a specific health check and guidance program in japan | Duplicate from August 2020 literature research |

| | | | | |
|---|---|---|---|---|
| 174 | Jeon, S. W.: Chang, Y.: Lim, S. W.: Cho, J.: Kim, H. N.: Kim, K. B.: Kim, J.: Kim, Y. H.: Shin, D. W.: Oh, K. S.: Shin, Y. C.: Ryu, S. | 2020 | Bidirectional association between blood pressure and depressive symptoms in young and middle-age adults: A cohort study | Duplicate from August 2020 literature research |
| 175 | Kabasawa, K.: Tanaka, J.: Nakamura, K.: Ito, Y.: Yoshida, K.: Takachi, R.: Sawada, N.: Tsugane, S.: Narita, I. | 2020 | Study Design and Baseline Profiles of Participants in the Uonuma CKD Cohort Study in Niigata, Japan | Duplicate from August 2020 literature research |
| 176 | Kameda, T.: Kumamaru, H.: Nishimura, S.: Kohsaka, S.: Miyata, H. | 2020 | Use of oral antidiabetic drugs in Japanese working-age patients with type 2 diabetes mellitus: dosing pattern for metformin initiators | Duplicate from August 2020 literature research |
| 177 | Kim, Y. G.: Han, K. D.: Choi, J. I.: Boo, K. Y.: Kim, D. Y.: Lee, K. N.: Shim, J.: Kim, J. S.: Kim, Y. H. | 2020 | Frequent drinking is a more important risk factor for new-onset atrial fibrillation than binge drinking: A nationwide population-based study | Duplicate from August 2020 literature research |
| 178 | Kobayashi, K.: Imagama, S.: Ando, K.: Tsushima, M.: Machino, M.: Ota, K.: Tanaka, S.: Morozumi, M.: Kanbara, S.: Ishiguro, N.: Hasegawa, Y. | 2020 | Weakness of grip strength reflects future locomotive syndrome and progression of locomotive risk stage: A 10-year longitudinal cohort study | Duplicate from August 2020 literature research |
| 179 | Kummer, S.: Waller, J.: Ruparel, M.: Cass, J.: Janes, S. M.: Quaife, S. L. | 2020 | Mapping the spectrum of psychological and behavioural responses to low-dose CT lung cancer screening offered within a Lung Health Check | Duplicate from August 2020 literature research |
| 180 | Kummer, Sonja: Waller, Jo: Ruparel, Mamta: Cass, Judith: Janes, Samuel M.: Quaife, Samantha L. | 2020 | Mapping the spectrum of psychological and behavioural responses to low-dose CT lung cancer screening offered within a lung health check | Duplicate from August 2020 literature research |
| 181 | Lam, M. T.: Li, H. W. R.: Wong, C. Y. G.: Yeung, W. S. B.: Ho, P. C.: Ng, E. H. Y. | 2020 | Women's age and total motile normal morphology sperm count predict fecundability: a prospective cohort study | Duplicate from August 2020 literature research |
| 182 | Landecho, M. F.: Alegría-Murillo, L.: López-Fidalgo, J.: Colina, I.: Santesteban, V.: García-Unciti, M.: Beloqui, O.: Frühbeck, G.: Cuervo, M. | 2020 | Unravelling gender-specific factors that link obesity to albuminuria | Duplicate from August 2020 literature research |
| 183 | Larsen, L. B.: Thilsing, T.: Pedersen, L. B. | 2020 | Patient preferences for preventive health checks in Danish general practice: a discrete choice experiment among patients at high risk of noncommunicable diseases | Duplicate from August 2020 literature research |
| 184 | Latz, Isabel K. | 2020 | Associations between perceptions of U.S. immigration enforcement policies, physical health, psychological distress, and health care utilization in a Hispanic border community | Duplicate from August 2020 literature research |
| 185 | Lebrett, M. B.: Balata, H.: Evison, M.: Colligan, D.: Duplicateerden, R.: Elton, P.: Greaves, M.: Howells, J.: Irion, K.: Karunaratne, D.: Lyons, J.: Mellor, S.: Myerscough, A.: Newton, T.: Sharman, A.: Smith, E.: Taylor, B.: Taylor, S.: Walsham, A.: | 2020 | Analysis of lung cancer risk model (PLCO M2012 and LLP v2) performance in a community-based lung cancer screening programme | Duplicate from August 2020 literature research |

| | | | | |
|---|---|---|---|---|
| | Whittaker, J.: Barber, P. V.: Tonge, J.: Robbins, H. A.: Booton, R.: Crosbie, P. A. J. | | | |
| 186 | Lewis, M. A.: Harshbarger, C.: Bann, C.: Burrus, O.: Peinado, S.: Garner, B. R.: Khavjou, O.: Shrestha, R. K.: Karns, S.: Borkowf, C. B.: Zulkiewicz, B. A.: Ortiz, A.: Galindo, C. A.: DallaPiazza, M.: Holm, P.: Marconi, V. C.: Somboonwit, C.: Swaminathan, S. | 2020 | Positive Health Check evaluation: A type 1 hybrid design randomized trial to decrease HIV viral loads in patients seen in HIV primary care | Duplicate from August 2020 literature research |
| 187 | Liu, K.: Xu, J. F.: Tao, L. X.: Yang, K.: Sun, Y.: Guo, X. H. | 2020 | Platelet counts are associated with arterial stiffness in Chinese Han population: a longitudinal study | Duplicate from August 2020 literature research |
| 188 | Lloyd-Williams, F.: Hyseni, L.: Guzman-Castillo, M.: Kypridemos, C.: Collins, B.: Capewell, S.: Schwaller, E.: O'Flaherty, M. | 2020 | Evaluating stakeholder involvement in building a decision support tool for NHS health checks: co-producing the WorkHORSE study | Duplicate from August 2020 literature research |
| 189 | Mc Namara, K. P.: Krass, I.: Peterson, G. M.: Alzubaidi, H.: Grenfell, R.: Freedman, B.: Duplicatenbar, J. A. | 2020 | Implementing screening interventions in community pharmacy to promote interprofessional coordination of primary care - A mixed methods evaluation | Duplicate from August 2020 literature research |
| 190 | Mengoni, S. E.: Redman, S. | 2020 | Health monitoring of young children with Down syndrome: A parent-report study | Duplicate from August 2020 literature research |
| 191 | Nagamatsu, Y.: Barroga, E.: Sakyo, Y.: Igarashi, Y.: Hirano O, Y. | 2020 | Risks and perception of non-communicable diseases and health promotion behavior of middle-aged female immigrants in Japan: A qualitative exploratory study | Duplicate from August 2020 literature research |
| 192 | Nagamatsu, Y.: Barroga, E.: Sakyo, Y.: Igarashi, Y.: Hirano, Y. O. | 2020 | Risks and perception of non-communicable diseases and health promotion behavior of middle-aged female immigrants in Japan: a qualitative exploratory study | Duplicate from August 2020 literature research |
| 193 | Nahar, P.: van Marwijk, H.: Gibson, L.: Musinguzi, G.: Anthierens, S.: Ford, E.: Bremner, S. A.: Bowyer, M.: Le Reste, J. Y.: Sodi, T.: Bastiaens, H. | 2020 | A protocol paper: community engagement interventions for cardiovascular disease prevention in socially disadvantaged populations in the UK: an implementation research study | Duplicate from August 2020 literature research |
| 194 | Oakey-Neate, L.: Schrader, G.: Strobel, J.: Bastiampillai, T.: van Kasteren, Y.: Bidargaddi, N. | 2020 | Using algorithms to initiate needs-based interventions for people on antipsychotic medication: implementation protocol | Duplicate from August 2020 literature research |
| 195 | Pahk, K.: Kwon, Y.: Kim, M. K.: Park, S.: Kim, S. | 2020 | Visceral fat metabolic activity evaluated by 18F-FDG PET/CT is associated with osteoporosis in healthy postmenopausal Korean women | Duplicate from August 2020 literature research |
| 196 | Pahk, K.: Kwon, Y.: Kim, M. K.: Park, S.: Kim, S. | 2020 | Visceral fat metabolic activity evaluated by F-18-FDG PET/CT is associated with osteoporosis in healthy postmenopausal Korean women | Duplicate from August 2020 literature research |

| | | | | |
|---|---|---|---|---|
| 197 | Paldanius, S.: Seilo, N.: Kunttu, K.: Autio, R.: Kaila, M. | 2020 | Screening University Students for Health Checks With an Electronic Health Questionnaire in Finland: Protocol for a Retrospective, Register-Based Cohort Study | Duplicate from August 2020 literature research |
| 198 | Park, S. M.: Hong, S. P. | 2020 | The association of health care access and utilization with self-perceived health in South Korea: the significance of age | Duplicate from August 2020 literature research |
| 199 | Petersen, J.: Kontsevaya, A.: McKee, M.: Richardson, E.: Cook, S.: Malyutina, S.: Kudryavtsev, A. V.: Leon, D. A. | 2020 | Primary care use and cardiovascular disease risk in Russian 40-69 year olds: a cross-sectional study | Duplicate from August 2020 literature research |
| 200 | Petersen, J.: Malyutina, S.: Ryabikov, A.: Kontsevaya, A.: Kudryavtsev, A. V.: Eggen, A. E.: McKee, M.: Cook, S.: Hopstock, L. A.: Schirmer, H.: Leon, D. A. | 2020 | Uncontrolled and apparent treatment resistant hypertension: A cross-sectional study of Russian and Norwegian 40-69 year olds | Duplicate from August 2020 literature research |
| 201 | Quaife, S. L.: Ruparel, M.: Dickson, J. L.: Beeken, R. J.: McEwen, A.: Baldwin, D. R.: Bhowmik, A.: Navani, N.: Sennett, K.: Duplicateffy, S. W.: Wardle, J.: Waller, J.: Janes, S. M. | 2020 | Lung screen uptake trial (LSUT): Randomized controlled clinical trial testing targeted invitation materials | Duplicate from August 2020 literature research |
| 202 | Ramagiri, R.: Kannuri, N. K.: Lewis, M. G.: Murthy, G. V. S.: Gilbert, C. | 2020 | Evaluation of whether health education using video technology increases the uptake of screening for diabetic retinopathy among individuals with diabetes in a slum population in Hyderabad | Duplicate from August 2020 literature research |
| 203 | Rauf, M.: Houlbrooke, A.: Birks, P.: Barnett, J.: Alison, B.: Owen, G.: Leyakathali Khan, S.: Hussain, I. | 2020 | Lung health check: should there be a general health check? | Duplicate from August 2020 literature research |
| 204 | Robertson, G.: Fleming, A.: Williams, M. C.: Trucco, E.: Quinn, N.: Hogg, R.: McKay, G. J.: Kee, F.: Young, I.: Pellegrini, E.: Newby, D. E.: Van Beek, E. J. R.: Peto, T.: Dhillon, B.: Van Hemert, J.: MacGillivray, T. J. | 2020 | Association between hypertension and retinal vascular features in ultra-widefield fundus imaging | Duplicate from August 2020 literature research |
| 205 | Robertson, G.: Fleming, A.: Williams, M. C.: Trucco, E.: Quinn, N.: Hogg, R.: McKay, G. J.: Kee, F.: Young, I.: Pellegrini, E.: Newby, D. E.: van Beek, E. J. R.: Peto, T.: Dhillon, B.: van Hemert, J.: MacGillivray, T. J.: Northern Ireland Cohort, Longitu | 2020 | Association between hypertension and retinal vascular features in ultra-widefield fundus imaging | Duplicate from August 2020 literature research |
| 206 | Ruparel, M.: Quaife, S. L.: Dickson, J. L.: Horst, C.: Tisi, S.: Hall, H.: Taylor, M. N.: Ahmed, A.: Shaw, P. J.: Burke, S.: Soo, M. J.: Nair, A.: Devaraj, A.: Sennett, K.: Hurst, J. R.: Duplicateffy, S. W.: Navani, N.: Bhowmik, A.: Baldwin, D. R.: Janes, S. M. | 2020 | Prevalence, symptom burden, and underdiagnosis of chronic obstructive pulmonary disease in a lung cancer screening cohort | Duplicate from August 2020 literature research |
| 207 | Ruparel, M.: Quaife, S. L.: Dickson, J. L.: Horst, C.: Tisi, S.: Hall, H.: Taylor, M.: Ahmed, A.: Shaw, P.: Burke, S.: Soo, M. J.: Nair, A.: Devaraj, A.: Sennett, K.: Duplicateffy, S. W.: Navani, N.: Bhowmik, A.: Baldwin, D. R.: Janes, S. M. | 2020 | Lung Screen Uptake Trial: Results from a single lung cancer screening round | Duplicate from August 2020 literature research |

| | | | | |
|---|---|---|---|---|
| 208 | Sakurada, K.: Konta, T.: Watanabe, M.: Ishizawa, K.: Ueno, Y.: Yamashita, H.: Kayama, T. | 2020 | Associations of Frequency of Laughter With Risk of All-Cause Mortality and Cardiovascular Disease Incidence in a General Population: Findings From the Yamagata Study | Duplicate from August 2020 literature research |
| 209 | Sasaki, M.: Harada, S.: Tsubota, K.: Yasukawa, T.: Takebayashi, T.: Nishiwaki, Y.: Kawasaki, R. | 2020 | Dietary saturated fatty acid intake and early age-related macular degeneration in a Japanese population | Duplicate from August 2020 literature research |
| 210 | Schluter, P. J.: Kokaua, J.: Lee, M. | 2020 | Severe early childhood caries: a modern (neglected) epidemic? | Duplicate from August 2020 literature research |
| 211 | Shidei, H.: Maeda, H.: Isaka, T.: Matsumoto, T.: Yamamoto, T.: Nagashima, Y.: Kanzaki, M. | 2020 | Mediastinal paraganglioma successfully resected by robot-assisted thoracoscopic surgery with en bloc chest wall resection: a case report | Duplicate from August 2020 literature research |
| 212 | Shimizu, Y.: Nabeshima-Kimura, Y.: Kawashiri, S. Y.: Noguchi, Y.: Nagata, Y.: Maeda, T.: Hayashida, N. | 2020 | Anti-thyroid peroxidase antibody and thyroid cysts among the general Japanese population: A cross-sectional study | Duplicate from August 2020 literature research |
| 213 | Song, M.: Ware, R.: Doan, T. N.: Harley, D. | 2020 | Psychotropic medication use in adults with intellectual disability in Queensland, Australia, from 1999 to 2015: a cohort study | Duplicate from August 2020 literature research |
| 214 | Stone, T. J.: Brangan, E.: Chappell, A.: Harrison, V.: Horwood, J. | 2020 | Telephone outreach by community workers to improve uptake of NHS Health Checks in more deprived localities and minority ethnic groups: a qualitative investigation of implementation | Duplicate from August 2020 literature research |
| 215 | Tisi, S.: Dickson, J.: Horst, C.: Hall, H.: Mullin, A.: Farrelly, L.: Gyertson, K.: Levermore, C.: Clarke, C.: Allen, B.: Hamilton, S.: Hartmann, A.: Nair, A.: Devaraj, A.: Hackshaw, A.: Janes, S. | 2020 | SUMMIT study: protocolised management of pulmonary incidental findings in a lung cancer screening cohort | Duplicate from August 2020 literature research |
| 216 | Tomokawa, S.: Asakura, T.: Njenga, S. M.: Njomo, D. W.: Takeuch, R.: Akiyama, T.: Kazama, H.: Mutua, A.: Barnett, W.: Henzan, H.: Shimada, M.: Ichinose, Y.: Kamiya, Y.: Kaneko, S.: Miyake, K.: Kobayashi, J. | 2020 | Examining the appropriateness and reliability of the strategy of the Kenyan Comprehensive School Health Program | Duplicate from August 2020 literature research |
| 217 | Tran-Duplicatey, A.: McDermott, R.: Knight, J.: Hua, X.: Barr, E. L. M.: Arabena, K.: Palmer, A.: Clarke, P. M. | 2020 | Development and Use of Prediction Models for Classification of Cardiovascular Risk of Remote Indigenous Australians | Duplicate from August 2020 literature research |
| 218 | van der Put, A. C.: van der Lippe, T. | 2020 | Work Environment and Worksite Health Promotion in Nine European Countries | Duplicate from August 2020 literature research |
| 219 | Whicher, C. A.: O'Neill, S.: Holt, R. I. G. | 2020 | Diabetes in the UK: 2019 | Duplicate from August 2020 literature research |

| | | | | |
|---|---|---|---|---|
| 220 | Whicher, C. A.: O'Neill, S.: Holt, R. I. G. | 2020 | Diabetes in the UK: 2019 | Duplicate from August 2020 literature research |
| 221 | Zhang, C.: Xie, C. B.: Duplicate, Q.: Hu, H. H.: Liu, X. Q.: Long, J.: Zhong, J. L.: Jiang, W. | 2020 | Prevalence and genotype distribution of human papillomavirus from 9,182 individuals participated health examination | Duplicate from August 2020 literature research |
| 222 | Zhu, H. L.: Liang, X. Y.: Pan, X. F.: Huang, C. Y.: Kuang, J.: Lv, W. B.: Zeng, Q. C.: Mai, W. Y.: Huang, Y. L. | 2020 | A prospective cohort study of home blood pressure monitoring based on an intelligent cloud platform (the HBPM-iCloud study): rationale and design | Duplicate from August 2020 literature research |
| 223 | Zhu, H.: Liang, X.: Pan, X. F.: Huang, C.: Kuang, J.: Lv, W.: Zeng, Q.: Mai, W.: Huang, Y. | 2020 | A prospective cohort study of home blood pressure monitoring based on an intelligent cloud platform (the HBPM-iCloud study): rationale and design | Duplicate from August 2020 literature research |
| 224 | Zulkiewicz, Brittany A.: Burrus, Olivia: Harshbarger, Camilla: Ortiz, Alexa: Garner, Bryan R.: Lewis, Megan A. | 2020 | Identifying implementation strategies that address barriers and facilitate implementation of digital interventions in hiv primary care settings: Results from the pilot implementation of positive health check | Duplicate from August 2020 literature research |

**Literature search update: May 2021**
**Records assessed at full text level and the excluded reasons**

| # | Author | Year | Title | Excluded with reason |
|---|---|---|---|---|
| 1 | Badenbroek, I. F.: Nielen, M. M. J.: Hollander, M.: Stol, D. M.: Kraaijenhagen, R. A.: de Wit, N. J.: Schellevis, F. G. | 2020 | Feasibility and success rates of response enhancing strategies in a stepwise prevention program for cardiometabolic diseases in primary care | B |
| 2 | Kuronen, J.: Winell, K.: Kopra, J.: Rasanen, K. | 2020 | Quality improvement activity in occupational healthcare associated with reduced need for disability retirement: A Bayesian mixed effects modelling study in Finland | B |
| 3 | Williams, M.: Thomson, L.: Butcher, E.: Morriss, R.: Khunti, K.: Packham, C. | 2020 | NHS Health Check Programme: a qualitative study of prison experience | C |
| 4 | de Waard, A. M.: Korevaar, J. C.: Hollander, M.: Nielen, M. M. J.: Seifert, B.: Carlsson, A. C.: Lionis, C.: Søndergaard, J.: Schellevis, F. G.: de Wit, N. J. | 2021 | Unwillingness to participate in health checks for cardiometabolic diseases: A survey among primary health care patients in five European countries | C |
| 5 | Park, B. H.: Lee, B. K.: Ahn, J.: Kim, N. S.: Park, J.: Kim, Y. | 2021 | Association of Participation in Health Check-ups with Risk Factors for Cardiovascular Diseases | C |
| 6 | Thilsing, T.: Larsen, L. B.: Sonderlund, A. L.: Andreassen, S. S.: Christensen, J. R.: Svensson, N. H.: Dahl, M.: Sondergaard, J. | 2021 | Effects of a Co-Design-Based Invitation Strategy on Participation in a Preventive Health Check Program: Randomized Controlled Trial | D |
| 7 | Butler, J.: De Cassan, S.: Lennox, B.: Turner, P.: Glogowska, M.: Fanshawe, T.: Hayward, G. | 2020 | Evaluation of a point of care device in improving physical health check uptake in two community mental health teams | G |
| 8 | Butler, Joseph: de Cassan, Simone: Glogowska, Margaret: Fanshawe, Thomas R.: Turner, Phil: Walton, Debbie: Lasserson, Daniel: Bale, Robert: Lennox, Belinda: Hayward, Gail | 2020 | Effect of point of care blood testing on physical health check completion in mental health services: Mixed-methods evaluation | G |
| 9 | Sallis, A.: Gold, N.: Agbebiyi, A.: James, R. J. E.: Berry, D.: Bonus, A.: Vlaev, I.: Chadborn, T. | 2021 | Increasing uptake of National Health Service Health Checks in primary care: a pragmatic randomized controlled trial of enhanced invitation letters in Northamptonshire, England | Included RCT identified in Aug 2020 literature research |

## Literature search update: June 2023
## Titles and abstracts screening with excluded reasons

| # | Author | Year | Title | Excluded with reason |
|---|---|---|---|---|
| 1 | Abe, M.; Tsunawaki, S.; Dejonckheere, M.; Cigolle, C. T.; Phillips, K.; Rubinstein, E. B.; Matsuda, M.; Fetters, M. D.; Inoue, M. | 2021 | Practices and perspectives of primary care physiciansin Japan and the United States about diagnosing dementia: a qualitative study | A |
| 2 | Adeniyi, A.; Donnelly, L.; Janssen, P.; Jevitt, C.; von Bergman, H.; Brondani, M. | 2021 | A Qualitative Study of Health Care Providers' Views on Integrating Oral Health into Prenatal Care | A |
| 3 | Ahankari, A. S.; Kabra, P.; Tata, L. J.; Hayter, M.; Fogarty, A. W. | 2021 | Two measures of systemic inflammation are positively associated with haemoglobin levels in adolescent girls living in rural India: a cross-sectional study | A |
| 4 | Alghofaili, A. A.; Sudersanadas, K.; Basheer, B.; Philip, W.; Almoubayed, A.; Alotaibi, R.; Aldrees, S. | 2021 | Effect of nutritional factors on dental caries among preschool children in Ngha, Riyadh | A |
| 5 | Amazouz, H.; Roda, C.; Beydon, N.; Lezmi, G.; Bourgoin-Heck, M.; Just, J.; Momas, I.; Rancire, F. | 2021 | Mediterranean diet and lung function, sensitization, and asthma at school age: The PARIS cohort | A |
| 6 | Balata, H.; Ruparel, M.; O'Dowd, E.; Ledson, M.; Janes, S.; Booton, R.; Baldwin, D.; Crosbie, P. | 2021 | MA05.06 Lung Cancer Screening  Cumulative Results from Five UK-Based Programmes | A |
| 7 | Bhutia, T. D.; Mehendale, A.; Lad, N.; Vaishnav, P. | 2021 | Capacity building of teachers: A key to success of Tobacco Free School campaign | A |
| 8 | Borrull-Guardeo, J.; Sebasti-Laguarda, C.; Donat-Colomer, F.; Snchez-Martnez, V. | 2021 | Women's knowledge and attitudes towards cervical cancer prevention: A qualitative study in the Spanish context | A |
| 9 | Callejas, Enrique; Byrne, Sonia; Rodrigo, Mara J. | 2021 | Feasibility and effectiveness of Gaining health & wellbeing from birth to three positive parenting programme | A |
| 10 | Chakrabarti, S.; Pan, A.; Singh, P. | 2021 | Maternal and Child Health Benefits of the Mamata Conditional Cash Transfer Program in Odisha, India | A |
| 11 | Chatterjee, A. S. | 2021 | Health Profile of Mining Workers of an Iron Ore Mine in West Singhbhum District: A Cross-sectional Study on Non- Communicable Diseases | A |
| 12 | Chi, P. C.; Owino, E. A.; Jao, I.; Olewe, F.; Ogutu, B.; Bejon, P.; Kapulu, M.; Kamuya, D.; Marsh, V.; Abdi, A. I.; Abebe, Y.; Audi, A.; Billingsley, P.; Bull, P. C.; Hamaluba, M.; de Laurent, Z.; Hodgson, S. H.; Hoffman, S.; James, E.; Kamuyu, G.; Kariuki, S.; Kibinge, N.; Kimathi, R.; Kinyanjui, S.; Kivisi, C.; Koskei, N.; Imwong, M.; Lowe, B.; Makale, J.; Marsh, K.; Mohammed, K. S.; Mosobo, M.; Murphy, S. C.; Murungi, L.; Musyoki, J.; Muthui, M.; | 2021 | Understanding the benefits and burdens associated with a malaria human infection study in Kenya: experiences of study volunteers and other stakeholders | A |

| | | | | |
|---|---|---|---|---|
| | Mwacharo, J.; Mwanga, D.; Mwongeli, J.; Ndungu, F.; Njue, M.; Njuguna, P.; Nyangweso, G.; Kimani, D.; Ngoi, J. M.; Musembi, J.; Ngoto, O.; Otieno, E.; Osier, F.; Oloo, J.; Omuoyo, D.; Ongecha, J.; Ongas, M. O.; Ooko, M.; Shangala, J.; Sim, B. K. L.; Tarning, J.; Tuju, J.; Wambua, J.; Williams, T. N.; Winterberg, M. | | | |
| 13 | Claus, M.; Antoni, C.; Hofmann, B. | 2021 | Factors associated with elevated alanine aminotransferase in employees of a German chemical company: results of a large cross-sectional study | A |
| 14 | Coombs, N. M.; Missen, K.; Allen, L. | 2021 | Beyond simulation - Extracurricular volunteering in nursing education: A focus group | A |
| 15 | Cooray, U.; Watt, R. G.; Tsakos, G.; Heilmann, A.; Hariyama, M.; Yamamoto, T.; Kuruppuarachchige, I.; Kondo, K.; Osaka, K.; Aida, J. | 2021 | Importance of socioeconomic factors in predicting tooth loss among older adults in Japan: Evidence from a machine learning analysis | A |
| 16 | Cuccu, Zara; Bourne, Tom; AbiAad, Gerrard; Bennett, Samantha | 2021 | Linked data analysis of learning disability health checks and emergency hospital admissions in the Kent Integrated Dataset | A |
| 17 | Dahl, M.; Sondergaard, S. F.; Diederichsen, A.; Pouwer, F.; Pedersen, S. S.; Sondergaard, J.; Lindholt, J. | 2021 | Facilitating participation in cardiovascular preventive initiatives among people with diabetes: a qualitative study | A |
| 18 | Daimaru, K.; Wagatsuma, Y. | 2021 | Hearing loss before and after COVID-19 pandemic | A |
| 19 | Daly, E.; Pearce, A. J.; Ryan, L. | 2021 | A Systematic Review of Strength and Conditioning Protocols for Improving Neck Strength and Reducing Concussion Incidence and Impact Injury Risk in Collision Sports; Is There Evidence? | A |
| 20 | Demirtas, R. N.; Ozel, C. B.; Arslan, A.; Yildirim, G. K. | 2021 | The Experiences of Children/Adolesences with Cystic Fibros During the COVID-19 Pandemic | A |
| 21 | Dickson, J. L.; Quaife, S. L.; Horst, C.; Tisi, S.; Hall, H.; Verghese, P.; Mullin, A.; Sarpong, R.; Teague, J.; Farrelly, L.; Bowyer, V.; Gyertson, K.; Pervez, H.; Bojang, F.; Levermore, C.; Anastasiadis, T.; Sennett, K.; Navani, N.; Hackshaw, A.; Janes, S. M. | 2021 | The summit study: Uptake from re-invitation | A |
| 22 | Duong, L.; Lee, N.; Kim, Y. | 2021 | FP13.01 Assessment of the Fear of COVID-19 and Its Impact on Lung Cancer Screening Participation Among the Korean General Population | A |
| 23 | Duong, T. L.; Lee, N.; Kim, Y.; Kim, Y. | 2021 | Assessment of the fear of COVID-19 and its impact on lung cancer screening participation among the Korean general population | A |
| 24 | Ester, M.; McNeely, M. L.; McDonough, M. H.; Dreger, J.; Culos-Reed, S. N. | 2021 | Protocol: A cluster randomized controlled trial of a mobile application to support physical activity maintenance after an exercise oncology program | A |
| 25 | Eun, Y.; Kim, I. Y.; Han, K. D.; Kang, S. Y.; Lee, S.; Cha, H. S.; Koh, E. M.; Kim, H.; Lee, J. | 2021 | Altered risk of gout according to change of metabolic parameters in young adults | A |

| | | | | |
|---|---|---|---|---|
| 26 | Flynn, A. C.; Pryke, E.; Wadhera, M.; Poston, L.; White, S. L. | 2021 | A preconception intervention targeted at women with modifiable risk factors before pregnancy to improve outcomes; protocol for the Get Ready! feasibility trial | A |
| 27 | Furusawa, T.; Pitakaka, F.; Gabriel, S.; Sai, A.; Tsukahara, T.; Ishida, T. | 2021 | Health and well-being in small island communities: A cross-sectional study in the Solomon Islands | A |
| 28 | Glen, J.; Patel, R. K. | 2021 | Outcomes for potential living kidneydonors the Glasgow experience 2017 2020 | A |
| 29 | Gourlay, E.; Atikins, A.; Grundy, S. | 2021 | 59 Should patients with an unknown smoking status be routinely invited for targeted lung health check? | A |
| 30 | Guo, J.; Li, J. S.; Huang, K. H.; Huang, N.; Feng, X. L. | 2021 | Socio-economic inequalities in the chronic diseases management among Chinese adults aged 45 years and above: a cross sectional study | A |
| 31 | Habukawa, C.; Nagamitsu, S.; Koyanagi, K.; Nishikii, Y.; Yanagimoto, Y.; Yoshida, S.; Suzuki, Y.; Go, S.; Murakami, K. | 2021 | Late bedtime reflects QTA30 anxiety symptoms in adolescents in a school checkup | A |
| 32 | Hamaya, R.; Fukuda, H.; Takebayashi, M.; Mori, M.; Matsushima, R.; Nakano, K.; Miyake, K.; Tani, Y.; Yokokawa, H. | 2021 | Effects of an mHealth app (Kencom) with integrated functions for healthy lifestyles on physical activity levels and cardiovascular risk biomarkers: Observational study of 12,602 users | A |
| 33 | Hardern, A. J. | 2021 | Secondary data analysis between patient's baseline health data and presentation of low back, hip and knee musculoskeletal conditions | A |
| 34 | Harikrishnan, B.; Mithun, C. B.; Srikanth, J.; Bhaskaran, R.; Easwar, S. | 2021 | Determination of significant titre of antiphospholipid antibodies in south indian population | A |
| 35 | Harshbarger, C.; Burrus, O.; Rangarajan, S.; Bollenbacher, J.; Zulkiewicz, B.; Verma, R.; Galindo, C. A.; Lewis, M. A. | 2021 | Challenges of and Solutions for Developing Tailored Video Interventions That Integrate Multiple Digital Assets to Promote Engagement and Improve Health Outcomes: Tutorial | A |
| 36 | Harvie, M.; French, D. P.; Pegington, M.; Cooper, G.; Howell, A.; McDiarmid, S.; Lombardelli, C.; Donnelly, L.; Ruane, H.; Sellers, K.; Barrett, E.; Armitage, C. J.; Evans, D. G. | 2021 | Testing a breast cancer prevention and a multiple disease prevention weight loss programme amongst women within the UK NHS breast screening programmea randomised feasibility study | A |
| 37 | Henry, S. M.; Kopari, N. M.; Peters, L. S. | 2021 | Creative aftercare program amidst coronavirus pandemic | A |
| 38 | Herpertz-Dahlmann, B.; Bonin, E.; Dahmen, B. | 2021 | Can you find the right support for children, adolescents and young adults with anorexia nervosa: Access to age-appropriate care systems in various healthcare systems | A |
| 39 | Hooper, P.; Boulange, C.; Arciniegas, G.; Foster, S.; Bolleter, J.; Pettit, C. | 2021 | Exploring the potential for planning support systems to bridge the research-translation gap between public health and urban planning | A |
| 40 | Hozawa, A.; Tanno, K.; Nakaya, N.; Nakamura, T.; Tsuchiya, N.; Hirata, T.; Narita, A.; Kogure, M.; Nochioka, K.; Sasaki, R.; Takanashi, N.; Otsuka, K.; Sakata, K.; Kuriyama, S.; Kikuya, M.; Tanabe, O.; Sugawara, J.; | 2021 | Study Profile of the Tohoku Medical Megabank Community-Based Cohort Study | A |

| | | | | |
|---|---|---|---|---|
| | Suzuki, K.; Suzuki, Y.; Kodama, E. N.; Fuse, N.; Kiyomoto, H.; Tomita, H.; Uruno, A.; Hamanaka, Y.; Metoki, H.; Ishikuro, M.; Obara, T.; Kobayashi, T.; Kitatani, K.; Takai-Igarashi, T.; Ogishima, S.; Satoh, M.; Ohmomo, H.; Tsuboi, A.; Egawa, S.; Ishii, T.; Ito, K.; Ito, S.; Taki, Y.; Minegishi, N.; Ishii, N.; Nagasaki, M.; Igarashi, K.; Koshiba, S.; Shimizu, R.; Tamiya, G.; Nakayama, K.; Motohashi, H.; Yasuda, J.; Shimizu, A.; Hachiya, T.; Shiwa, Y.; Tominaga, T.; Tanaka, H.; Oyama, K.; Tanaka, R.; Kawame, H.; Fukushima, A.; Ishigaki, Y.; Tokutomi, T.; Osumi, N.; Kobayashi, T.; Nagami, F.; Hashizume, H.; Arai, T.; Kawaguchi, Y.; Higuchi, S.; Sakaida, M.; Endo, R.; Nishizuka, S.; Tsuji, I.; Hitomi, J.; Nakamura, M.; Ogasawara, K.; Yaegashi, N.; Kinoshita, K.; Kure, S.; Sakai, A.; Kobayashi, S.; Sobue, K.; Sasaki, M.; Yamamoto, M. | | | |
| 41 | Huang, H. L.; Chuang, Y. H.; Lin, T. H.; Lin, C.; Chen, Y. H.; Hung, J. Y.; Chan, T. C. | 2021 | Ambient cumulative pm2.5 exposure and the risk of lung cancer incidence and mortality: A retrospective cohort study | A |
| 42 | Hulsegge, G.; Proper, K. I.; Loef, B.; Paagman, H.; Anema, J. R.; van Mechelen, W. | 2021 | The mediating role of lifestyle in the relationship between shift work, obesity and diabetes | A |
| 43 | Hussen, S.; Asnake, S.; Wachamo, D.; Tadesse, B. T. | 2021 | Pneumococcal nasopharyngeal carriage and antimicrobial susceptibility profile in children under five in southern Ethiopia | A |
| 44 | Isin, A.; Turgut, A.; Peden, A. E. | 2021 | Epidemiology of Football-Related Sudden Cardiac Death in Turkey | A |
| 45 | Jain, N.; Lipova, O.; Lipov, A.; Cuming, S. | 2021 | Improvements in paediatric diabetes service using kaizen-a process of continuous improvement using lean tools | A |
| 46 | Jain, N.; Lipova, O.; Lipov, A.; Cuming, S. | 2021 | Improvements in Pediatric Diabetes Service using Kaizen: A process of continuous improvement using lean tools | A |
| 47 | Jin, Seok Won; Lee, Jongwook; Yun Lee, Hee | 2021 | Analyzing factors associated with decisional stage of adopting breast cancer screening among Korean American women using precaution adoption process model | A |
| 48 | Jones, W.; Somerset, S.; Evans, C.; Whittingham, K.; Middleton, M.; Blake, H. | 2021 | Test@work: evaluation of workplace HIV testing for construction workers using the RE-AIM framework | A |
| 49 | Kaimoto, K.; Yamashita, M.; Suzuki, T.; Makizako, H.; Koriyama, C.; Kubozono, T.; Takenaka, T.; Ohishi, M.; Kanouchi, H.; The Tarumizu Study Diet, Group | 2021 | Association of Protein and Magnesium Intake with Prevalence of Prefrailty and Frailty in Community-Dwelling Older Japanese Women | A |
| 50 | Ke, Y.; Xu, J.; Zhang, X.; Guo, Q.; Zhu, Y. | 2021 | Association Between Serum Follicle-Stimulating Hormone and Sarcopenia and Physical Disability Among Older Chinese Men: Evidence From a Cross-Sectional Study | A |

| | | | | |
|---|---|---|---|---|
| 51 | Kim, S. J.; Kwon, O. D.; Kim, K. S. | 2021 | Prevalence, awareness, treatment, and control of dyslipidemia among diabetes mellitus patients and predictors of optimal dyslipidemia control: results from the Korea National Health and Nutrition Examination Survey | A |
| 52 | Kingsbury, A.; Brown, S.; Hunton, C.; Taylor, H.; Esterbrook, G.; Ameri, A. | 2021 | 130 The impact of a smoking cessation intervention in the Wakefield lung health check pilot | A |
| 53 | Kitamura, A.; Seino, S.; Abe, T.; Nofuji, Y.; Yokoyama, Y.; Amano, H.; Nishi, M.; Taniguchi, Y.; Narita, M.; Fujiwara, Y.; Shinkai, S. | 2021 | Sarcopenia: prevalence, associated factors, and the risk of mortality and disability in Japanese older adults | A |
| 54 | Kitetele, F. | 2021 | The peer educator is the game changer of my life: Involving HIV infected adolescents in the disclosure in Congo | A |
| 55 | Kobayashi, H.; Joshita, S.; Akahane, Y.; Matsuzaki, K.; Yamada, H.; Aomura, D.; Joshita, N.; Midorikawa, H.; Suyama, K.; Ota, M.; Wakabayashi, S. I.; Yamashita, Y.; Sugiura, A.; Yamazaki, T.; Misawa, H.; Umemura, T. | 2021 | Protocol: Prospective observational study aiming for micro-elimination of hepatitis C virus in Nagawa town: The Nagawa Project | A |
| 56 | Kobayashi, K.; Ando, K.; Nakashima, H.; Machino, M.; Kanbara, S.; Ito, S.; Inoue, T.; Yamaguchi, H.; Koshimizu, H.; Ishiguro, N.; Hasegawa, Y.; Imagama, S. | 2021 | Overcoming locomotive syndrome: The Yakumo Study | A |
| 57 | Kogure, M.; Nakaya, N.; Hirata, T.; Tsuchiya, N.; Nakamura, T.; Narita, A.; Suto, Y.; Honma, Y.; Sasaki, H.; Miyagawa, K.; Ushida, Y.; Ueda, H.; Hozawa, A. | 2021 | Sodium/potassium ratio change was associated with blood pressure change: finding from health check-up data | A |
| 58 | Kondhalkar, A.; Ambad, R.; Bhatt, N.; Jha, R. K. | 2021 | Determination of Clinical Utility of Novel Biochemical Markers in Osteoarthritis | A |
| 59 | Kuhlberg, H.; Kujala, S.; Hrhammer, I.; Koskela, T. | 2021 | STAR Duodecim eHealth Tool to Recognize Chronic Disease Risk Factors and Change Unhealthy Lifestyle Choices Among the Long-Term Unemployed: Protocol for a Mixed Methods Validation Study | A |
| 60 | Kumalo, A.; Gambura, E.; Dodicho, T.; Ahmed, K. S.; Balcha, T.; Beshir, B.; Abraham, M. | 2021 | Prevalence of Intestinal Parasites and Salmonella typhi among Food Handlers Working in Catering Establishments of Public Institutes Found in Dawuro Zone, South-Western Ethiopia | A |
| 61 | Kuriyama, N.; Ozaki, E.; Koyama, T.; Matsui, D.; Watanabe, I.; Inaba, M.; Yamada, S.; Horii, M.; Uehara, R. | 2021 | Evaluation of myostatin as a possible regulator of the skeletal muscle-cortical bone interaction in adults | A |
| 62 | Kuriyama, N.; Ozaki, E.; Koyama, T.; Matsui, D.; Watanabe, I.; Tomida, S.; Nagamitsu, R.; Hashiguchi, K.; Inaba, M.; Yamada, S.; Horii, M.; Mizuno, S.; Yoneda, Y.; Kurokawa, M.; Kobayashi, D.; Fukuda, S.; Iwasa, K.; Watanabe, Y.; Uehara, R. | 2021 | Evaluation of myostatin as a possible regulator and marker of skeletal musclecortical bone interaction in adults | A |
| 63 | Kwon, S.; Lee, H. J.; Han, K. D.; Kim, D. H.; Lee, S. P.; Hwang, I. C.; Yoon, Y.; Park, J. B.; Lee, H.; Kwak, S.; Yang, S.; Cho, G. Y.; Kim, Y. J.; Kim, H. K.; Ommen, S. R. | 2021 | Association of physical activity with all-cause and cardiovascular mortality in 7666 adults with hypertrophic cardiomyopathy (HCM): more physical activity is better | A |

| | | | | |
|---|---|---|---|---|
| 64 | Laso-Alonso, A. E.; Mata-Zubillaga, D.; Gonzalez-Garcia, L. G.; Rodriguez-Manchon, S.; Corral-Hospital, S.; Garcia-Aparicio, C. | 2021 | Impact of COVID-19s alarm states in the care of pediatric patients in Primary Care in a health area in northern Spain | A |
| 65 | Laub, O.; Leipold, G.; Toncheva, A. A.; Peterhoff, D.; Einhauser, S.; Neckermann, P.; Borchers, N.; Santos-Valente, E.; Kheiroddin, P.; Buntrock-Dpke, H.; Laub, S.; Schberl, P.; Schweiger-Kabesch, A.; Ewald, D.; Horn, M.; Niggel, J.; Ambrosch, A.; berla, K.; Gerling, S.; Brandstetter, S.; Wagner, R.; Kabesch, M. | 2021 | Symptoms, SARS-CoV-2 Antibodies, and Neutralization Capacity in a Cross Sectional-Population of German Children | A |
| 66 | Leach, A. J.; Morris, P. S.; Coates, H. L. C.; Nelson, S.; O'Leary, S. J.; Richmond, P. C.; Gunasekera, H.; Harkus, S.; Kong, K.; Brennan-Jones, C. G.; Brophy-Williams, S.; Currie, K.; Das, S. K.; Isaacs, D.; Jarosz, K.; Lehmann, D.; Pak, J.; Patel, H.; Perry, C.; Reath, J. S.; Sommer, J.; Torzillo, P. J. | 2021 | Otitis media guidelines for Australian Aboriginal and Torres Strait Islander children: summary of recommendations | A |
| 67 | Lee, J. H.; Park, H. M.; Lee, Y. J. | 2021 | Using Dietary Macronutrient Patterns to Predict Sarcopenic Obesity in Older Adults: A Representative Korean Nationwide Population-Based Study | A |
| 68 | Liptovszky, M.; Dobbs, P.; Moittie, S. | 2021 | Acute fatal upper respiratory obstruction in a chimpanzee (Pan troglodytes) during anaesthesia Case report | A |
| 69 | Liu, Y. T.; Wang, W.; Tong, J.; Wang, B. Y. | 2021 | Relationship between triglyceride-glucose index and non-alcoholic fatty liver disease | A |
| 70 | Ma, M.; Adeney, M.; Long, H. | 2021 | Functional Settings of Hospital Outdoor Spaces and the Perceptions from Public and Hospital Occupant during COVID-19 | A |
| 71 | Maruta, M.; Makizako, H.; Ikeda, Y.; Miyata, H.; Nakamura, A.; Han, G.; Shimokihara, S.; Tokuda, K.; Kubozono, T.; Ohishi, M.; Tabira, T. | 2021 | Association between apathy and satisfaction with meaningful activities in older adults with mild cognitive impairment: Apopulation-based cross-sectional study | A |
| 72 | McShea, Lynzee; Giles, Karen; Murphy, Ashley; Ling, Jonathan | 2021 | An alternative approach for detecting hearing loss in adults with learning disabilities | A |
| 73 | Meguro, K.; Svensson, T.; Chung, U. I.; Svensson, A. K. | 2021 | Associations of work-related stress and total sleep time with cholesterol levels in an occupational cohort of Japanese office workers | A |
| 74 | Mills, K.; Paxton, B.; Walter, F. M.; Griffin, S. J.; Sutton, S.; Usher-Smith, J. A. | 2021 | Incorporating a brief intervention for personalised cancer risk assessment to promote behaviour change into primary care: a multi-methods pilot study | A |
| 75 | Min, K. D.; Kim, J. S.; Park, Y. H.; Shin, H. Y.; Kim, C.; Seo, S. W.; Kim, S. Y. | 2021 | New assessment for residential greenness and the association with cortical thickness in cognitively healthy adults | A |
| 76 | Moittie, S.; Dobbs, P.; Redrobe, S.; Liptovszky, M.; White, K. | 2021 | Evaluation of the agreement of two oscillometric blood pressure devices with invasive blood pressure in anaesthetized chimpanzees (Pan troglodytes) | A |

| | | | | |
|---|---|---|---|---|
| 77 | Moral Pelez, I.; Brotons Cuixart, C.; Fernndez Valverde, D.; Puig Palma, M.; Calvo Bonacho, E.; Martnez Muoz, P.; Catalina Romero, C.; Quevedo Aguado, L. J. | 2021 | External validation of the European and American equations for calculating cardiovascular risk in a Spanish working population | A |
| 78 | Moriyama, M.; Kazawa, K.; Jahan, Y.; Ikeda, M.; Mizukawa, M.; Fukuoka, Y.; Harada, K.; Rahman, M. M. | 2021 | The Effectiveness of Telenursing for Self-Management Education on Cardiometabolic Conditions: A Pilot Project on a Remote Island of sakikamijima, Japan | A |
| 79 | Nakanishi, K.; Daimon, M.; Yoshida, Y.; Sawada, N.; Hirose, K.; Iwama, K.; Yamamoto, Y.; Ishiwata, J.; Hirokawa, M.; Kaneko, H.; Nakao, T.; Mizuno, Y.; Morita, H.; Di Tullio, M. R.; Homma, S.; Komuro, I. | 2021 | Subclinical Hypothyroidism as an Independent Determinant of Left Atrial Dysfunction in the General Population | A |
| 80 | Noguchi, T.; Nojima, I.; Inoue-Hirakawa, T.; Sugiura, H. | 2021 | Role of nonface-to-face social contacts in moderating the association between living alone and mental health among community-dwelling older adults: a cross-sectional study | A |
| 81 | Noguchi, T.; Nojima, I.; Inoue-Hirakawa, T.; Sugiura, H. | 2021 | Role of non-face-to-face social contacts in moderating the association between living alone and mental health among community-dwelling older adults: a cross-sectional study | A |
| 82 | O'Kane, N.; Mesaritis, A.; Peacock, M.; Johnson, G. | 2021 | Reducing barriers, improving awareness: Primary care liaison meeting pilot | A |
| 83 | Okamoto, C.; Hasegawa, T.; Tsukamoto, O.; Hitsumoto, T.; Matsuoka, K.; Takashima, S.; Amaki, M.; Kanzaki, H.; Izumi, C.; Ito, S.; Kitakaze, M. | 2021 | Low plasma levels of B-type natriuretic peptide predict the insulin resistance and left ventricular concentric remodeling in subjects without heart diseases: the observational arita cohort study | A |
| 84 | Okoli, G. N.; Lam, O. L. T.; Abdulwahid, T.; Neilson, C. J.; Mahmud, S. M.; Abou-Setta, A. M. | 2021 | Seasonal influenza vaccination among cancer patients: A systematic review and meta-analysis of the determinants | A |
| 85 | Omae, K.; Kurita, N.; Takeshima, T.; Naganuma, T.; Takahashi, S.; Yoshioka, T.; Ohnishi, T.; Ito, F.; Hamaguchi, S.; Fukuhara, S. | 2021 | Overactive bladder with/without urinary incontinence and falls in the community-dwelling elderly | A |
| 86 | Otaki, Y.; Watanabe, T.; Konta, T.; Watanabe, M.; Asahi, K.; Yamagata, K.; Fujimoto, S.; Tsuruya, K.; Narita, I.; Kasahara, M.; Shibagaki, Y.; Iseki, K.; Moriyama, T.; Kondo, M.; Watanabe, T. | 2021 | One-year change in plasma volume and mortality in the Japanese general population: An observational cohort study | A |
| 87 | Otaki, Y.; Watanabe, T.; Konta, T.; Watanabe, M.; Asahi, K.; Yamagata, K.; Fujimoto, S.; Tsuruya, K.; Narita, I.; Kasahara, M.; Shibagaki, Y.; Iseki, K.; Moriyama, T.; Kondo, M.; Watanabe, T. | 2021 | Impact of chronic kidney disease on aortic disease-related mortality: A four-year community-based cohort study | A |
| 88 | Otaki, Y.; Watanabe, T.; Konta, T.; Watanabe, M.; Fujimoto, S.; Sato, Y.; Asahi, K.; Yamagata, K.; Tsuruya, K.; Narita, I.; Kasahara, M.; Shibagaki, Y.; Iseki, K.; Moriyama, T.; Kondo, M.; Watanabe, T. | 2021 | One-year change in diastolic blood pressure and aortic disease-related mortality in a japanese general population aged 50-75 years | A |

| | | | | |
|---|---|---|---|---|
| 89 | Park, H. S.; Kim, K. I.; Chung, H. Y.; Jeong, S.; Soh, J. Y.; Hyun, Y. H.; Kim, H. S. | 2021 | A Worker-Centered Personal Health Record App for Workplace Health Promotion Using National Health Care Data Sets: Design and Development Study | A |
| 90 | Park, S.; Greene, M. C.; Melby, M. K.; Fujiwara, T.; Surkan, P. J. | 2021 | Postpartum Depressive Symptoms as a Mediator Between Intimate Partner Violence During Pregnancy and Maternal-Infant Bonding in Japan | A |
| 91 | Parker, Lauren J.; Marx, Katherine; Gaugler, Joseph E.; Gitlin, Laura N. | 2021 | Implications of the COVID-19 pandemic on adult day services and the families they serve | A |
| 92 | Pelaez, I. M.; Cuixart, C. B.; Valverde, D. F.; Palma, M. P.; Bonacho, E. C.; Munoz, P. M.; Romero, C. C.; Aguado, L. J. Q. | 2021 | External validation of the European and American equations for calculating cardiovascular risk in a Spanish working population | A |
| 93 | Perez, M. S. R.; Rosales, J. S.; Dossi, D. D.; Ameriso, S. F. | 2021 | Control of vascular risk factors and response to stroke symptoms during the most restrictive period of the COVID-19 Quarantine. A survey of population behavior in Argentina | A |
| 94 | Prattipati, S.; Mlangi, J. J.; Tarimo, T. G.; Kweka, G. L.; Thielman, N. M.; Bettger, J. P.; Mmbaga, B. T.; Sakita, F. M.; Hertz, J. T. | 2021 | Knowledge, attitudes, and preventive practices regarding ischemic heart disease among HIV-positive individuals in northern Tanzania | A |
| 95 | Quaife, S.; Dickson, J. L.; Brain, K. E.; Kurtidu, C.; McCabe, J.; Hackshaw, A.; Duffy, S. W.; Janes, S. M.; Waller, J. | 2021 | Psychological correlates of lung cancer screening uptake: Baseline data from a prospective screening uptake behaviour cohort study | A |
| 96 | Quaife, S. L.; Waller, J.; Dickson, J. L.; Brain, K. E.; Kurtidu, C.; McCabe, J.; Hackshaw, A.; Duffy, S. W.; Janes, S. M. | 2021 | Psychological Targets for Lung Cancer Screening Uptake: A Prospective Longitudinal Cohort Study | A |
| 97 | Rachdi, M.; Feki, A.; Gassara, Z.; Ben Jemaa, S.; Ghali, M.; Ezzeddine, M.; Kallel, M. H.; Fourati, H.; Akrout, R.; Baklouti, S. | 2021 | Impact of covid 19 pandemic on Tunisian spa patients: Psychological state and treatment adherence | A |
| 98 | Raza, S.; Hussain, I.; Barnett, J.; Gidlow, C.; Owen, G.; Hanna, F.; Fryer, A. | 2021 | Coronary calcification and HbA1c in the north Staffordshire cohort of the UK-based national targeted lung health check | A |
| 99 | Renjhen, P.; Kaundal, A.; Rathi, A.; Kumar, V. | 2021 | Impact of health education on knowledge and attitude of school teachers regarding cervical cancer, hpv vaccine and cervical cancer screening | A |
| 100 | Roman-Urrestarazu, A.; Yanez, C.; Lopez-Gari, C.; Elgueta, C.; Allison, C.; Brayne, C.; Troncoso, M.; Baron-Cohen, S. | 2021 | Autism screening and conditional cash transfers in Chile: Using the Quantitative Checklist (Q-CHAT) for early autism detection in a low resource setting | A |
| 101 | Ryu, K. J.; Yi, K. W.; Kim, Y. J.; Shin, J. H.; Hur, J. Y.; Kim, T.; Seo, J. B.; Lee, K. S.; Park, H. | 2021 | Machine Learning Approaches to Identify Factors Associated with Women's Vasomotor Symptoms Using General Hospital Data | A |
| 102 | Saavedra-Aracena, L.; Grimm-Seyfarth, A.; Schuttler, E. | 2021 | Do dog-human bonds influence movements of free-ranging dogs in wilderness? | A |

| | | | | |
|---|---|---|---|---|
| 103 | Sakamoto, Y.; Shimoyama, S.; Furukawa, T.; Adachi, M.; Takahashi, M.; Mikami, T.; Kuribayashi, M.; Osato, A.; Tsushima, D.; Saito, M.; Ueno, S.; Nakamura, K. | 2021 | Copy number variations in Japanese children with autism spectrum disorder | A |
| 104 | Sakurai, A.; Yamada, S. I.; Karasawa, I.; Kondo, E.; Kurita, H. | 2021 | Accuracy of a salivary examination kit for the screening of periodontal disease in a group medical check-up (Japanese-specific health check-up) | A |
| 105 | Sasaki, K.; Hirasawa, A.; Yamazaki, Y.; Ishikawa, M. | 2021 | Analysis of sweet snack eating habits and lifestyle using a health check for toddlers | A |
| 106 | Shapira, U.; Brezinski, R. Y.; Rogowski, O.; Zeltser, D.; Berliner, S.; Shapira, I.; Shenhar-Tsarfaty, S.; Fireman, E. | 2021 | Association between elevated serum bilirubin levels with preserved lung function under conditions of exposure to air pollution | A |
| 107 | Shimizu, Y.; Arima, K.; Noguchi, Y.; Kawashiri, S. Y.; Yamanashi, H.; Tamai, M.; Nagata, Y.; Maeda, T. | 2021 | Possible mechanisms underlying the association between human T-cell leukemia virus type 1 (HTLV-1) and hypertension in elderly Japanese population | A |
| 108 | Soiland-Reyes, C.; O'Flaherty, M.; Kypridemos, C. | 2021 | IMPLEMENTING A NATIONAL WEIGHT-REDUCTION LIFESTYLE INTERVENTION TO THE NHS HEALTH CHECKS POPULATION: LONG-TERM OUTCOMES MICROSIMULATION MODELLING OF THE NHS DIABETES PREVENTION PROGRAMME | A |
| 109 | Somerset, S.; Evans, C.; Blake, H. | 2021 | Accessing voluntary hiv testing in the construction industry: A qualitative analysis of employee interviews from the test@work study | A |
| 110 | Sonu, ; Devi, A. M.; Grover, A.; Deepak, | 2021 | Assess the knowledge, utilization & barrier of non-utilization regarding maternal health services | A |
| 111 | Storholm, E. D.; Ober, A. J.; Mizel, M. L.; Matthews, L.; Sargent, M.; Todd, I.; Zajdman, D.; Green, H. | 2021 | Primary care providers knowledge, attitudes, and beliefs about HIV pre-exposure prophylaxis (PrEP): Informing network-based interventions | A |
| 112 | Sultana, S.; Sah, B. N. K.; Kumar, J. | 2021 | A Study on The Assessment of Utilization of the Antenatal Services and Associated Barriers Among Mothers in Northern Area of Bihar | A |
| 113 | Taira, K.; Mori, T.; Ishimaru, M.; Iwagami, M.; Sakata, N.; Watanabe, T.; Takahashi, H.; Tamiya, N. | 2021 | Regional Inequality in Dental Care Utilization in Japan: An Ecological Study Using the National Database of Health Insurance Claims | A |
| 114 | Takahashi, J.; Kawai, H.; Fujiwara, Y.; Watanabe, Y.; Hirano, H.; Kim, H.; Ihara, K.; Ejiri, M.; Ishii, K.; Oka, K.; Obuchi, S. | 2021 | Association between activity diversity and frailty among community-dwelling older Japanese: A cross-sectional study | A |
| 115 | Takami, A.; Watanabe, S.; Yamamoto, Y.; Miyachi, H.; Bamba, Y.; Ohata, M.; Mishima, S.; Kubota, H.; Nishiura, A.; Inaba, T.; Enomoto, M.; Mitsuhashi, T.; Nakanishi, K.; Miura, R.; Nonaka, E.; Shimbo, K.; Yatomi, Y.; Tohyama, K. | 2021 | Reference intervals of white blood cell parameters for healthy adults in japan | A |
| 116 | Takami, A.; Watanabe, S.; Yamamoto, Y.; Miyachi, H.; Bamba, Y.; Ohata, M.; Mishima, S.; Kubota, H.; Nishiura, A.; Inaba, T.; Enomoto, M.; Mitsuhashi, T.; Nakanishi, K.; | 2021 | Reference intervals of white blood cell parameters for healthy adults in japan | A |

| | | | | |
|---|---|---|---|---|
| | Miura, R.; Nonaka, E.; Shimbo, K.; Yatomi, Y.; Tohyama, K.; Japanese Soc Lab Hematology, Subcom | | | |
| 117 | Takao, Toshihiro; Sumi, Naoki; Yamanaka, Yoshiyuki; Fujimoto, Sohachi; Kamada, Tomoari | 2021 | Associations between lifestyle behaviour changes and the optimal well-being of middle-aged Japanese individuals | A |
| 118 | Takizawa, M.; Kawachi, I.; Fujiwara, T.; Kizuki, M.; Nawa, N.; Kino, S. | 2021 | Association Between Maternal Working Status and Unintentional Injuries Among 3 to 4-Month-Old Infants in Japan | A |
| 119 | Takura, T.; Hirano Goto, K.; Honda, A. | 2021 | Development of a predictive model for integrated medical and long-term care resource consumption based on health behaviour: application of healthcare big data of patients with circulatory diseases | A |
| 120 | Tammemagi, M. | 2021 | ES12.02 Defining High Risk | A |
| 121 | Tan, Y. R.; Tan, E. H.; Jawahir, S.; Mohd Hanafiah, A. N.; Mohd Yunos, M. H. | 2021 | Demographic and socioeconomic inequalities in oral healthcare utilisation in Malaysia: evidence from a national survey | A |
| 122 | Tan, Y. R.; Tan, E. H.; Jawahir, S.; Hanafiah, A. N. M.; Yunos, M. H. M. | 2021 | Demographic and socioeconomic inequalities in oral healthcare utilisation in Malaysia: evidence from a national survey | A |
| 123 | Tanaka, C.; Wakaizumi, K.; Kosugi, S.; Tanaka, S.; Matsudaira, K.; Morisaki, H.; Mimura, M.; Fujisawa, D. | 2021 | Association of work performance and interoceptive awareness of 'body trusting' in an occupational setting: A cross-sectional study | A |
| 124 | Tanaka, M.; Saito, M.; Takahashi, M.; Adachi, M.; Nakamura, K. | 2021 | Interformat Reliability of Web-Based Parent-Rated Questionnaires for Assessing Neurodevelopmental Disorders Among Preschoolers: Cross-sectional Community Study | A |
| 125 | Taylan, S.; zkan, ; Adbelli, D. | 2021 | Breast cancer perception scale: Psychometric development study | A |
| 126 | Tekavec, E.; Lfqvist, L.; Larsson, A.; Fisk, K.; Riddar, J.; Nilsson, T.; Nordander, C. | 2021 | Adverse health manifestations in the hands of vibration exposed carpenters - a cross sectional study | A |
| 127 | Terui, T.; Yoshida, K.; Sasaki, M.; Murakami, M.; Goto, A. | 2021 | The Association Between Fathers' Self-assessment of Their Own Parenting and Mothers' Recognition of Paternal Support: A Municipal-Based Cross-Sectional Study | A |
| 128 | Tomita, Y.; Sakata, S.; Arima, H.; Yamato, I.; Ibaraki, A.; Ohtsubo, T.; Matsumura, K.; Fukuhara, M.; Goto, K.; Kitazono, T. | 2021 | Relationship between casual serum triglyceride levels and the development of hypertension in Japanese | A |
| 129 | Trenta, A. M.; Belloni, S.; Ausili, D.; Caruso, R.; Arrigoni, C.; Vellone, E.; Moro, M.; Dellafiore, F. | 2021 | The lived experience of patients with a left ventricular assist device during the COVID-19 pandemic: A qualitative study | A |
| 130 | Tsou, M. T.; Chang, Y. C.; Hsu, C. P.; Kuo, Y. C.; Yun, C. H.; Huang, W. H.; Hu, K. C.; Liu, C. Y.; Chen, Y. J.; Sung, K. T.; Liu, C. C.; Hung, C. L.; Kuo, J. Y.; Chen, T. Y.; Hung, T. C.; Yeh, H. I. | 2021 | Visceral adiposity index outperforms conventional anthropometric assessments as predictor of diabetes mellitus in elderly Chinese: a population-based study | A |
| 131 | Tsuboi, Y.; Yamada, H.; Munetsuna, E.; Fujii, R.; Yamazaki, M.; Ando, Y.; Mizuno, G.; Ishikawa, H.; Ohashi, K.; Hashimoto, S.; Hamajima, N.; Suzuki, K. | 2021 | Global DNA hypermethylation in peripheral blood mononuclear cells and cardiovascular disease risk: a population-based propensity score-matched cohort study | A |

| | | | | |
|---|---|---|---|---|
| 132 | Usher-Smith, A. J.; Haggstrom, C.; Wennberg, P.; Lindvall, K.; Strelitz, J.; Sharp, J. S.; Griffin, J. S. | 2021 | Impact of achievement and change in achievement of lifestyle recommendations in middle-age on risk of the most common potentially preventable cancers | A |
| 133 | van der Steeg, G. E.; Takken, T. | 2021 | Reference values for maximum oxygen uptake relative to body mass in Dutch/Flemish subjects aged 6-65years: the LowLands Fitness Registry | A |
| 134 | van Erp, L. W.; Groenen, M. J. M.; Heida, W.; Wisse, J.; Roosenboom, B.; Wahab, P. J. | 2021 | Mobile application to monitor inflammatory bowel disease patients on intravenous biologic treatment: a feasibility study | A |
| 135 | Van Stappen, V.; Cardon, G.; De Craemer, M.; Mavrogianni, C.; Usheva, N.; Kivel, J.; Wikstrm, K.; De Miquel-Etayo, P.; Gonzlez-Gil, E. M.; Rad, A. S.; Nnsi, A.; Iotova, V.; Manios, Y.; Brondeel, R. | 2021 | The effect of a cluster-randomized controlled trial on lifestyle behaviors among families at risk for developing type 2 diabetes across Europe: the Feel4Diabetes-study | A |
| 136 | Waade, J.; Seibt, U.; Honscha, W.; Rachidi, F.; Starke, A.; Speck, S.; Truyen, U. | 2021 | Multidrug-resistant enterobacteria in newborn dairy calves in Germany | A |
| 137 | Wakasugi, M.; Narita, I.; Iseki, K.; Asahi, K.; Yamagata, K.; Fujimoto, S.; Moriyama, T.; Konta, T.; Tsuruya, K.; Kasahara, M.; Shibagaki, Y.; Kondo, M.; Watanabe, T. | 2021 | The effect of CKD on associations between lifestyle factors and all-cause, cancer, and cardiovascular mortality: A population-based cohort study | A |
| 138 | Wanjari, M. B.; Mendhe, D. | 2021 | Prevalence Rate of Hepatitis C Among the Solid Waste Handler in Wardha City | A |
| 139 | Wei, L.; Cheng, X.; Luo, Y.; Yang, R.; Lei, Z.; Jiang, H.; Chen, L. | 2021 | Lean non-alcoholic fatty liver disease and risk of incident diabetes in a euglycaemic population undergoing health check-ups: A cohort study | A |
| 140 | Wernette, G. T.; Countryman, K.; Mmeje, O.; Ngo, Q. M.; Zlotnick, C. | 2021 | Adapting to the Pandemic: Protocol of a Web-Based Perinatal Health Study to Improve Maternal and Infant Outcomes | A |
| 141 | Wong, T. Y. E. | 2021 | Factors associated with the presence of non-alcoholic fatty liver disease (nafld) detected in japanese adults undergoing health check-ups | A |
| 142 | Xiao, W.; Xueming, H.; Lei, W.; Xinyi, H.; Quanzhong, Y. | 2021 | Analysis of factors of atherosclerotic cardiovascular disease risk in patients with type 2 diabetes based on community health screening | A |
| 143 | Yahata, Y.; Fielding, J. E.; Kamiya, H.; Takimoto, N.; Ishii, J.; Fukusumi, M.; Sunagawa, T. | 2021 | Factors associated with knowledges and attitudes about measles and rubella immunization in a non-health care occupational setting in Japan | A |
| 144 | Yeap, S. S.; Thambiah, S. C.; Suppiah, S.; Md-Said, S.; Appannah, G.; Samsudin, I. N.; Zainuddin, N.; Zahari-Sham, S. Y.; Hew, F. L. | 2021 | Asymptomatic morphometric vertebral fractures and its associated factors: A cross-sectional study among adults in a selected urban area in Selangor, Malaysia | A |
| 145 | Yokokawa, H.; Fukuda, H.; Saita, M.; Goto, K.; Kaku, T.; Miyagami, T.; Takahashi, Y.; Hamada, C.; Hisaoka, T.; Naito, T. | 2021 | An association between visceral or subcutaneous fat accumulation and diabetes mellitus among Japanese subjects | A |
| 146 | Zeng, Y.; He, H.; Wang, X.; Zhang, M.; An, Z. | 2021 | Climate and air pollution exposure are associated with thyroid function parameters: a retrospective cross-sectional study | A |
| 147 | Zhang, M.; Bao, H.; Wang, L.; Zhao, Z.; Huang, Z.; Zhang, X.; Li, C.; Zhou, M.; Wu, J.; Wang, L. | 2021 | Analysis of cervical cancer screening and related factors in China | A |

| | | | | |
|---|---|---|---|---|
| 148 | Zulkiewicz, B. A.; Burrus, O.; Harshbarger, C.; Ortiz, A.; Garner, B. R.; Lewis, M. A. | 2021 | Identifying Implementation Strategies That Address Barriers and Facilitate Implementation of Digital Interventions in HIV Primary Care Settings: Results from the Pilot Implementation of Positive Health Check | A |
| 149 | Abu Awwad, D.; Hossain, S. Z.; Mackey, M.; Brennan, P.; Adam, S. | 2022 | Exploring the role of healthcare organisations in increasing women's participation in breast-screening in the United Arab Emirates | A |
| 150 | Ahcioglu, A.; Yilmazel, G. | 2022 | Health literacy, behavioral and psychosocial characteristics in coronary artery patients: A hospital-based study in Turkey | A |
| 151 | Altunkalem Seydi, K.; Ates Bulut, E.; Yavuz, I.; Kavak, H.; Kaya, D.; Isik, A. T. | 2022 | E-mail-based health care in patients with dementia during the pandemic | A |
| 152 | Arcucci, M. S.; Gallo, J.; Messere, G.; Busoni, V.; Bigliardi, R.; Orsi, M. | 2022 | Differences in psychosocial aspects in patients with and without pediatric IBD due to the COVID-19 pandemic in Argentina. A two center study | A |
| 153 | Bae, W.; Lee, C. H.; Lee, J.; Kim, Y. W.; Han, K.; Choi, S. M. | 2022 | Impact of smoking on the development of idiopathic pulmonary fibrosis: results from a nationwide population-based cohort study | A |
| 154 | Balata, H.; Bradley, P.; Alonso, A.; Hewitt, K.; Booton, R.; Crosbie, P. | 2022 | Lung cancer detection in the expanded Manchester Lung Health Check programme | A |
| 155 | Bhamani, A.; Dickson, J. L.; Horst, C.; Tisi, S.; Hall, H.; Creamer, A.; Prendecki, R.; McCabe, J.; Quaife, S. L.; Bojang, F.; Arancon, D.; Gyertson, K.; Mullin, A.; Teague, J.; Farrelly, L.; Hackshaw, A.; Janes, S. M. | 2022 | Prevalence and demographics of marijuana users in a Lung Cancer Screening cohort | A |
| 156 | Boffetta, P.; Collatuzzo, G. | 2022 | Application of P4 (Predictive, Preventive, Personalized, Participatory) Approach to Occupational Medicine | A |
| 157 | Bhm, A.; Rinner, S.; Missmann, T.; Huemer, M.; Keil, F. | 2022 | IONA - Interdisciplinary Oncological Follow-Up Clinic in Vienna | A |
| 158 | Boitano, T.; Evans, E.; Gardner, A.; Leath, C.; Straughn, M.; Smith, H. | 2022 | Use of a patient engagement technology platform improves gynecologic oncology patient outcomes in the perioperative setting (001) | A |
| 159 | Boopathi, S.; Raja, P. K. K.; Balasubramaniam, S. S.; Sanjaikrishna, P. K. | 2022 | QUALITY OF SLEEP ASSESSMENT IN MASTER HEALTH CHECK-UP PATIENTS USING PIRS-20 SCALE | A |
| 160 | Bradley, P.; Balata, H.; Alonso, A.; Booton, R.; Crosbie, P. A. | 2022 | P1.04-01 Risk Stratification for Personalised Screening Intervals: Performance of PLCOm2012NoRace at Second Round of Manchester LHC | A |
| 161 | Bukman, A. J. | 2022 | Targeting persons with low socioeconomic status of different ethnic origins with lifestyle interventions : Opportunities and effectiveness | A |
| 162 | Bustamante Loyola, J.; Prez Retamal, M.; Mendiburo-Seguel, A.; Guedeney, A. C.; Salinas Gonzlez, R.; Muoz, L.; Cox Melane, H.; Gonzlez Mas, J. M.; Sim Teufel, S.; Morgues Nudman, M. | 2022 | The Impact of an Interactive Guidance Intervention on Sustained Social Withdrawal in Preterm Infants in Chile: Randomized Controlled Trial | A |
| 163 | Buttery, S. C.; Williams, P.; Mweseli, R.; Philip, K. E. J.; Sadaka, A.; Bartlett, E. J.; Devaraj, A.; Kemp, S.; Addis, J.; Derbyshire, J.; Chen, M.; Morris, K.; Laverty, A.; Hopkinson, N. S. | 2022 | Immediate smoking cessation support versus usual care in smokers attending a targeted lung health check: the QuLIT trial | A |

| | | | | |
|---|---|---|---|---|
| 164 | Cao, W.; Fakile, Y.; Shukla, M. R.; Pettus, K.; Lupoli, K.; Hong, J.; Pillay, A.; Ballard, R.; Blondeel, K.; Toskin, I.; Kersh, E. | 2022 | External Quality Assessment to Support the WHO ProsPeRo Study for Evaluating HIV/Syphilis Point-of-Care Testing in 7 Countries | A |
| 165 | Cavers, D.; Nelson, M.; Rostron, J.; Robb, K. A.; Brown, L. R.; Campbell, C.; Akram, A. R.; Dickie, G.; Mackean, M.; van Beek, E. J. R.; Sullivan, F.; Steele, R. J.; Neilson, A. R.; Weller, D. | 2022 | Optimizing the implementation of lung cancer screening in Scotland: Focus group participant perspectives in the LUNGSCOT study | A |
| 166 | Creamer, A.; Horst, C.; Dickson, J.; Tisi, S.; Hall, H.; Verghese, P.; Prendecki, R.; Bhamani, A.; Teague, J.; Farrelly, L.; Gyertson, K.; Mullin, A. M.; Devaraj, A.; Nair, A.; Hackshaw, A.; Janes, S. | 2022 | Quality assurance of radiology reporting in lung cancer screening: the role of a radiology review meeting | A |
| 167 | Crosbie, P. A. J.; Gabe, R.; Simmonds, I.; Hancock, N.; Alexandris, P.; Kennedy, M.; Rogerson, S.; Baldwin, D.; Booton, R.; Bradley, C.; Darby, M.; Eckert, C.; Franks, K. N.; Lindop, J.; Janes, S. M.; Mller, H.; Murray, R. L.; Neal, R. D.; Quaife, S. L.; Upperton, S.; Shinkins, B.; Tharmanathan, P.; Callister, M. E. J. | 2022 | Participation in community-based lung cancer screening: the Yorkshire Lung Screening Trial | A |
| 168 | Davis, M.; Jones, J. D.; So, A.; Benton, T. D.; Boyd, R. C.; Melhem, N.; Ryan, N. D.; Brent, D. A.; Young, J. F. | 2022 | Adolescent depression screening in primary care: Who is screened and who is at risk? | A |
| 169 | Den Harink, T.; Hoek, A.; Groen, H.; Roseboom, T. J.; Deutekom, A. V. | 2022 | Which factors play a role in the decision of mothers to participate in child follow-up examinations after participation in an RCT?: A semi-quantitative study | A |
| 170 | den Harink, T.; Hoek, A.; Groen, H.; Roseboom, T. J.; van Deutekom, A. | 2022 | Which factors play a role in the decision of mothers to participate in child follow-up examinations after participation in an RCT?: a semi-quantitative study | A |
| 171 | Deng, C. L.; Pearce, A. J.; Mentiplay, B. F.; Middleton, K. J.; Clarke, A. C. | 2022 | An isometric neck strengthening program does not improve neck strength in elite women's football-code athletes: A randomised controlled trial | A |
| 172 | Eliasson, K.; Fjellman-Wiklund, A.; Dahlgren, G.; Hellman, T.; Svartengren, M.; Nyman, T.; Lewis, C. | 2022 | Ergonomists' experiences of executing occupational health surveillance for workers exposed to hand-intensive work: a qualitative exploration | A |
| 173 | Endo, K.; Stanyon, D.; Yamasaki, S.; Nakanishi, M.; Niimura, J.; Kanata, S.; Fujikawa, S.; Morimoto, Y.; Hosozawa, M.; Baba, K.; Oikawa, N.; Nakajima, N.; Suzuki, K.; Miyashita, M.; Ando, S.; Hiraiwa-Hasegawa, M.; Kasai, K.; Nishida, A. | 2022 | Self-Reported Maternal Parenting Stress From 9 m Is Longitudinally Associated With Child ADHD Symptoms at Age 12: Findings From a Population-Based Birth Cohort Study | A |
| 174 | Enomoto, M.; Fukami, A.; Morikawa, N.; Yamamoto, M.; Sato, H.; Adachi, H.; Fukumoto, Y. | 2022 | Association of cognitive function with oxytocin as a social hormone in a community dwelling Japanese women; UKU study | A |
| 175 | Eun, Y.; Han, K.; Lee, S. W.; Kim, K.; Kang, S.; Lee, S.; Cha, H. S.; Koh, E. M.; Kim, H.; Lee, J. | 2022 | Increased risk of incident gout in young men with metabolic syndrome: A nationwide population-based cohort study of 3.5 million men | A |

| | | | | |
|---|---|---|---|---|
| 176 | Fain, R. S.; Hayat, S. A.; Luben, R.; Abdul Pari, A. A.; Yip, J. L. Y. | 2022 | Effects of social participation and physical activity on all-cause mortality among older adults in Norfolk, England: an investigation of the EPIC-Norfolk study | A |
| 177 | Fedorowicz, S.; Riley, V.; Cowap, L.; Ellis, N. J.; Chambers, R.; Grogan, S.; Crone, D.; Cottrell, E.; Clark-Carter, D.; Roberts, L.; Gidlow, C. J. | 2022 | Using social media for patient and public involvement and engagement in health research: The process and impact of a closed Facebook group | A |
| 178 | Fedorowicz, Sophia; Riley, Victoria; Cowap, Lisa; Ellis, Naomi J.; Chambers, Ruth; Grogan, Sarah; Crone, Diane; Cottrell, Elizabeth; ClarkCarter, David; Roberts, Lesley; Gidlow, Christopher J. | 2022 | Using social media for patient and public involvement and engagement in health research: The process and impact of a closed facebook group | A |
| 179 | Fenemore, J.; Boerckel, W.; Rigney, M.; McNamara, A.; Gaspar, B.; Mayans, J.; Hennink, M.; Fox, J.; Pretorius, L.; Daniels, M.; Winstone, S.; Thakrar, R. | 2022 | P2.08-05 Lung Cancer Patients Willingness to Attend a Screening Appointment or Lung Health Check: Insights from a Global Patient Experience Survey | A |
| 180 | Fraser, A.; Neil, R. M.; Bartholomew, K. | 2022 | EP01.03-001 Mori Perspectives on a Potential Lung Cancer Screening Programme Aotearoa NZ: An Indigenous People's Perspective | A |
| 181 | Gao, X. X.; Wang, L. M.; Zhang, X.; Zhao, Z. P.; Li, C.; Huang, Z. J.; Liu, C. Y.; Yu, N.; Zhang, Y. S.; Deng, X. Q.; Zhang, M. | 2022 | [Awareness and influencing factors on weight and waist circumference among adult Chinese residents in 2018] | A |
| 182 | Garca-Vigara, A.; Cano, A.; Fernndez-Garrido, J.; Carbonell-Asns, J. A.; Tarn, J. J.; Snchez-Snchez, M. L. | 2022 | Non-use of information and communication technology as a predictor of frailty in postmenopausal midlife and older women | A |
| 183 | Ghoshal, A.; Bradley, P.; Whales, A.; Alonso, A.; Crosbie, P.; Booton, R.; Balata, H. | 2022 | EP01.05-002 Role of the Lung Cancer Screening MDT in the Manchester Lung Health Check Programme | A |
| 184 | Greenhalgh, N.; Akinola, R.; Garcha, S. | 2022 | Impact of ethnicity on clozapine prescribing and monitoring in community mental health teams | A |
| 185 | Hieu, H. T.; S, B. T. | 2022 | Kidney Transplant Recipients with JC Virus Infection Have Decreased Function of the Transplanted Kidney | A |
| 186 | Huang, Y.; Li, Z.; Yang, K.; Zhang, L.; Wei, C.; Yang, P.; Xu, W. | 2022 | The association of uric acid with the development of thyroid nodules: a retrospective cohort study | A |
| 187 | Huang, Z.; Lin, Q.; Sun, T.; Xu, Y.; Yue, X.; Jia, J.; Zhang, H.; Li, X.; Zhang, G.; Yi, W.; Zheng, C.; Tao, Z. | 2022 | Value of urinary C-terminal agrin fragment in monitoring the progression of early kidney injury in type 2 diabetic patients | A |
| 188 | Hunter, B.; Chen, M.; Ratnakumar, P.; Alemu, E.; Logan, A.; Linton-Reid, K.; Tong, D.; Senthivel, N.; Bhamani, A.; Bloch, S.; Kemp, S. V.; Boddy, L.; Jain, S.; Gareeboo, S.; Rawal, B.; Doran, S.; Navani, N.; Nair, A.; Bunce, C.; Kaye, S.; Blackledge, M.; Aboagye, E. O.; Devaraj, A.; Lee, R. W. | 2022 | A radiomics-based decision support tool improves lung cancer diagnosis in combination with the Herder score in large lung nodules | A |
| 189 | Iguchi, S.; Inoue-Hirakawa, T.; Nojima, I.; Noguchi, T.; Sugiura, H. | 2022 | Relationships between stress urinary incontinence and trunk muscle mass or spinal alignment in older women | A |

| | | | | |
|---|---|---|---|---|
| 190 | Ito, M.; Sugiyama, A.; Mino, M.; Kodama, M.; Nagaoki, Y.; Abe, K.; Imada, H.; Ouoba, S.; Bunthen, E.; Ko, K.; Akita, T.; Harakawa, T.; Sako, T.; Chayama, K.; Tanaka, J. | 2022 | Prevalence of Helicobacter pylori infection in the general population evaluated by a resident-register-based epidemiological study | A |
| 191 | Iwasaki, K.; Takeshima, T.; Tateyama, M.; Ha, C.; Yamamoto, Y.; Takeda, J. | 2022 | EPH174 Utilization of Intravenous Iron Preparations for Iron Deficiency Anemia: A Longitudinal Study Using a Japanese Health Insurance Claims Database With Annual Health Check-Ups in 2018-2021 | A |
| 192 | Jallow, M.; Black, G.; van Os, S.; Baldwin, D. R.; Brain, K. E.; Donnelly, M.; Janes, S. M.; Kurtidu, C.; McCutchan, G.; Robb, K. A.; Ruparel, M.; Quaife, S. L. | 2022 | Acceptability of a standalone written leaflet for the National Health Service for England Targeted Lung Health Check Programme: A concurrent, think-aloud study | A |
| 193 | Jallow, Mbasan; Black, Georgia; Os, Sandra; Baldwin, David R.; Brain, Kate E.; Donnelly, Michael; Janes, Samuel M.; Kurtidu, Clara; McCutchan, Grace; Robb, Kathryn A.; Ruparel, Mamta; Quaife, Samantha L. | 2022 | Acceptability of a standalone written leaflet for the national health service for england targeted lung health check programme: A concurrent, thinkaloud study | A |
| 194 | Joseph, Maya Ellampally | 2022 | The impact of social determinants of health on the diagnosis of type 2 diabetes mellitus among Asian Indians in New Jersey | A |
| 195 | Julia, D.; Berit, H.; Karoline, O.; Andrea, B.; Katharina, B.; Christoph, M. | 2022 | Prevalence of hearing loss and hearing aid supply in a large German cohort | A |
| 196 | Katsampouris, E.; Kotti, T.; Ruparel, M.; McEwen, A.; Dickson, J.; Duffy, S.; Waller, J.; Janes, S.; Quaife, S. | 2022 | Rates of acceptance between opt-out vs opt-in smoking cessation referral strategies among current smokers attending a lung health check offering lung cancer screening | A |
| 197 | Kawaguchi, K.; Yokoyama, M.; Ide, K.; Kondo, K. | 2022 | Associations between group exercise and exercise adherence among older community-dwelling adults who attend a community sports club: Resol no Mori Wellness Age Club Longitudinal Study | A |
| 198 | Kawamura, K.; Doi, T.; Kano, K.; Matsui, M.; Hattori, Y.; Onishi, F.; Fukata, H.; Miyake, T. | 2022 | Association between smoking habits and dental care utilization and cost using administrative claims database and specific medical check-up data | A |
| 199 | Ke, Y.; Xu, J.; Zhang, X.; Guo, Q.; Zhu, Y. | 2022 | Association Between Serum Follicle-Stimulating Hormone and Sarcopenia and Physical Disability Among Older Chinese Men: Evidence From a Cross-Sectional Study | A |
| 200 | Kerwin, A. L.; Burhans, W. S.; Mann, S.; Tetreault, M.; Nydam, D. V.; Overton, T. R. | 2022 | Transition cow nutrition and management strategies of dairy herds in the northeastern United States: Part I-Herd description and performance characteristics | A |
| 201 | Khatua, C. R.; Singh, S. P. | 2022 | Prevalence of HBsAg positive subjects in Southern Odisha: experience from a resource constrained region | A |
| 202 | Kobayashi, K.; Ishida, Y.; Gunji, M.; Nagase, K.; Yoshimoto-Suzuki, Y.; Hosoya, Y.; Hasegawa, D.; Manabe, A.; Ohde, S.; Ozawa, M. | 2022 | Factors related to employment in childhood cancer survivors in Japan: A preliminary study | A |
| 203 | Komon, W.; Kijmanawat, A.; Chattrakulchai, K.; Sarit-apirak, S.; Silpakit, C.; Manonai, J. | 2022 | Validation of the Thai version of the Female Genital Self-Image Scale (FGSIS) | A |

| | | | | |
|---|---|---|---|---|
| 204 | Kumaratunga, V.; Donaldson, D.; Ioannides, J.; Parker, J.; Niphuis, H.; Shopland, S. | 2022 | Ophthalmic Findings in a Small Group of Related Pygmy Slow Loris (Nycticebus pygmaeus) | A |
| 205 | Labenz, C.; Arslanow, A.; Nguyen-Tat, M.; Nagel, M.; Wrns, M. A.; Reichert, M. C.; Heil, F. J.; Mainz, D.; Zimper, G.; Rmer, B.; Binder, H.; Farin-Glattacker, E.; Fichtner, U.; Graf, E.; Stelzer, D.; Van Ewijk, R.; Ortner, J.; Velthuis, L.; Lammert, F.; Galle, P. R. | 2022 | Structured Early detection of Asymptomatic Liver Cirrhosis: Results of the population-based liver screening program SEAL | A |
| 206 | Lan, Q.; Zhang, Y.; Lin, F.; Meng, Q.; Buys, N. J.; Fan, H.; Sun, J. | 2022 | Association Between Serum Aminotransferases and Risk of New-Onset Cardiometabolic Disease in a Healthy Chinese Population: A Cohort Study | A |
| 207 | Lebrett, M. B.; Crosbie, E. J.; Yorke, J.; Hewitt, K.; Rowlands, A.; Badrick, E.; Gareth Evans, D.; Balata, H.; Booton, R.; Crosbie, P. A. J. | 2022 | Risk perception and disease knowledge in attendees of a community-based lung cancer screening programme | A |
| 208 | Lebrett, M. B.; Crosbie, E. J.; Yorke, J.; Hewitt, K.; Rowlands, A.; Badrick, E.; Evans, D. G.; Balata, H.; Booton, R.; Crosbie, P. A. J. | 2022 | Risk perception and disease knowledge in attendees of a community-based lung cancer screening programme | A |
| 209 | Lee, J. Y.; Kim, S. E.; Park, S. J.; Park, M. I.; Moon, W.; Kim, J. H.; Jung, K. | 2022 | Helicobacter pylori infection and iron deficiency in non-elderly adults participating in a health check-up program | A |
| 210 | Lee, R.; Nair, A.; Graham, C.; Garcia-Gillam, N.; Fitzgerald, D.; Quaife, S.; Sasieni, P.; Janes, S.; Baldwin, D. | 2022 | NHS England's National Targeted Lung Health Check: preliminary findings | A |
| 211 | Leick, C.; Larsen, L. B.; Larrabee Sonderlund, A.; Svensson, N. H.; Sondergaard, J.; Thilsing, T. | 2022 | Non-participation in a targeted prevention program aimed at lifestyle-related diseases: a questionnaire-based assessment of patient-reported reasons | A |
| 212 | Lewis, M. A.; Harshbarger, C.; Bann, C.; Marconi, V. C.; Somboonwit, C.; Piazza, M. D.; Swaminathan, S.; Burrus, O.; Galindo, C.; Borkowf, C. B.; Marks, G.; Karns, S.; Zulkiewicz, B.; Ortiz, A.; Abdallah, I.; Garner, B. R.; Courtenay-Quirk, C. | 2022 | Effectiveness of an Interactive, Highly Tailored "Video Doctor" Intervention to Suppress Viral Load and Retain Patients With HIV in Clinical Care: A Randomized Clinical Trial | A |
| 213 | Lim, J.; Yoon, S. J.; Shin, J. E.; Han, J. H.; Lee, S. M.; Eun, H. S.; Park, M. S.; Park, K. I. | 2022 | Growth Pattern With Morbidities From Birth to 5 Years of Age in Very Low Birth Weight Infants: Comparison of the Korean National Network and National Health Insurance Service | A |
| 214 | Liu, G. L.; Tao, L.; Zhu, Q.; Jiao, X. J.; Yan, L.; Shao, F. M. | 2022 | Association between the metabolic score for insulin resistance (METS-IR) and estimated glomerular filtration rate (eGFR) among health check-up population in Japan: A retrospective cross-sectional study | A |
| 215 | Maruta, M.; Makizako, H.; Ikeda, Y.; Han, G.; Shimokihara, S.; Miyata, H.; Nakamura, A.; Tokuda, K.; Kubozono, T.; Ohishi, M.; Tomori, K.; Akaida, S.; Tabira, T. | 2022 | Characteristics of meaningful activities in community-dwelling Japanese older adults with pre-frailty and frailty | A |
| 216 | Maruta, M.; Shimokihara, S.; Makizako, H.; Ikeda, Y.; Han, G.; Akasaki, Y.; Hidaka, Y.; Kamasaki, T.; Kubozono, T.; Ohishi, M.; Tabira, T. | 2022 | Associations between apathy and comprehensive frailty as assessed by the Kihon Checklist among community-dwelling Japanese older adults | A |

| | | | | |
|---|---|---|---|---|
| 217 | Masenga, S. K.; Pilic, L.; Malumani, M.; Hamooya, B. M. | 2022 | Erythrocyte sodium buffering capacity status correlates with self-reported salt intake in a population from Livingstone, Zambia | A |
| 218 | Matsumoto, Y.; Nadatani, Y.; Otani, K.; Higashimori, A.; Ominami, M.; Fukunaga, S.; Hosomi, S.; Kamata, N.; Kimura, T.; Fukumoto, S.; Tanaka, F.; Taira, K.; Nagami, Y.; Watanabe, T.; Fujiwara, Y. | 2022 | Prevalence and risk factor for chronic diarrhea in participants of a Japanese medical checkup | A |
| 219 | Mbunda, Theodora | 2022 | Count in the young people: HIV vaccine trial participation in Tanzania | A |
| 220 | McDonagh, S. T. J.; Norris, B.; Fordham, A. J.; Greenwood, M. R.; Richards, S. H.; Campbell, J. L.; Clark, C. E. | 2022 | Inter-arm blood pressure difference and cardiovascular risk estimation in primary care: a pilot study | A |
| 221 | McKenzie, Karen; Wigham, Sarah; Bourne, Jane; Rowlands, Gill; Hackett, Simon | 2022 | Exploring the views of uk regional primary care practitioners on the use and role of screening tools for learning disabilities in their services | A |
| 222 | Mighton, C.; Clausen, M.; Sebastian, A.; Muir, S.; Shickh, S.; Baxter, N. N.; Scheer, A.; Glogowski, E.; Schrader, K.; Thorpe, K.; Kim, T.; Lerner-Ellis, J.; Kim, R.; Regier, D.; Bayoumi, A.; Bombard, Y. | 2022 | eP502: How will returning variants of uncertain significance impact healthcare use? A cross-sectional survey | A |
| 223 | Miyake, T.; Matsuura, B.; Furukawa, S.; Ishihara, T.; Yoshida, O.; Miyazaki, M.; Watanebe, K.; Shiomi, A.; Nakaguchi, H.; Yamamoto, Y.; Koizumi, Y.; Tokumoto, Y.; Hirooka, M.; Takeshita, E.; Kumagi, T.; Abe, M.; Ikeda, Y.; Iwata, T.; Hiasa, Y. | 2022 | Fatty liver with metabolic disorder, such as metabolic dysfunction-associated fatty liver disease, indicates high risk for developing diabetes mellitus | A |
| 224 | Mizutani, S.; Takahashi, K.; Matsuura, M. | 2022 | Situation analyses of caregivers knowledge on infant liquid formula in Japan: a cross-sectional questionnaire-based study | A |
| 225 | Montemor, M. S.; Neumann, L. T. V.; Siqueira, A. C. A.; Barros, L. H. C.; Sanches, S. M. | 2022 | Assessing patients perceptions on hormone therapy for breast cancer: Insights from a Brazilian patient group | A |
| 226 | Morishima, R.; Usami, S.; Ando, S.; Kiyono, T.; Morita, M.; Fujikawa, S.; Araki, T.; Kasai, K. | 2022 | Trajectory and course of problematic alcohol use after the Great East Japan Earthquake: Eight-year follow-up of the Higashi-Matsushima cohort study | A |
| 227 | Murray, R.; Brain, K.; Britton, J.; Lewis, S.; Thorley, R.; Baldwin, D.; Quaife, S.; Chalitsios, C.; Alexandris, P.; Crosbie, P.; Copeland, H.; Quinn-Scoggins, H.; McCutchan, G.; Rogerson, S.; Parrott, S.; Wu, Q.; Gabe, R.; Neal, R.; Beeken, R.; Callister, M. | 2022 | PL03.03 Personalised Smoking Cessation Support in a Lung Cancer Screening Programme: The Yorkshire Enhanced Stop Smoking Study (YESS) | A |
| 228 | Nadal, I. P.; Cliffton, C.; Tolani, E.; Achilleos, S.; Winkley, K.; Chamley, M.; Gaughran, F.; Kottegoda, R.; Gallo, F.; Ismail, K. | 2022 | Eliciting the mechanisms of action of care navigators in the management of type 2 diabetes in people with severe mental illness: A qualitative study | A |
| 229 | Nagashima, Y.; Inokuchi, M.; Yasui, Y.; Uchida, K.; Tokumura, M.; Hasegawa, T. | 2022 | Impact of school closure due to the coronavirus disease 2019 pandemic on body mass index in Japanese children: Retrospective longitudinal study | A |
| 230 | Nakase, K.; Shitara, H.; Tajika, T.; Kuboi, T.; Ichinose, T.; Sasaki, T.; Hamano, N.; Endo, F.; Kamiyama, M.; | 2022 | The Relationship Between Dynamic Balance Ability and Shoulder Pain in High School Baseball Pitchers | A |

| | | | | |
|---|---|---|---|---|
| | Miyamoto, R.; Yamamoto, A.; Kobayashi, T.; Takagishi, K.; Chikuda, H. | | | |
| 231 | Nguyen, T. T.; Le, Q. T.; Hoang, D. T. T.; Du Nguyen, H.; Ha, T. M. T.; Nguyen, M. N. B.; Ta, T. T. T.; Tran, N. T.; Trinh, T. H. N.; Doan, K. P. T.; Lam, D. T.; Tran, S. T. T.; Nguyen, T. X.; Le, H. T.; Ha, V. T.; Nguyen, M. H.; Le, B. L. K.; Duong, M. L.; Pham, T. H.; Tran, A. T.; Phan, X. L. T.; Huynh, T. L.; Nguyen, L. P. T.; Vo, T. B.; Le, D. K. N.; Tran, N. N. T.; Tran, Q. N. T.; Van, Y. L. T.; Huynh, B. N. T.; Nguyen, T. P. T.; Dao, T. T.; Nguyen, L. P. T.; Vo, T. G.; Do, T. T. T.; Truong, D. K.; Tang, H. S.; Phan, M. D.; Nguyen, H. N.; Giang, H. | 2022 | Massively parallel sequencing uncovered disease-associated variant spectra of glucose-6-phosphate dehydrogenase deficiency, phenylketonuria and galactosemia in Vietnamese pregnant women | A |
| 232 | Niranjan, V.; Fitzpatrick, P.; Morrogh, R.; O'Hagan, K. | 2022 | Evaluation of community outreach feasibility programmes on improving cancer-related, preventive, health behaviour | A |
| 233 | O'Brien, K.; Agostino, J.; Ciszek, K.; Douglas, K. A. | 2022 | Parents' perceptions of their child's weight among children in their first year of primary school: a mixed-methods analysis of an Australian cross-sectional (complete enumeration) study | A |
| 234 | Ogunsina, K.; Koru-Sengul, T.; Rodriguez, V.; Caban-Martinez, A. J.; Schaefer-Solle, N.; Ahn, S.; Kobetz-Kerman, E. N.; Lee, D. J. | 2022 | Correlates of Positive Thyroid Peroxidase Antibodies among Firefighters: A Cross-Sectional-Study | A |
| 235 | Okamoto, C.; Tsukamoto, O.; Hasegawa, T.; Hitsumoto, T.; Matsuoka, K.; Takashima, S.; Amaki, M.; Kanzaki, H.; Izumi, C.; Ito, S.; Kitakaze, M. | 2022 | Lower B-type natriuretic peptide levels predict left ventricular concentric remodelling and insulin resistance | A |
| 236 | Osuka, Y.; Okubo, Y.; Nofuji, Y.; Sasai, H.; Seino, S.; Maruo, K.; Fujiwara, Y.; Oka, H.; Shinkai, S.; Lord, S. R.; Kim, H. | 2022 | Modifiable intrinsic factors related to occupational falls in older workers | A |
| 237 | Otaki, Y.; Watanabe, T.; Konta, T.; Watanabe, M.; Fujimoto, S.; Sato, Y.; Asahi, K.; Yamagata, K.; Tsuruya, K.; Narita, I.; Kasahara, M.; Shibagaki, Y.; Iseki, K.; Moriyama, T.; Kondo, M.; Watanabe, T. | 2022 | A Body Shape Index and Aortic Disease-Related Mortality in Japanese General Population | A |
| 238 | Pak, K.; Maln, T.; Santavirta, S.; Shin, S.; Nam, H. Y.; De Maeyer, S.; Nummenmaa, L. | 2022 | Brain glucose metabolism and ageing: A 5-year longitudinal study in a large PET cohort | A |
| 239 | Parker, S. M.; Barr, M.; Stocks, N.; Denney-Wilson, E.; Zwar, N.; Karnon, J.; Kabir, A.; Nutbeam, D.; Roseleur, J.; Liaw, S. T.; McNamara, C.; Frank, O.; Tran, A.; Osborne, R.; Lau, A. Y. S.; Harris, M. | 2022 | Preventing chronic disease in overweight and obese patients with low health literacy using eHealth and teamwork in primary healthcare (HeLP-GP): A cluster randomised controlled trial | A |

| | | | | |
|---|---|---|---|---|
| 240 | Parris, W.; Philip, K.; Kaur-Gill, N.; Flannery, D.; Buttery, S.; Bartlett, E.; Devaraj, A.; Addis, J.; Kemp, S.; Chen, M.; Derbyshire, J.; Morris, K.; Laverty, A.; Hopkinson, N. | 2022 | Effect of an immediate, remote smoking cessation intervention vs usual care among participants enrolled in lung health check: QuLIT2 study | A |
| 241 | Patel Dharmik, S.; Chhatriwala Mitul, N.; Sodavadiya Kirankumar, B.; Jainita, P. | 2022 | Establishment of Biological Reference Interval for Liver Function Test in Tertiary Care Hospital in the Rural Area of Anand District | A |
| 242 | Patnaik, A.; Dash, A.; Samal, M. R.; Nanda, S. | 2022 | Health profile of security personnel in a tertiary care hospital in Odisha | A |
| 243 | Pokora, R. M.; Bttner, M.; Schulz, A.; Schuster, A. K.; Merzenich, H.; Teifke, A.; Michal, M.; Lackner, K.; Mnzel, T.; Zeissig, S. R.; Wild, P. S.; Singer, S.; Wollschlger, D. | 2022 | Determinants of mammography screening participationa cross-sectional analysis of the German population-based Gutenberg Health Study (GHS) | A |
| 244 | Pukki, H.; Bettin, J.; Outlaw, A. G.; Hennessy, J.; Brook, K.; Dekker, M.; Doherty, M.; Shaw, S. C. K.; Bervoets, J.; Rudolph, S.; Corneloup, T.; Derwent, K.; Lee, O.; Rojas, Y. G.; Lawson, W.; Gutierrez, M. V.; Petek, K.; Tsiakkirou, M.; Suoninen, A.; Minchin, J.; Dohle, R.; Lipinski, S.; Natri, H.; Reardon, E.; Estrada, G. V.; Platon, O.; Chown, N.; Satsuki, A.; Milton, D.; Walker, N.; Roldan, O.; Herran, B.; Canedo, C. L.; McCowan, S.; Johnson, M.; Turner, E. J.; Lammers, J.; Yoon, W. H. | 2022 | Autistic Perspectives on the Future of Clinical Autism Research | A |
| 245 | Ryu, K. J.; Yi, K. W.; Kim, Y. J.; Shin, J. H.; Hur, J. Y.; Kim, T.; Seo, J. B.; Lee, K. S.; Park, H. | 2022 | Artificial intelligence approaches to the determinants of womens vaginal dryness using general hospital data | A |
| 246 | Saito, J.; Odawara, M.; Takahashi, H.; Fujimori, M.; Yaguchi-Saito, A.; Inoue, M.; Uchitomi, Y.; Shimazu, T. | 2022 | Barriers and facilitative factors in the implementation of workplace health promotion activities in small and medium-sized enterprises: a qualitative study | A |
| 247 | Sakurada, K.; Konta, T.; Murakami, N.; Kosugi, N.; Saito, T.; Watanabe, M.; Ishizawa, K.; Ueno, Y.; Kayama, T. | 2022 | Association between lack of sexual interest and all-cause mortality in a Japanese general population: The Yamagata prospective observational study | A |
| 248 | Sato, S.; Kawai, H.; Sato, S.; Iwasaki, H.; Omori, M.; Kita, Y.; Ikeda, Y.; Awatsu, T.; Murata, A.; Taniguchi, G.; Shimada, Y.; Genda, T. | 2022 | Hypertension and diabetes mellitus are associated with high FIB-4 index in a health checkup examination cohort without known liver disease | A |
| 249 | Sato, Y.; Takahashi, M.; Ochiai, Y.; Matsuo, T.; Sasaki, T.; Fukasawa, K.; Araki, T.; Tsuchiya, M.; Jniosh Cohort Study | 2022 | Study profile: protocol outline and study perspectives of the cohort by the National Institute of Occupational Safety and Health, Japan (JNIOSH cohort) | A |
| 250 | Schott, E.; Schaller, K.; Mons, U.; Oudraogo, N. | 2022 | [Approaches to increase HPV vaccination rate in Germany - challenges and opportunities. A qualitative study] | A |
| 251 | Seidel-Jacobs, E.; Kohl, F.; Tamayo, M.; Rosenbauer, J.; Schulze, M. B.; Kuss, O.; Rathmann, W. | 2022 | Impact of applying a diabetes risk score in primary care on change in physical activity: a pragmatic cluster randomised trial | A |
| 252 | Seino, S.; Kitamura, A.; Abe, T.; Taniguchi, Y.; Murayama, H.; Amano, H.; Nishi, M.; Nofuji, Y.; Yokoyama, Y.; Narita, M.; Shinkai, S.; Fujiwara, Y. | 2022 | Doseresponse relationships of sarcopenia parameters with incident disability and mortality in older Japanese adults | A |

| | | | | |
|---|---|---|---|---|
| 253 | Shah, S.; Taylor, J.; Bradbury-Jones, C. | 2022 | Barriers and enablers to participating in regular screening programmes for women with cerebral palsy: A qualitative life course study | A |
| 254 | Shang, X. W.; Zhang, X. L.; Huang, Y.; Zhu, Z. T.; Zhang, X. Y.; Liu, S. M.; Liu, J. H.; Tang, S. L.; Wang, W.; Yu, H. H.; Ge, Z. Y.; He, M. G. | 2022 | Temporal trajectories of important diseases in the life course and premature mortality in the UK Biobank | A |
| 255 | Simbolon, L. H.; Purba, R. H. P. | 2022 | Utilization of community-based health centers (Puskesmas) to improve accessibility of health services for liver patients | A |
| 256 | Sivagnanam, T. S.; Venkataraman, J.; Raju, S.; Yugandar, B.; Karthikeyan, V. | 2022 | Normatve Fibroscan Metrics of Liver Stiffness and Fat Attenuation in South Indian Population | A |
| 257 | Smith, C.; Grundy, S. | 2022 | Salford Targeted Lung Health Checks: a retrospective analysis of differences between responders and non-responders | A |
| 258 | Somerset, S.; Jones, W.; Evans, C.; Cirelli, C.; Mbang, D.; Blake, H. | 2022 | Opt-in HIV testing in construction workplaces: an exploration of its suitability, using the socioecological framework | A |
| 259 | Sripongpun, P.; Sono, S.; Sae-Chan, J.; Keawdech, A.; Chamroonkul, N. | 2022 | HBV seroprevalence and liver fibrosis status among population born before national immunization in thailand: findings from a health check-up program | A |
| 260 | Sugiyama, T.; Yamada, Y.; Ito, Y.; Mineo, R.; Iwamoto, R.; Tamba, S.; Fujimoto, T.; Yamamoto, K.; Matsuzawa, Y. | 2022 | Increase in glycemic set point, alongside a decrease in waist circumference, in the non-diabetic population during the Japanese National Intervention Program for metabolic syndrome: A single-center, large-scale, matched-pair analysis | A |
| 261 | Takayama, A.; Takeshima, T.; Yamazaki, H.; Kamitani, T.; Shimizu, S.; Fukuhara, S.; Yamamoto, Y. | 2022 | Resting respiration rate predicts all-cause mortality in older outpatients | A |
| 262 | Tamiru, S.; Bidira, K.; Moges, T.; Dugasa, M.; Amsalu, B.; Gezimu, W. | 2022 | Food safety practice and its associated factors among food handlers in food establishments of Mettu and Bedelle towns, Southwest Ethiopia, 2022 | A |
| 263 | Tang, L.; Chen, D.; Shao, J.; Zhang, H.; Wu, J.; Ye, Z. | 2022 | Design and development of a Wechat applet for intelligent health management of metabolic syndrome | A |
| 264 | Tangjittipokin, W.; Srisawat, L.; Teerawattanapong, N.; Narkdontri, T.; Tangjittipokin, W.; Plengvidhya, N.; Srisawat, L.; Teerawattanapong, N.; Narkdontri, T.; Homsanit, M.; Plengvidhya, N. | 2022 | Prevalence and Characteristics of Prediabetes and Metabolic Syndrome in Seemingly Healthy Persons at a Health Check-Up Clinic | A |
| 265 | Thomas, F.; Pannier, B.; Meghiref, K.; Galtier, D.; Lamande, J. P.; Raison, J.; Danchin, N. | 2022 | Adapted educational health program among deprived subjects with prediabetes | A |
| 266 | Thompson, F.; Harriss, L. R.; Russell, S.; Taylor, S.; Cysique, L. A.; Strivens, E.; Maruff, P.; McDermott, R. | 2022 | Using health check data to investigate cognitive function in Aboriginal and Torres Strait Islanders living with diabetes in the Torres Strait, Australia | A |
| 267 | Tisi, S.; Dickson, J. L.; Horst, C.; Quaife, S. L.; Hall, H.; Verghese, P.; Gyertson, K.; Bowyer, V.; Levermore, C.; Mullin, A. M.; Teague, J.; Farrelly, L.; Nair, A.; Devaraj, A.; Hackshaw, A.; Hurst, J. R.; Janes, S. M. | 2022 | Detection of COPD in the SUMMIT Study lung cancer screening cohort using symptoms and spirometry | A |

| | | | | |
|---|---|---|---|---|
| 268 | Tisi, S.; Dickson, J. L.; Horst, C.; Quaife, S. L.; Hall, H.; Verghese, P.; Gyertson, K.; Bowyer, V.; Levermore, C.; Mullin, A. M.; Teague, J.; Farrelly, L.; Nair, A.; Devaraj, A.; Hackshaw, A.; Hurst, J. R.; Janes, S. M.; Summit Consortium | 2022 | Detection of COPD in the SUMMIT Study lung cancer screening cohort using symptoms and spirometry | A |
| 269 | Ueyama, J.; Ito, Y.; Hamada, R.; Oya, N.; Kato, S.; Matsuki, T.; Tamada, H.; Kaneko, K.; Saitoh, S.; Sugiura-Ogasawara, M.; Ebara, T.; Kamijima, M. | 2022 | Simultaneous quantification of pyrethroid metabolites in urine of non-toilet-trained children in Japan | A |
| 270 | Usher-Smith, J. A.; Godoy, A.; Burge, S. W.; Burbidge, S.; Cartledge, J.; Crosbie, P. A. J.; Eckert, C.; Farquhar, F.; Hammond, D.; Hancock, N.; Iball, G. R.; Kimuli, M.; Masson, G.; Neal, R. D.; Rogerson, S.; Rossi, S. H.; Sala, E.; Smith, A.; Sharp, S. J.; Simmonds, I.; Wallace, T.; Ward, M.; Callister, M. E. J.; Stewart, G. D. | 2022 | The Yorkshire Kidney Screening Trial (YKST): protocol for a feasibility study of adding non-contrast abdominal CT scanning to screen for kidney cancer and other abdominal pathology within a trial of community-based CT screening for lung cancer | A |
| 271 | Vallejo, M. S.; Blmel, J. E.; Bencosme, A.; Calle, A.; Dextre, M.; Daz, K.; Lpez, M.; Miranda, C.; aez, M.; Ojeda, E.; Rey, C.; Rodrigues, M. A.; Salinas, C.; Tserotas, K.; Prez-Lpez, F. R. | 2022 | Factors affecting climacteric women with SARS-CoV-2 infection: A multinational Latin America study (REDLINC XI) | A |
| 272 | Verma, M.; Kosambiya, J. K.; Divakar, B. | 2022 | Forecasting HIV positivity through identification of predictors amongst high-risk women: A cohort study | A |
| 273 | Wada, A.; Makizako, H.; Nakai, Y.; Tomioka, K.; Taniguchi, Y.; Sato, N.; Kiuchi, Y.; Kiyama, R.; Kubozono, T.; Takenaka, T.; Ohishi, M. | 2022 | Association between cognitive frailty and higher-level competence among community-dwelling older adults | A |
| 274 | Waller, D.; Robards, F.; Schneider, C. H.; Sanci, L.; Steinbeck, K.; Gibson, S.; Usherwood, T.; Hawke, C.; Jan, S.; Kong, M.; Kang, M. | 2022 | Building evidence into youth health policy: a case study of the Access 3 knowledge translation forum | A |
| 275 | Wang, J.; Sun, Q.; An, Y.; Liu, J.; Leng, S.; Wang, G. | 2022 | The association of remnant cholesterol (RC) and interaction between RC and diabetes on the subsequent risk of hypertension | A |
| 276 | Watson, J.; Green, M. A.; Giebel, C.; Darlington-Pollock, F.; Akpan, A. | 2022 | Social and spatial inequalities in healthcare use among people living with dementia in England (2002-2016) | A |
| 277 | Williams, P. J.; Philip, K. E. J.; Gill, N. K.; Flannery, D.; Buttery, S.; Bartlett, E. C.; Devaraj, A.; Kemp, S. V.; Addis, J.; Derbyshire, J.; Chen, M.; Morris, K.; Laverty, A. A.; Hopkinson, N. S. | 2022 | Immediate, remote smoking cessation intervention in participants undergoing a targeted lung health check: QuLIT2 a randomised controlled trial | A |
| 278 | Xingxing, G.; Limin, W.; Xiao, Z.; Zhenping, Z.; Chun, L.; Zhengjing, H.; Chenyi, L.; Ning, Y.; Yushu, Z.; Xiaoqing, D.; Mei, Z. | 2022 | Awareness and influencing factors on weight and waist circumference among adult Chinese residents in 2018 | A |

| | | | | |
|---|---|---|---|---|
| 279 | Yanagisawa, T.; Suzuki, F.; Tsujiguchi, H.; Hara, A.; Miyagi, S.; Kannon, T.; Suzuki, K.; Shimizu, Y.; Nguyen, T. T. T.; Oku, F.; Sato, K.; Nakamura, M.; Hayashi, K.; Shibata, A.; Konoshita, T.; Kambayashi, Y.; Tsuboi, H.; Tajima, A.; Nakamura, H. | 2022 | Hypertension and Low Body Weight Are Associated with Depressive Symptoms Only in Females: Findings from the Shika Study | A |
| 280 | Yao, S. Y.; Xiao, S. Y.; Jin, X.; Xiong, M.; Peng, J.; Jian, L. J.; Mei, Y. L.; Huang, Y. H.; Zhou, H. Q.; Xu, T. | 2022 | Effect of a community-based child health counselling intervention on health-seeking behaviours, complementary feeding and nutritional condition among children aged 6-23 months in rural China: A pre- and post-comparison study | A |
| 281 | Yeung, S. S. Y.; Zhu, Z. L. Y.; Kwok, T.; Woo, J. | 2022 | Serum Amino Acids Patterns and 4-Year Sarcopenia Risk in Community-Dwelling Chinese Older Adults | A |
| 282 | Yoo, T. K.; Sung, K. C. | 2022 | Associations of Cardiovascular Mortality with Physical Activity and Concurrent Coronary Artery Calcification | A |
| 283 | Yusuf Mohamud, M. F.; Omar Jeele, M. O. | 2022 | Knowledge, attitude, and practice regarding lifestyle modification among type 2 diabetes patients with cardiovascular disease at a Tertiary Hospital in Somalia | A |
| 284 | Zheng, S.; Nie, Z.; Lv, Z.; Wang, T.; Wei, W.; Fang, D.; Zou, X.; Fu, Y.; Cao, T.; Liang, Z.; Lu, Q.; Huang, H.; Wen, Y.; Huang, S. | 2022 | Associations between plasma metal mixture exposure and risk of hypertension: A cross-sectional study among adults in Shenzhen, China | A |
| 285 | Zhou, X.; Lee, E. W. J.; Wang, X.; Lin, L.; Xuan, Z.; Wu, D.; Lin, H.; Shen, P. | 2022 | Infectious diseases prevention and control using an integrated health big data system in China | A |
| 286 | Amira, B.; Ghada, B.; Dorra, B.; Imen, Y.; Mariem, M.; Hanene, B. S.; Najla, M.; Nizar, L. | 2023 | SLEEP APNEA SYNDROME AND NIGHT WORK IN HEALTH CARE PERSONNEL | A |
| 287 | Anwar, K.; Shikha, ; Sinha, P.; Sinha, A. | 2023 | A Study on Menstrual Hygiene and its Association with Perceived Reproductive Morbidity in Adolescent Girls of Slum of Patna District, Bihar, India | A |
| 288 | Bahreini, M. S.; Sedghi, S.; Badalzadeh, Y.; Motazedian, M. H.; Shirani, M.; Jahromi, S. S.; Teimouri, A.; Agholi, M.; Asgari, Q. | 2023 | Molecular diagnosis of Trichomonas vaginalis in liquid-based Papanicolaou samples in Shiraz, southern Iran | A |
| 289 | Bhamani, A.; Horst, C.; Bojang, F.; Quaife, S. L.; Dickson, J. L.; Tisi, S.; Hall, H.; Verghese, P.; Creamer, A.; Prendecki, R.; McCabe, J.; Gyertson, K.; Bowyer, V.; El-Emir, E.; Cotton, A.; Mehta, S.; Levermore, C.; Mullin, A. M.; Teague, J.; Farrelly, L.; Nair, A.; Devaraj, A.; Hackshaw, A.; Janes, S. M. | 2023 | The SUMMIT Study: Utilising a written Next Steps information booklet to prepare participants for potential lung cancer screening results and follow-up | A |
| 290 | Bradley, C.; Boland, A.; Clarke, L.; Dallinson, N.; Eckert, C.; Ellames, D.; Finn, J.; Gabe, R.; Hancock, N.; Kennedy, M. P. T.; Lindop, J.; Mohamed, A.; Mullen, G.; Murray, R. | 2023 | Diagnosis and treatment outcomes from prebronchodilator spirometry performed alongside lung cancer screening in a Lung Health Check programme | A |

| | | | | |
|---|---|---|---|---|
| | L.; Rogerson, S.; Shinkins, B.; Simmonds, I.; Upperton, S.; Wilkinson, A.; Crosbie, P. A.; Callister, M. E. J. | | | |
| 291 | Campbell, F.; Muszynska, K.; Julian, M.; Woodhead, T.; MacKenzie, P.; Crompton, L.; Powney, B. | 2023 | Improving care and outcomes for teenagers and young adults with diabetes: A new blended training programme for healthcare professionals | A |
| 292 | Choi, E. Y.; Park, J. S.; Min, D.; Ahn, S.; Ahn, J. A. | 2023 | Heart Failure-Smart Life: a randomized controlled trial of a mobile app for self-management in patients with heart failure | A |
| 293 | Dheepa Gayathri, L.; Aarathy, D.; Viveka, M.; Raju, A.; Suganthy, K.; Shenoy, M. T.; Hariharan, A. | 2023 | Study of Dyselectrolytemia in a Tertiary Care Hospital | A |
| 294 | Dickson, J. L.; Hall, H.; Horst, C.; Tisi, S.; Verghese, P.; Mullin, A. M.; Teague, J.; Farrelly, L.; Bowyer, V.; Gyertson, K.; Bojang, F.; Levermore, C.; Anastasiadis, T.; McCabe, J.; Navani, N.; Nair, A.; Devaraj, A.; Hackshaw, A.; Quaife, S. L.; Janes, S. M. | 2023 | Uptake of invitations to a lung health check offering low-dose CT lung cancer screening among an ethnically and socioeconomically diverse population at risk of lung cancer in the UK (SUMMIT): a prospective, longitudinal cohort study | A |
| 295 | Eun, Y.; Han, K.; Lee, S. W.; Kim, K.; Kang, S.; Lee, S.; Cha, H. S.; Koh, E. M.; Kim, H.; Lee, J. | 2023 | Altered Risk of Incident Gout According to Changes in Metabolic Syndrome Status: A Nationwide, Population-Based Cohort Study of 1.29 Million Young Men | A |
| 296 | Fukuda, K.; Matsuzaki, H.; Nomura, Y.; Nakao, T.; Yamaguchi, T.; Hanaoka, S.; Saito, A.; Nagase, T.; Yoshikawa, T. | 2023 | Association of 18F-FDG uptake in semiquantitative PET/CT with emphysema progression in smokers: A retrospective observational study | A |
| 297 | Galindo, C. A.; Freeman, A.; Abdallah, I.; Courtenay-Quirk, C. | 2023 | Positive Health Check intervention tool usage during a feasibility pilot in HIV primary care clinics | A |
| 298 | Goodley, P.; Crosbie, P.; Booton, R.; Balata, H. | 2023 | When to re-invite initially ineligible populations for lung cancer screening? Modelling from Manchester Lung Health Check cohorts | A |
| 299 | Guo, H. J.; Zhou, J. S.; Chen, H.; Cao, X. | 2023 | Prevalence and associated factors of poor sleep quality among Chinese retirees: A multicenter cross-sectional study | A |
| 300 | Hidaka, Y.; Tabira, T.; Maruta, M.; Makizako, H.; Ikeda, Y.; Nakamura, A.; Han, G.; Miyata, H.; Shimokihara, S.; Akasaki, Y.; Kamasaki, T.; Kubozono, T.; Ohishi, M. | 2023 | Relationship between grave visitation and apathy among community-dwelling older adults | A |
| 301 | Hoeck, S.; Tran, T. N. | 2023 | Self-Reported Reasons for Inconsistent Participation in Colorectal Cancer Screening Using FIT in Flanders, Belgium | A |
| 302 | Ikeda, Y.; Kawamura, R.; Takata, Y.; Tabara, Y.; Maruyama, K.; Takakado, M.; Hadate, T.; Ohashi, J.; Saito, I.; Ogawa, Y.; Osawa, H. | 2023 | Resistin GA haplotype at SNP-420/-358 is associated with the latent sarcopenic obesity index in the toon genome study | A |
| 303 | Jackson, J. A.; Liv, P.; Sayed-Noor, A. S.; Punnett, L.; Wahlstrm, J. | 2023 | Risk factors for surgically treated cervical spondylosis in male construction workers: a 20-year prospective study | A |
| 304 | Jiang, X.; Zhang, L.; Gao, Y.; He, C.; Tang, Z.; Sun, J. | 2023 | An Empirical Study on Physical Subhealth Risk Perception: A Physical Examination Data of Tertiary Grade-A Hospitals in Anhui Province, China | A |

| | | | | |
|---|---|---|---|---|
| 305 | Kaneko, H.; Tsuboi, H. | 2023 | Depressive symptoms predict antibody titers after a second dose of the SARS-CoV-2 BNT162b2 vaccine among hospital workers in Japan | A |
| 306 | Kasashi, K.; Sato, A.; Stuart, M.; Hollywood, ; Kawaguchi-Suzuki, M.; Yagishita, K.; Akama, T. | 2023 | The Tokyo 2020 Olympic and Paralympic pharmacy services during the COVID-19 pandemic | A |
| 307 | Khatua, C. R.; Singh, S. P. | 2023 | A study of the prevalence of acute and chronic liver diseases among newly detected HBsAg positive subjects: A single centre experience | A |
| 308 | Kotze, M.; Van Rensburg, S.; Davids, M.; Stroetmann, K.; Daramola, F.; Nyasulu, P.; Von Metzinger, J.; Daramola, O. | 2023 | TRANSLATING POPULATION RISK INTO PERSONAL UTILITY USING A MOBILE PHONE APP FOR APPLICATION OF GENOMIC MEDICINE INTEGRATING SERVICE AND RESEARCH IN THE COVID-19 ERA | A |
| 309 | Ku, P. W.; Steptoe, A.; Lai, Y. J.; Yen, Y. F.; Ahmadi, M.; Inan-Eroglu, E.; Wang, S. F.; Chen, L. J.; Stamatakis, E. | 2023 | Are associations of leisure-time physical activity with mortality attenuated by high levels of chronic ambient fine particulate matter (PM2.5) in older adults? A prospective cohort study | A |
| 310 | Kumar, R.; Amir-Ud-Din, R.; Ahmed, J.; Asim, M.; Rashid, F.; Khan, S. A.; Ali, S.; Pongpanich, S. | 2023 | Correlates of early initiation of breast feeding and prelacteal feeding: A cross-sectional study in Sindh province of Pakistan | A |
| 311 | Kurita, A.; Nakamura, Y. | 2023 | Health check-up results, death, and occurrence of the need for nursing care among Japanese older adults: Analysis using the Kokuho Database system | A |
| 312 | Li, S.; Lu, C.; Kang, L.; Li, Q.; Chen, H.; Zhang, H.; Tang, Z.; Lin, Y.; Bai, M.; Xiong, P. | 2023 | Study on correlations of BDNF, PI3K, AKT and CREB levels with depressive emotion and impulsive behaviors in drug-nave patients with first-episode schizophrenia | A |
| 313 | Lin, H. J.; Wang, J.; Tseng, P. Y.; Fu, L. C.; Lee, Y. C.; Wu, M. S.; Yang, W. S.; Chiu, H. M. | 2023 | Lower-than-normal glycemic levels to achieve optimal reduction of diabetes risk among individuals with prediabetes: A prospective cohort study | A |
| 314 | Magnavita, N. | 2023 | Workplace Health Promotion Embedded in Medical Surveillance: The Italian Way to Total Worker Health Program | A |
| 315 | Michel, M.; Arvis Souar, M.; Dindorf, C.; Danguy, V.; Chevreul, K. | 2023 | Bilans de sant pour la population agricole sous-consommante franaise : valuation pilote du programme des Instants sant de la MSA | A |
| 316 | Moghul, M.; Croft, F.; Kaur, K.; Kinsella, N.; Cahill, D.; James, N. | 2023 | THE MAN VAN: COMMUNITY BASED TARGETED CASE FINDING FOR PROSTATE CANCER | A |
| 317 | Nagasawa, M.; Tajima, T.; Kawagoe, S.; Yamaguchi, N.; Morita, Y.; Yokoe, T.; Ota, T.; Izumi, T.; Ishida, Y.; Chosa, E. | 2023 | Risk factors associated with throwing injuries in young baseball players | A |
| 318 | Ohuchi, K.; Kijima, H.; Saito, H.; Sugimura, Y.; Yoshikawa, T.; Miyakoshi, N. | 2023 | Risk Factors for Glenohumeral Internal Rotation Deficit in Adolescent Athletes: A Comparison of Overhead Sports and Non-overhead Sports | A |
| 319 | Pahk, K.; Joung, C.; Kwon, H. W.; Kim, S. | 2023 | Chronic physical exercise alleviates stress-associated amygdala metabolic activity in obese women: A prospective serial 18F-FDG PET/CT study | A |
| 320 | Saaed, F. M. A.; Ongerth, J. E. | 2023 | Prevalence of Hepatitis B and Hepatitis C in Migrants from Sub-Saharan Africa Before Onward Dispersal Toward Europe | A |

| | | | | |
|---|---|---|---|---|
| 321 | Schmidt, T. L.; Catani, C.; Dumke, L.; Gro, M.; Neldner, S.; Scharpf, F.; Weitkmper, A.; Wilker, S.; Wittmann, J.; Stammnitz, A.; van den Heuvel, R.; Neuner, F. | 2023 | Welcome, how are you doing? - towards a systematic mental health screening and crisis management for newly arriving refugees | A |
| 322 | Shimizu, Y.; Yamanashi, H.; Kitamura, M.; Miyata, J.; Nonaka, F.; Nakamichi, S.; Saito, T.; Nagata, Y.; Maeda, T. | 2023 | Association between periodontitis and chronic kidney disease by functional atherosclerosis status among older Japanese individuals: A cross-sectional study | A |
| 323 | Shrestha, R. K.; Galindo, C. A.; Courtenay-Quirk, C.; Harshbarger, C.; Abdallah, I.; Marconi, V. C.; DallaPiazza, M.; Swaminathan, S.; Somboonwit, C.; Lewis, M. A.; Khavjou, O. A. | 2023 | Cost Analysis of the Positive Health Check Intervention to Suppress HIV Viral Load and Retain Patients in HIV Clinical Care | A |
| 324 | Shrivastava, Y.; Yuwanati, M.; Ganesh, N. | 2023 | Absence of Synergistic Effect of Toluidine Blue and Cytomorphometry in Discriminating Dysplasia in Oral Exfoliative Cytology | A |
| 325 | Sivabalah, K.; Ahuja, G.; Goodley, P.; Hiu, N. C. J.; Ghoshal, A.; Alonso, A.; Sharman, A.; Sinnott, N.; Brockelsby, C.; Craig, C.; Merchant, Z.; Evison, M.; Crosbie, P.; Booton, R.; Balata, H. | 2023 | The impact of real-world lung cancer screening programmes on downstream diagnostic capacity | A |
| 326 | Thies-Lagergren, L.; Johansson, M. | 2023 | Home-based postnatal midwifery care facilitated a smooth succession into motherhood: A Swedish interview study | A |
| 327 | Tsai, J.; Solis, V.; Schick, V. | 2023 | Medical Care Needs of Laundromat Users in San Antonio, Texas: A Potentially Unique Setting for Health Interventions | A |
| 328 | von Sommoggy, J.; Grepmeier, E. M.; Apfelbacher, C.; Brandstetter, S.; Curbach, J. | 2023 | Pediatricians experiences of managing outpatient care during the COVID-19 pandemic: A qualitative study in Germany | A |
| 329 | Ward, A.; Muller, M.; Sharrock, R.; Lou, T.; Tasker, C. | 2023 | Incidental findings arising from targeted lung health checks: a GP practice perspective | A |
| 330 | Weichenberger, M.; Esefeld, K.; Mller, S. | 2023 | Exercise testing in top athletes | A |
| 331 | Williams, P. J.; Philip, K. E. J.; Gill, N. K.; Flannery, D.; Buttery, S.; Bartlett, E. C.; Devaraj, A.; Kemp, S. V.; Addis, J.; Derbyshire, J.; Chen, M.; Morris, K.; Laverty, A. A.; Hopkinson, N. S. | 2023 | Immediate, Remote Smoking Cessation Intervention in Participants Undergoing a Targeted Lung Health Check: Quit Smoking Lung Health Intervention Trial, a Randomized Controlled Trial | A |
| 332 | Yang, H.; Luo, Z. Y.; Lin, F.; Li, L. J.; Lu, M.; Xie, L. X.; Yang, L. Y. | 2023 | Comparison of Urine and Genital Samples for Detecting Human Papillomavirus (HPV) in Clinical Patients | A |
| 333 | Zhan, Y.; Ruan, X.; Huang, D.; Huang, J.; Huang, J.; Chun, T. T.; Ho, B.; Ng, A.; Tsu, J.; Na, R. | 2023 | EFFECTS OF PROSTATE-SPECIFIC ANTIGEN SCREENING ON PROSTATE CANCER INCIDENCE AND MORTALITY: A POPULATION-BASED COHORT STUDY IN CHINA | A |
| 334 | Zhao, D.; Luo, J.; Li, J.; Gao, T.; Fu, P.; Wang, Y.; Zhou, C. | 2023 | Tooth loss, body mass index and cognitive function among middle-aged and older adults in China: Does gender matter? | A |
| 335 | Chingono, R. M. S.; Mackworth-Young, C. R. S.; Ross, D. A.; Tshuma, M.; Chiweshe, T.; Nyamayaro, C.; Sekanevana, | 2021 | Designing Routine Health Checkups for Adolescents in Zimbabwe | B |

| | | | | |
|---|---|---|---|---|
| | C.; Doyle, A. M.; Weiss, H. A.; Kohl, K.; Mangombe, A.; Madzima, B.; McHugh, G.; Ferrand, R. A. | | | |
| 336 | Nikander, K.; Hermanson, E.; Vahlberg, T.; Kaila, M.; Sannisto, T.; Kosola, S. | 2021 | Associations between study questionnaire-assessed need and school doctor-evaluated benefit of routine health checks: an observational study | B |
| 337 | Stich, H. | 2021 | Preventive Check-ups and Routine Vaccinations in Pre-school Children - An Analysis of Mutual Participation Behaviour, Taking into Account Living Conditions | B |
| 338 | Adams, R.; Jordan, R.; Adab, P.; Barrett, T.; Bevan, S.; Cooper, L.; DuRand, I.; Hardy, P.; Heneghan, N.; Jolly, K.; Jowett, S.; Marshall, T.; OHara, M.; Rai, K.; Rickards, H.; Riley, R.; Sadhra, S.; Tearne, S.; Walters, G.; Sapey, E. | 2022 | Enhancing the health of NHS staff: eTHOS protocol for a randomised controlled pilot trial of an employee health screening clinic for NHS staff to reduce absenteeism and presenteeism, compared with usual care | B |
| 339 | Bjerregaard, A. L.; Dalsgaard, E. M.; Bruun, N. H.; Norman, K.; Witte, D. R.; Stovring, H.; Maindal, H. T.; Sandbk, A. | 2022 | Effectiveness of the population-based a check your health preventive programme' conducted in a primary care setting: A pragmatic randomised controlled trial | B |
| 340 | Collatuzzo, G.; Boffetta, P. | 2022 | Application of P4 (Predictive, Preventive, Personalized, Participatory) Approach to Occupational Medicine | B |
| 341 | Garner, B. R.; Burrus, O.; Ortiz, A.; Tueller, S. J.; Peinado, S.; Hedrick, H.; Harshbarger, C.; Galindo, C.; Courtenay-Quirk, C.; Lewis, M. A. | 2022 | A Longitudinal Mixed-Methods Examination of Positive Health Check: Implementation Results From a Type 1 Effectiveness-Implementation Hybrid Trial | B |
| 342 | Gosadi, I. M.; Ayoub, R. A.; Albrahim, H. T.; Alhakami, M. S.; Ageely, E. H.; Alwadani, R. S.; Shayani, H. M.; Shteafi, S. A. | 2022 | An Assessment of the Knowledge and Practices of Adults in Jazan, Saudi Arabia, Concerning Routine Medical Checkups | B |
| 343 | Mason, D.; Taylor, H.; Ingham, B.; Finch, T.; Wilson, C.; Scarlett, C.; Urbanowicz, A.; Nicolaidis, C.; Lennox, N.; Moss, S.; Buckley, C.; Cooper, S. A.; Osborne, M.; Garland, D.; Raymaker, D.; Parr, J. R. | 2022 | Views about primary care health checks for autistic adults: UK survey findings | B |
| 344 | Nikander, K.; Kosola, S.; Vahlberg, T.; Kaila, M.; Hermanson, E. | 2022 | Associating school doctor interventions with the benefit of the health check: an observational study | B |
| 345 | Seilo, N.; Paldanius, S.; Autio, R.; Kunttu, K.; Kaila, M. | 2022 | Health check attendance association with health and study-related factors: a register-based cohort study of Finnish university entrants | B |
| 346 | Seilo, N.; Paldanius, S.; Autio, R.; Koskela, T.; Kunttu, K.; Kaila, M. | 2022 | Association between university students' two-staged health screening and student health care utilisation: register based observational study | B |
| 347 | Weniger, M.; Beesdo-Baum, K.; Roessner, V.; Hense, H.; Knappe, S. | 2022 | Successful prevention of mental health problems? From regular health check-ups to indicative preventive measures for emotional and behavioral problems in preschool and primary school age: a prospective implementation study | B |
| 348 | Wigham, S.; Bourne, J.; McKenzie, K.; Rowlands, G.; Petersen, K.; Hackett, S. | 2022 | Improving access to primary care and annual health checks for people who have a learning disability: a multistakeholder qualitative study | B |

| | | | | |
|---|---|---|---|---|
| 349 | Williams, M.; Thomson, L.; Butcher, E.; Morriss, R.; Khunti, K.; Packham, C. | 2022 | NHS Health Check Programme: a qualitative study of prison experience | B |
| 350 | Chapman, N.; Otahal, P.; Bonner, C.; Nelson, M. R.; Sharman, E. | 2023 | Uptake of the Australian Heart Health Check before and after the COVID-19 pandemic outbreak | B |
| 351 | Chatterjee, A. S.; Kumar, D. | 2023 | Screening for COVID-19 among Workers Attending Medical Examinations Under Occupational Health Service of an Open Cast Iron Ore Mine in Jharkhand: A Cross-Sectional Study | B |
| 352 | Getahun, G. K.; Arega, M.; Keleb, G.; Shiferaw, A.; Bezabih, D. | 2023 | Assessment of routine medical checkups for common noncommunicable diseases and associated factors among healthcare professionals in Addis Ababa, Ethiopia, in 2022 a cross-sectional study | B |
| 353 | Ikegami, K.; Ando, H.; Baba, H.; Sekoguchi, S.; Yoshitake, H.; Sugano, R.; Nozawa, H.; Hasegawa, M.; Ogami, A. | 2023 | Evaluation of self-administered questionnaire items used in regular health check-ups at the workplace by occupational health professionals | B |
| 354 | Lange, A. E.; Mahlo-Nguyen, J.; Pierdant, G.; Allenberg, H.; Heckmann, M.; Ittermann, T. | 2023 | Antenatal Care and Health Behavior of Pregnant Women-An Evaluation of the Survey of Neonates in Pomerania | B |
| 355 | Mujoo, H.; Bowden, N.; Thabrew, H.; Kokaua, J.; Audas, R.; Taylor, B. | 2023 | Identifying neurodevelopmental disabilities from nationalised preschool health check | B |
| 356 | Badenbroek, I. F.; Nielen, M. M. J.; Hollander, M.; Stol, D. M.; de Wit, N. J.; Schellevis, F. G. | 2021 | Characteristics and motives of non-responders in a stepwise cardiometabolic disease prevention program in primary care | C |
| 357 | Chu, C. L.; Lawana, N. | 2021 | Decomposition of income-related inequality in health check-ups services participation among elderly individuals across the 2008 financial crisis in Taiwan | C |
| 358 | Dahl, M.; Sondergaard, S. F.; Diederichsen, A.; Sondergaard, J.; Thilsing, T.; Lindholt, J. S. | 2021 | Involving people with type 2 diabetes in facilitating participation in a cardiovascular screening programme | C |
| 359 | Duddy, C.; Wong, G.; Gadsby, E. W.; Krska, J.; Hibberd, V. | 2021 | NHS Health Check programme: A protocol for a realist review | C |
| 360 | Grauman, ; Hansson, M.; James, S.; Hauber, B.; Veldwijk, J. | 2021 | Communicating Test Results from a General Health Check: Preferences from a Discrete Choice Experiment Survey | C |
| 361 | Hernandez, L. D.; Giezendanner, S.; Fischer, R.; Zeller, A. | 2021 | Expectations about check-up examinations among Swiss residents: A nationwide population-based cross-sectional survey | C |
| 362 | Kim, B.; Lee, Y.; Noh, J. W.; Kim, T. H. | 2021 | Factors Associated with Health Check-up and Cancer Screening Participation among Family Caregivers of Patients with Dementia: A Cross-Sectional Study | C |
| 363 | Lee, H. Y.; Kim, S.; Neese, J.; Lee, M. H. | 2021 | Does health literacy affect the uptake of annual physical check-ups?: Results from the 2017 US health information national trends survey | C |
| 364 | O'Flaherty, M.; Lloyd-Williams, F.; Capewell, S.; Boland, A.; Maden, M.; Collins, B.; Bandosz, P.; Hyseni, L.; Kypridemos, C. | 2021 | Modelling tool to support decision-making in the NHS Health Check programme: workshops, systematic review and co-production with users | C |
| 365 | Park, B. H.; Lee, B. K.; Ahn, J.; Kim, N. S.; Park, J.; Kim, Y. | 2021 | Association of Participation in Health Check-ups with Risk Factors for Cardiovascular Diseases | C |

| | | | | |
|---|---|---|---|---|
| 366 | Robson, J.; Garriga, C.; Coupland, C.; Hippisley-Cox, J. | 2021 | NHS Health Checks: an observational study of equity and outcomes 2009-2017 | C |
| 367 | Starker, A.; Hovener, C.; Rommel, A. | 2021 | Utilization of preventive care among migrants and non-migrants in Germany: results from the representative cross-sectional study 'German health interview and examination survey for adults (DEGS1)' | C |
| 368 | Usher, K.; Bhullar, N.; Sibbritt, D.; Anubha Amarasena, S. S.; Peng, W.; Durkin, J.; Smallwood, R.; Power, T.; Porter, C.; McGowen, D.; Jackson, D. | 2021 | Influence of COVID-19 on the preventive health behaviours of indigenous peoples of Australia residing in New South Wales: A mixed-method study protocol | C |
| 369 | Duddy, C.; Gadsby, E.; Hibberd, V.; Krska, J.; Wong, G. | 2022 | Understanding what happens to attendees after an NHS Health Check: a realist review | C |
| 370 | Grauman, A.; Johansson, J. V.; Falahee, M.; Veldwijk, J. | 2022 | Public perceptions of myocardial infarction: Do illness perceptions predict preferences for health check results | C |
| 371 | Kuwabara, Y.; Fujii, M.; Kinjo, A.; Osaki, Y. | 2022 | Abstaining from annual health check-ups is a predictor of advanced cancer diagnosis: a retrospective cohort study | C |
| 372 | Lakhan, P.; Cooney, A.; Palamuthusingam, D.; Torrens, G.; Spurling, G.; Martinez, A.; Johnson, D. | 2022 | Challenges of conducting kidney health checks among patients at risk of chronic kidney disease and attending an urban Aboriginal and Torres Strait Islander primary healthcare service | C |
| 373 | Lal, S.; Nguyen, T. X. T.; Sulemana, A. S.; Khan, M. S. R.; Kadoya, Y. | 2022 | Does financial literacy influence preventive health check-up behavior in Japan? a cross-sectional study | C |
| 374 | Lei, L.; Tang, Y.; Zhang, Q.; Xiao, M.; Dai, L.; Lu, J.; Lin, X.; Lu, X.; Luo, W.; Pan, J.; Xin, X.; Qiu, S.; Li, Y.; An, S.; Xiu, J. | 2022 | The Association Between the Frequency of Annual Health Checks Participation and the Control of Cardiovascular Risk Factors | C |
| 375 | Molokhia, M.; Ayis, D. S.; Karamanos, A.; L'Esperance, D. V.; Yousif, S.; Durbaba, S.; urin, V.; Ashworth, M.; Harding, S. | 2022 | What factors influence differential uptake of NHS Health Checks, diabetes and hypertension reviews among women in ethnically diverse South London? Cross-sectional analysis of 63,000 primary care records | C |
| 376 | Ogunlayi, F.; Chauhan-Lall, N.; Hughes, D.; Myers, P.; Sitch, A. | 2022 | A cross-sectional study examining the equitability of invitation, uptake and coverage for NHS Health Check | C |
| 377 | Rombouts, M.; Raaijmakers, L. G. M.; Kuunders, T. J. M.; Van Steijn-Martens, R.; de Vuijst, T.; van Donkersgoed, H.; van de Goor, L. A. M. | 2022 | The Acceptance and Use of the e-Health Instrument 'The Personal Health Check' in Four Dutch Municipalities: Lessons Learned | C |
| 378 | Tanner, L.; Kenny, R. P. W.; Still, M.; Ling, J.; Pearson, F.; Thompson, K.; Bhardwaj-Gosling, R. | 2022 | NHS Health Check programme: A rapid review update | C |
| 379 | Zhao, D. T.; Zhou, Z. L.; Shen, C.; Zhai, X. H.; Zhao, Y. X.; Cao, D.; Deng, Q. W.; Liu, G. P.; Lim, J. F. Y. | 2022 | The Effect of Health Check-Ups on Health Among the Elderly in China: Evidence From 2011-2018 Longitudinal Data | C |
| 380 | Andrka, L.; Csenteri, O.; Andrka, P.; Vajer, P. | 2023 | [Health status and cardiovascular risk of Roma and non-Roma population in underprivileged settlements] | C |
| 381 | Ogita, M.; Okura, M.; Katayose, R.; Miyamatsu, N.; Arai, H. | 2023 | Participation in health and frailty check-ups predicted functional outcomes and mortality in older adults in Japan | C |

| | | | | |
|---|---|---|---|---|
| 382 | Balata, H.; Ruparel, M.; O'Dowd, E.; Ledson, M.; Janes, S.; Booton, R.; Baldwin, D.; Crosbie, P. | 2021 | Lung cancer screening - Cumulative results from five UK-based programmes | Duplicate |
| 383 | Balata, H.; Ruparel, M.; O'Dowd, E.; Ledson, M.; Janes, S.; Booton, R.; Baldwin, D.; Crosbie, P. | 2021 | Lung cancer screening-Cumulative results from five UK-based programmes | Duplicate |
| 384 | Bestoso, J.; Stefani, C. V.; Ciancaglini, L.; Bauso, D. | 2021 | Parkinson's disease and covid-19 pandemic: Modifications in motor and non-motor symptoms and in the access to medical and nonmedical consultations and treatments | Duplicate |
| 385 | BorrullGuardeo, Jessica; SebastiLaguarda, Cruz; DonatColomer, Francisco; SnchezMartnez, Vanessa | 2021 | Womens knowledge and attitudes towards cervical cancer prevention: A qualitative study in the Spanish context | Duplicate |
| 386 | Chi, P. C.; Owino, E. A.; Jao, I.; Olewe, F.; Ogutu, B.; Bejon, P.; Kapulu, M.; Kamuya, D.; Marsh, V.; Chmi-Sika Study, Team | 2021 | Understanding the benefits and burdens associated with a malaria human infection study in Kenya: experiences of study volunteers and other stakeholders | Duplicate |
| 387 | de Waard, A. K. M.; Korevaar, J. C.; Hollander, O.; Nielen, M. M. J.; Seifert, B.; Carlsson, A. C.; Lionis, C.; Sondergaard, J.; Schellevis, F. G.; de Wit, N. J.; Spimeu Project Grp | 2021 | Unwillingness to participate in health checks for cardiometabolic diseases: A survey among primary health care patients in five European countries | Duplicate |
| 388 | de Waard, A. M.; Korevaar, J. C.; Hollander, M.; Nielen, M. M. J.; Seifert, B.; Carlsson, A. C.; Lionis, C.; Sndergaard, J.; Schellevis, F. G.; de Wit, N. J. | 2021 | Unwillingness to participate in health checks for cardiometabolic diseases: A survey among primary health care patients in five European countries | Duplicate |
| 389 | Harvie, M.; French, D. P.; Pegington, M.; Cooper, G.; Howell, A.; McDiarmid, S.; Lombardelli, C.; Donnelly, L.; Ruane, H.; Sellers, K.; Barrett, E.; Armitage, C. J.; Evans, D. G. | 2021 | Testing a breast cancer prevention and a multiple disease prevention weight loss programme amongst women within the UK NHS breast screening programme-a randomised feasibility study | Duplicate |
| 390 | HerpertzDahlmann, Beate; Bonin, Eva; Dahmen, Brigitte | 2021 | Can you find the right support for children, adolescents and young adults with anorexia nervosa: Access to ageappropriate care systems in various healthcare systems | Duplicate |
| 391 | Huang, H. L.; Chuang, Y. H.; Lin, T. H.; Lin, C. Q.; Chen, Y. H.; Hung, J. Y.; Chan, T. C. | 2021 | Ambient Cumulative PM2.5 Exposure and the Risk of Lung Cancer Incidence and Mortality: A Retrospective Cohort Study | Duplicate |
| 392 | In, A.; Turgut, A.; Peden, A. E. | 2021 | Epidemiology of Football-Related Sudden Cardiac Death in Turkey | Duplicate |
| 393 | Kobayashi, H.; Joshita, S.; Akahane, Y.; Matsuzaki, K.; Yamada, H.; Aomura, D.; Joshita, N.; Midorikawa, H.; Suyama, K.; Ota, M.; Wakabayashi, S.; Yamashita, Y.; Sugiura, A.; Yamazaki, T.; Misawa, H.; Umemura, T. | 2021 | Protocol: Prospective observational study aiming for micro-elimination of hepatitis C virus in Nagawa town: The Nagawa Project | Duplicate |
| 394 | Kuriyama, N.; Ozaki, E.; Koyama, T.; Matsui, D.; Watanabe, I.; Tomida, S.; Nagamitsu, R.; Hashiguchi, K.; Inaba, M.; Yamada, S.; Horii, M.; Mizuno, S.; Yoneda, Y.; Kurokawa, M.; Kobayashi, D.; Fukuda, S.; Iwasa, K.; Watanabe, Y.; Uehara, R. | 2021 | Evaluation of myostatin as a possible regulator and marker of skeletal muscle-cortical bone interaction in adults | Duplicate |

| | | | | |
|---|---|---|---|---|
| 395 | Leach, A. J.; Morris, P. S.; Coates, H. L.; Nelson, S.; O'Leary, S. J.; Richmond, P. C.; Gunasekera, H.; Harkus, S.; Kong, K. L.; Brennon-Jones, C. G.; Brophy-Williams, S.; Currie, K.; Das, S. K.; Isaacs, D.; Jarosz, K.; Lehmann, D.; Pak, J.; Patel, H.; Perry, C.; Reath, J. S.; Sommer, J.; Torzillo, P. J. | 2021 | Otitis media guidelines for Australian Aboriginal and Torres Strait Islander children: summary of recommendations | Duplicate |
| 396 | Maruta, Michio; Makizako, Hyuma; Ikeda, Yuriko; Miyata, Hironori; Nakamura, Atsushi; Han, Gwanghee; Shimokihara, Suguru; Tokuda, Keiichiro; Kubozono, Takuro; Ohishi, Mitsuru; Tabira, Takayuki | 2021 | Association between apathy and satisfaction with meaningful activities in older adults with mild cognitive impairment: A populationbased crosssectional study | Duplicate |
| 397 | Murayama, H.; Takahashi, Y.; Shimada, S. | 2021 | Effectiveness of an Out-of-Pocket Cost Removal Intervention on Health Check Attendance in Japan | Duplicate |
| 398 | OFlaherty, M.; Lloyd-Williams, F.; Capewell, S.; Boland, A.; Maden, M.; Collins, B.; Bandosz, P.; Hyseni, L.; Kypridemos, C. | 2021 | Modelling tool to support decision-making in the NHS Health Check programme: Workshops, systematic review and co-production with users | Duplicate |
| 399 | Storholm, E. D.; Ober, A. J.; Mizel, M. L.; Matthews, L.; Sargent, M.; Todd, I.; Zajdman, D.; Green, H. | 2021 | PRIMARY CARE PROVIDERS' KNOWLEDGE, ATTITUDES, AND BELIEFS ABOUT HIV PRE-EXPOSURE PROPHYLAXIS (PREP): INFORMING NETWORK-BASED INTERVENTIONS | Duplicate |
| 400 | Taylan, S.; Ozkan, I.; Adibelli, D. | 2021 | Breast Cancer Perception Scale: Psychometric Development Study | Duplicate |
| 401 | Tsou, M. T.; Chang, Y. C.; Hsu, C. P.; Kuo, Y. C.; Yun, C. H.; Huang, W. H.; Hu, K. C.; Liu, C. Y.; Chen, Y. J.; Sung, K. Z.; Liu, C. C.; Hung, C. L.; Kuo, J. Y.; Chen, T. Y.; Hung, T. C.; Yeh, H. | 2021 | Visceral adiposity index outperforms conventional anthropometric assessments as predictor of diabetes mellitus in elderly Chinese: a population-based study | Duplicate |
| 402 | Usher, K.; Bhullar, N.; Sibbritt, D.; Amarasena, S. S. A.; Peng, W. B.; Durkin, J.; Smallwood, R.; Power, T.; Porter, C.; McGowen, D.; Jackson, D. | 2021 | Influence of COVID-19 on the preventive health behaviours of indigenous peoples of Australia residing in New South Wales: a mixed-method study protocol | Duplicate |
| 403 | Usher-Smith, J. A.; Hggstrm, C.; Wennberg, P.; Lindvall, K.; Strelitz, J.; Sharp, S. J.; Griffin, S. J. | 2021 | Impact of achievement and change in achievement of lifestyle recommendations in middle-age on risk of the most common potentially preventable cancers | Duplicate |
| 404 | Wei, L. M.; Cheng, X.; Luo, Y. L.; Yang, R. X.; Lei, Z. T.; Jiang, H. L.; Chen, L. | 2021 | Lean non-alcoholic fatty liver disease and risk of incident diabetes in a euglycaemic population undergoing health check-ups: A cohort study | Duplicate |
| 405 | Adams, R.; Jordan, R.; Adab, P.; Barrett, T.; Bevan, S.; Cooper, L.; DuRand, I.; Hardy, P.; Heneghan, N.; Jolly, K.; Jowett, S.; Marshall, T.; O'Hara, M.; Rai, K.; Rickards, H.; Riley, R.; Sadhra, S.; Tearne, S.; Walters, G.; Sapey, E. | 2022 | Enhancing the health of NHS staff: eTHOS - protocol for a randomised controlled pilot trial of an employee health screening clinic for NHS staff to reduce absenteeism and presenteeism, compared with usual care | Duplicate |
| 406 | Bjerregaard, A. L.; Dalsgaard, E. M.; Bruun, N. H.; Norman, K.; Witte, D. R.; Stovring, H.; Maindal, H. T.; Sandbk, A. | 2022 | Effectiveness of the population-based 'check your health preventive programme' conducted in a primary care setting: a pragmatic randomised controlled trial | Duplicate |

| | | | | |
|---|---|---|---|---|
| 407 | Cavers, Debbie; Nelson, Mia; Rostron, Jasmin; Robb, Kathryn A.; Brown, Lynsey R.; Campbell, Christine; Akram, Ahsan R.; Dickie, Graeme; Mackean, Melanie; Beek, Edwin J. R.; Sullivan, Frank; Steele, Robert J.; Neilson, Aileen R.; Weller, David | 2022 | Optimizing the implementation of lung cancer screening in scotland: Focus group participant perspectives in the lungscot study | Duplicate |
| 408 | Duddy, C.; Gadsby, E.; Hibberd, V.; Krska, J.; Wong, G. F. | 2022 | Understanding what happens to attendees after an NHS Health Check: a realist review | Duplicate |
| 409 | Fain, R. S.; Hayat, S. A.; Luben, R.; Pari, A. A. A.; Yip, J. L. Y. | 2022 | Effects of social participation and physical activity on all-cause mortality among older adults in Norfolk, England: an investigation of the EPIC-Norfolk study | Duplicate |
| 410 | Gosadi, I. M.; Ayoub, R. A.; Albrahim, H.; Alhakami, M. S.; Ageely, E. H.; Alwadani, R. S.; Shayani, H. M.; Shteafi, S. A. | 2022 | An Assessment of the Knowledge and Practices of Adults in Jazan, Saudi Arabia, Concerning Routine Medical Checkups | Duplicate |
| 411 | Grauman, ; Viberg Johansson, J.; Falahee, M.; Veldwijk, J. | 2022 | Public perceptions of myocardial infarction: Do illness perceptions predict preferences for health check results | Duplicate |
| 412 | Ito, M.; Sugiyama, A.; Mino, M.; Kodama, M.; Nagaoki, Y.; Abe, K.; Imada, H.; Ouoba, S.; E, B.; Ko, K.; Akita, T.; Harakawa, T.; Sako, T.; Chayama, K.; Tanaka, J. | 2022 | Prevalence of Helicobacter pylori infection in the general population evaluated by a resident-register-based epidemiological study | Duplicate |
| 413 | Ke, Y. Y.; Xu, J.; Zhang, X. Y.; Guo, Q. H.; Zhu, Y. X. | 2022 | Association Between Serum Follicle-Stimulating Hormone and Sarcopenia and Physical Disability Among Older Chinese Men: Evidence From a Cross-Sectional Study | Duplicate |
| 414 | Leick, C.; Larsen, L. B.; Sonderlund, A. L.; Svensson, N. H.; Sondergaard, J.; Thilsing, T. | 2022 | Non-participation in a targeted prevention program aimed at lifestyle-related diseases: a questionnaire-based assessment of patient-reported reasons | Duplicate |
| 415 | Liu, G.; Tao, L.; Zhu, Q.; Jiao, X.; Yan, L.; Shao, F. | 2022 | Association between the metabolic score for insulin resistance (METS-IR) and estimated glomerular filtration rate (eGFR) among health check-up population in Japan: A retrospective cross-sectional study | Duplicate |
| 416 | Maruta, Michio; Shimokihara, Suguru; Makizako, Hyuma; Ikeda, Yuriko; Han, Gwanghee; Akasaki, Yoshihiko; Hidaka, Yuma; Kamasaki, Taishiro; Kubozono, Takuro; Ohishi, Mitsuru; Tabira, Takayuki | 2022 | Associations between apathy and comprehensive frailty as assessed by the kihon checklist among communitydwelling japanese older adults | Duplicate |
| 417 | Mizutani, S.; Takahashi, K.; Matsuura, M. | 2022 | Situation analyses of caregivers' knowledge on infant liquid formula in Japan: a cross-sectional questionnaire-based study | Duplicate |
| 418 | Molokhia, M.; Ayis, S.; Karamanos, A.; L'Esperance, V.; Yousif, S.; Durbaba, S.; Curcin, V.; Ashworth, M.; Harding, S. | 2022 | What factors influence differential uptake of NHS Health Checks, diabetes and hypertension reviews among women in ethnically diverse South London? Cross-sectional analysis of 63,000 primary care records | Duplicate |
| 419 | Morishima, Ryo; Usami, Satoshi; Ando, Shuntaro; Kiyono, Tomoki; Morita, Masaya; Fujikawa, Shinya; Araki, Tsuyoshi; Kasai, Kiyoto | 2022 | Trajectory and course of problematic alcohol use after the great East Japan earthquake: Eightyear followup of the HigashiMatsushima cohort study | Duplicate |

| | | | | |
|---|---|---|---|---|
| 420 | Nakase, K.; Shitara, H.; Tajika, T.; Kuboi, T.; Ichinose, T.; Sasaki, T.; Hamano, N. | 2022 | The Relationship Between Dynamic Balance Ability and Shoulder Pain in High School Baseball Pitchers | Duplicate |
| 421 | Nguyen, T. T.; Le, Q. T.; Hoang, D. T. T.; Nguyen, H. D.; Ha, T. M. T.; Nguyen, M. N. B.; Ta, T. T. T.; Tran, N. T.; Trinh, T. H. N.; Doan, K. P. T.; Lam, D. T.; Tran, S. T. T.; Nguyen, T. X.; Le, H. T.; Ha, V. T.; Nguyen, M. H.; Le, B. L. K.; Duong, M. L.; Ha Pham, T.; Tran, A. T.; Phan, X. L. T.; Huynh, T. L.; Nguyen, L. P. T.; Vo, T. B.; Le, D. K. N.; Tran, N. N. T.; Tran, Q. N. T.; Van, Y. L. T.; Huynh, B. N. T.; Dao, T. T.; Nguyen, L. P. T.; Vo, T. G.; Do, T. T. T.; Truong, D. K.; Tang, H. S.; Phan, M. D.; Nguyen, H. N.; Giang, H. | 2022 | Massively parallel sequencing uncovered disease-associated variant spectra of glucose-6-phosphate dehydrogenase deficiency, phenylketonuria and galactosemia in Vietnamese pregnant women | Duplicate |
| 422 | OBrien, K.; Agostino, J.; Ciszek, K.; Douglas, K. A. | 2022 | Parents perceptions of their childs weight among children in their first year of primary school: a mixed-methods analysis of an Australian cross-sectional (complete enumeration) study | Duplicate |
| 423 | Pokora, R. M.; Buttner, M.; Schulz, A.; Schuster, A. K.; Merzenich, H.; Teifke, A.; Michal, M.; Lackner, K.; Munzel, T.; Zeissig, S. R.; Wild, P. S.; Singer, S.; Wollschlager, D. | 2022 | Determinants of mammography screening participation-a cross-sectional analysis of the German population-based Gutenberg Health Study (GHS) | Duplicate |
| 424 | Ryu, K. J.; Yi, K. W.; Kim, Y. J.; Shin, J. H.; Hur, J. Y.; Kim, T.; Seo, J. B.; Lee, K. S.; Park, H. | 2022 | Artificial intelligence approaches to the determinants of women's vaginal dryness using general hospital data | Duplicate |
| 425 | Schott, E.; Schaller, K.; Mons, U.; Ouedraogo, N. | 2022 | Approaches to increase HPV vaccination rate in Germany - challenges and opportunities. A qualitative study | Duplicate |
| 426 | Seino, S.; Kitamura, A.; Abe, T.; Taniguchi, Y.; Murayama, H.; Amano, H.; Nishi, M.; Nofuji, Y.; Yokoyama, Y.; Narita, M.; Shinkai, S.; Fujiwara, Y. | 2022 | Dose-response relationships of sarcopenia parameters with incident disability and mortality in older Japanese adults | Duplicate |
| 427 | Wang, M. J.; Lo, Y. T. | 2022 | Strategies for Improving the Utilization of Preventive Care Services: Application of Importance-Performance Gap Analysis Method | Duplicate |
| 428 | Watson, James; Green, Mark A.; Giebel, Clarissa; Darlington-Pollock, Frances; Akpan, Asangaedem | 2022 | Social and spatial inequalities in healthcare use among people living with dementia in england (20022016) | Duplicate |
| 429 | Zhao, D.; Zhou, Z.; Shen, C.; Zhai, X.; Zhao, Y.; Cao, D.; Deng, Q.; Liu, G.; Lim, J. F. Y. | 2022 | The Effect of Health Check-Ups on Health Among the Elderly in China: Evidence From 2011-2018 Longitudinal Data | Duplicate |
| 430 | Bhamani, A.; Horst, C.; Bojang, F.; Quaife, S. L.; Dickson, J. L.; Tisi, S.; Hall, H.; Verghese, P.; Creamer, A.; Prendecki, R.; McCabe, J.; Gyertson, K.; Bowyer, V.; El-Emir, E.; Cotton, A.; Mehta, S.; Levermore, C.; Mullin, A. M.; Teague, J.; Farrelly, L.; Nair, A.; Devaraj, A.; Hackshaw, A.; Janes, S. M. | 2023 | The SUMMIT Study: Utilising a written 'Next Steps' information booklet to prepare participants for potential lung cancer screening results and follow-up | Duplicate |

| | | | | |
|---|---|---|---|---|
| 431 | Bhamani, A.; Horst, C.; Bojang, F.; Quaife, S. L.; Dickson, J. L.; Tisi, S.; Hall, H.; Verghese, P.; Creamer, A.; Prendecki, R.; McCabe, J.; Gyertson, K.; Bowyer, V.; El - Emir, E.; Cotton, A.; Mehta, S.; Levermore, C.; Mullin, A. M.; Teague, J.; Farrelly, L.; Nair, A.; Devaraj, A.; Hackshaw, A.; Janes, S.; Summit Consortium | 2023 | The SUMMIT Study: Utilising a written 'Next Steps' information booklet to prepare participants for potential lung cancer screening results and follow-up | Duplicate |
| 432 | Choi, E. Y.; Park, J. S.; Min, D. L.; Ahn, S.; Ahn, J. A. | 2023 | Heart Failure-Smart Life: a randomized controlled trial of a mobile app for self-management in patients with heart failure | Duplicate |
| 433 | Guo, H.; Zhou, J.; Chen, H.; Cao, X. | 2023 | Prevalence and associated factors of poor sleep quality among Chinese retirees: A multicenter cross-sectional study | Duplicate |
| 434 | Hidaka, Yuma; Tabira, Takayuki; Maruta, Michio; Makizako, Hyuma; Ikeda, Yuriko; Nakamura, Atsushi; Han, Gwanghee; Miyata, Hironori; Shimokihara, Suguru; Akasaki, Yoshihiko; Kamasaki, Taishiro; Kubozono, Takuro; Ohishi, Mitsuru | 2023 | Relationship between grave visitation and apathy among communitydwelling older adults | Duplicate |
| 435 | Ikegami, K.; Ando, H.; Baba, H.; Sekoguchi, S.; Yoshitake, H.; Sugano, R.; Nozawa, H.; Hasegawa, M.; Ogami, A. | 2023 | [Evaluation of self-administered questionnaire items used in regular health check-ups at the workplace by occupational health professionals] | Duplicate |
| 436 | Ku, P. W.; Steptoe, A.; Lai, Y. J.; Yen, Y. F.; Ahmadi, M.; Inan-Eroglu, E.; Wangj, S. F.; Chen, L. J.; Stamatakis, E. | 2023 | Are associations of leisure-time physical activity with mortality attenuated by high levels of chronic ambient fine particulate matter (PM2.5) in older adults? A prospective cohort study | Duplicate |
| 437 | Kurita, A.; Nakamura, Y. | 2023 | [Health check-up results, death, and occurrence of the need for nursing care among Japanese older adults: Analysis using the Kokuho Database system] | Duplicate |
| 438 | Williams, P. J.; Philip, K. E. J.; Gill, N. K.; Flannery, D.; Buttery, S.; Bartlett, E. C.; Devaraj, A.; Kemp, S. V.; Addis, J.; Derbyshire, J.; Chen, M. C. L.; Morris, K.; Laverty, A. A.; Hopkinson, N. S. | 2023 | Immediate, Remote Smoking Cessation Intervention in Participants Undergoing a Targeted Lung Health Check Quit Smoking Lung Health Intervention Trial, a Randomized Controlled Trial | Duplicate |

**Literature search update: June 2023**
**Records assessed at full text level and the excluded reasons**

| # | Author | Year | Title | Excluded with reason |
|---|---|---|---|---|
| 1 | Baeza-Rivera, M. J.; Salinas-Oate, N.; Gmez-Prez, D.; Daz-Loving, R.; Ortiz, M. S. | 2022 | Model to Predict Healthcare Behaviors: Comparison of a Chilean and Mexican Sample | A |
| 2 | Thilsing, T.; Larsen, L. B.; Sonderlund, A. L.; Andreassen, S. S.; Christensen, J. R.; Svensson, N. H.; Dahl, M.; Sondergaard, J. | 2021 | Effects of a Co-Design-Based Invitation Strategy on Participation in a Preventive Health Check Program: Randomized Controlled Trial | B<br>(29 to 59 age) |
| 3 | Al-Hanawi, M. K.; Chirwa, G. C. | 2021 | Economic Analysis of Inequality in Preventive Health Check-Ups Uptake in Saudi Arabia | C |
| 4 | de Waard, A. K. M.; Korevaar, J. C.; Hollander, M.; Nielen, M. M. J.; Seifert, B.; Carlsson, A. C.; Lionis, C.; Sndergaard, J.; Schellevis, F. G.; de Wit, N. J.; Angelaki, A.; Krl, N.; Sonderlund, A.; Wndell, P. | 2021 | Unwillingness to participate in health checks for cardiometabolic diseases: A survey among primary health care patients in five European countries | C |
| 5 | Rombouts, M.; Raaijmakers, L. G. M.; Kuunders, T. J. M.; Van Steijn-Martens, R.; de Vuijst, T.; van Donkersgoed, H.; van de Goor, L. A. M. | 2021 | The acceptance and use of the e-health instrument the personal health check in four dutch municipalities: Lessons learned | C |
| 6 | Butler, D. C.; Agostino, J.; Paige, E.; Korda, R. J.; Douglas, K. A.; Wade, V.; Banks, E. | 2022 | Aboriginal and Torres Strait Islander health checks: sociodemographic characteristics and cardiovascular risk factors | C |
| 7 | Chua, G. N.; Bond, C.; Porteous, T.; Ryan, M. | 2022 | Will the Public Engage with New Pharmacy Roles? Assessing Future Uptake of a Community Pharmacy Health Check Using a Discrete Choice Experiment | C |
| 8 | Wang, M. J.; Lo, Y. T. | 2022 | Strategies for Improving the Utilization of Preventive Care Services: Application of ImportancePerformance Gap Analysis Method | C |
| 9 | Murayama, H.; Takahashi, Y.; Shimada, S. | 2021 | Effectiveness of an outofpocket cost removal intervention on health check attendance in japan | E |
| 10 | Sallis, A.; Gold, N.; Agbebiyi, A.; James, R. J. E.; Berry, D.; Bonus, A.; Vlaev, I.; Chadborn, T. | 2021 | Increasing uptake of National Health Service Health Checks in primary care: a pragmatic randomized controlled trial of enhanced invitation letters in Northamptonshire, England | Included RCT identified from previous literature search |

**Literature search update: May 2024**
**Titles and abstracts screening with excluded reasons**

| # | Author | Year | Title | Excluded with reason |
|---|---|---|---|---|
| 1 | Akbar, H. G. G., D.;Te Kani, A. W.;Anderson, D.;Windsor, C. | 2023 | Towards collective community education: Pasifika diabetes health forums in Southeast Queensland, Australia | A |
| 2 | Amira, B. G., B.;Dorra, B.;Imen, Y.;Mariem, M.;Hanene, B. S.;Najla, M.;Nizar, L. | 2023 | SLEEP APNEA SYNDROME AND NIGHT WORK IN HEALTH CARE PERSONNEL | A |
| 3 | Andrade, G. I., I.;Hsieh, M. K.;Milani, G.;Zandoná, P. C. E.;Teixeira, T. A.;Drevet, J. R.;Costa, E. M.;Hallak, J. | 2023 | Serum lipid profile levels and semen quality: new insights and clinical perspectives for male infertility and men's health | A |
| 4 | Andréka, L. C., O.;Andréka, P.;Vajer, P. | 2023 | [Health status and cardiovascular risk of Roma and non-Roma population in underprivileged settlements] | A |
| 5 | Anwar, K. S., ;Sinha, P.;Sinha, A. | 2023 | A Study on Menstrual Hygiene and its Association with Perceived Reproductive Morbidity in Adolescent Girls of Slum of Patna District, Bihar, India | A |
| 6 | Ayu, F. D., F. I. S.;Sahri, M.;Sunaryo, M. | 2023 | Relationship between the level of participation in implementing fit-to-work on unsafe behavior in lift and transport operators at PT. BJTI | A |
| 7 | Bahreini, M. S. S., S.;Badalzadeh, Y.;Motazedian, M. H.;Shirani, M.;Jahromi, S. S.;Teimouri, A.;Agholi, M.;Asgari, Q. | 2023 | Molecular diagnosis of <i>Trichomonas vaginalis</i> in liquid-based Papanicolaou samples in Shiraz, southern Iran | A |
| 8 | Bailey, H. C., P. A. J.;Darby, M.;Franks, K.;Gabe, R.;Kennedy, M. P. T.;Tam, H. Z.;Brunelli, A.;Callister, M. E. J. | 2023 | OUTCOMES AFTER CURATIVE TREATMENT FOR PATIENTS DIAGNOSED WITH CLINICAL STAGE I LUNG CANCER IN THE YORKSHIRE LUNG SCREENING TRIAL | A |
| 9 | Bhamani, A. H., C.;Bojang, F.;Quaife, S. L.;Dickson, J. L.;Tisi, S.;Hall, H.;Verghese, P.;Creamer, A.;Prendecki, R.;McCabe, J.;Gyertson, K.;Bowyer, V.;El -Emir, E.;Cotton, A.;Mehta, S.;Levermore, C.;Mullin, A. M.;Teague, J.;Farrelly, L.;Nair, A.;Devaraj, A.;Hackshaw, A.;Janes, S.;Summit Consortium | 2023 | The SUMMIT Study: Utilising a written 'Next Steps' information booklet to prepare participants for potential lung cancer screening results and follow-up | A |
| 10 | Bhamani, A. K., E.;Bojang, F.;Dickson, J. L.;Horst, C.;Tisi, S.;Hall, H.;Verghese, P.;Creamer, A.;Prendecki, R.;Khaw, C. R.;McCabe, J.;Gyertson, K.;Hacker, A. M.;Farrelly, L.;Hackshaw, A.;Quaife, S. L.;Janes, S. M. | 2023 | THE SUMMIT STUDY: FOUR-WEEK QUIT RATES AMONGST INDIVIDUALS REFERRED TO STOP SMOKING SERVICES FOLLOWING ATTENDANCE AT A LUNG HEALTH CHECK | A |
| 11 | Boitano, T. K. L. G., A.;Chu, D. I.;Leath, C. A., III;Straughn, J. M., Jr.;Smith, H. J. | 2023 | Use of a mobile health patient engagement technology improves perioperative outcomes in gynecologic oncology patients | A |

| | | | | |
|---|---|---|---|---|
| 12 | Bradley, C. A., P.;Baldwin, D. R.;Booton, R.;Darby, M.;Eckert, C. J.;Gabe, R.;Hancock, N.;Janes, S.;Kennedy, M.;Lindop, J.;Neal, R. D.;Rogerson, S.;Shinkins, B.;Simmonds, I.;Upperton, S.;Vestbo, J.;Crosbie, P. A. J.;Callister, M. E. J. | 2023 | Measuring spirometry in a lung cancer screening cohort highlights possible underdiagnosis and misdiagnosis of COPD | A |
| 13 | Bradley, C. B., A.;Clarke, L.;Dallinson, N.;Eckert, C.;Ellames, D.;Finn, J.;Gabe, R.;Hancock, N.;Kennedy, M. P. T.;Lindop, J.;Mohamed, A.;Mullen, G.;Murray, R. L.;Rogerson, S.;Shinkins, B.;Simmonds, I.;Upperton, S.;Wilkinson, A.;Crosbie, P. A.;Callister, M. E. J. | 2023 | Diagnosis and treatment outcomes from prebronchodilator spirometry performed alongside lung cancer screening in a Lung Health Check programme | A |
| 14 | Bros, I. N., S.;Lewendon, J.;Anderson, C. | 2023 | TYPE 1 DIABETES YOUTH CLINIC MODEL: TAKING THE CLINIC TO THE COMMUNITY | A |
| 15 | Calderwood, C. M., E.;Sibanda, S.;Madziva, K.;Dixon, J.;Fielding, K.;Kranzer, K. | 2023 | IMPLEMENTATION OF INTEGRATED HEALTH-CHECKS FOR TB-AFFECTED HOUSEHOLDS IN ZIMBABWE | A |
| 16 | Cameron, J. S.-T., D.;Li, J.;Varnfield, M.;Allan, L. P.;Smallbon, V.;Redd, C.;Lannin, N. A.;Cadilhac, D. A. | 2023 | Co-design of a multicomponent digital Care Assistant and support Program for people after Stroke or transient ischaemic attack (CAPS) | A |
| 17 | Campbell, F. M., K.;Julian, M.;Woodhead, T.;MacKenzie, P.;Crompton, L.;Powney, B. | 2023 | Improving care and outcomes for teenagers and young adults with diabetes: A new blended training programme for healthcare professionals | A |
| 18 | Choi, E. Y. P., J. S.;Min, D. L.;Ahn, S.;Ahn, J. A. | 2023 | Heart Failure-Smart Life: a randomized controlled trial of a mobile app for self-management in patients with heart failure | A |
| 19 | Choi, K. P., J. S.;Kwon, Y. S.;Park, S. H.;Kim, H. J.;Noh, H.;Won, K. S.;Song, B. I.;Kim, H. W. | 2023 | Development of lung cancer risk prediction models based on F-18 FDG PET images | A |
| 20 | Chung, C. L., K. N.;Han, K.;Shin, D. W.;Lee, S. W. | 2023 | Effect of smoking on the development of chronic obstructive pulmonary disease in young individuals: a nationwide cohort study | A |
| 21 | Dheepa Gayathri, L. A., D.;Viveka, M.;Raju, A.;Suganthy, K.;Shenoy, M. T.;Hariharan, A. | 2023 | Study of Dyselectrolytemia in a Tertiary Care Hospital | A |
| 22 | Dickson, J. L. H., H.;Horst, C.;Tisi, S.;Verghese, P.;Mullin, A. M.;Teague, J.;Farrelly, L.;Bowyer, V.;Gyertson, K.;Bojang, F.;Levermore, C.;Anastasiadis, T.;McCabe, J.;Navani, N.;Nair, A.;Devaraj, A.;Hackshaw, A.;Quaife, S. L.;Janes, S. M.;Summit consortium | 2023 | Uptake of invitations to a lung health check offering low-dose CT lung cancer screening among an ethnically and socioeconomically diverse population at risk of lung cancer in the UK (SUMMIT): a prospective, longitudinal cohort study | A |
| 23 | Dunphy, M. C. | 2023 | Midwives can conduct a cervical screen test (CST)at a 6-week postnatal comprehensive health check | A |
| 24 | Eccles, S. R. L., D.;Morgan, J.;Wright, C.;Coslett, C.;Smith, A. | 2023 | PLANNING THORACIC SURGERY CAPACITY FOR LUNG CANCER SCREENING IN WALES | A |

| | | | | |
|---|---|---|---|---|
| 25 | Eun, Y. H., K.;Lee, S. W.;Kim, K.;Kang, S.;Lee, S.;Cha, H. S.;Koh, E. M.;Kim, H.;Lee, J. | 2023 | Altered Risk of Incident Gout According to Changes in Metabolic Syndrome Status: A Nationwide, Population-Based Cohort Study of 1.29 Million Young Men | A |
| 26 | Francis, C. H. R. H., J. I.;Ryanna, K.;Batista, C. M. | 2023 | THE CLINICAL CHARACTERISTICS OF PATIENTS WITH MODERATE OR SEVERE EMPHYSEMA IDENTIFIED BY THE TARGETED LUNG HEALTH CHECK SCREENING PROGRAMME | A |
| 27 | Fuat, P. A. A., E.;Monane, M.;Coll, R.;Little, E.;Wild, J.;Kamali, F.;Soni, Y.;Haining, S.;Riding, H.;Delgado, C. G.;Weale, M. E.;Harrison, S.;Donnelly, P. | 2023 | Patient and Primary Care Provider Experiences From an Implementation Study of a Risk Prediction Tool Integrating a Polygenic Risk Score and the QRISK2 Cardiovascular Risk Calculator | A |
| 28 | Fukuda, K. M., H.;Nomura, Y.;Nakao, T.;Yamaguchi, T.;Hanaoka, S.;Saito, A.;Nagase, T.;Yoshikawa, T. | 2023 | Association of 18F-FDG uptake in semiquantitative PET/CT with emphysema progression in smokers: A retrospective observational study | A |
| 29 | Galindo, C. A. F., A.;Abdallah, I.;Courtenay-Quirk, C. | 2023 | Positive Health Check intervention tool usage during a feasibility pilot in HIV primary care clinics | A |
| 30 | Goodley, P. B., H.;Alonso, A.;Brockelsby, C.;Conroy, M.;Cooper-Moss, N.;Craig, C.;Evison, M.;Hewitt, K.;Higgins, C.;Johnson, W.;Lyons, J.;Merchant, Z.;Rowlands, A.;Sharman, A.;Sinnott, N.;Sperrin, M.;Booton, R.;Crosbie, P. A. J. | 2023 | Invitation strategies and participation in a community-based lung cancer screening programme located in areas of high socioeconomic deprivation | A |
| 31 | Goodley, P. B., H.;Sperrin, M.;Booton, R.;Crosbie, P. A. J. | 2023 | OA07.03 Performance of PLCOm2012-Based Screening Eligibility in the Manchester Lung Health Check Pilot Cohort after 6 years | A |
| 32 | Goodley, P. C., P.;Booton, R.;Balata, H. | 2023 | When to re-invite initially ineligible populations for lung cancer screening? Modelling from Manchester Lung Health Check cohorts | A |
| 33 | Goonoo, M. S. S., D.;Sloan, G. P.;Tesfaye, S. | 2023 | Abnormal Combined Point-of-Care-Device DPN-Check and SUDOSCAN Results Predict All-Cause Mortality in People with Diabetes | A |
| 34 | Guo, H. J. Z., J. S.;Chen, H.;Cao, X. | 2023 | Prevalence and associated factors of poor sleep quality among Chinese retirees: A multicenter cross-sectional study | A |
| 35 | Harris, R. W., S.;Abbott, M.;Bennett, L.;Morling, J.;Wilkes, E.;Guha, N. | 2023 | ADDRESSING HEALTH INEQUALITY IN CHRONIC LIVER DISEASE BY EVOLVING COMMUNITY PATHWAYS IN NOTTINGHAM | A |
| 36 | Hashimoto, K. N., H.;Sakai, A.;Shimabukuro, M.;Kazama, J. J.;Takahashi, A.;Ohira, T.;Hashimoto, S.;Tsubokura, M.;Watanabe, K.;Hayashi, F.;Nagao, M.;Okazaki, K.;Sato, S.;Yasumura, S.;Ohto, H.;Kamiya, K.;Hosoya, M. | 2023 | Blood data trends of children in Fukushima after the Great East Japan Earthquake: Fukushima health management survey | A |

| | | | | |
|---|---|---|---|---|
| 37 | Hidaka, Y. T., Takayuki;Maruta, Michio;Makizako, Hyuma;Ikeda, Yuriko;Nakamura, Atsushi;Han, Gwanghee;Miyata, Hironori;Shimokihara, Suguru;Akasaki, Yoshihiko;Kamasaki, Taishiro;Kubozono, Takuro;Ohishi, Mitsuru | 2023 | Relationship between grave visitation and apathy among community-dwelling older adults | A |
| 38 | Hoeck, S. T., T. N. | 2023 | Self-Reported Reasons for Inconsistent Participation in Colorectal Cancer Screening Using FIT in Flanders, Belgium | A |
| 39 | Hussain, S. N., A.;Trushell-Pottinger, D.;Waldron, I.;Page, J.;Matthews, S.;Kyi, M. | 2023 | REAL-WORLD IMPACT OF TARGETED LUNG HEALTH CHECK (TLHC) PROGRAMME ON DOWNSTREAM ACTIVITIES IN THE SECONDARY CARE AND BEYOND: A PILOT SITE EXPERIENCE | A |
| 40 | Ichikado, K. I., H.;Iyonaga, K.;Kawamura, K.;Higashi, N.;Johkoh, T.;Fujimoto, K.;Morinaga, J.;Yoshida, M.;Mitsuzaki, K.;Suga, M.;Tanabe, N.;Handa, T.;Hirai, T.;Sakagami, T. | 2023 | An observational cohort study of interstitial lung abnormalities (ILAs) in a large Japanese health screening population (Kumamoto ILA study in Japan: KILA-J) | A |
| 41 | Ikeda, Y. K., R.;Takata, Y.;Tabara, Y.;Maruyama, K.;Takakado, M.;Hadate, T.;Ohashi, J.;Saito, I.;Ogawa, Y.;Osawa, H. | 2023 | Resistin G–A haplotype at SNP-420/-358 is associated with the latent sarcopenic obesity index in the toon genome study | A |
| 42 | Jackson, J. A. L., P.;Sayed-Noor, A. S.;Punnett, L.;Wahlström, J. | 2023 | Risk factors for surgically treated cervical spondylosis in male construction workers: a 20-year prospective study | A |
| 43 | James-Morley, E. P., I.;Ryanna, K.;Walder, D. P. | 2023 | A PILOT OF CASE-FINDING FOR COPD WITH COMMUNITY SPIROMETRY WITHIN A TARGETED LUNG HEALTH CHECK (TLHC) PROGRAMME | A |
| 44 | Jiang, X. Z., L.;Gao, Y.;He, C.;Tang, Z.;Sun, J. | 2023 | An Empirical Study on Physical Subhealth Risk Perception: A Physical Examination Data of Tertiary Grade-A Hospitals in Anhui Province, China | A |
| 45 | Jones, J. L. S., K.;Manski-Nankervis, J. A.;Lumsden, N. G.;Fernando, S.;de Courten, M. P.;Cox, N.;Hamblin, P. S.;Janus, E. D.;Nelson, C. L. | 2023 | Chronic disease IMPACT (chronic disease early detection and improved management in primary care project): An Australian stepped wedge cluster randomised trial | A |
| 46 | Kaawa-Mafigiri, D. N., M.;Odie, M.;Johnson, J. L. | 2023 | Perceptions about and reasons for participation in research bronchoscopy in Uganda: A qualitative analysis | A |
| 47 | Kaneko, H. T., H. | 2023 | Depressive symptoms predict antibody titers after a second dose of the SARS-CoV-2 BNT162b2 vaccine among hospital workers in Japan | A |
| 48 | Kang, J. K., T.;Han, K. D.;Jung, J. H.;Jeong, S. M.;Yeo, Y. H.;Jung, K.;Lee, H.;Cho, J. H.;Shin, D. W. | 2023 | Risk factors for early-onset lung cancer in Korea: analysis of a nationally representative population-based cohort | A |
| 49 | Kasashi, K. S., A.;Stuart, M.;Hollywood, U.;Kawaguchi-Suzuki, M.;Yagishita, K.;Akama, T. | 2023 | The Tokyo 2020 Olympic and Paralympic pharmacy services during the COVID-19 pandemic | A |
| 50 | Kazemian, A. H., M.;Rad, S. A. B.;Jouya, A.;Tahani, B. | 2023 | Nudging oral habits; application of behavioral economics in oral health promotion: a critical review | A |

| | | | | |
|---|---|---|---|---|
| 51 | Khatua, C. R. S., S. P. | 2023 | A study of the prevalence of acute and chronic liver diseases among newly detected HBsAg positive subjects: A single centre experience | A |
| 52 | Kommavarapu, H. M., S.;Nix, J.;Montoya, O.;Pamganamamula, T.;Dronavalli, G.;Pamganamamula, M. | 2023 | THYROID NODULES ON THE RISE: UNDERSTANDING PREVALENCE AND RISK FACTORS IN PRIMARY CARE PATIENTS | A |
| 53 | Kotti, T. K., E.;Ruparel, M.;McEwen, A.;Dickson, J. L.;Duffy, S. W.;Waller, J.;Janes, S. M.;Quaife, S. L. | 2023 | A randomised controlled trial testing acceptance of practitioner-referral versus self-referral to stop smoking services within the Lung Screen Uptake Trial | A |
| 54 | Kotze, M. V. R., S.;Davids, M.;Stroetmann, K.;Daramola, F.;Nyasulu, P.;Von Metzinger, J.;Daramola, O. | 2023 | TRANSLATING POPULATION RISK INTO PERSONAL UTILITY USING A MOBILE PHONE APP FOR APPLICATION OF GENOMIC MEDICINE INTEGRATING SERVICE AND RESEARCH IN THE COVID-19 ERA | A |
| 55 | Ku, P. W. S., A.;Lai, Y. J.;Yen, Y. F.;Ahmadi, M.;Inan-Eroglu, E.;Wangj, S. F.;Chen, L. J.;Stamatakis, E. | 2023 | Are associations of leisure-time physical activity with mortality attenuated by high levels of chronic ambient fine particulate matter (PM2.5) in older adults? A prospective cohort study | A |
| 56 | Kuehl, K. E., Diane;DeFrancesco, Carol;McGinnis, Wendy;Ek, Susanna;Garg, Bharti | 2023 | A web-based total worker health intervention for those fighting wildland fires: Mixed methods development and effectiveness trial | A |
| 57 | Kumar, R. A.-u.-D., R.;Ahmed, J.;Asim, M.;Rashid, F.;Khan, S. A.;Ali, S.;Pongpanich, S. | 2023 | Correlates of early initiation of breast feeding and prelacteal feeding: a cross-sectional study in Sindh province of Pakistan | A |
| 58 | Kwon, S. K., B.;Han, K. D.;Jung, W.;Cho, E. B.;Shin, D. W.;Min, J. H. | 2023 | Increased Risk of Ischemic Stroke in Amyotrophic Lateral Sclerosis: A Nationwide Cohort Study in South Korea | A |
| 59 | Lagnado, A. T., S.;May, T.;Kesten, J.;Yardley, L.;Hawken, J.;Gitahi, J.;Hickman, M.;Gordon, F.;Abeysekera, K. | 2023 | ACCEPTABILITY AND FEASIBILITY OF A MULTIMODAL EARLY DETECTION PILOT STUDY FOR LIVER DISEASE IN HIGH RISK GROUPS: “ALRIGHT MY LIVER?” | A |
| 60 | Laowjan, P. M., K.;Permpool, P.;Talungchit, P.;Jareemit, N. | 2023 | Factors Associated with Cervical Cancer Screening Overuse and Underuse, and Attitude towards Human Papillomavirus Self-sampling among Hospital Staffs | A |
| 61 | Lee, R. W. B., H.;Bartholomeuz, T.;Booton, R.;Callister, M.;Cheyne, L.;Crosbie, P.;Daneshvar, C.;Desai, D.;Devaraj, A.;Grundy, S.;Hussein, I.;McGeachy, D.;Messenger, J.;Howells, J.;Janes, S.;Muller, M.;Osborne, C.;Page, J.;Quaife, S.;Raza, S.;Randle, A.;Rawlinson, J.;Richards, P.;Stevenson, A.;Tsaknis, G.;Ward, A.;Sasieni, P.;Baldwin, D.;Nair, A. | 2023 | IMPLEMENTING LUNG CANCER SCREENING IN THE UK: BASELINE RESULTS FROM THE NHS ENGLAND NATIONAL 'TARGETED LUNG HEALTH CHECK' PROGRAMME | A |
| 62 | Lee, S. E. Y., J.;Choi, H. S.;Han, K.;Kim, K. A. | 2023 | Two-Year Changes in Diabetic Kidney Disease Phenotype and the Risk of Heart Failure: A Nationwide Population-Based Study in Korea | A |

| | | | | |
|---|---|---|---|---|
| 63 | Lefebvre, L. R., C.;Grunemwald, T.;Hamrene, K.;Momas, I. | 2023 | Unsupervised identification of cardiometabolic profiles among adolescents: Findings from the PARIS birth cohort study | A |
| 64 | Li, S. L., Cailian;Kang, Lin;Li, Qianqian;Chen, Hongxu;Zhang, Han;Tang, Ziling;Lin, Yanwen;Bai, Meiyan;Xiong, Peng | 2023 | Study on correlations of BDNF, PI3K, AKT and CREB levels with depressive emotion and impulsive behaviors in drug-naïve patients with first-episode schizophrenia | A |
| 65 | Lin, H. J. W., J.;Tseng, P. Y.;Fu, L. C.;Lee, Y. C.;Wu, M. S.;Yang, W. S.;Chiu, H. M. | 2023 | Lower-than-normal glycemic levels to achieve optimal reduction of diabetes risk among individuals with prediabetes: A prospective cohort study | A |
| 66 | Lizhi, J. P., L.;Jiayi, X.;Xufei, L.;Fulai, S.;Wenqin, G.;Ya, S. | 2023 | An interview survey on the development of family doctor work mode in Shanghai Xuhui district | A |
| 67 | Magnavita, N. | 2023 | Workplace Health Promotion Embedded in Medical Surveillance: The Italian Way to Total Worker Health Program | A |
| 68 | Matsuo, R. M., N.;Mitsuhashi, T.;Yorifuji, T. | 2023 | COVID-19 pandemic and language development in children at 18 months: a repeated cross-sectional study over a 6-year period in Japan | A |
| 69 | Matsuol, R. I., T.;Takamori, A.;Kishi, T.;Minami, M.;Miyakawal, J.;Yoshitakel, N.;Hayashil, A.;Nakayamal, Y.;Egashiral, N.;Teramotol, M.;Ishinaril, H.;Kajiyamal, I.;Fujisakil, S.;Kakiyamal, H.;Satoul, K.;Nakafusal, R.;Tanakal, C.;Tanakal, M.;Isomural, Y.;Izumi, K.;Ohta, S.;Souta, N.;Matsuo, N.;Yamamoto, K.;Tsuji, S.;Umemura, T.;Takagi, K.;Fujimoto, K. | 2023 | Improvement trend for individual health guidance intervention according to Japan clinical guidelines by public health nurses for type 2 diabetes mellitus who visited for medical checkups regularly: a case-control preliminary report | A |
| 70 | McCartan, C. J. R., J.;Jordan, J. A. | 2023 | Centre-based early education interventions for improving school readiness: A systematic review | A |
| 71 | McDonagh, S. T. J. N., B.;Fordham, A. J.;Greenwood, M. R.;Richards, S. H.;Campbell, J. L.;Clark, C. E. | 2023 | Inter- arm blood pressure difference and cardiovascular risk estimation in primary care: a pilot study | A |
| 72 | McHugh, N. B., L.;Coates, L.;Davies, C.;Helliwell, P.;Packham, J.;Ransom, M.;Spackman, E.;Tillett, W.;Brown, S. | 2023 | INCIDENCE OF PSORIATIC ARTHRITIS AND PREVALENCE OF UNDIAGNOSED CASES IN A UK PRIMARY CARE POPULATION FOLLOWED PROSPECTIVELY FOR 2 YEARS-RESULTS FROM THE TUDOR RANDOMIZED CLINICAL TRIAL | A |
| 73 | Michel, M. A. S., M.;Dindorf, C.;Danguy, V.;Chevreul, K. | 2023 | Bilans de santé pour la population agricole sous-consommante française : évaluation pilote du programme des Instants santé de la MSA | A |
| 74 | Moghul, M. C., F.;Kaur, K.;Kinsella, N.;Cahill, D.;James, N. | 2023 | The Man Van - Community Based Targeted Case Finding For Prostate Cancer | A |
| 75 | Moghul, M. C., F.;Kinsella, N.;Cahill, D.;James, N. D. | 2023 | The Man Van: Mobile targeted case finding for prostate cancer | A |
| 76 | Moghul, M. C., F.;Kaur, K.;Kinsella, N.;Cahill, D.;James, N. | 2023 | THE MAN VAN: COMMUNITY BASED TARGETED CASE FINDING FOR PROSTATE CANCER | A |

| | | | | |
|---|---|---|---|---|
| 77 | Molina-Donoso, M. P., T.;Meillon, C.;Thumala, D.;Lillo, P.;Villagra, R.;Ibañez, A.;Cerda, M.;Zitko, P.;Amieva, H.;Slachevsky, A. | 2023 | Assessing subjective cognitive decline in older adults attending primary health care centers: what question should be asked? | A |
| 78 | Mujoo, H. B., N.;Thabrew, H.;Kokaua, J.;Audas, R.;Taylor, B. | 2023 | Identifying neurodevelopmental disabilities from nationalised preschool health check | A |
| 79 | Nagasawa, M. T., T.;Kawagoe, S.;Yamaguchi, N.;Morita, Y.;Yokoe, T.;Ota, T.;Izumi, T.;Ishida, Y.;Chosa, E. | 2023 | Risk factors associated with throwing injuries in young baseball players | A |
| 80 | Nguyen, C. K. P., H. M.;Lee, C. H.;Do, L. A. T. | 2023 | Assessing Awareness of Colorectal Cancer Symptoms among Outpatients: A Cross-Sectional Study at a Hospital in Vietnam | A |
| 81 | Nievas, C. M. G., B. J.;Toledo, E.;Albornoz, J. | 2023 | [Not Available] | A |
| 82 | Ohuchi, K. K., H.;Saito, H.;Sugimura, Y.;Yoshikawa, T.;Miyakoshi, N. | 2023 | Risk Factors for Glenohumeral Internal Rotation Deficit in Adolescent Athletes: A Comparison of Overhead Sports and Non-overhead Sports | A |
| 83 | Otaki, Y. W., T.;Konta, T.;Watanabe, M.;Fujimoto, S.;Sato, Y.;Asahi, K.;Yamagata, K.;Tsuruya, K.;Narita, I.;Kasahara, M.;Shibagaki, Y.;Iseki, K.;Moriyama, T.;Kondo, M.;Watanabe, T. | 2023 | A Body Shape Index and Aortic Disease-Related Mortality in Japanese General Population | A |
| 84 | Pahk, K. J., C.;Kwon, H. W.;Kim, S. | 2023 | Chronic physical exercise alleviates stress-associated amygdala metabolic activity in obese women: A prospective serial 18F-FDG PET/CT study | A |
| 85 | Pahk, K. K., H.;Kim, S. | 2023 | A 3-month of physical exercise reduces stress-related neurobiological activity in obese women: a prospective 18F-FDG PET/CT study | A |
| 86 | Pathirana, M. M. A., P. H.;Aldridge, E.;Harrison, M.;Harrison, J.;Leemaqz, S.;Arstall, M. A.;Dekker, G. A.;Roberts, C. T. | 2023 | The association of breast feeding for at least six months with hemodynamic and metabolic health of women and their children aged three years: an observational cohort study | A |
| 87 | Philp, L. K., N.;Jorgensen, L.;Bello, A. | 2023 | IMPLEMENTATION OF THE ALBERTA KIDNEY HEALTH CHECK PROTOCOL THROUGH CO-DESIGN WITH INDIGENOUS COMMUNITIES | A |
| 88 | Rai, R. K. B., S. | 2023 | Estimated effect of age of marriage on utilisation of India's Integrated Child Development Service programme | A |
| 89 | Riley, A. W., N.;Harris, M.;Giebel, C. | 2023 | A MIXED METHODS EVALUATION OF A COMMUNITY HEALTH AND WELLBEING WORKER PILOT IN THREE SETTINGS IN ENGLAND | A |
| 90 | Rizakhanova, O. A. A., S. N.;Avdeeva, M. V.;Nikitina, L. Y. | 2023 | Problems of medical care for patients with chronic obstructive pulmonary disease in the administrative territories of the Russian Federation | A |
| 91 | Saaed, F. M. A. O., J. E. | 2023 | Prevalence of Hepatitis B and Hepatitis C in Migrants from Sub-Saharan Africa Before Onward Dispersal Toward Europe | A |

| | | | | |
|---|---|---|---|---|
| 92 | Salman, M. F. C., R.;Judah, G. | 2023 | Impact of messaging informed by behavioural science on uptake of targeted lung health checks: a service evaluation | A |
| 93 | Saravi, N. M. A., F.;Najafi, A.;Khajavi, R.;Rahmani, Z.;Jalali, H.;Mousavi, T. | 2023 | A seromolecular study to determine the prevalence of cytomegalovirus in pregnant women referred to health centers in the north of Iran | A |
| 94 | Schmidt, T. L. C., C.;Dumke, L.;Groß, M.;Neldner, S.;Scharpf, F.;Weitkämper, A.;Wilker, S.;Wittmann, J.;Stammnitz, A.;van den Heuvel, R.;Neuner, F. | 2023 | Welcome, how are you doing? - towards a systematic mental health screening and crisis management for newly arriving refugees | A |
| 95 | Shimizu, Y. H., H.;Honda, E.;Sasaki, N.;Takada, M.;Okada, T.;Ohira, T.;Kiyama, M. | 2023 | Association between serum albumin levels and height loss in Japanese workers: a retrospective study | A |
| 96 | Shimizu, Y. Y., H.;Kitamura, M.;Miyata, J.;Nonaka, F.;Nakamichi, S.;Saito, T.;Nagata, Y.;Maeda, T. | 2023 | Association between periodontitis and chronic kidney disease by functional atherosclerosis status among older Japanese individuals: A cross-sectional study | A |
| 97 | Shrestha, R. K. G., C. A.;Courtenay-Quirk, C.;Harshbarger, C.;Abdallah, I.;Marconi, V. C.;DallaPiazza, M.;Swaminathan, S.;Somboonwit, C.;Lewis, M. A.;Khavjou, O. A. | 2023 | Cost Analysis of the Positive Health Check Intervention to Suppress HIV Viral Load and Retain Patients in HIV Clinical Care | A |
| 98 | Shrivastava, Y. Y., M.;Ganesh, N. | 2023 | Absence of Synergistic Effect of Toluidine Blue and Cytomorphometry in Discriminating Dysplasia in Oral Exfoliative Cytology | A |
| 99 | Sivabalah, K. A., G.;Goodley, P.;Hiu, N. C. J.;Ghoshal, A.;Alonso, A.;Sharman, A.;Sinnott, N.;Brockelsby, C.;Craig, C.;Merchant, Z.;Evison, M.;Crosbie, P.;Booton, R.;Balata, H. | 2023 | The impact of real-world lung cancer screening programmes on downstream diagnostic capacity | A |
| 100 | Sukumar, G. M. R., R.;Philip, M.;Gopalkrishna, G. | 2023 | Reliability of a Newly Developed Tool to Assess and Classify Work-related Stress (TAWS-16) for Indian Workforce | A |
| 101 | Sulaiman, W. S. W. H., R. M. Z. R.;Mohsin, S. H.;Pardan, N. | 2023 | Association between mothers' utilization of oral health care and oral health status of their toddlers in Kota Bharu district, Kelantan | A |
| 102 | Sunjaya, A. P. M., A.;Arnott, C.;Marks, G. B.;Jenkins, C. R. | 2023 | Chronic Breathlessness Management in Primary Care: Current Gaps and Practical Solutions | A |
| 103 | Supínová, M. B., P.;Bartosík, P. | 2023 | The influence of social environment on the health of disadvantaged population groups | A |
| 104 | Szawlowski, S. T., C.;Lagarde, M.;Mbaye, E.;Gueye, K.;Ndour, C. T.;Lépine, A. | 2023 | Reforming the registration policy of female sex workers in Senegal? Evidence from a discrete choice experiment | A |
| 105 | Taruvinga, T. C., R. S.;Marambire, E.;Larsson, L.;Olaru, I. D.;Sibanda, S.;Nzvere, F.;Redzo, N.;Ndhlovu, C. E.;Rusakaniko, S.;Mujuru, H.;Sibanda, E.;Chonzi, P.;Siamuchembu, M.;Chikodzore, R.;Mahomva, A.;Ferrand, R. A.;Dixon, J.;Kranzer, K. | 2023 | Exploring COVID-19 vaccine uptake among healthcare workers in Zimbabwe: A mixed methods study | A |
| 106 | Thies-Lagergren, L. J., M. | 2023 | Home-based postnatal midwifery care facilitated a smooth succession into motherhood: A Swedish interview study | A |

| | | | | |
|---|---|---|---|---|
| 107 | Tonouchi, T. K., H.;Maruyama, H.;Miida, S.;Sakai, N.;Watanabe, Y.;Kimura, N.;Setsu, T.;Abe, H.;Yokoo, T.;Sakamaki, A.;Tsuchiya, A.;Kamimura, K.;Terai, S. | 2023 | CLINICAL SUPPORT USING DEEP LEARNING TO PREDICT THE NEED FOR INTERVENTION IN ELDERLY PATIENTS WITH PRIMARY BILIARY CHOLANGITIS FROM MULTICENTER DATA | A |
| 108 | Tsai, J. S., V.;Schick, V. | 2023 | Medical Care Needs of Laundromat Users in San Antonio, Texas: A Potentially Unique Setting for Health Interventions | A |
| 109 | Tsuji, M. W., K.;Nagata, C. | 2023 | Associations of soy food intake with menopausal symptoms, including hot flushes, in Japanese women | A |
| 110 | Twisk, D. E. W., A.;Götz, H. M. | 2023 | Community-based HIV testing through a general health check event in a high HIV-prevalent multicultural area in Rotterdam, The Netherlands: a pilot study on feasibility and acceptance | A |
| 111 | Usher, K. J., D.;Kabir, H.;Jones, R.;Miller, J.;Peake, R.;Smallwood, R. | 2023 | Preventative health assessments and indigenous people of Australia: a scoping review | A |
| 112 | Van Hoang, D. I., Y.;Fukunaga, A.;Nakagawa, T.;Honda, T.;Yamamoto, S.;Okazaki, H.;Yamamoto, M.;Miyamoto, T.;Gommori, N.;Kochi, T.;Shirasaka, T.;Eguchi, M.;Ogasawara, T.;Yamamoto, K.;Konishi, M.;Katayama, N.;Kabe, I.;Dohi, S.;Mizoue, T. | 2023 | Metabolic syndrome and the risk of severe cancer events: a longitudinal study in Japanese workers | A |
| 113 | Verma, M. K., J. K.;Balusamy, D.;Mehta, S. | 2023 | Causality Assessment of Different Set of Variables using Time Series Data Analysis — A Prospective Cohort Study | A |
| 114 | von Sommoggy, J. G., E. M.;Apfelbacher, C.;Brandstetter, S.;Curbach, J. | 2023 | Pediatricians' experiences of managing outpatient care during the COVID-19 pandemic: A qualitative study in Germany | A |
| 115 | Wang, Y. Q. Y., T.;Deng, S. W.;Zhu, X. L.;Deng, Y. L.;Liu, X. L.;Liu, L.;Wang, C. F. | 2023 | Metabolic health phenotype better predicts subclinical atherosclerosis than body mass index-based obesity phenotype in the non-alcoholic fatty liver disease population | A |
| 116 | Ward, A. M., M.;Sharrock, R.;Lou, T.;Tasker, C. | 2023 | Incidental findings arising from targeted lung health checks: a GP practice perspective | A |
| 117 | Ward, T. J. C. C., L.;Rai, S.;Steiner, M. C.;Tsaknis, G. | 2023 | EMPHYSEMA IDENTIFIED DURING LUNG CANCER SCREENING - AN OPPORTUNITY TO INTERVENE OR JUST ANOTHER INCIDENTAL FINDING? | A |
| 118 | Watson, J. G., M. A.;Giebel, C.;Darlington-Pollock, F.;Akpan, A. | 2023 | Social and spatial inequalities in healthcare use among people living with dementia in England (2002-2016) | A |
| 119 | Weichenberger, M. E., K.;Müller, S. | 2023 | Exercise testing in top athletes | A |
| 120 | Wickramasinghe, K. H., M. M.;Buoncristiano, M.;Pudule, I.;Rito, A. I.;Spinelli, A.;Ahrens, W.;Borghi, E.;Flores-Urrutia, M. C.;McColl, K.;Sassi, F.;Williams, J.;Rakovac, I. | 2023 | Improving data on overweight, obesity and undernutrition among children under the age of 5 years in the WHO European Region | A |

| | | | | |
|---|---|---|---|---|
| 121 | Williams, P. J. P., K. E. J.;Gill, N. K.;Flannery, D.;Buttery, S.;Bartlett, E. C.;Devaraj, A.;Kemp, S. V.;Addis, J.;Derbyshire, J.;Chen, M. C. L.;Morris, K.;Laverty, A. A.;Hopkinson, N. S. | 2023 | Immediate, Remote Smoking Cessation Intervention in Participants Undergoing a Targeted Lung Health Check Quit Smoking Lung Health Intervention Trial, a Randomized Controlled Trial | A |
| 122 | Williams, P. J. P., K. E. J.;Buttery, S. C.;Perkins, A.;Chan, L. Y.;Bartlett, E. C.;Devaraj, A.;Kemp, S.;Addis, J.;Derbyshire, J.;Chen, M. C. L.;Polkey, M.;Laverty, A. A.;Hopkinson, N. S. | 2023 | Immediate smoking cessation support during lung cancer screening: long-term outcomes from two randomised controlled trials | A |
| 123 | Winter, H. A., F.;Matthews, C.;Hajinur, H.;Mohamed, A.;Dawson, S. | 2023 | Ethnically diverse communities and their priorities for cancer research | A |
| 124 | Xu, H. S., L.;Wang, H.;Wang, X.;Zheng, S.;Jiang, Y. | 2023 | Prevalence of nonalcoholic fatty liver disease in community-dwelling elderly women and its influencing factors | A |
| 125 | Xue, B. Y., N.;Shihuan, L.;Xinhuan, Z.;Ronghui, X.;Yahui, S.;Yuntao, L. | 2023 | Stratified management for cardiovascular diseases risk in community population based on China-PAR | A |
| 126 | Yang, H. L., Z. Y.;Lin, F.;Li, L. J.;Lu, M.;Xie, L. X.;Yang, L. Y. | 2023 | Comparison of Urine and Genital Samples for Detecting Human Papillomavirus (HPV) in Clinical Patients | A |
| 127 | Zaman, M. d. V., M. L.;Coultas, C.;Goff, L.;Mernagh-Iles, A.;L'Esperance, V.;Karamanos, A.;Ayis, S.;Ćurčin, V.;Durbaba, S.;Inyang, M.;Molokhia, M.;Harding, S. | 2023 | Factors affecting the delivery of community-based salon interventions to prevent cardiovascular disease and breast cancer among ethnically diverse women in South London: a concept-mapping approach | A |
| 128 | Zhan, Y. R., X.;Huang, D.;Huang, J.;Huang, J.;Chun, T. T.;Ho, B.;Ng, A.;Tsu, J.;Na, R. | 2023 | EFFECTS OF PROSTATE-SPECIFIC ANTIGEN SCREENING ON PROSTATE CANCER INCIDENCE AND MORTALITY: A POPULATION-BASED COHORT STUDY IN CHINA | A |
| 129 | Zhao, D. L., J. J.;Li, J.;Gao, T. T.;Fu, P. P.;Wang, Y.;Zhou, C. C. | 2023 | Tooth loss, body mass index and cognitive function among middle-aged and older adults in China: Does gender matter? | A |
| 130 | Abeysekera, K. W. M. V., L.;Younossi, Z.;Dillon, J. F.;Allen, A. M.;Nourredin, M.;Rinella, M. E.;Tacke, F.;Francque, S.;Ginès, P.;Thiele, M.;Newsome, P. N.;Guha, I. N.;Eslam, M.;Schattenberg, J. M.;Alqahtani, S. A.;Arrese, M.;Berzigotti, A.;Holleboom, A. G.;Caussy, C.;Cusi, K.;Roden, M.;Hagström, H.;Wong, V. W.;Mallet, V.;Castera, L.;Lazarus, J. V.;Tsochatzis, E. A. | 2024 | Implementation of a liver health check in people with type 2 diabetes | A |
| 131 | Abraham, M. M., T.;Greetham, H.;Hicks, A.;Bainbridge, H. | 2024 | 171 The impact of introducing a Targeted Lung Health Check on oncology services at Portsmouth Hospitals University NHS Trust | A |
| 132 | Akbar, H. C., M.;Niumata, W.;Anderson, D.;Gallegos, D. | 2024 | Effectiveness of the Pasifika Women's Diabetes Wellness Program (PWDWP): Protocol for a Pilot Intervention and Feasibility Randomized Controlled Trial | A |
| 133 | Andersson, N. W., T.;Nylander, E.;Idahl, A. | 2024 | Seroprevalence of sexually transmitted infections over 44 years - A cross-sectional study in Sweden | A |

| | | | | |
|---|---|---|---|---|
| 134 | Arriaza, R. S.-G., M.;Arriaza, A.;Cruz-Cámara, A.;Leyes, M.;Cerezal, L.;Maestro, A. | 2024 | Prevalence of hip femoroacetabular impingement deformities in high-level (La Liga) male professional football players | A |
| 135 | Bone, H. S., T.;Taylor, R. | 2024 | 176 Ensuring Health Equality in Lung Cancer Screening | A |
| 136 | Buss, V. H. B., M.;Parker, S. M.;Kabir, A.;Lau, A. Y. S.;Liaw, S. T.;Stocks, N.;Harris, M. F. | 2024 | Mobile App Intervention of a Randomized Controlled Trial for Patients With Obesity and Those Who Are Overweight in General Practice: User Engagement Analysis Quantitative Study | A |
| 137 | Cai, G. L., Y.;Zhuang, J.;Chen, Z.;Lu, Y.;Wu, J.;Hu, Z.;Zhang, J.;He, F. | 2024 | Differences in socio-demographics status, risk behaviours, healthcare uptake and HIV/ sexually transmitted infections (STIs) between brothel-based and street-based female sex workers in Yunnan, China | A |
| 138 | Calderwood, C. J. M., E.;Nzvere, F. P.;Larsson, L. S.;Chingono, R. M. S.;Kavenga, F.;Redzo, N.;Bandason, T.;Rusakaniko, S.;Mujuru, H. A.;Simms, V.;Khan, P.;Gregson, C. L.;Ndhlovu, C. E.;Ferrand, R. A.;Fielding, K.;Kranzer, K. | 2024 | Prevalence of chronic conditions and multimorbidity among healthcare workers in Zimbabwe: Results from a screening intervention | A |
| 139 | Chen, S. W., Y.;Xie, Y.;Guo, J.;Li, J.;Xie, X.;Li, P. | 2024 | HPV infection rate and subtype distribution among 326 824 cervical cancer screening and health check ups in Hunan Province from 2020 to 2022 | A |
| 140 | Clair-Sullivan, N. S. B., K.;Khan, I.;Maddocks, M.;Harding, R.;Bremner, S.;Levett, T.;Roberts, J.;Adler, Z.;Yi, D.;Vera, J. H. | 2024 | Implementation of frailty screening for older people living with HIV in Brighton, UK | A |
| 141 | Coslett, C. W., C. I.;Smith, A.;Grace, M.;Quaife, S.;Brain, K.;Snelling, D.;Ramessur-Marsden, H.;Eccles, S. | 2024 | 163 Optimising uptake of the Lung Health Check Operational Pilot within a socioeconomically deprived area of Wales | A |
| 142 | Darley, J. C., K.;Drought, J. | 2024 | 170 Patients diagnosed with lung cancer through the Targeted Lung Health Check Programme have a better performance status than patients from other sources of referral, as well as earlier stage disease | A |
| 143 | Eccles, S. E.-V., J.;Abel, N.;Smith, A.;Wright, C.;Coslett, C. | 2024 | 160 Updating primary care smoking records by text message in preparation for lung cancer screening in Wales | A |
| 144 | Gleeson, F. | 2024 | 15 The Integration and Analysis of Data using Artificial Intelligence to Improve Patient Outcomes with Thoracic Diseases (DART) research programme | A |
| 145 | Goodley, P. A., D.;Merchant, Z.;Hewitt, K.;Crosbie, P.;Booton, R. | 2024 | 181 Targeted Lung Health Check screening outcomes from invited ‘aged-in’ populations | A |
| 146 | Goodley, P. B., H.;Alonso, A.;Brockelsby, C.;Conroy, M.;Cooper-Moss, N.;Craig, C.;Evison, M.;Hewitt, K.;Higgins, C.;Johnson, W.;Lyons, J.;Merchant, Z.;Rowlands, A.;Sharman, A.;Sinnott, N.;Sperrin, M.;Booton, R.;Crosbie, P. A. J. | 2024 | Invitation strategies and participation in a community-based lung cancer screening programme located in areas of high socioeconomic deprivation | A |
| 147 | Graham, S. W., J. L.;Andrews, N.;Hulme, W. J.;Nitsch, D.;Parker, E. P. K.;McDonald, H. I. | 2024 | Quantifying and adjusting for confounding from health-seeking behaviour and healthcare access in observational research | A |

| | | | | |
|---|---|---|---|---|
| 148 | Harkus, S. M., V.;O'Keeffe, I.;Kung, C.;Ward, M.;Orr, N.;Skinner, J.;Hughes, J. K.;Fonua Wiradjuri, L.;Kennedy Wiradjuri, M.;Kong Worimi, K.;Belfrage, M. | 2024 | Development of the national consensus statement on ear health and hearing check recommendations for Aboriginal and Torres Strait Islander children aged under 6 years attending primary care: systematic scoping review and e-Delphi | A |
| 149 | Hermanussen, M. S., C. | 2024 | Stop stunting-A misguided campaign by well-meaning nutritionists | A |
| 150 | Hsu, C. L. W., P. C.;Wu, F. Z.;Yu, H. C. | 2024 | LASSO-derived model for the prediction of lean-non-alcoholic fatty liver disease in examinees attending a routine health check-up | A |
| 151 | Huang, X. Y., H.;Huang, E.;Wang, S.;Chen, J.;Weng, J.;Cho, P. S. P.;Moon, M. H.;Song, M.;Fang, X. | 2024 | Feasibility and performance of the chronic obstructive pulmonary disease population screener and chronic obstructive pulmonary disease screening questionnaire in a Chinese physical examination center | A |
| 152 | Inoue, S. S., Y.;Miyazaki, O.;Hanawa, T.;Minegishi, Y.;Murano, H.;Sato, K.;Kobayashi, M.;Sato, M.;Nemoto, T.;Nishiwaki, M.;Igarashi, A.;Ichikawa, K.;Watanabe, T.;Watanabe, M. | 2024 | Participant Selection from the General Japanese Population for Pulmonary Function Tests Using a Questionnaire on Symptoms and Smoking Habits during Annual Health Checkups: The Yamagata-Takahata Study | A |
| 153 | Iseselo, M. K. T., E. A. M. | 2024 | Comprehension of informed consent and voluntary participation in registration cohorts for phase IIb HIV vaccine trial in Dar Es Salaam, Tanzania: a qualitative descriptive study | A |
| 154 | Iyer, A. M., O.;Leslie, K.;Daneshvar, C. | 2024 | 180 Assessment of the down-stream secondary care impact of targeted lung health checks | A |
| 155 | Karavadra, S. B., A.;Khaw, C. R.;Gowers, K.;Gyertson, K.;Janes, S. | 2024 | 167 An evaluation of recruitment for research from a clinical Lung Cancer Screening service | A |
| 156 | Kawamoto, R. K., A.;Ninomiya, D.;Kumagi, T. | 2024 | Aspartate Aminotransferase/Alanine Aminotransferase Ratio: A Predictor of All-Cause Mortality Rate Among Japanese Community-Dwelling Individuals | A |
| 157 | Kim, G. S. B., S.;Kim, N.;Shim, M. S.;Lee, S.;Lee, Y.;Park, C. G.;Kim, L. | 2024 | Network visualization to interpret which healthcare services are central to people living with HIV | A |
| 158 | Kim, J. Y. H., J.;Yoon, J.;Park, J.;Kim, T. H. | 2024 | Regularity of cervical cancer screening in Korea: analysis using national public data for 12 years | A |
| 159 | Koga, H. O., G.;Takagi, S.;Mochizuki, T.;Nishino, K.;Koga, Y. | 2024 | Prevalence and deforming process of Wind-Swept Deformity in Knee Osteoarthritis from the Matsudai Knee Osteoarthritis Survey | A |
| 160 | Landgren, V. S., L.;Törnhage, C. J.;Theodosious, M.;Gillberg, C.;Johnson, M.;Knez, R.;Landgren, M. | 2024 | Neurodevelopmental problems, general health and academic achievements in a school-based cohort of 11-year-old Swedish children | A |

| | | | | |
|---|---|---|---|---|
| 161 | Lee, K. H. L., G.;Lee, T.;Byun, D. W.;Ha, Y. C. | 2024 | Patient Perception on Osteoporosis in Korean Female Patients with Osteoporosis | A |
| 162 | Lefebvre, L. R., F.;Roda, C.;Amazouz, H.;Momas, I. | 2024 | Exposure to sunlight and allergic morbidity in children from the PARIS birth cohort | A |
| 163 | Magnavita, N. M., I.;Viti, G.;Borghese, L. | 2024 | The Work Ability Index (WAI) in the Healthcare Sector: A Cross-Sectional/Retrospective Assessment of the Questionnaire | A |
| 164 | Maltz, A. R., S.;Sarid, A.;Cohen, Y.;Landau, T.;Saifer, E.;Belkin, N. A.;Alcalay, T. | 2024 | The Framing Effect of Digital Textual Messages on Uptake Rates of Medical Checkups: Field Study | A |
| 165 | Melgar, X. C. A., D.;Hugo, F. N. | 2024 | Towards the integration of prevention and control of oral diseases within child primary healthcare: The case of Peru | A |
| 166 | Moghul, M. M., F.;Westaway, E.;Croft, F.;Kinsella, N.;Cahill, D.;James, N. D. | 2024 | The Man Van project: Second phase interim results | A |
| 167 | Moghul, M. Y., D.;Croft, F.;Mutch, F.;Westaway, E.;Kinsella, N.;Cahill, D.;James, N. D. | 2024 | A cost-consequence analysis of the Man Van project: Comparing prostate-specific antigen testing on a nurse-led mobile service with primary care | A |
| 168 | Moon, H. C., Y. J.;Kim, Y. A.;Ju, M. J. | 2024 | Age-specific association of physical activity on visceral obesity: Cross-sectional study | A |
| 169 | Moynihan, A. B., P.;Cucek, J.;Erzen, S.;Hardy, N.;McEntee, P.;Rojc, J.;Cahill, R. | 2024 | Technical and functional design considerations for a real-world interpretable AI solution for NIR perfusion analysis (including cancer) | A |
| 170 | Naidu, S. B. B., A.;Patrick, T.;Anandan, L.;Desai, K.;Robinson, P.;Molyneux, L.;Bojang, F.;Verghese, P.;Nair, A.;Patel, S.;Thakrar, R.;Navani, N.;Janes, S. | 2024 | 169 Can reminders improve lung cancer screening uptake amongst higher-risk populations? | A |
| 171 | Pak, K. S., S.;Shin, S.;Nam, H. Y.;De Maeyer, S.;Nummenmaa, L. | 2024 | Glucose metabolism and Radiodensity of Abdominal Adipose Tissue: A 5-year longitudinal study in a large PET cohort | A |
| 172 | Park, H. J., S. Y.;Han, M. K.;Jang, Y.;Moon, Y. R.;Kim, T.;Shin, S. Y.;Hwang, H. | 2024 | Lowering Barriers to Health Risk Assessments in Promoting Personalized Health Management | A |
| 173 | Park, J. H. P., D. J.;Kim, H.;Park, H.;Nam, H.;Lee, B.;Kim, J.;Cho, Y. S.;Kong, S. H.;Lee, H. J.;Yang, H. K. | 2024 | Long-term impact of weight loss in people with class II obesity on the overall burden of disease: evidence from the national health screening cohort in Korea | A |
| 174 | Parker, S. T., A.;Saito, S.;McNamara, C.;Denney-Wilson, E.;Nutbeam, D.;Harris, M. F.;Heal e-Literacy Prevention, Gen | 2024 | Exploring organisational readiness to implement a preventive intervention in Australian general practice for overweight and obese patients: key learnings from the HeLP-GP trial | A |
| 175 | Pearce, A. H., P.;Katikireddi, S. V.;Dundas, R.;Leyland, A. H.;Nicholls, D.;Viner, R. M.;Fenton, L.;Hope, S. | 2024 | Childhood attention-deficit hyperactivity disorder: socioeconomic inequalities in symptoms, impact, diagnosis and medication | A |
| 176 | Pinheiro, P. S. K.-S., T.;Zhao, W.;Hernandez, D. R.;Hernandez, M. N.;Kobetz, E. N.;Caban-Martinez, A. J.;Lee, D. J. | 2024 | Distinct Prostate Cancer Survival Outcomes in Firefighters: A Population-Based Study | A |

| | | | | |
|---|---|---|---|---|
| 177 | Playford, D. A. I., A. R. | 2024 | IDENTIFYING GAPS IN DETECTION OF HEART VALVE DISEASE: A POPULATION SURVEY | A |
| 178 | Rai, R. K. B., S. | 2024 | Estimated effect of age of marriage on utilisation of India's Integrated Child Development Service programme | A |
| 179 | Runge, K. v. Z., S. K. R.;Henkens, K.;Bültmann, U. | 2024 | Metabolic syndrome and poor self-rated health as risk factors for premature employment exit: a longitudinal study among 55 016 middle-aged and older workers from the Lifelines Cohort Study and Biobank | A |
| 180 | Sahatqija, F. H., M.;Cook, S.;Kholmatova, K.;Shapkina, M.;Malyutina, S.;Kudryavtsev, A. V. | 2024 | Awareness of Hypertension, Hypercholesterolemia, and Diabetes Mellitus and Associated Characteristics in Russian Adults | A |
| 181 | Scott, N. A. C., P. E.;Jones, A. R.;Sandiford, P.;Masters-Awatere, B.;Clark, H. | 2024 | Harti Hauora Tamariki: randomised controlled trial protocol for an opportunistic, holistic and family centred approach to improving outcomes for hospitalised children and their families in Aotearoa, New Zealand | A |
| 182 | Veena Lakshmi, P. S., R.;Praveena, P. | 2024 | A Study on Impact of Smoking on Semen Parameters | A |
| 183 | Yamada, N. N., K.;Tezuka, M.;Murata, F.;Maeda, M.;Akisue, T.;Fukuda, H.;Ono, R. | 2024 | Pneumococcal vaccination coverage and vaccination-related factors among older adults in Japan: LIFE Study | A |
| 184 | Yang, X. C. Z., X. Y.;Liu, Y. H.;Liu, F. J.;Lin, H. X.;Chang, C.;Cao, W. N. | 2024 | Association between workplace health promotion service utilisation and depressive symptoms among workers: a nationwide survey | A |
| 185 | Ye, P. P., J.;Jin, Y.;Duan, L.;Yao, Y.;Ivers, R.;Keay, L.;Tian, M. | 2024 | Using a participatory design to develop an implementation framework for integrating falls prevention for older people within the Chinese primary health care system | A |
| 186 | Chatterjee, A. S. K., D. | 2023 | Screening for COVID-19 among Workers Attending Medical Examinations Under Occupational Health Service of an Open Cast Iron Ore Mine in Jharkhand: A Cross-Sectional Study | B |
| 187 | Getahun, G. K. A., M.;Keleb, G.;Shiferaw, A.;Bezabih, D. | 2023 | Assessment of routine medical checkups for common noncommunicable diseases and associated factors among healthcare professionals in Addis Ababa, Ethiopia, in 2022 a cross-sectional study | B |
| 188 | Ikegami, K. A., H.;Baba, H.;Sekoguchi, S.;Yoshitake, H.;Sugano, R.;Nozawa, H.;Hasegawa, M.;Ogami, A. | 2023 | [Evaluation of self-administered questionnaire items used in regular health check-ups at the workplace by occupational health professionals] | B |
| 189 | Lange, A. E. M.-N., J.;Pierdant, G.;Allenberg, H.;Heckmann, M.;Ittermann, T. | 2023 | Antenatal Care and Health Behavior of Pregnant Women-An Evaluation of the Survey of Neonates in Pomerania | B |
| 190 | McNeil, K. A., Jillian;Lawson, Beverley;Delahunty-Pike, Alannah;Barber, Brittany;Diepstra, Heidi | 2023 | Towards developing an intervention to support periodic health checks for adults with intellectual and developmental disabilities: Striving for health equity | B |

| | | | | |
|---|---|---|---|---|
| 191 | Michel, M. S., M. A.;Dindorf, C.;Danguy, V.;Chevreul, K. | 2023 | Health check-ups for the French under-consuming agricultural population: A pilot evaluation of the<i> Instants</i><i> sante</i><i> MSA</i> program | B |
| 192 | Yun, B. O., J.;Choi, J.;Rozek, L. S.;Park, H.;Sim, J.;Kim, Y.;Lee, J.;Yoon, J. H. | 2023 | Socioeconomic Disparities in the Association Between All-Cause Mortality and Health Check-Up Participation Among Healthy Middle-Aged Workers: A Nationwide Study | B |
| 193 | Baek, S.-U. L., Yu-Min;Yoon, Jin-Ha | 2024 | Association between long working hours and engagement in preventive healthcare services in Korean workers: Findings from the Korean National Health and Nutrition Examination Survey | B |
| 194 | Davies, J. R., A.;Buckley, C.;Crane, L.;Smalley, K. | 2024 | 'It seems like a luxury to be able to offer that': Factors influencing the implementation of annual health checks for autistic people in England | B |
| 195 | Johnson, C. F. I., F.;Thomson, F.;Srireddy, P.;Jani, B. D.;Greenlaw, N. | 2024 | General practice pharmacist-led antipsychotic physical health monitoring: a prospective intervention scoping study | B |
| 196 | Lunsky, Y. V., Tiziana;St. John, Laura;Thakur, Anupam;Lake, Johanna | 2024 | Evaluation of a co-designed health check-in for adults with intellectual and developmental disabilities and family caregivers to support pandemic recovery | B |
| 197 | Matias, M. A. J., R.;Aragón, M. J.;Fernandes, L.;Gutacker, N.;Siddiqi, N.;Kasteridis, P. | 2024 | Assessing the uptake of incentivised physical health checks for people with serious mental illness | B |
| 198 | Matsuo, R. I., T.;Takamori, A.;Kishi, T.;Minami, M.;Miyakawa, J.;Yoshitake, N.;Hayashi, A.;Nakayama, Y.;Egashira, N.;Teramoto, M.;Ishinari, H.;Kajiyama, I.;Fujisaki, S.;Kakiyama, H.;Satou, K.;Nakafusa, R.;Tanaka, C.;Tanaka, M.;Isomura, Y.;Izumi, K.;Ohta, S.;Souta, N.;Matsuo, N.;Yamamoto, K.;Tsuji, S.;Umemura, T.;Takagi, K.;Fujimoto, K. | 2024 | Improvement trend for individual health guidance intervention according to Japan clinical guidelines by public health nurses for type 2 diabetes mellitus who visited for medical checkups regularly: a case-control preliminary report | B |
| 199 | McNeil, K. A., J.;Lawson, B.;Delahunty-Pike, A.;Barber, B.;Diepstra, H. | 2024 | Towards developing an intervention to support periodic health checks for adults with intellectual and developmental disabilities: Striving for health equity | B |
| 200 | Selänne, L. P., M.;Aslan, F.;Pakarinen, A. | 2024 | Gamified Intervention for Health Promotion of Families in Child Health Clinics - A Cluster Randomised Trial | B |
| 201 | Chapman, N. O., P.;Bonner, C.;Nelson, M. R.;Sharman, E. | 2023 | Uptake of the Australian Heart Health Check before and after the COVID-19 pandemic outbreak | C |
| 202 | Christoffersen, N. B. N., F. E.;Thilsing, T.;Larsen, L. B.;Ostergaard, J. N.;Broholm-Jorgensen, M. | 2023 | Exploring targeted preventive health check interventions - a realist synthesis | C |
| 203 | Fazal, F. S., H. A.;Gondal, M. F.;Tanveer, U.;Haider, M.;Sabah, N. U.;Shahzad, F.;Rehman, M. E. U. | 2023 | Attitudes and Factors Determining the Practice of Routine Medical Checkups in the People of Rawalpindi, Pakistan: A Cross-Sectional Study | C |

| | | | | |
|---|---|---|---|---|
| 204 | Ito, N. S., H.;Odajima, T.;Yoshimura, N.;Muto, S.;Hirao, M.;Ninohei, M.;Nakayama, T. | 2023 | Factors Associated with Refraining from Health Checkups during the COVID-19 Pandemic in Japan | C |
| 205 | Kurita, A. N., Y. | 2023 | [Health check-up results, death, and occurrence of the need for nursing care among Japanese older adults: Analysis using the Kokuho Database system] | C |
| 206 | Mithrason, A. T. T., V. | 2023 | Health Seeking Behaviour among Tribal Population of Shekharakund Colony, Wayanad, Kerala | C |
| 207 | Mori, Y. M., K.;Inoue, K.;Fukuma, S. | 2023 | Patterns and predictors of adherence to follow-up health guidance invitations in a general health check-up program in Japan: A cohort study with an employer-sponsored insurer database | C |
| 208 | Ogita, M. O., M.;Katayose, R.;Miyamatsu, N.;Arai, H. | 2023 | Participation in health and frailty check-ups predicted functional outcomes and mortality in older adults in Japan | C |
| 209 | Ogunlayi, F. C.-L., N.;Hughes, D.;Myers, P.;Sitch, A. | 2023 | A cross-sectional study examining the equitability of invitation, uptake and coverage for NHS Health Check | C |
| 210 | Yamaguchi, S. A., T.;Okada, A.;Nasu, S.;Yamauchi, T.;Arase, Y.;Aizawa, T.;Nangaku, M.;Kadowaki, T. | 2023 | Impact of the COVID-19 Pandemic on Health Check-ups: A Nationwide Questionnaire Survey in 639 Healthcare Facilities in Japan Society of Ningen Dock | C |
| 211 | Ai Theng, C. | 2024 | Factors influencing the public's decision-making to undergo health checks for prevention of cardiovascular disease | C |
| 212 | Arulselvan, G. C., S.;George, N.;Rizvana, S.;Narayan, P.;Annamalai, P.;Vadakaraiyan, P. H. R.;Rajagopal, N.;Dharmaraj, R. B.;Tamilarasan, M. | 2024 | Preventive Health Checkup: Utilization, Motivators, and Barriers Among the General Population in a Rural District in Tamil Nadu, India | C |
| 213 | Cowap, L. R., V.;Grogan, S.;Ellis, N. J.;Crone, D.;Cottrell, E.;Chambers, R.;Clark-Carter, D.;Gidlow, C. J. | 2024 | They are saying it's high, but I think it's quite low: exploring cardiovascular disease risk communication in NHS health checks through video-stimulated recall interviews with patients - a qualitative study | C |
| 214 | McCracken, C. R.-E., Z.;Szabo, L.;Robson, J.;Raman, B.;Topiwala, A.;Roca-Fernández, A.;Husain, M.;Petersen, S. E.;Neubauer, S.;Nichols, T. E. | 2024 | NHS Health Check attendance is associated with reduced multiorgan disease risk: a matched cohort study in the UK Biobank | C |
| 215 | Shoji, A. K., K.;Murashita, K.;Nakaji, S.;Igarashi, A. | 2024 | Reduction in all-cause medical and caregiving costs through innovative health awareness projects in a rural area in Japan: a retrospective cohort study | C |
| 216 | Tayoun, A. A. | 2024 | Determinants of Periodic Health Examination Uptake: Insights from a Jordanian Cross-Sectional Study | C |
| 217 | Wald, N. J. H., A. D.;Vale, S. H.;Bestwick, J. P.;Morris, J. | 2024 | Comparing screening based on the NHS Health Check and Polypill Prevention Programmes in the primary prevention of heart attacks and strokes | C |

| | | | | |
|---|---|---|---|---|
| 218 | Fajardo, M. A. B., C.;Ayre, J.;McKinn, S.;Knight, J.;Raffoul, N.;Brims, K.;Nelson, A. J.;Bonner, C. | 2024 | Could nudges reduce health literacy disparities in CVD prevention? An experiment using alternative messages for CVD risk assessment screening | D |
| 219 | Bahreini, M. S. S., S.;Badalzadeh, Y.;Motazedian, M. H.;Shirani, M.;Jahromi, S. S.;Teimouri, A.;Agholi, M.;Asgari, Q. | 2023 | Molecular diagnosis of Trichomonas vaginalis in liquid-based Papanicolaou samples in Shiraz, southern Iran | Duplicate |
| 220 | Bhamani, A. H., C.;Bojang, F.;Quaife, S. L.;Dickson, J. L.;Tisi, S.;Hall, H.;Verghese, P.;Creamer, A.;Prendecki, R.;McCabe, J.;Gyertson, K.;Bowyer, V.;El-Emir, E.;Cotton, A.;Mehta, S.;Levermore, C.;Mullin, A. M.;Teague, J.;Farrelly, L.;Nair, A.;Devaraj, A.;Hackshaw, A.;Janes, S. M. | 2023 | The SUMMIT Study: Utilising a written 'Next Steps' information booklet to prepare participants for potential lung cancer screening results and follow-up | Duplicate |
| 221 | Choi, E. Y. P., J. S.;Min, D.;Ahn, S.;Ahn, J. A. | 2023 | Heart Failure-Smart Life: a randomized controlled trial of a mobile app for self-management in patients with heart failure | Duplicate |
| 222 | Coombs, N. M. S., L.;Jackson, M. R.;Borgelt, K.;Lee, J.;Porter, J. E. | 2023 | Student-led pop-up health check clinics: innovative health prevention strategy for a low socioeconomic community | Duplicate |
| 223 | Dickson, J. L. H., H.;Horst, C.;Tisi, S.;Verghese, P.;Mullin, A. M.;Teague, J.;Farrelly, L.;Bowyer, V.;Gyertson, K.;Bojang, F.;Levermore, C.;Anastasiadis, T.;McCabe, J.;Navani, N.;Nair, A.;Devaraj, A.;Hackshaw, A.;Quaife, S. L.;Janes, S. M. | 2023 | Uptake of invitations to a lung health check offering low-dose CT lung cancer screening among an ethnically and socioeconomically diverse population at risk of lung cancer in the UK (SUMMIT): a prospective, longitudinal cohort study | Duplicate |
| 224 | Fazal, F. S., H. A.;Gondal, M. F.;Tanveer, U.;Haider, M.;Us Sabah, N.;Shahzad, F.;Ur Rehman, M. E. | 2023 | Attitudes and Factors Determining the Practice of Routine Medical Checkups in the People of Rawalpindi, Pakistan: A Cross-Sectional Study | Duplicate |
| 225 | Guo, H. Z., J.;Chen, H.;Cao, X. | 2023 | Prevalence and associated factors of poor sleep quality among Chinese retirees: A multicenter cross-sectional study | Duplicate |
| 226 | Hidaka, Y. T., T.;Maruta, M.;Makizako, H.;Ikeda, Y.;Nakamura, A.;Han, G.;Miyata, H.;Shimokihara, S.;Akasaki, Y.;Kamasaki, T.;Kubozono, T.;Ohishi, M. | 2023 | Relationship between grave visitation and apathy among community-dwelling older adults | Duplicate |
| 227 | Kotti, T. K., Evangelos;Ruparel, Mamta;McEwen, Andy;Dickson, Jennifer L.;Duffy, Stephen W.;Waller, Jo;Janes, Samuel M.;Quaife, Samantha L. | 2023 | A randomised controlled trial testing acceptance of practitioner-referral versus self-referral to stop smoking services within the Lung Screen Uptake Trial | Duplicate |
| 228 | Ku, P. W. S., A.;Lai, Y. J.;Yen, Y. F.;Ahmadi, M.;Inan-Eroglu, E.;Wang, S. F.;Chen, L. J.;Stamatakis, E. | 2023 | Are associations of leisure-time physical activity with mortality attenuated by high levels of chronic ambient fine particulate matter (PM2.5) in older adults? A prospective cohort study | Duplicate |
| 229 | Raffoul, N. B., K.;Zeng, J.;Knight, J.;Asham, A.;Mitchell, J. A.;Jennings, G.;Bonner, C. | 2023 | Feasibility of a text-mediated recall system to increase cardiovascular disease risk assessment in general practice: Mixed-methods pilot evaluation | Duplicate |
| 230 | Szawlowski, S. T., C.;Lagarde, M.;Mbaye, E. H.;Gueye, K.;Ndour, C. T.;Lépine, A. | 2023 | Reforming the registration policy of female sex workers in Senegal? Evidence from a discrete choice experiment | Duplicate |

| | | | | |
|---|---|---|---|---|
| 231 | Wang, Y. Y., T.;Deng, S.;Zhu, X.;Deng, Y.;Liu, X.;Liu, L.;Wang, C. | 2023 | Metabolic health phenotype better predicts subclinical atherosclerosis than body mass index-based obesity phenotype in the non-alcoholic fatty liver disease population | Duplicate |
| 232 | Williams, P. J. P., K. E. J.;Gill, N. K.;Flannery, D.;Buttery, S.;Bartlett, E. C.;Devaraj, A.;Kemp, S. V.;Addis, J.;Derbyshire, J.;Chen, M.;Morris, K.;Laverty, A. A.;Hopkinson, N. S. | 2023 | Immediate, Remote Smoking Cessation Intervention in Participants Undergoing a Targeted Lung Health Check: Quit Smoking Lung Health Intervention Trial, a Randomized Controlled Trial | Duplicate |
| 233 | Williams, P. J. P., K. E. J.;Buttery, S. C.;Perkins, A.;Chan, L.;Bartlett, E. C.;Devaraj, A.;Kemp, S. V.;Addis, J.;Derbyshire, J.;Chen, M.;Polkey, M. I.;Laverty, A. A.;Hopkinson, N. S. | 2023 | Immediate smoking cessation support during lung cancer screening: long-term outcomes from two randomised controlled trials | Duplicate |
| 234 | Zhao, D. L., Jingjing;Li, Jie;Gao, Tingting;Fu, Peipei;Wang, Yi;Zhou, Chengchao | 2023 | Tooth loss, body mass index and cognitive function among middle-aged and older adults in China: Does gender matter? | Duplicate |
| 235 | Andersson, N. W., T.;Nylander, E.;Idahl, A. | 2024 | Seroprevalence of sexually transmitted infections over 44 years – A cross-sectional study in Sweden | Duplicate |
| 236 | Baek, S. U. L., Y. M.;Yoon, J. H. | 2024 | Association between long working hours and engagement in preventive healthcare services in Korean workers: Findings from the Korean National Health and Nutrition Examination Survey | Duplicate |
| 237 | Cai, G. X. L., Y. F.;Zhuang, J. M.;Chen, Z. S.;Lu, Y. X.;Wu, J. W.;Hu, Z. J.;Zhang, J. P.;He, F. | 2024 | Differences in socio-demographics status, risk behaviours, healthcare uptake and HIV/ sexually transmitted infections (STIs) between brothel-based and street-based female sex workers in Yunnan, China | Duplicate |
| 238 | Landgren, V. S., Leif;Törnhage, Carl-Johan;Theodosious, Michail;Gillberg, Christopher;Johnson, Mats;Knez, Rajna;Landgren, Magnus | 2024 | Neurodevelopmental problems, general health and academic achievements in a school-based cohort of 11-year-old Swedish children | Duplicate |
| 239 | Maltz, A. R., S.;Sarid, A.;Cohen, Y.;Landau, T.;Saifer, E.;Amorai Belkin, N.;Alcalay, T. | 2024 | The Framing Effect of Digital Textual Messages on Uptake Rates of Medical Checkups: Field Study | Duplicate |
| 240 | Scott, N. C., P. E. A.;Jones, A. R.;Sandiford, P.;Masters-Awatere, B.;Clark, H. | 2024 | Harti Hauora Tamariki: randomised controlled trial protocol for an opportunistic, holistic and family centred approach to improving outcomes for hospitalised children and their families in Aotearoa, New Zealand | Duplicate |
| 241 | Ye, P. P. P., J. Y.;Jin, Y.;Duan, L. L.;Yao, Y.;Ivers, R.;Keay, L.;Tian, M. Y. | 2024 | Using a participatory design to develop an implementation framework for integrating falls prevention for older people within the Chinese primary health care system | Duplicate |

**Literature search update: May 2024**
**Records assessed at full text level and the excluded reasons**

| # | Author | Year | Title | Excluded with reason |
|---|---|---|---|---|
| 1 | Chin, S. R., C. L.;Gardner, A.;Nazar, H. | 2024 | The impact and user experience of a student-led clinic providing preventative services | C |
| 2 | Coombs, N. M. S., L.;Jackson, M. R.;Borgelt, K.;Lee, J. S. C.;Porter, J. E. | 2023 | Student-led pop-up health check clinics: innovative health prevention strategy for a low socioeconomic community | E |
| 3 | Junghans, C. A., G.;Williams, A.;Harris, M. | 2023 | Learning from the universal, proactive outreach of the Brazilian Community Health Worker model: impact of a Community Health and Wellbeing Worker initiative on vaccination, cancer screening and NHS health check uptake in a deprived community in the UK | G |
| 4 | Raffoul, N. B., K.;Zeng, J.;Knight, J.;Asham, A.;Mitchell, J. A.;Jennings, G.;Bonner, C. | 2023 | Feasibility of a text-mediated recall system to increase cardiovascular disease risk assessment in general practice <i>Mixed</i>-<i>methods pilot evaluation</i> | G |
