# Supplementary material for: The effectiveness of interventions used to improve general health check uptake by the older adult population: a systematic review and meta-analysis
Source: PLOS Glob Public Health. 2025 Mar 31;5(3):e0004362. doi: 10.1371/journal.pgph.0004362 (PMC11957279; doi:10.1371/journal.pgph.0004362)
Supplement: S4 Appendix — (DOCX) [file pgph.0004362.s004.docx]

S4 Appendix. Identified BCTs in each intervention with illustrative quotes.

| **Author (year)** | **Intervention(s)** | **Intervention description/ illustrative quotation** ^a^ | **BCTs** ^b^ | **Intervention functions** |
| --- | --- | --- | --- | --- |
| Gidlow et al., 2019 | Telephone calls | - *"Can I book you in for an appointment?"* | 3.1 Social support (unspecified) | - Enablement |
|  |  | - *"Would you be interested in a free NHS Health Check?"* | 7.1 Prompts/ cues | - Environmental restructuring - Enablement |
|  |  | - *"I am calling to invite you for your free NHS Health Check. Patients aged between 40 and 74 are entitled to a free NHS Health Check to assess their risk of developing heart disease, stroke, kidney disease or diabetes. If there are any warning signs, then together we can do something about it."* | 5.1 Information about health consequences | - Education |
|  | Risk-personalised letter | - *“The risk-personalised letters were developed to include messages appropriate for different levels of CVD risk based on patient’s % 10-year risk score (QRISK®2). Three letter templates were developed according to risk category: high ≥20%; medium 10–19.9%; low < 10%.”* | 5.1 Information about health consequences | - Education - Persuasion |
| Gold et al., 2019 | Loss-framed leaflet | - *"Family history may increase risk, but taking action now can help you to prevent the onset of these conditions."* | 5.1 Information about health consequences | - Education |
|  |  | - *"Preventative checks are cheaper for the NHS than treating disease that has already developed."* | 5.3 Information about social and environmental consequences | - Education |
|  |  | - *"6 million people have already attended."* | 6.2 Social comparison | - Persuasion |
|  |  | - *"The NHS Health Check can reduce your chances of developing certain conditions. Many people find it beneficial. However, it is your choice whether or not you take it up."* | 6.3 Information about others’ approval | - Persuasion |
|  |  | - *"The other side of the loss-framed leaflet was a flowchart like that on the gain-framed leaflet, except that instead of having the statistics at the end, it had traffic-light coloured faces with appropriate emotional expressions to reinforce the personalised consequences of the NHS Health Check: ‘Not attending: You might be at risk of stroke. If you don’t know, you can’t do anything about it’, ‘Ignoring: You can’t ignore diabetes. Don’t ignore your NHS Health Check’, and ‘Attending: Your NHS Health Check could help prevent dementia, type 2 diabetes and more.’"* | 9.3 Comparative imagining of future outcomes | - Enablement |
|  |  | - *“I know I eat too much, don’t do enough exercise and smoke’. We are not here to judge you. Our advice is tailored to you personally and what you feel you can do. Small steps will help improve your health."* - *"But don’t these conditions run in the family? Family history may increase risk, but taking action now can help you to prevent the onset of these conditions."* | 13.2 Framing/ reframing | - Persuasion |
|  |  | - *“Although you feel fine, you could get diabetes, heart disease, kidney disease or dementia. Did you know they can be prevented, even if they run in your family? Doing nothing could lead to complications. Getting check could help you and help the NHS...”* | 15.1 Verbal persuasion about capability | - Persuasion |
|  | Gain-framed leaflet | - *"NHS Health Checks aim to prevent 1,600 people from heart attacks and stroke per year"* - *"Your NHS Health Check reduces your risk of developing dementia"* - *"NHS Health Checks could prevent you from developing type 2 diabetes or kidney disease"* | 5.1 Information about health consequences | - Education |
|  |  | - *"Won’t this cost the NHS money? No. Preventative checks are cheaper for the NHS than treating disease that has already developed."* | 5.3 Information about social and environmental consequences | - Education |
|  |  | - *"6 million people have already attended."* | 6.2 Social comparison | - Persuasion |
|  |  | - *"The NHS Health Check can reduce your chances of developing certain conditions. Many people find it beneficial. However, it is your choice whether or not you take it up."* | 6.3 Information about others’ approval | - Persuasion |
|  |  | - *“I know I eat too much, don’t do enough exercise and smoke’. We are not here to judge you. Our advice is tailored to you personally and what you feel you can do. Small steps will help improve your health."* - *"But don’t these conditions run in the family? Family history may increase risk, but taking action now can help you to prevent the onset of these conditions."* | 13.2 Framing/ reframing | - Persuasion |
| Gold et al., 2021 | Point-of-care prompt to clinical staff | - *"If the member of staff moved the cursor over the prompt, then they were given the instruction, ‘Please offer the patient an appointment for their free NHS Health Check’."* | 3.1 Social support (unspecified) | - Enablement |
| McDermott et al., 2016 | QBE questionnaire + standard invitation | - *“It was also decided to add items assessing anticipated regret because a previous QBE study45 found that, among participants who returned completed questionnaires, those who completed a TPB with anticipated regret questionnaire had a significantly higher cervical cancer screening attendance rate … than those who received a TPB-only questionnaire ...”* | 5.5 Anticipated regret | - Coercion |
|  |  | - *“In terms of the QBE, cognitive dissonance can arise when completing a questionnaire leads individuals to realise that their current or past actions are incompatible with their beliefs about how they should act.”* | 13.3 Incompatible belief | - Coercion |
|  | QBE questionnaire + incentive + standard invitation | - *“It was also decided to add items assessing anticipated regret because a previous QBE study45 found that, among participants who returned completed questionnaires, those who completed a TPB with anticipated regret questionnaire had a significantly higher cervical cancer screening attendance rate … than those who received a TPB-only questionnaire ...”* | 5.5 Anticipated regret | - Coercion |
|  |  | - *“The covering letter in this trial arm offered the £5 retail voucher as an incentive to return the questionnaire.”* | 10.1 Material reward (behaviour) | - Incentivisation |
|  |  | - *“In terms of the QBE, cognitive dissonance can arise when completing a questionnaire leads individuals to realise that their current or past actions are incompatible with their beliefs about how they should act.”* | 13.3 Incompatible belief | - Coercion |
| Sallis et al., 2016 | Enhanced invitation letter | - *"completing the tear off slip"* | 1.4 Action planning | - Enablement |
|  |  | - *"asking recipients to stick the slip to their fridge"* | 7.1 Prompts/ cues | - Environmental restructuring |
| Sallis, Gold, et al., 2019 | Sunk-cost letter | - *"Phone us on XXX XXXX XXXX to book your appointment"* | 4.1 Instruction on how to perform the behaviour | - Training |
|  |  | - *"Your NHS Health Check is now due.”* - *Phone us on XXX XXXX XXXX to book your appointment"* | 7.1 Prompts/ cues | - Environmental restructuring |
|  | Counterargument letter | - *“…explaining that the NHSHC can help to prevent the development of serious health conditions which take up lots of NHS resource…”* | 5.1 Information about health consequences | - Education |
|  |  | - *"Your GP says: I want you to attend the NHS Health Check, as it can help prevent you developing more serious conditions which will take up more NHS resources."* - *"Your GP says: Family history plays only a small role. For example, most of the causes of heart attacks are related to how much you look after your body."* | 9.1. Credible sources | - Persuasion |
|  |  | - *“Phone us on XXX XXXX XXXX to book your appointment”* | 4.1 Instruction on how to perform the behaviour | - Training |
|  |  | - *"Excuse: There’s nothing I can do about my family history of illness.”* - *“Your GP says: Family history plays only a small role. For example, most of the causes of heart attacks are related to how much you look after your body."* - *"Excuse: I don’t want to bother the NHS”* - *“Your GP says: I want you to attend the NHS Health Check, as it can help prevent you developing more serious conditions which will take up more NHS resources."* | 13.2 Framing/ reframing | - Persuasion - Enablement |
| Sallis, Sherlock, et al., 2019 | Open-ended letter | - *"I am going to my NHS Health Check on __/__/13 at _____am/pm"* | 1.4 Action planning | - Enablement |
|  |  | - *"Please call<to be inserted by mail merge> to book your appointment at your GP’s surgery and record this on the tear off slip below."* - *To book, please ring 0203 4039 9999 and quote ‘NHS Health Check’."* | 4.1 Instruction on how to perform the behaviour | - Training Enablement |
|  |  | - *"Please record the date and time of your appointment and stick it on your fridge."* | 7.1 Prompts/ cues | - Environmental restructuring |
|  | Time-limited letter | - *“I am going to my NHS Health Check on __/__/13 at _____am/pm"* | 1.4 Action planning | - Enablement |
|  |  | - *"Please call <to be inserted by mail merge> as soon as possible to make sure you get your appointment at your GP’s surgery and record this on the tear off slip below."* - *"To book, please ring 0203 4039 9999 and quote ‘NHS Health Check’."* | 4.1 Instruction on how to perform the behaviour | - Training |
|  |  | - *"Please record the date and time of your appointment and stick it on your fridge."* | 7.1 Prompts/ cues | - Environmental restructuring |
|  | Social-norm letter | - *"Please call <to be inserted by mail merge>to book your appointment at your GP’s surgery.  You can also have your health check at your local pharmacy listed in the enclosed leaflet. To book, please ring 0203 4039 9999 and quote ‘NHS Health Check’."* | 4.1 Instruction on how to perform the behaviour | - Training |
|  |  | - *"In Southwark, thousands of people like you have attended their health check and benefited from personalised health advice. "* - *"How an NHS Health Check helped me “My cholesterol was a little high, but I got peace of mind regarding my state of health generally. Since the health check, I’ve cut down on alcohol and made some small changes to my diet”"* | 6.2 Social comparison | - Persuasion |
|  | Reminder SMS | - *"Your GP recently sent you a letter inviting you to attend your NHS Health Check. Call xxxxxxxxx to book an appt."* | 4.1 Instruction on how to perform the behaviour | - Training - Enablement |
| Shimoda et al., 2022 | Postal reminder with clinical service information | - *"The intervention group received letters with information on the available clinics that were the nearest to participants' addresses,"* | 1.4 Action planning | - Enablement |

SMS = short message service; BCT = behaviour change technique

^a^ Intervention description were extracted from the published paper or from the published supplementary file online.

^b^ The number labelling each behaviour change technique follows the behaviour change technique taxonomy (version 1).
