# Supplementary material for: The effectiveness of interventions used to improve general health check uptake by the older adult population: a systematic review and meta-analysis
Source: PLOS Glob Public Health. 2025 Mar 31;5(3):e0004362. doi: 10.1371/journal.pgph.0004362 (PMC11957279; doi:10.1371/journal.pgph.0004362)
Supplement: S5 Appendix — (DOCX) [file pgph.0004362.s005.docx]

S5 Appendix. Linking BCTs to the identified intervention functions in the BCW.

| **Intervention function** | **Most used BCTs** | **Less frequently used BCTs** |
| --- | --- | --- |
| **Education** |  |  |
| Increase knowledge or understanding | - 5.1 Information about health consequences | - 6.3 Information about others’ approval |
|  | - 5.3 Information about social and environmental consequences |  |
|  | - 7.1 Prompt/ Cue |  |
| **Persuasion** |  |  |
| Use communication to induce positive or negative feeling to stimulate action | - 5.1 Information about health consequence | - 6.2 Social comparison |
|  | - 5.3 Information about social and environmental consequences | - 6.3 Information about others’ approval |
|  | - 9.1 Credible source | - 13.2 Framing/ reframing |
|  |  | - 15.1 Verbal persuasion about capability |
| **Coercion** |  |  |
| Create an expectation of punishment or cost |  | - 5.5 Anticipated regret |
|  |  | - 13.3 Incompatible beliefs |
| **Incentivization** |  |  |
| Create an expectation of reward |  | - 10.1 Material incentive (behaviour) |
| **Training** |  |  |
| Impart skills | - 4.1 Instruction on how to perform the behaviour |  |
| **Environment restructuring** |  |  |
| Change the physical or social context | - 7.1 prompt/ cues |  |
| **Enablement** |  |  |
| Increase means or reduce barriers to increase capability (beyond education or training) or opportunity (beyond environmental restructuring) | - 1.4 Action planning | - 5.5 Anticipated regret |
|  | - 3.1 Social Support (unspecified) | - 9.3 Comparative imagining of future outcomes |
|  |  | - 13.2 Framing/ reframing |
|  |  | - 13.3 Incompatible beliefs |
|  |  | - 15.1 Verbal persuasion about capability |

All the definitions ere extracted from Michie et al. (2014) designing guide.
